# Radical hydrodifluoromethylation of unsaturated C−C bonds via an electroreductively triggered two-pronged approach

**DOI:** 10.1038/s42004-022-00697-1

**Published:** 2022-08-11

**Authors:** Seonyoung Kim, Keon Ha Hwang, Hyeong Gyu Park, Jaesung Kwak, Hyuk Lee, Hyunwoo Kim

**Affiliations:** 1 Department of Chemistry and Nanoscience, Ewha Womans University, Seoul, 03760 Republic of Korea; 2 Infectious Diseases Therapeutic Research Center, Korea Research Institute of Chemical Technology (KRICT), Daejeon, 34114 Republic of Korea; 3 Graduate School of New Drug Discovery and Development, Chungnam University, Daejeon, 34134 Republic of Korea; 4 Department of Chemistry, Pohang University of Science and Technology (POSTECH), Pohang, 37673 Republic of Korea

**Keywords:** Synthetic chemistry methodology, Reaction mechanisms

## Abstract

Due to its superior ability in controlling pharmaceutical activity, the installation of difluoromethyl (CF_2_H) functionality into organic molecules has been an area of intensive research. In this context, difluoromethylation of C−C π bonds mediated by a CF_2_H radical have been pursued as a central strategy to grant access to difluoromethylated hydrocarbons. However, early precedents necessitate the generation of oxidative chemical species that can limit the generality and utility of the reaction. We report here the successful implementation of radical hydrodifluoromethylation of unsaturated C−C bonds via an electroreductively triggered two-pronged approach. Preliminary mechanistic investigations suggest that the key distinction of the present strategy originates from the reconciliation of multiple redox processes under highly reducing electrochemical conditions. The reaction conditions can be chosen based on the electronic properties of the alkenes of interest, highlighting the hydrodifluoromethylation of both unactivated and activated alkenes. Notably, the reaction delivers geminal (bis)difluoromethylated products from alkynes in a single step by consecutive hydrodifluoromethylation, granting access to an underutilized 1,1,3,3-tetrafluoropropan-2-yl functional group. The late-stage hydrodifluoromethylation of densely functionalized pharmaceutical agents is also presented.

## Abstract (web summary)

The installation of difluoromethylene groups into bio-relevant molecules allows access to important scaffolds, but existing methods to do so often rely on oxidative chemical species that can hamper reactivity. Here, radical hydrodifluoromethylation with a wide range of unsaturated C–C bonds is achieved via an electroreductive two-pronged approach, granting access to a variety of difluoromethylated organic molecules.

## Introduction

The replacement of hydrogen atoms with fluorine has become a quintessential approach for new chemical entities to regulate physicochemical and biological properties such as metabolic stability, lipophilicity, hydrogen bonding ability and bioavailability^1–8^. Particularly, the installation of difluoromethyl (CF_2_H) functionality instead of CH_3_ or CF_3_ groups in bio-relevant chemical structures has been an area of intensive research in drug development due to the highly polarized C−H bond of CF_2_H that serves as a bioisostere of hydrogen bonding donors such as hydroxyl, thiol, and amine groups^9–14^. Furthermore, the installation of difluoromethylene group to liquid crystals has also been considered to be important in controlling their physical properties^15,16^.

**Fig. 1:**
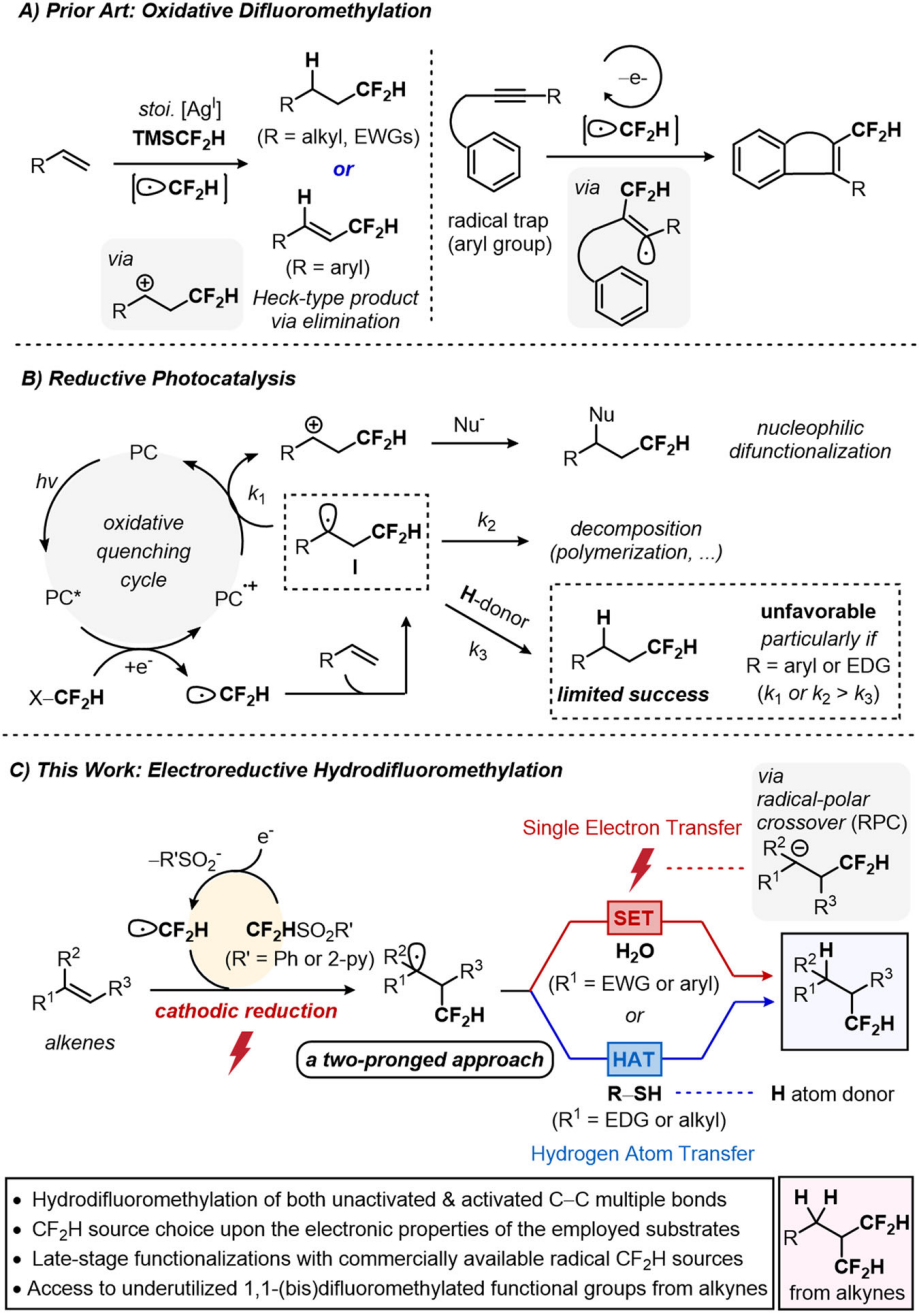
Radical hydrodifluoromethylation of unsaturated C–C bonds. **A** Precedent examples of difluoromethylation under oxidizing conditions. **B** Reductive photocatalysis for radical difluoromethylation. **C** A two-pronged electroreductive hydrodifluoromethylation.

In this context, difluoromethylative functionalization, where a difluoromethyl anion^17–26^, carbene^27–32^ or radical precursor^33–50^ is engaged in the direct transfer of the CF_2_H unit, has been vigorously pursued as a strategy with great promise in organic synthesis. Particularly, radical hydrodifluoromethylation in which CF_2_H^**•**^ and H^**•**^ equivalents add across to unsaturated C−C π bonds has become a central strategy to access a relatively limited class of aliphatic hydrocarbons that contain difluoromethyl group^51–57^. This includes the use of redox-active CF_2_H radical precursors with the alkene of interest (Fig. 1A, left). For example, CF_2_H radicals generated under oxidative conditions have frequently been utilized in hydrodifluoromethylation as well as difluoromethylative Heck-type coupling^43,51^. Additionally, several groups independently demonstrated oxidative difluoromethylative radical annulation of alkynes in the presence of aryl groups as the radical trap (Fig. 1A, right)^44,45^.

On the other hand, CF_2_H radicals generated by reductive photocatalysis^52,53^ or photosensitization^55^ have also enabled hydrodifluoromethylation of aliphatic or electron-deficient alkenes (Fig. 1B). While highly enabling, existing methods often require or involve oxidative chemical species that can presumably hamper the desired reactivity, thus limiting generality of the reaction. For example, a carbon-centered radical intermediate I, which is formed upon addition of CF_2_H radical into C=C bond can readily be sacrificially oxidized by the quenching cycle of photocatalysis to afford corresponding carbocation (*k*_1_), eventually leading to the formation of less-desirable difunctionalization products upon nucleophilic trapping^11^. Indeed, in early examples, tethered nucleophiles were requisite for the intramolecular trapping of resultant carbocation intermediate^58,59^.

In addition, inherent transiency of these radicals often led to the decomposition of the reaction intermediates particularly if R groups are aromatic or electron-donating substituents (*k*_2_)^55^. Furthermore, super-stoichiometric amounts of CF_2_H radical sources with high molecular weight has been often employed that can significantly limit potential utility of these early precedents. To address these intrinsic limitations in terms of modularity and structural diversity, the development of a mechanistically distinct and more generally valid hydrodifluoromethylation approach remains a key challenge.

Herein, we describe the successful implementation of radical hydrodifluoromethylation with a wide range of unsaturated C−C bonds via an electroreductively triggered two-pronged approach (Fig. 1C). The reaction conditions can be chosen based on electronic properties of the alkenes of interest, highlighting a hydrodifluoromethylation of both unactivated and activated alkenes. Notably, the developed protocol herein showcases unique reactivity towards alkynes, granting access to underutilized 1,1,3,3-tetrafluoropropan-2-yl functional group by a regioselective double hydrodifluoromethylation. To the best of our knowledge, this reactivity represents unique example of multiple difluoromethylation of alkynes. Furthermore, this electrochemical approach is applicable to late-stage functionalization and drug modification with use of commercially available CF_2_H sources and inexpensive H_2_O or thiophenol (PhSH) as the hydrogen sources.

## Results and discussion

**Fig. 2:**
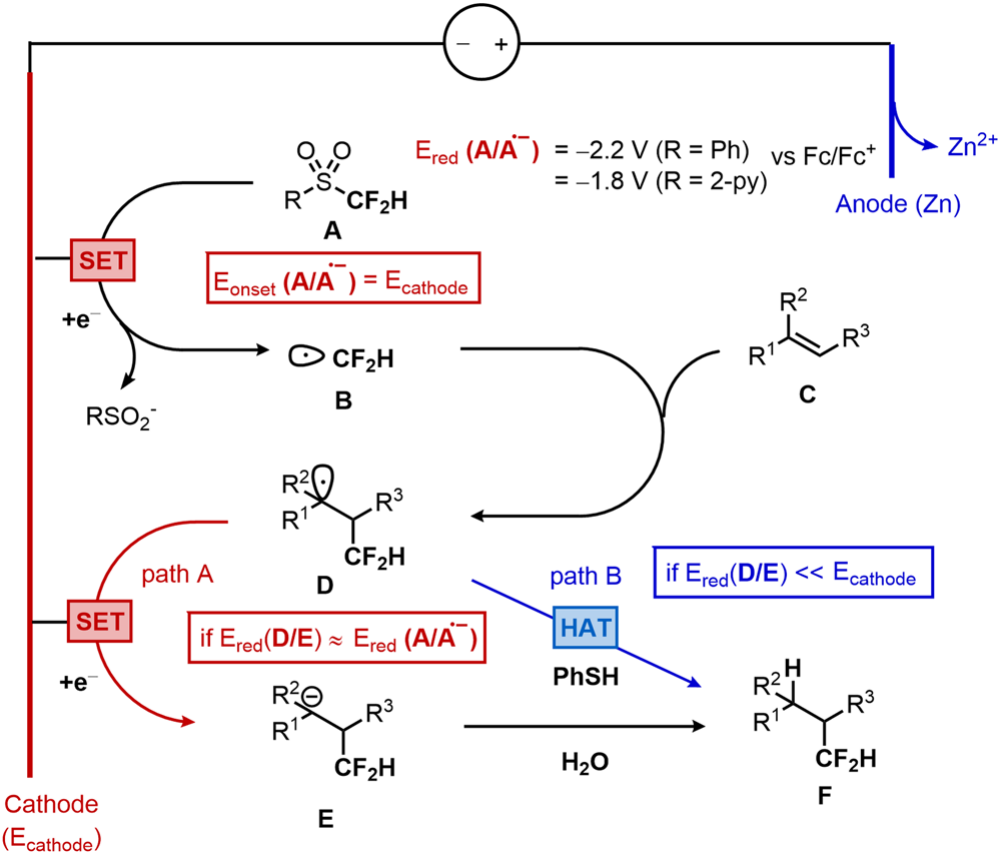
Proposed mechanism. A cathodic reduction of (**A**) would furnish a CF_2_H radical (**B**), which would afford carbon-centered radical (**D**) upon addition into alkene substrate (**C**). The radical (**D**) would be reduced into corresponding carbanion (**E**) when the reduction potential of (**D**) is on par with (**A**) (path A). A subsequent protonation with water would furnish hydrodifluoromethylation product (**F**). Alternatively, a carbon-centered radical (**D**) would directly perform hydrogen-atom transfer (HAT) to form (**F**) in the presence of a hydrogen atom donor, when E_red_(**D**/**E**) is too negative to be reduced on the cathode (path B).

Inspired by previous reports on the electrochemical or photoelectrochemical activations of strong bonds^60–73^, we anticipated electroreductive reaction conditions using a sacrificial anode^74,75^ would allow access to deeply reductive potentials and cleavage of strong bonds (Fig. 2). Thereby, a cathodic reduction of A would furnish a CF_2_H radical (B), which would afford carbon-centered radical D upon addition into alkene substrate. Two mechanistic scenarios are envisioned based upon electronic properties of the employed alkenes. This radical D would be reduced into corresponding carbanion E when the reduction potential of D [E_red_(D/E) = −2.0 V vs Fc/Fc^+^]^76^ is on par with E_red_(A/A^•**-**^) (path A, if R^1^ = EWG or Aryl). A subsequent protonation with water would furnish hydrodifluoromethylation product F, constituting an ECEC-type^62,77,78^ radical-polar crossover mechanism^69,70,79–82^. Alternatively, a carbon-centered radical D would directly perform hydrogen-atom transfer (HAT) to form F in the presence of a hydrogen atom donor such as thiol, when E_red_(D/E) is too negative to be reduced on the cathode (path B, if R^1^ = EDG or Alkyl).

**Fig. 3:**
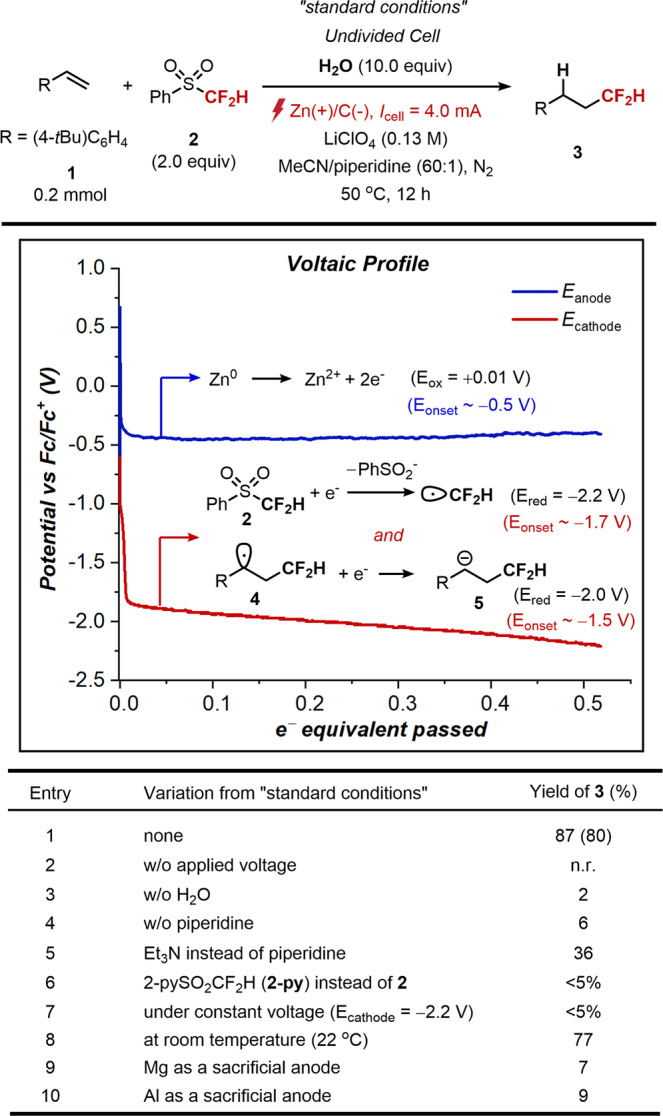
Reaction parameter optimization. Yields determined by ^1^H NMR using CH_2_Br_2_ as an internal standard (isolated yields in parenthesis). The voltaic profile of each electrode during electrolysis of entry 1 is shown (inset box). The sacrificial Zn anode operates at the anticipated onset potential for Zn oxidation (blue line, E_onset_ = ca. −0.5 V vs Fc/Fc^+^). The initial cathodic potential (E_cathode_ = −1.8 V) is in accordance with the onset potential for reduction of 2 (red line). Under the given cathodic potential, a simultaneous reduction of benzylic radical intermediate 4 into corresponding carbanion 5 is also conceivable.

To put this hypothetic system in context, we initially commenced our investigation by choosing conjugated alkene 1 as a model substrate for path A, with 2 as CF_2_H radical precursor (Fig. 3). After optimization, we observed that the application of a constant current of 4 mA in the presence of stoichiometric amounts of water enabled the formation of the desired hydrodifluoromethylation product 3 in 80% yield. The optimal conditions employed lithium perchlorate (LiClO_4_) as the electrolyte, carbon felt and zinc plate as the cathode and the sacrificial anode respectively, acetonitrile and piperidine as the co-solvent under 50 °C. To verify our initial mechanistic hypothesis, we monitored the voltaic profile of each electrode during electrolysis of entry 1 (Fig. 2, inset box). The sacrificial Zn anode operates at the anticipated onset potential for Zn oxidation (E_onset_ = ca.−0.5 V vs Fc/Fc^+^, typically 0.5 V below the thermodynamic potential)^83,84^. Likewise, the initial cathodic potential (E_cathode_ = −1.8 V) is in accordance with the onset potential for reduction of 2. Under the given cathodic potential, a simultaneous reduction of benzylic radical intermediate 4 into corresponding carbanion 5 is conceivable presumably via a radical-polar crossover mechanism [path A in Fig. 2, E_red_(4/5 $\cong$ −2.0 V)]^76^.

**Fig. 4:**
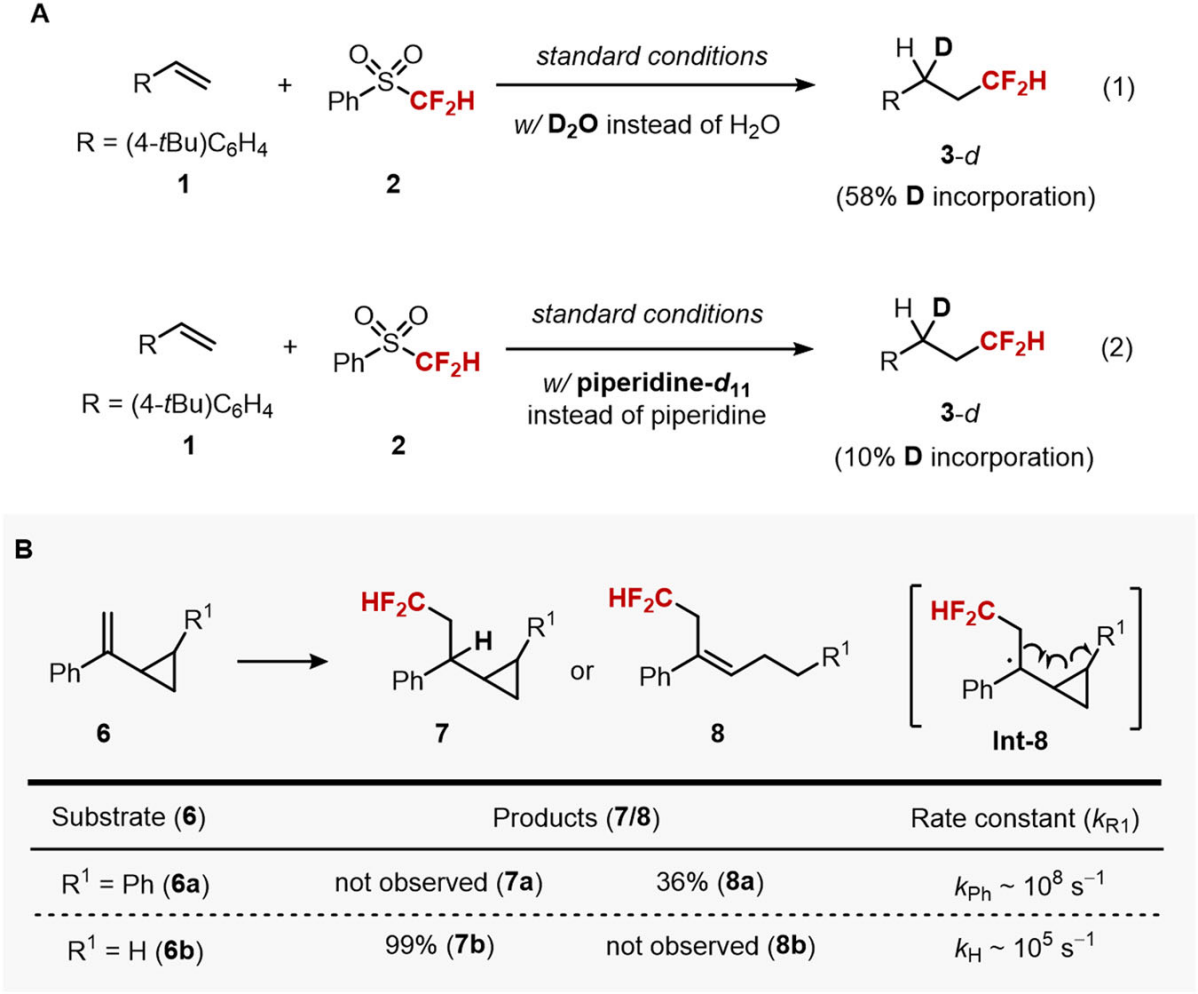
Preliminary mechanistic investigation. **A** A significant amount of deuterium incorporation was observed upon employment of D_2_O in under optimized reaction conditions (Eq. 1). The reaction with piperidine-*d*_11_ result in low deuterium incorporation (Eq. 2). **B** Vinyl cyclopropane 6a with higher ring opening rate constant (*k*_Ph_ = ~10^8 ^s^−1^) underwent rupture of the three-membered ring (8a), while the cyclopropyl ring in 6b (*k*_H_ = ~10^5 ^s^−1^) remained intact after electrolysis.

Subsequently, a set of control experiments using deuterated reaction components were conducted to verify our mechanistic hypothesis (Fig. 4A). A significant amount of deuterium incorporation was observed upon employment of D_2_O in lieu of H_2_O under optimized reaction conditions, as we envisioned at the outset (Eq. 1). On the contrast, we found that the reaction with piperidine-*d*_11_ result in low deuterium incorporation, suggesting that an alternative mechanism where an α C−H bond of amine additive is engaged in hydrogen atom transfer is less conceivable (Eq. 2)^85,86^.

Further experiments using radical probe substrates were conducted (Fig. 4B). Interestingly, vinyl cyclopropane 6a with higher ring opening rate constant due to the generation of more stable benzyl radical (*k*_Ph_ = ~10^8^ s^−1^) underwent rupture of the three-membered ring (8a), while the cyclopropyl ring in 6b (*k*_H_ = ~10^5^ s^−1^) remained intact after electrolysis under standard conditions (7b)^76^. These results imply that the reduction of the benzylic radical intermediate (Int-8) is sufficiently fast to prevent undesired side reactions, constituting radical-polar crossover mechanism (Fig. 2, path A).

**Fig. 5:**
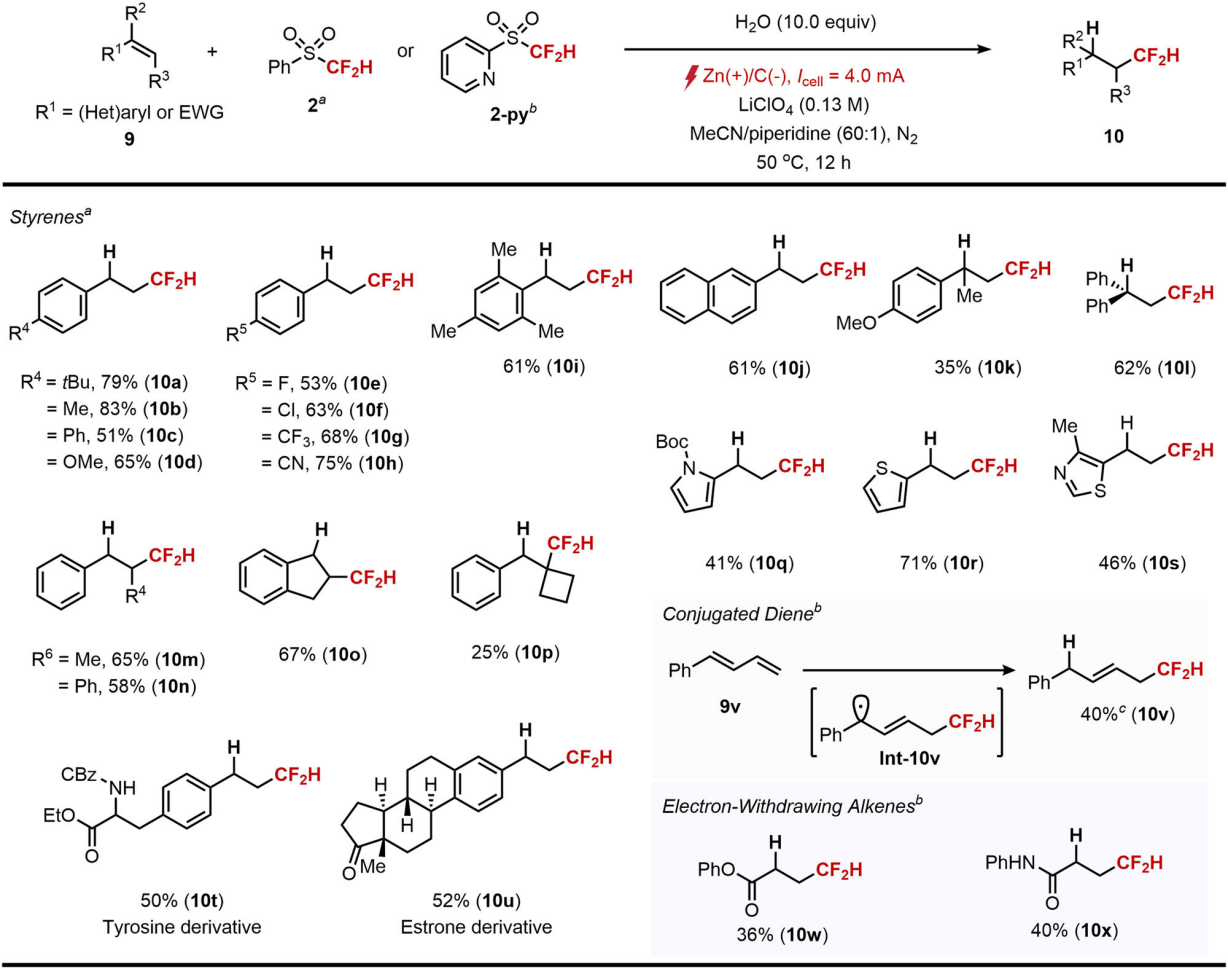
Substrate scope of conjugated alkenes^*a*^. ^*a*^9 (0.2 mmol), 2 (0.4 mmol), H_2_O (2.0 mmol), LiClO_4_ (0.8 mmol) in MeCN/piperidine (6.0 mL, 40:1) at 50 °C. ^*b*^9 (0.2 mmol), 2-py (0.6 mmol), LiClO_4_ (0.8 mmol) and H_2_O (1.0 mL) in MeCN/Et_3_N (14/1, 4.0 mL) at 22 °C. ^*c*^Yield was measured by ^1^H NMR spectroscopy with CH_2_Br_2_ as an internal standard. Isolated yields are reported unless otherwise noted.

Having identified the optimized reaction parameters, we next explored the substrate scope of conjugated alkenes (Fig. 5). A wide range of terminal styrenes that possess functional groups such as alkyls (10a−b), phenyl (10c), methoxy (10d), halides (10e−f), trifluoromethyl (10g) and cyano (10h) were well tolerated. The methyl groups in vinylmesitylene that are potentially oxidizable remained intact after electrolysis to deliver the product 10i. We found that 2-vinylnaphthalene also afforded the desired hydrodifluoromethylation product 10j in good yield. Additionally, 1,1-disubstituted styrenes such as 9k and 9l were successfully converted into the desired products in good yields. Besides terminal styrenes, more challenging internal styrenes were proved to be compatible with the current electrolytic system with exclusive regioselectivity, due to inherent stability of benzylic radicals over secondary radicals (10m−p). It was notable to see that the regioselectivity was consistent even with tertiary radicals, furnishing corresponding difluoromethylated quaternary carbon center from the trisubstituted styrene 9p. Importantly, vinyl heteroarenes such as pyrrole, thiophene and thiazole were all suitable substrates, transforming into the desired products in satisfactory yields (10q−s). Moreover, the reactivity toward biorelevant structures such as tyrosine (10t) and estrone (10u) was successfully illustrated.

We note that the choice of the radical precursor was important when highly conjugated or electron-withdrawing alkenes were subjected to the reaction. For example, 1-phenyl-1,3-butadiene (9v) was efficiently converted to 1,4-hydrodifluorodifluoromethylated product 10v in the presence of more readily reducible 2-py as a radical precursor. We assumed that less negative reaction potential modulated by the use of 2-py was key to the desired polar crossover of highly conjugated radical intermediate Int-10v. Similarly, electron-deficient alkenes were smoothly participated in the reaction to give corresponding hydrodifluoromethylation products (10w−x) under room temperature.

**Fig. 6:**
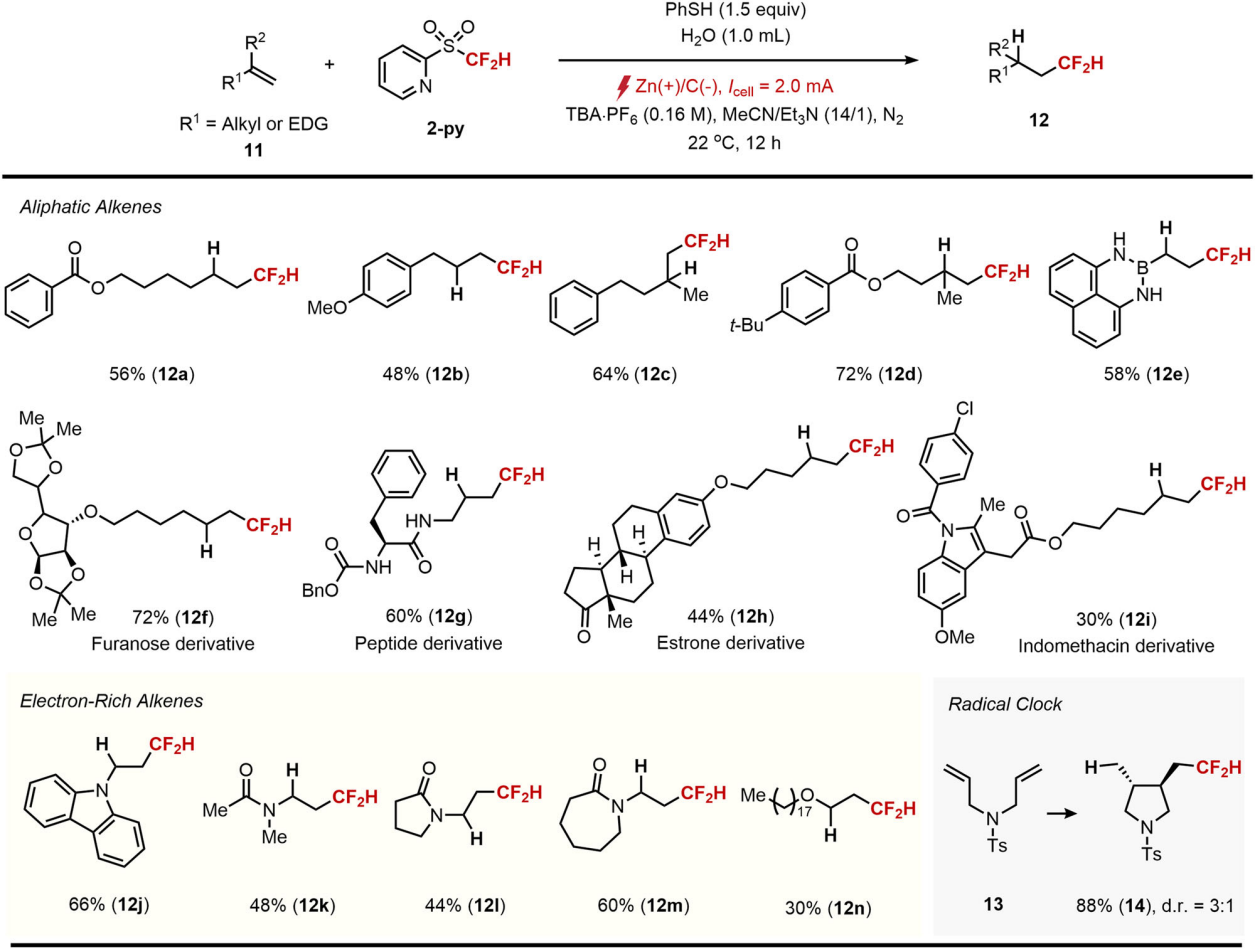
Substrate scope of aliphatic and electron-rich alkenes^*a*^. ^*a*^11 (0.2 mmol), 2-py (0.6 mmol), TBA·PF_6_ (0.8 mmol) and H_2_O (1.0 mL) in MeCN/Et_3_N (14/1, 4.0 mL) at 22 °C. Isolated yields are reported unless otherwise noted.

We further expanded the scope of the current hydrodifluoromethylation reaction with respect to the aliphatic and electron-rich alkenes on the basis of initial mechanistic hypothesis in Fig. 2, path B (Fig. 6). The plausibility of this hypothesis was verified by choosing thiophenol as a hydrogen atom donor in the presence of 2-py as a CF_2_H radical source. After optimization of the reaction parameters, we found that the desired reaction can be achieved using TBA·PF_6_ as the electrolyte with the application of a constant current of 2 mA in MeCN at room temperature. In addition, the slightly increased amounts of amine (Et_3_N) and water additives were found to be optimal in preventing a short-circuit caused by a zinc precipitation.

We have found that a wide range of terminal aliphatic alkenes was compatible with the reaction conditions (12a−i). The hydrodifluoromethylated products derived from both monosubstituted (12a−b) and 1,1-disubstituted alkenes (12c−d) gave good yields. Importantly, a difluoromethylated alkylboron product 12e could readily be obtained from a masked vinylboronic acid 11e, which can serve as a useful synthon in difluoroalkylative functionalization via cross-coupling. Moreover, the synthetic utility of the current protocol was successfully illustrated by applying it to the derivatization of biorelevant structures (12f−h) and a pharmacophore (Indomethacin derivative, 12i). More importantly, electron rich alkenes that have been previously unexplored in photocatalytic hydrodifluoromethylation such as *N*-vinylcarbazole (11j), enamide (11k), *N*-vinyllactams (11l−m) and vinyl ether (11n) underwent smooth conversion to the corresponding products in good to moderate yields under identical reaction conditions. Finally, radical clock substrate 13 afforded cyclized product 14 under standard reaction conditions. This result again highlights the radical intermediacy of the reaction.

As a logical extension, we envisioned the development of an innovative platform for the regioselective consecutive hydrodifluoromethylation of alkynes based on the fact that the hydrocarbons bearing two difluoromethyl groups remain rare but should possess unique properties for drug discovery. Indeed, the ability to introduce multiple difluoromethyl groups into unsaturated C−C bonds presents a difficult proposition even with modern organic chemistry.

**Fig. 7:**
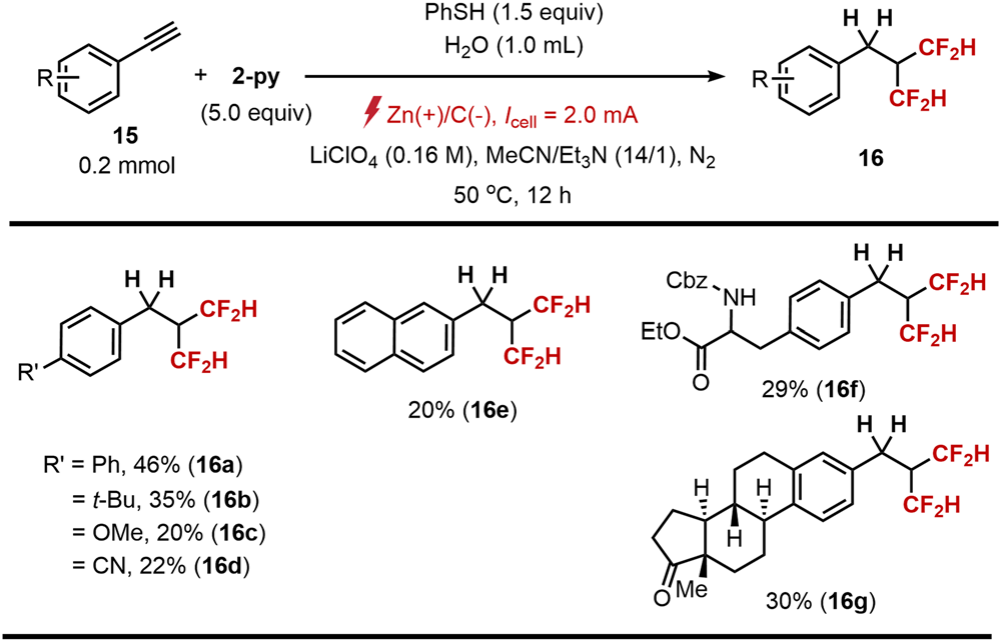
Double hydrodifluoromethylation of alkynes^*a*^. ^*a*^15 (0.2 mmol), 2-py (0.6 mmol), LiClO_4_ (0.8 mmol) and H_2_O (1.0 mL) in MeCN/Et_3_N (14/1, 4.0 mL) at 50 °C. Isolated yields are reported unless otherwise noted.

As envisioned, we found that a multiple hydrodifluoromethylation of arylalkyne 15 can be achieved with increased amount of 2-py at 50 °C and LiClO_4_ as the electrolyte under otherwise identical conditions to the aliphatic alkene hydrodifluoromethylation reaction, successfully generating a geminal bis-difluoromethylation products in good to moderate yields as single regioisomers (Fig. 7, 16a−g). We assumed that the first hydrodifluoromethylation proceeds via HAT of difluoromethylated vinyl radical intermediate, while the second reaction proceeds presumably via a radical-polar crossover mechanism driven by aryl substituents of the employed alkynes (Supplementary Fig. 7).

**Fig. 8:**
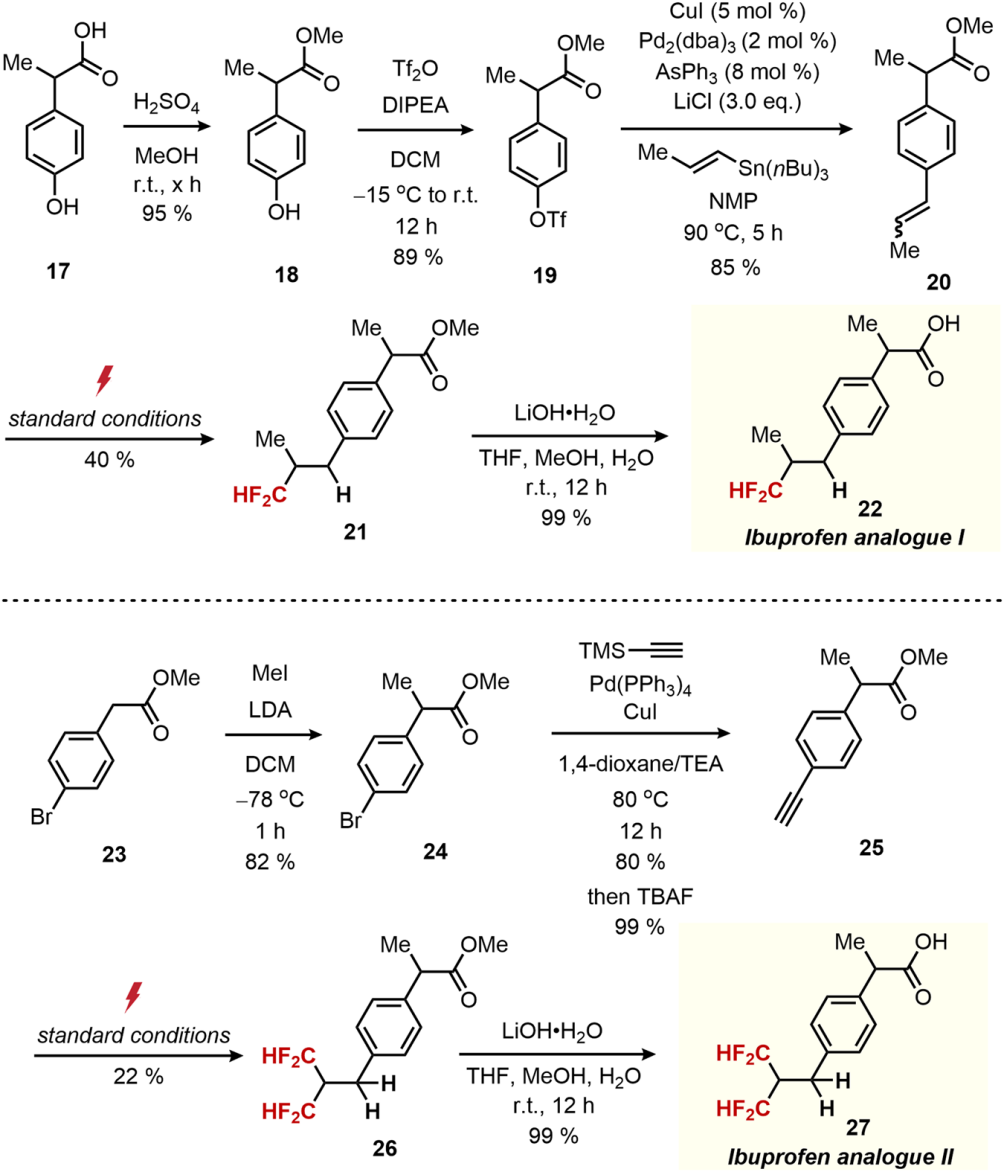
Synthesis of CF_2_H analogues of ibuprofen. The requisite starting material 20 was prepared in three steps from 2-(4-hydroxyphenyl)propanoic acid (17). The desired hydrodifluoromethylation was proceeded under the standard conditions (21). Hydrolysis of 21 led to the formation of difluoromethyl analogue of Ibuprofen (22) in 28% overall yield (5 steps). The developed double hydrodifluoromethylation protocol allowed conversion of alkyne 25 into corresponding geminal bis-difluoromethylation product 26. Upon treatment of 26 with base under aqueous conditions, a bis-difluoromethyl analogue of Ibuprofen (27) was obtained.

Finally, we showcased our methodology in late-stage drug modification of Ibuprofen, a popular analgesic and antipyretic in which its ameliorative derivatization has attracted constant attention in the pharmaceutical chemistry (Fig. 8)^87,88^. The requisite starting material 20 was prepared with good efficiency in three steps from commercially available 2-(4-hydroxyphenyl)propanoic acid (17). As anticipated, the desired hydrodifluoromethylation was smoothly proceeded under the standard conditions (21). Hydrolysis of 21 was facile under basic conditions, leading to the formation of difluoromethyl analogue of Ibuprofen (22) in 28% overall yield (5 steps).

Notably, the developed double hydrodifluoromethylation protocol allowed conversion of alkyne 25 into corresponding geminal bis-difluoromethylation product 26. Upon treatment of 26 with base under aqueous conditions, a bis-difluoromethyl analogue of Ibuprofen (27) could readily be obtained, demonstrating an operationally simple two-track derivatization of a pharmaceutical agent from commercially available starting materials.

## Conclusion

In conclusion, we developed a general electroreductive protocol to achieve hydrodifluoromethylation of a wide range of unsaturated C−C bonds by means of a two-pronged strategy based upon electronic properties of the employed substrates. A key distinction of the present strategy originates from the reconciliation of multiple redox processes under highly reducing electrochemical conditions. We anticipate that this mechanistically distinct and modular protocol will enhance accessibility of a diverse suite of difluoromethylated hydrocarbons which possess high potential utility in pharmaceutical applications.

## Methods

### General procedure for electroreductive hydrodifluoromethylation of vinylarenes

An oven-dried, 10 mL two-neck glass tube was equipped with a magnetic stir bar, a rubber septum, a threaded Teflon cap fitted with electrical feed-throughs, a carbon felt anode (1.0 × 0.5 cm^2^) (connected to the electrical feedthrough via a 9 cm in length, 2 mm in diameter graphite rod), and a zinc plate anode (1 × 0.5 × 0.02 cm^3^). To this reaction vessel, LiClO_4_ (85.1 mg, 0.8 mmol) was added. The cell was sealed and backfilled with nitrogen gas for 3 times, followed by the sequential addition via syringe of MeCN (6.0 mL), water (2.0 mmol, 36.0 μL), **2** (0.4 mmol, 57.0 μL), piperidine (1.0 mmol, 99 μL) and olefin substrate (0.2 mmol, 1.0 equiv). A nitrogen-filled balloon was adapted through the septum to sustain a nitrogen atmosphere. Electrolysis was initiated at a constant current of 4.0 mA at 50 °C for 12 h. The mixture was then diluted with ethyl acetate (30 mL) and then washed with water, brine, dried over anhydrous Na_2_SO_4_, and concentrated under reduced pressure. The residue was subjected to flash column chromatography on silica gel (eluted with hexanes/ethyl acetate) to yield the desired product.

### General procedure for electroreductive hydrodifluoromethylation of electron-deficient alkenes

An oven-dried, 10 mL two-neck glass tube was equipped with a magnetic stir bar, a rubber septum, a threaded Teflon cap fitted with electrical feed-throughs, a carbon felt anode (1.0 × 0.5 cm^2^) (connected to the electrical feedthrough via a 9 cm in length, 2 mm in diameter graphite rod), and a zinc plate anode (1 × 0.5 × 0.02 cm^3^). To this reaction vessel, LiClO_4_ (85.1 mg, 0.8 mmol) and 2-py (0.6 mmol, 115.9 mg) were added. The cell was sealed and backfilled with nitrogen gas for 3 times, followed by the sequential addition via syringe of MeCN (4.0 mL), water (1.0 mL) triethylamine (2.0 mmol, 280 μL) and olefin substrate (0.2 mmol, 1.0equiv). A nitrogen-filled balloon was adapted through the septum to sustain a nitrogen atmosphere. Electrolysis was initiated at a constant current of 2.0 mA at 50 °C for 12 h. The mixture was then diluted with ethyl acetate (30 mL) and then washed with water, brine, dried over anhydrous Na_2_SO_4_, and concentrated under reduced pressure. The residue was subjected to flash column chromatography on silica gel (eluted with hexanes/ethyl acetate) to yield the desired product.

### General procedure for electroreductive hydrodifluoromethylation of unactivated alkenes

An oven-dried, 10 mL two-neck glass tube was equipped with a magnetic stir bar, a rubber septum, a threaded Teflon cap fitted with electrical feed-throughs, a carbon felt anode (1.0 × 0.5 cm^2^) (connected to the electrical feedthrough via a 9 cm in length, 2 mm in diameter graphite rod), and a zinc plate anode (1 × 0.5 × 0.02 cm^3^). To this reaction vessel, TBA·PF_6_ (309.9 mg, 0.8 mmol) and 2-py (0.6 mmol, 115.9 mg) were added. The cell was sealed and backfilled with nitrogen gas for 3 times, followed by the sequential addition via syringe of MeCN (4.0 mL), water (1.0 mL), thiophenol (0.3 mmol. 31 μL) triethylamine (2.0 mmol, 280 μL) and olefin substrate (0.2 mmol, 1.0equiv). A nitrogen-filled balloon was adapted through the septum to sustain a nitrogen atmosphere. Electrolysis was initiated at a constant current of 2.0 mA at 22 °C for 12 h. The mixture was then diluted with ethyl acetate (30 mL) and then washed with water, brine, dried over anhydrous Na_2_SO_4_, and concentrated under reduced pressure. The residue was subjected to flash column chromatography on silica gel (eluted with hexanes/ethyl acetate) to yield the desired product.

### General procedure for electroreductive double hydrodifluoromethylation of alkynes

An oven-dried, 10 mL two-neck glass tube was equipped with a magnetic stir bar, a rubber septum, a threaded Teflon cap fitted with electrical feed-throughs, a carbon felt anode (1.0 × 0.5 cm^2^) (connected to the electrical feedthrough via a 9 cm in length, 2 mm in diameter graphite rod), and a zinc plate anode (1 × 0.5 × 0.02 cm^3^). To this reaction vessel, LiClO_4_ (85.0 mg, 0.8 mmol) and 2-py (1.0 mmol, 193.2 mg) were added. The cell was sealed and backfilled with nitrogen gas for 3 times, followed by the sequential addition via syringe of MeCN (4.0 mL), water (1.0 mL), thiophenol (0.3 mmol. 31 μL) triethylamine (2.0 mmol, 280 μL) and alkyne substrate (0.2 mmol, 1.0equiv). A nitrogen-filled balloon was adapted through the septum to sustain a nitrogen atmosphere. Electrolysis was initiated at a constant current of 2.0 mA at 50 °C for 18 h. The mixture was then diluted with ethyl acetate (30 mL) and then washed with water, brine, dried over anhydrous Na_2_SO_4_, and concentrated under reduced pressure. The residue was subjected to flash column chromatography on silica gel (eluted with hexanes/ethyl acetate) to yield the desired product.

## Supplementary information

Supplementary Information

Supplementary data 1

Description of Additional Supplementary Files

## Data Availability

The authors declare that the data supporting the findings of this study are available within the article and Supplementary Information. For experimental details and compound characterization data see Supplementary Notes 1–4. For ^1^H NMR, ^11^B NMR, ^13^C NMR, and ^19^F NMR spectra see Supplementary Note 5, pages. 31–123.
