# Supplementary material for: Radical hydrodifluoromethylation of unsaturated C−C bonds via an electroreductively triggered two-pronged approach
Source: Commun Chem. 2022 Aug 11;5:96. doi: 10.1038/s42004-022-00697-1 (PMC9814520; doi:10.1038/s42004-022-00697-1)
Supplement: Supplementary file 1 — Supplementary Information [file 42004_2022_697_MOESM1_ESM.pdf]

Supplementary Information

Radical Hydrodifluoromethylation of Unsaturated C–C Bonds via an Electroreductively Triggered Two-pronged Approach

Seonyoung Kim,[1,+] Keon Ha Hwang,[2,3,+] Hyeong Gyu Park,[2,3] Jaesung Kwak,[2*] Hyuk Lee, [2*] and Hyunwoo Kim[1*]

[1]Department of Chemistry and Nanoscience, Ewha Womans University, Seoul 03760, Republic of Korea
[2]Infectious Diseases Therapeutic Research Center, Korea Research Institute of Chemical Technology (KRICT), Daejeon 34114, Republic of Korea
[3]Graduate School of New Drug Discovery and Development, Chungnam University, Daejeon 34134, Republic of Korea

Table of Contents

Supplementary Note 1. General Information 2

Supplementary Note 2. General Procedures for Electroreductive Hydrodifluoromethylation 3

Supplementary Note 3. Optimization of the Reaction Parameters 5

Supplementary Note 4. Procedures of Mechanistic Studies 6

Supplementary Note 5. Spectral Data for Products 12

Supplementary References. 141

Supplementary Note 1

All reactions were performed in oven-dried two-neck glass tubes unless otherwise noted. The tubes were fitted with a rubber septum and a threaded Teflon cap with airtight, electrical feed-throughs. The reactions were conducted under a nitrogen atmosphere. Flash chromatography was performed using silica gel 60 (230-400 mesh) from SiliCycle. Commercial reagents were purchased from Sigma Aldrich, Alfa Aesar, Acros, and TCI and used as received. The starting materials for **8a**, **12a**, **12c**, **12d**, **12e**, **12f**, **12g**, **12h**, **12i, 14, 21,** and **24** respectively) were synthesized by the previously reported procedures ([1-(2-Phenylcyclopropyl)vinyl]benzene[1], hex-5-en-1-yl benzoate[2], (3-methylbut-3-en-1-yl)benzene[3], 3-methylbut-3-en-1-yl 4-(*tert*-butyl)benzoate[4], 2-vinyl-2,3-dihydro-1H-naphtho[1,8-de][1,3,2]diazaborinine[5], (3*aR*,6*S*,6*aR*)-5-(2,2-dimethyl-1,3-dioxolan-4-yl)-6-(hex-5-en-1-yloxy)-2,2-dimethyltetrahydrofuro[2,3-d][1,3]dioxole[6], benzyl (*S*)-[1-(allylamino)-1-oxo-3-phenylpropan-2-yl]carbamate[7] (8*R*,9*S*,13*S*,14*S*)-13-methyl-3-(pent-4-en-1-yloxy)-6,7,8,9,11,12,13,14,15,16-decahydro-17H-cyclopenta[α]phenanthren-17-one[8], hex-5-en-1-yl 2-[1-(4-chlorobenzoyl)-5-methoxy-2-methyl-1H-indol-3-yl]acetate[9], *N*,*N*-diallyl-4-methylbenzenesulfonamide[10], methyl (*E/Z*)-2-(4-(prop-1-en-1-yl)phenyl)propanoate[11], methyl 2-(4-bromophenyl)propanoate[12]). Proton nuclear magnetic resonance (^{1}H NMR) spectra was recorded on 300 MHz, 400 MHz, carbon nuclear magnetic resonance (^{13}C NMR) spectra was recorded on 75 MHz, 100 MHz, or 125 MHz and fluorine nuclear magnetic resonance (^{19}F NMR) was recorded on 282 MHz or 471 MHz by Varian Gemini 500 or Bruker 300 & 400. Chemical shifts for protons are reported in parts per million downfield from tetramethylsilane and are referenced to residual protium in the NMR solvent ($CHCl_3$ = δ 7.26). Chemical shifts for carbon are reported in parts per million downfield from tetramethylsilane and are referenced to the carbon resonances of the solvent ($CDCl_3$ = δ 77.0). Data are represented as follows: chemical shift, multiplicity (br. s = broad, s = singlet, d = doublet, t = triplet, q = quartet, p = pentet, m = multiplet), coupling constants in Hertz (Hz), integration. Infrared (IR) spectra of the newly synthesized compounds were obtained using a Bruker Alpha FT-IR spectrometer. The voltaic profiles were recorded with a Biologics SP-50 potentiostat. High-resolution mass spectra (HRMS) were recorded on a JEOL JMS-700 high resolution mass spectrometer(EI) or Varian 1200L quadrupole MS (EI) spectrophotometer.

Electrolysis experiments were performed using a Biologics SP-50 potentiostat/galvanostat or a DC power supply. Carbon Felt was purchased from Fuel Cell Store. The carbon was cut into 1 x 0.5 x 0.6 cm^3 pieces before use, and was connected to electrical feed-through on the Teflon cap of the electrochemical cell via a piece of graphite (2B pencil lead, 2 mm in diameter). The zinc plate was cut into 1 x 0.5 x 0.02 cm^3 and was connected to electrical feed-through on the Teflon cap of the electrochemical cell via a piece of graphite (2B pencil lead, 2 mm in diameter). Saturated calomel electrode (SCE) reference electrodes were obtained from CH Instruments.

Abbreviations: ^{t}Bu—*tert*-butyl, Me—methyl, Ac—acetyl, Ph—phenyl, Bz—benzoyl, DCM—dichloromethane, MeCN—acetonitrile, TBA—tetrabutylammonium.

Supplementary Note 2

General Procedure for Electroreductive Hydrodifluoromethylation of Unsaturated C−C bonds

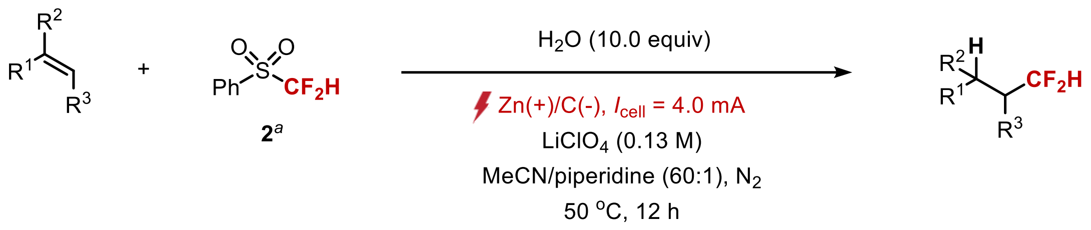

Method A: An oven-dried, 10 mL two-neck glass tube was equipped with a magnetic stir bar, a rubber septum, a threaded Teflon cap fitted with electrical feed-throughs, a carbon felt anode (1.0 *0.5 cm^2) (connected to the electrical feedthrough via a 9 cm in length, 2 mm in diameter graphite rod), and a zinc plate anode (1 x 0.5 x 0.02 cm^3). To this reaction vessel, $LiClO_4$ (85.1 mg, 0.8 mmol) was added. The cell was sealed and backfilled with nitrogen gas for 3 times, followed by the sequential addition via syringe of MeCN (6.0 mL), water (2.0 mmol, 36.0 μL), **2** (0.4 mmol, 57.0 μL), piperidine (1.0 mmol, 99 μL) and olefin substrate (0.2 mmol, 1.0 equiv). A nitrogen-filled balloon was adapted through the septum to sustain a nitrogen atmosphere. Electrolysis was initiated at a constant current of 4.0 mA at 50 °C for 12 h. The mixture was then diluted with ethyl acetate (30 mL) and then washed with water, brine, dried over anhydrous Na_2SO_4, and concentrated under reduced pressure. The residue was subjected to flash column chromatography on silica gel (eluted with hexanes/ethyl acetate) to yield the desired product.

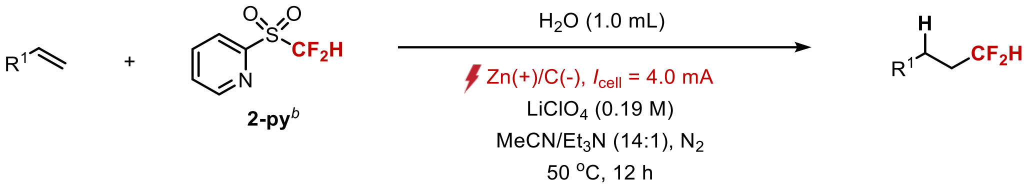

Method B: An oven-dried, 10 mL two-neck glass tube was equipped with a magnetic stir bar, a rubber septum, a threaded Teflon cap fitted with electrical feed-throughs, a carbon felt anode (1.0 *0.5 cm^2) (connected to the electrical feedthrough via a 9 cm in length, 2 mm in diameter graphite rod), and a zinc plate anode (1 x 0.5 x 0.02 cm^3). To this reaction vessel, $LiClO_4$ (85.1 mg, 0.8 mmol) and **2-py** (0.6 mmol, 115.9 mg) were added. The cell was sealed and backfilled with nitrogen gas for 3 times, followed by the sequential addition via syringe of MeCN (4.0 mL), water (1.0 mL) triethylamine (2.0 mmol, 280 μL) and olefin substrate (0.2 mmol, 1.0 equiv). A nitrogen-filled balloon was adapted through the septum to sustain a nitrogen atmosphere. Electrolysis was initiated at a constant current of 2.0 mA at 50 °C for 12 h. The mixture was then diluted with ethyl acetate (30 mL) and then washed with water, brine, dried over anhydrous Na_2SO_4, and concentrated under reduced pressure. The residue was subjected to flash column chromatography on silica gel (eluted with hexanes/ethyl acetate) to yield the desired product.

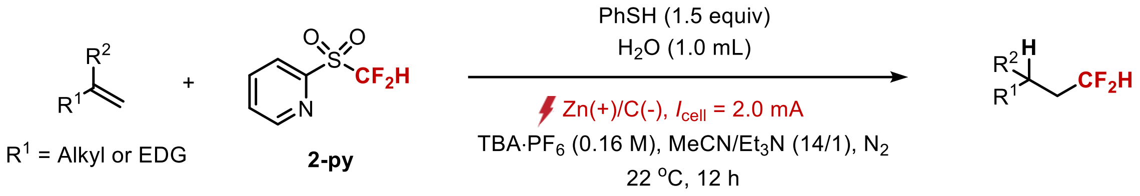

Method C: An oven-dried, 10 mL two-neck glass tube was equipped with a magnetic stir bar, a rubber septum, a threaded Teflon cap fitted with electrical feed-throughs, a carbon felt anode (1.0 *0.5 cm^2) (connected to the electrical feedthrough via a 9 cm in length, 2 mm in diameter graphite rod), and a zinc plate anode (1 x 0.5 x 0.02 cm^3). To this reaction vessel, TBA· PF_6 (309.9 mg, 0.8 mmol) and **2-py** (0.6 mmol, 115.9 mg) were added. The cell was sealed and backfilled with nitrogen gas for 3 times, followed by the sequential addition via syringe of MeCN (4.0 mL), water (1.0 mL), thiophenol (0.3 mmol. 31 μL) triethylamine (2.0 mmol, 280 μL) and olefin substrate (0.2 mmol, 1.0 equiv). A nitrogen-filled balloon was adapted through the septum to sustain a nitrogen atmosphere. Electrolysis was initiated at a constant current of 2.0 mA at 22 °C for 12 h. The mixture was then diluted with ethyl acetate (30 mL) and then washed with water, brine, dried over anhydrous Na_2SO_4, and concentrated under reduced pressure. The residue was subjected to flash column chromatography on silica gel (eluted with hexanes/ethyl acetate) to yield the desired product.

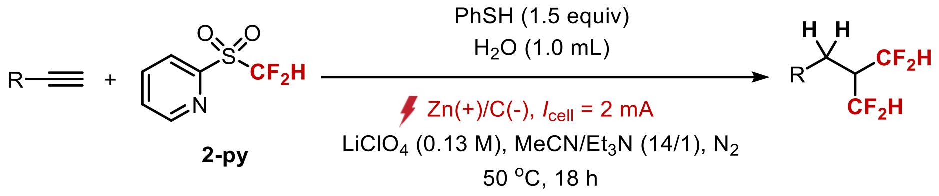

.

Method D: An oven-dried, 10 mL two-neck glass tube was equipped with a magnetic stir bar, a rubber septum, a threaded Teflon cap fitted with electrical feed-throughs, a carbon felt anode (1.0 *0.5 cm^2) (connected to the electrical feedthrough via a 9 cm in length, 2 mm in diameter graphite rod), and a zinc plate anode (1 x 0.5 x 0.02 cm^3). To this reaction vessel, $LiClO_4$ (85.0 mg, 0.8 mmol) and **2-py** (1.0 mmol, 193.2 mg) were added. The cell was sealed and backfilled with nitrogen gas for 3 times, followed by the sequential addition via syringe of MeCN (4.0 mL), water (1.0 mL), thiophenol (0.3 mmol. 31 μL) triethylamine (2.0 mmol, 280 μL) and alkyne substrate (0.2 mmol, 1.0 equiv). A nitrogen-filled balloon was adapted through the septum to sustain a nitrogen atmosphere. Electrolysis was initiated at a constant current of 2.0 mA at 50 °C for 18 h. The mixture was then diluted with ethyl acetate (30 mL) and then washed with water, brine, dried over anhydrous Na_2SO_4, and concentrated under reduced pressure. The residue was subjected to flash column chromatography on silica gel (eluted with hexanes/ethyl acetate) to yield the desired product.

Unsuccessful Scopes

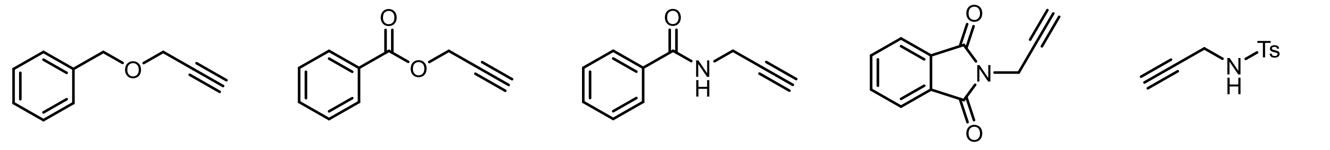

conversion <20%
s.m. remaining 80%
no reactivity observed

conversion >70%
s.m. remaining 25%
no reactivity observed

conversion >70%
s.m. remaining 27%
no reactivity observed

conversion >99%
s.m. remaining 0%
no reactivity observed

conversion >70%
s.m. remaining 25%
no reactivity observed

Supplementary Note 3

Optimization of Reaction Parameters

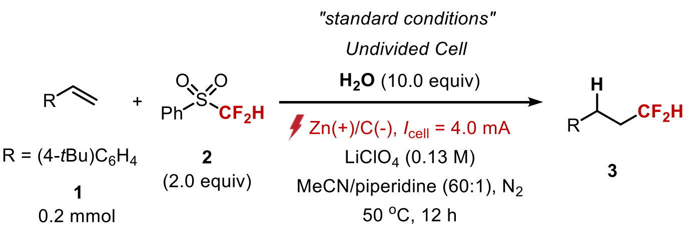

Entry	Variation from "standard conditions"	Yield of **3** (%)
1	none	87 (80)
2	w/o applied voltage	n.r.
3	w/o H_2O	2
4	w/o piperidine	6
5	TEA instead of piperidine	36 (29)
8	**A1** instead of piperidine	36
9	**A2** instead of piperidine	n.r.
10	**A3** instead of piperidine	32
11	**A4** instead of piperidine	30
12	**A5** instead of piperidine	38
13	**A6** instead of piperidine	58
14	**A7** instead of piperidine	46
15	**A8** instead of piperidine	n.r.
16	**A9** instead of piperidine	58
17	2-pySO_2CF_2H (**2-py**) instead of **2**	<5%
18	**2-1** instead of **2**	<5%
19	**2-2** instead of **2**	n.r.
20	**2-3** instead of **2**	n.r.
21	**2-4** instead of **2**	<5%
21	**2-5** instead of **2**	n.r.
22	under constant voltage ($E_{cathode}$ = –2.2 V)	<5%
23	Mg as a sacrificial anode	7
24	Al as a sacrificial anode	9

Amines Tested

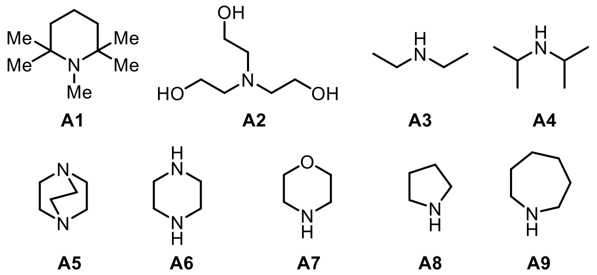

CF_2H Radical Sources Tested

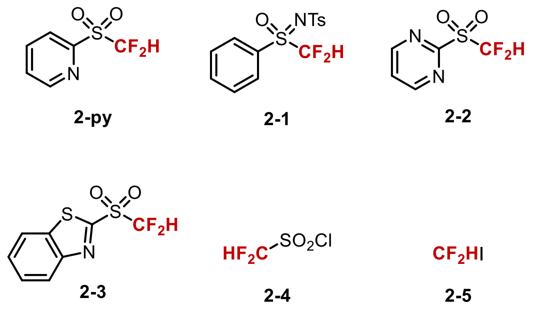

Supplementary Table 1. Optimization Study

Supplementary Note 4

Procedures of Mechanistic Studies

1. Deuterium Labeling Experiments

1.1. Reaction under D_2O as an additive

An identical procedure was followed as described in **Section 2** with the exception that the reaction was carried out in the presence of D_2O (10 equiv) instead of H_2O. When the reaction was finished, the mixture was then diluted with ethyl acetate (30 mL) and then washed with water, brine, dried over anhydrous Na_2SO_4, and concentrated under reduced pressure. The residue was subjected to flash column chromatography on silica gel (eluted with hexanes/ethyl acetate) to yield the desired product. The ratio of proton/deuterium (H/D) of desired product was determined by integration using a set of aromatic H peaks (7.14 ppm, 2H) as an internal standard.

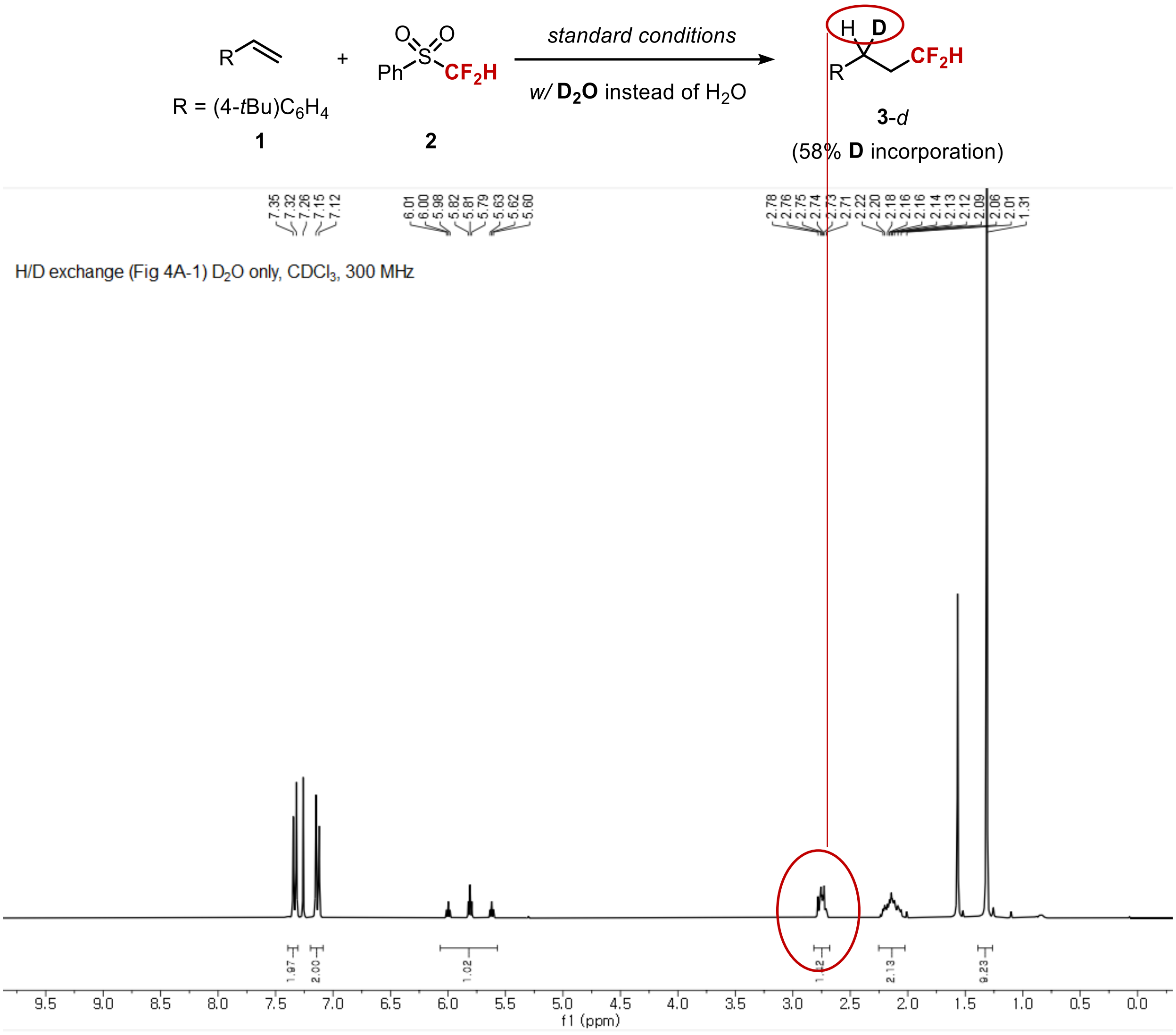

Supplementary Figure 1. ^{1}H NMR of Deuterium Scrambling Experiment Using D_2O

1.2. Reaction under piperidine-d_{11} as a base additive

An identical procedure was followed as described in **Section 2** with the exception that the reaction was carried out in the presence of piperidine-d_{11} instead of piperidine. When the reaction was finished, the mixture was then diluted with ethyl acetate (30 mL) and then washed with water, brine, dried over anhydrous Na_2SO_4, and concentrated under reduced pressure. The residue was subjected to flash column chromatography on silica gel (eluted with hexanes/ethyl acetate) to yield the desired product. The ratio of proton/deuterium (H/D) of desired product was determined by integration using a set of aromatic H peaks (7.14 ppm, 2H) as an internal standard.

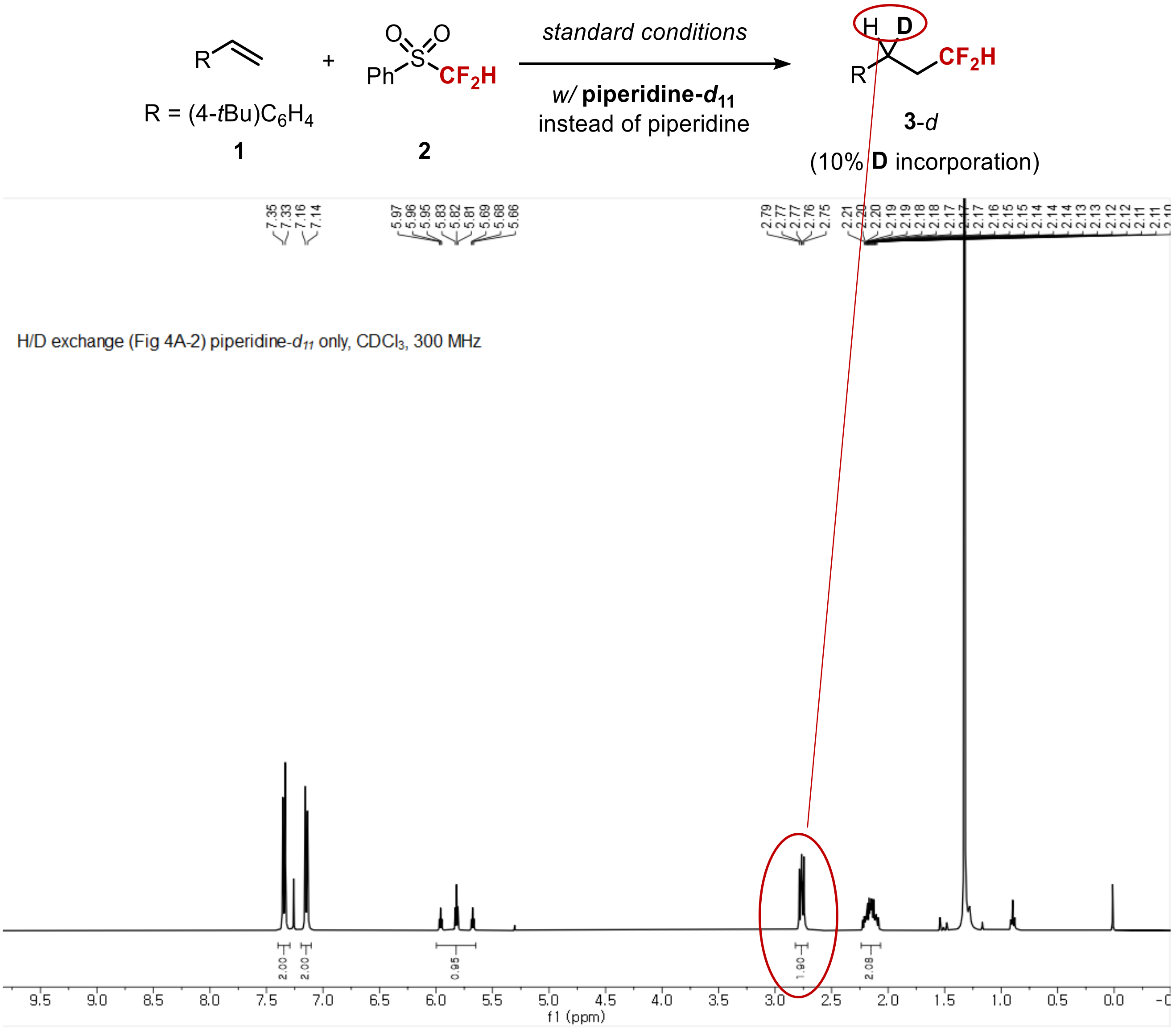

Supplementary Figure 2. ^{1}H NMR of Deuterium Scrambling Experiment Using Piperidine-d_{11}

2. Radical probe experiments

An identical procedure was followed as described in **Section 2, Method A** with the radical probe substrates **6a** and **6b**.

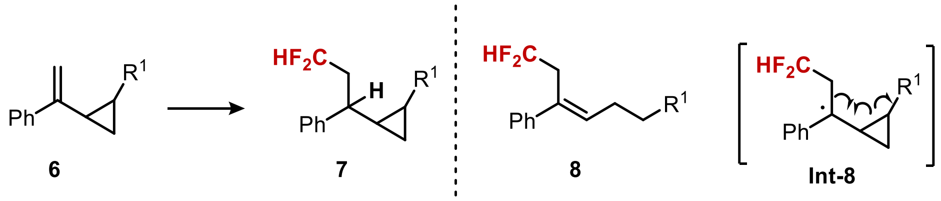

Substrates (**6**)	Products (**7**/**8**)		Rate constant (k_{R1})
R^1 = Ph (**6a**)	not observed (**7a**)	36% (**8a**)	k_{Ph} ~ 10^8 s^{-1}
R^1 = H (**6b**)	99% (**7b**)	not observed (**8b**)	k_H ~ 10^5 s^{-1}

Supplementary Figure 3. Radical Probe Experiments

3. Voltammetric studies

General Procedure for Voltaic Profile

An identical procedure was followed as described in **Section 2** with a Ag/Ag+ quasi-reference electrode with 0.1 M TBA·ClO4 and 0.01 M AgNO3 in MeCN. The reference electrode was calibrated against an internal standard of ferrocene (Fc) following electrolysis so that voltaic profiles could be referenced against Fc/Fc+.

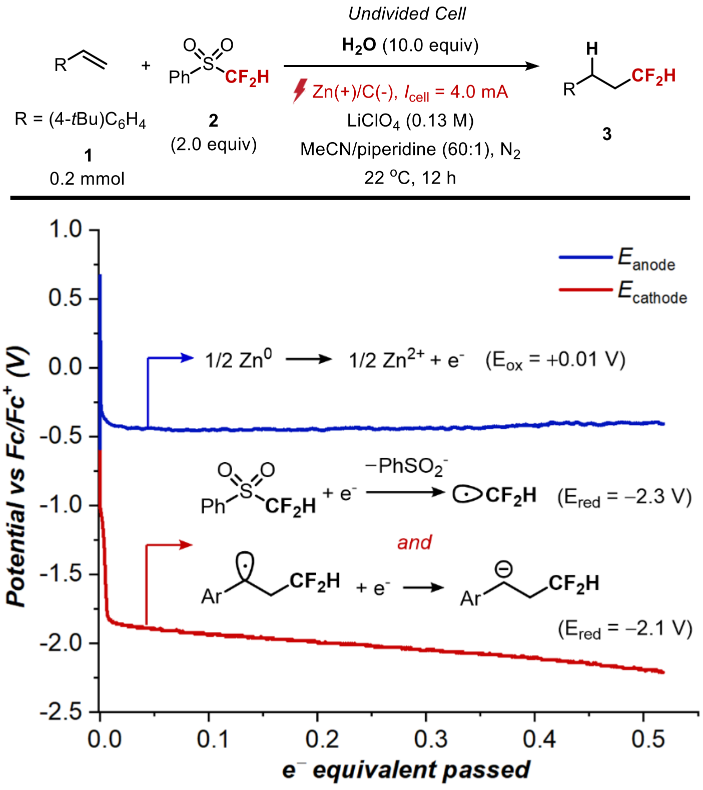

Supplementary Figure 4. Voltaic Profile Under the Optimized Reaction Conditions

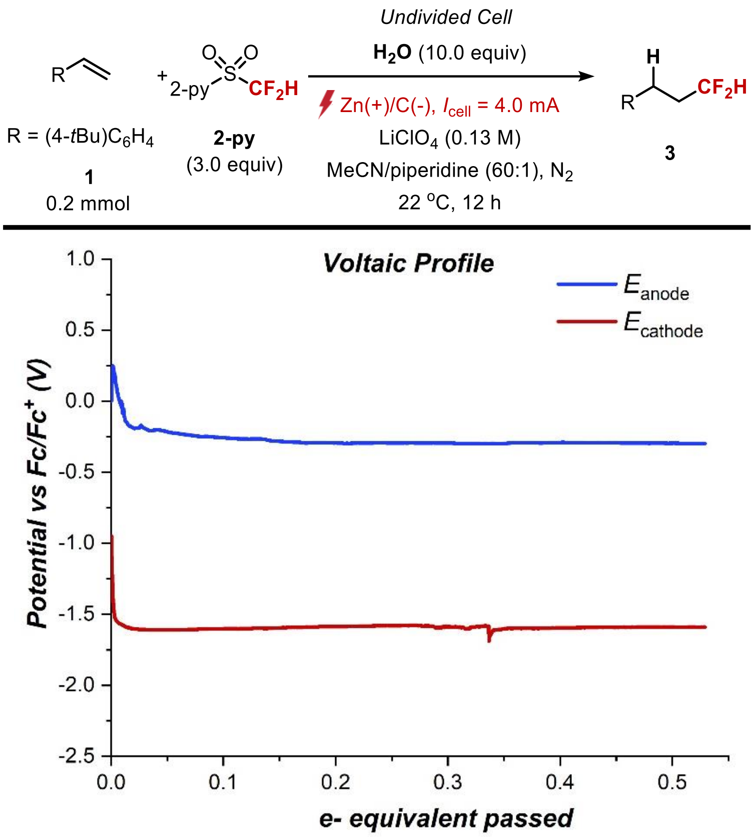

Supplementary Figure 5. Voltaic Profile in the Presence of **2-py** as a CF_2H Radical Precuror

General Procedure for Current Profile

An oven-dried, 10 mL two-neck glass tube was equipped with a magnetic stir bar, a rubber septum, a threaded Teflon cap fitted with electrical feed-throughs, a carbon felt anode (1.0 *0.5 cm^2) (connected to the electrical feedthrough via a 9 cm in length, 2 mm in diameter graphite rod), a zinc plate anode (1 x 0.5 x 0.02 cm^3), and . a Ag/Ag+ quasi-reference electrode with 0.1 M TBA·ClO_4 and 0.01 M $AgNO_3$ in MeCN. The reference electrode was calibrated against an internal standard of ferrocene (Fc) following electrolysis so that voltaic profiles could be referenced against Fc/Fc+.

To this reaction vessel, $LiClO_4$ (85.1 mg, 0.8 mmol) was added. The cell was sealed and backfilled with nitrogen gas for 3 times, followed by the sequential addition via syringe of MeCN (4.0 mL), water (2.0 mmol, 36.0 μL), **2** (0.4 mmol, 57.0 μL), piperidine (1.0 mmol, 99 μL) and olefin substrate (0.2 mmol, 1.0 equiv). A nitrogen-filled balloon was adapted through the septum to sustain a nitrogen atmosphere. Electrolysis was performed at a constant cathodic potential of –2.3 V vs Fc/Fc+ at 22 °C for 12 h.

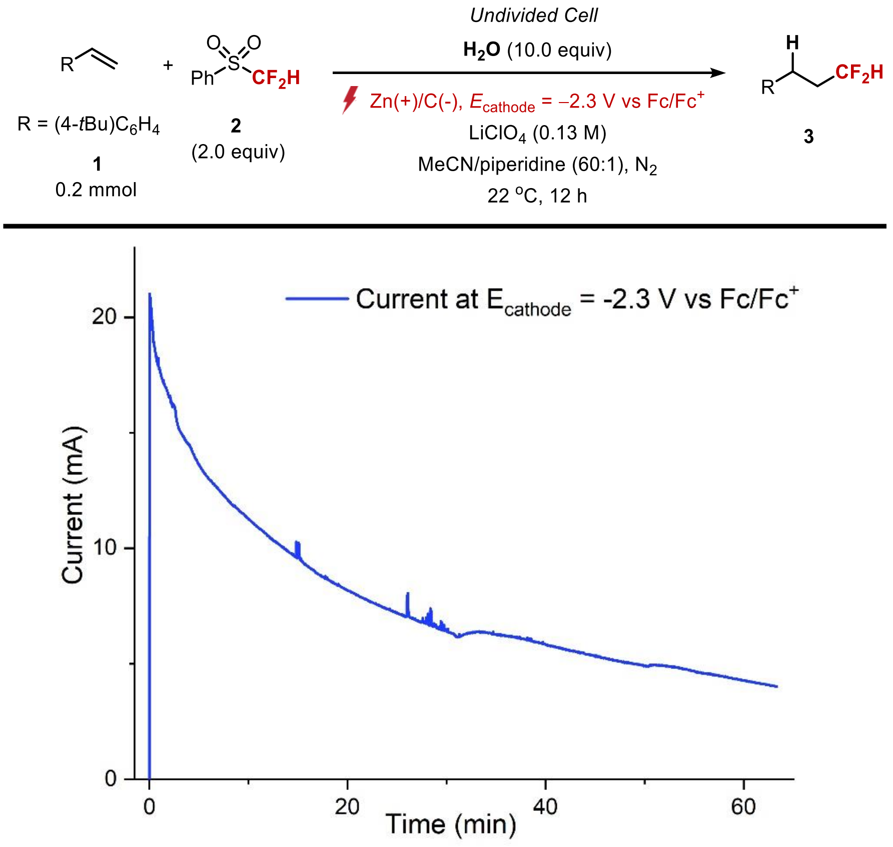

Supplementary Figure 6. Current Profile under Constant Cathodic Voltage of –2.3 V vs Fc/Fc^+

4. Mechanistic Postulate for Double Hydrodifluoromethylation of Alkynes

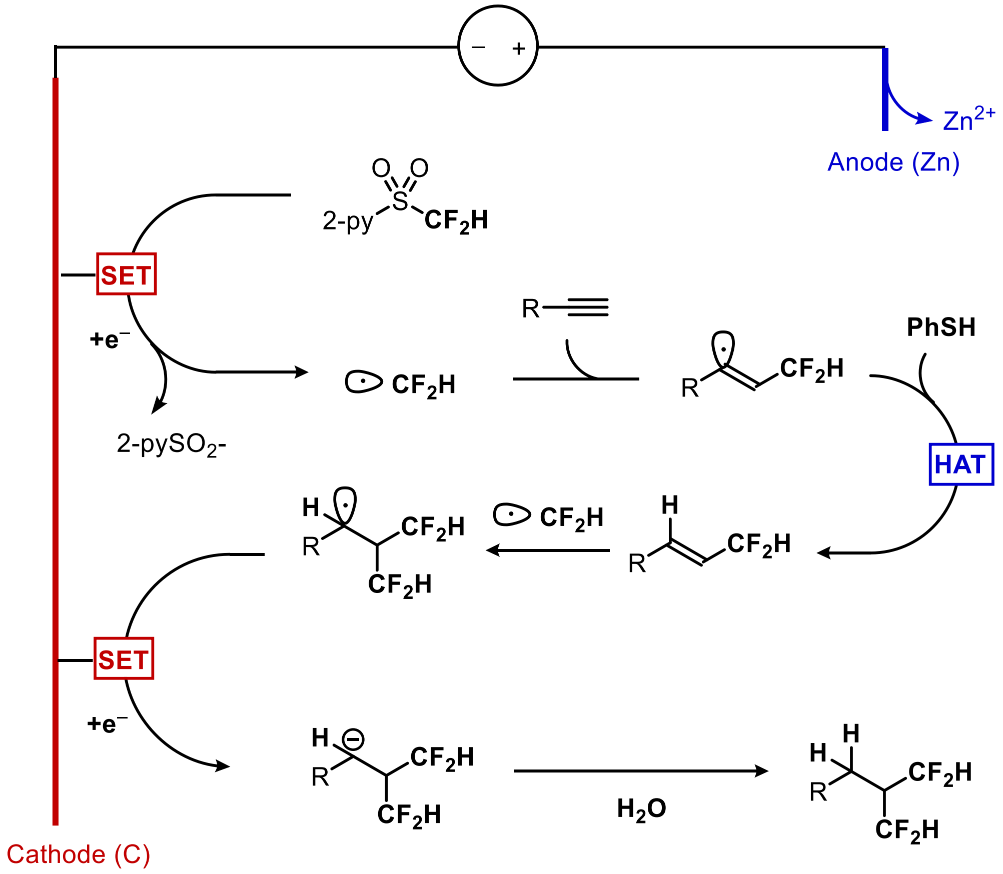

Supplementary Figure 7. Mechanistic Rationale for Double Hydrodifluoromethylation

Supplementary Note 5

Spectral Data for Products

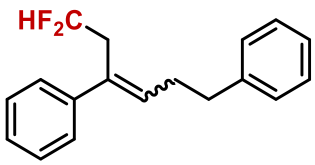

(6,6-Difluorohex-3-ene-1,4-diyl)dibenzene (8a). Method A. Purified using silica gel chromatography to give 36% yield of **8a** as a colorless oil; ^{1}H NMR (300 MHz, $CDCl_3$) δ 7.36 – 7.27 (m, 7H), 7.25 – 7.20 (m, 3H), 5.95 (t, *J* = 7.4 Hz, 1H), 5.63 (tt, *J* = 56.8, 4.9 Hz, 1H), 3.00 (td, *J* = 16.5, 4.8 Hz, 2H), 2.80 (t, *J* = 7.6 Hz, 2H), 2.57 (dd, *J* = 15.0, 7.4 Hz, 2H); ^{13}C NMR (75 MHz, $CDCl_3$) δ 141.9, 141.4, 133.0, 131.9 (t, *J* = 6.1 Hz), 128.5, 128.5, 128.4, 127.2, 126.3, 126.0, 116.0 (t, *J* = 241.3 Hz), 35.6, 35.3 (t, *J* = 22.6 Hz), 30.9; ^{19}F NMR (282 MHz, $CDCl_3$) δ -114.2 (dt, *J* = 32.3, 17.0 Hz); IR (Film): 3061, 3027, 2929, 2858, 1601, 1495, 1451, 1393, 1365, 1283, 1215, 1117, 1049, 884, 747, 699, 497 cm^{-1}; HRMS (EI) exact mass calculated for [M^{+}, $C_{18}H_{18}F_2^{+}$]: 272.1377, found 272.1376.

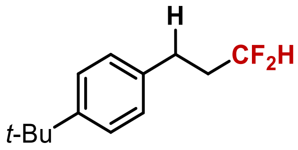

1-(*tert*-Butyl)-4-(3,3-difluoropropyl)benzene (10a). Method A. Purified using silica gel chromatography to give 79% yield of **10a** as a colorless oil; ^{1}H NMR (400 MHz, $CDCl_3$) δ 7.33 (d, *J* = 8.3 Hz, 2H), 7.14 (d, *J* = 8.3 Hz, 2H), 5.81 (tt, *J* = 56.7, 4.5 Hz, 1H), 2.78 – 2.74 (m, 2H), 2.21 – 2.08 (m, 2H), 1.31 (s, 9H); ^{13}C NMR (100 MHz, $CDCl_3$) δ 149.3, 136.8, 128.0, 125.6, 116.8 (t, *J* = 242.4 Hz), 35.7 (t, *J* = 20.2 Hz), 34.4, 31.4, 27.9 (t, *J* = 10.1 Hz); ^{19}F NMR (376 MHz, $CDCl_3$) δ -117.1 (dt, *J* = 56.4, 17.0 Hz); IR (neat): 2964, 2870, 1514, 1402, 1121, 1055, 834, 582 cm^{-1}; HRMS (EI) exact mass calculated for [$C_{13}H_{18}F_2^{+}$]: 212.1377, found 212.1370.

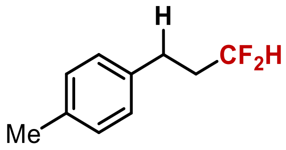

1-(3,3-Difluoropropyl)-4-methylbenzene (10b). Method A. Purified using silica gel chromatography to give 83% yield of **10b** as a colorless oil; ^{1}H NMR (400 MHz, $CDCl_3$) δ 7.13 – 7.08 (m, 4H), 5.79 (tt, *J* = 56.7, 4.5 Hz, 1H), 2.76 – 2.72 (m, 2H), 2.33 (s, 3H), 2.20 – 2.06 (m, 2H); ^{13}C NMR (100 MHz, $CDCl_3$) δ 136.8, 135.9, 129.3, 128.2, 116.8 (t, *J* = 232.3 Hz), 35.8 (t, *J* = 20.2 Hz), 28.0 (t, *J* = 10.1 Hz), 21.0; ^{19}F NMR (376 MHz, $CDCl_3$) δ -117.1 (dt, *J* = 57.0, 17.1 Hz); IR (neat): 2972, 2931, 2866, 1516, 1403, 1120, 1055, 837 cm^{-1}; HRMS (EI) exact mass calculated for [$C_{10}H_{12}F_2^{+}$]: 170.0907, found 170.0916.

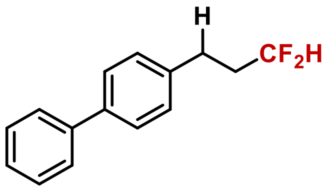

4-(3,3-Difluoropropyl)-1,1'-biphenyl (10c). Method A. Purified using silica gel chromatography to give 51% yield of **10c** as a white solid; m.p. 44 °C; ^{1}H NMR (400 MHz, $CDCl_3$) δ 7.60 – 7.54 (m, 4H), 7.46 – 7.42 (m, 2H), 7.37 – 7.32 (m, 1H), 7.29 – 7.27 (m, 2H), 5.85 (tt, *J* = 56.6, 4.5 Hz, 1H), 2.86 – 2.82 (m, 2H), 2.26 – 2.13 (m, 2H); ^{13}C NMR (100 MHz, $CDCl_3$) δ 140.8, 139.4, 139.0, 128.8, 128.8, 127.4, 127.2, 127.0, 116.7 (t, *J* = 242.4 Hz), 35.7 (t, *J* = 20.2 Hz), 28.0 (t, *J* = 10.1 Hz); ^{19}F NMR (376 MHz, $CDCl_3$) δ -117.1 (dt, *J* = 57.6, 17.2 Hz); IR (neat): 3032, 2970, 2934, 2866, 1952, 1676, 1525, 1487, 1403, 1127, 1049, 945, 763, 690, 506 cm^{-1}; HRMS (EI) exact mass calculated for [$C_{15}H_{14}F_2^+$]: 232.1064, found 232.1057.

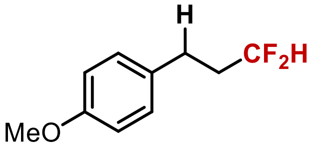

1-(3,3-Difluoropropyl)-4-methoxybenzene (10d). Method A. Purified using silica gel chromatography to give 65% yield of **10d** as a colorless oil; ^{1}H NMR (400 MHz, $CDCl_3$) δ 7.12 (d, *J* = 8.6 Hz, 2H), 6.85 (d, *J* = 8.6 Hz, 2H), 5.79 (tt, *J* = 56.7, 4.5 Hz, 1H), 3.80 (s, 3H), 2.75 – 2.71 (m, 2H), 2.18 – 2.05 (m, 2H); ^{13}C NMR (100 MHz, $CDCl_3$) δ 158.2, 131.9, 129.3, 116.8 (t, *J* = 242.4 Hz), 114.1, 55.3, 35.9 (t, *J* = 20.2 Hz), 27.6 (t, *J* = 10.1 Hz); ^{19}F NMR (376 MHz, $CDCl_3$) δ -117.2 (dt, *J* = 56.6, 17.0 Hz); IR (Film): 2961, 2939, 2839, 1614, 1514, 1460, 1404, 1248, 1180, 1121, 1050, 945, 830, 521 cm^{-1}; HRMS (EI) exact mass calculated for [$C_9H_9F_3^+$]: 186.0856, found 186.0860.

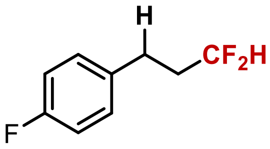

1-(3,3-Difluoropropyl)-4-fluorobenzene (10e). Method A. Purified using silica gel chromatography to give 53% yield of **10e** as a colorless oil; ^{1}H NMR (400 MHz, $CDCl_3$) δ 7.17 – 7.14 (m, 2H), 7.01 – 6.97 (m, 2H), 5.80 (tt, *J* = 56.6, 4.4 Hz, 1H), 2.78 – 2.74 (m, 2H), 2.19 – 2.06 (m, 2H); ^{13}C NMR (100 MHz, $CDCl_3$) δ 161.6 (d, *J* = 244.4 Hz), 135.5 (d, *J* = 3.2 Hz), 129.7 (d, *J* = 7.9 Hz),116.5 (t, *J* = 242.4 Hz), 115.5 (d, *J* = 21.3 Hz), 35.8 (t, *J* = 20.2 Hz), 27.6 (t, *J* = 10.1 Hz); ^{19}F NMR (376 MHz, $CDCl_3$) δ -116.8 – -116.9 (m, 1F), -117.1 (dt, *J* = 56.6, 17.1 Hz, 2F); IR (Film): 3044, 2971, 2937, 2871, 1891, 1604, 1512, 1405, 1225, 1121, 1052, 948, 918, 831, 469 cm^{-1}; HRMS (EI) exact mass calculated for [$C_9H_9F_3^+$]: 174.0656, found 174.0657.

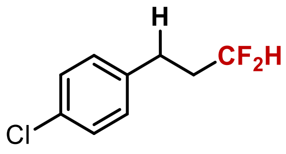

1-Chloro-4-(3,3-difluoropropyl)benzene (10f). Method A. Purified using silica gel chromatography to give 63% yield of **10f** as a colorless oil; ^{1}H NMR (400 MHz, $CDCl_3$) δ 7.28 (d, *J* = 8.4 Hz, 2H), 7.13 (d, *J* = 8.2 Hz, 2H), 5.80 (tt, *J* = 56.5, 4.4 Hz, 1H), 2.78 – 2.74 (m, 2H), 2.19 – 2.08 (m, 2H); ^{13}C NMR (125 MHz, $CDCl_3$) δ 138.3, 132.2, 129.7, 128.8, 116.4 (t, *J* = 239.4 Hz), 35.5 (t, *J* = 25.2 Hz), 27.7 (t, *J* = 12.6 Hz); ^{19}F NMR (376 MHz, $CDCl_3$) δ -117.2 (dt, *J* = 56.4, 17.0 Hz); IR (neat): 2974, 2938, 1493, 1405, 1122, 1094, 1052, 919, 836 cm^{-1}; HRMS (EI) exact mass calculated for [$C_9H_9ClF_2^+$]: 190.0361, found 190.0368.

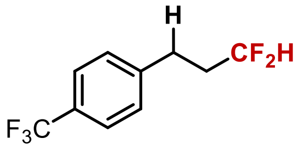

1-(3,3-Difluoropropyl)-4-(trifluoromethyl)benzene (10g). Method A. Purified using silica gel chromatography to give 68% yield of **10g** as a colorless oil; ^{1}H NMR (400 MHz, $CDCl_3$) δ 7.57 (d, *J* = 8.0 Hz, 2H), 7.32 (d, *J* = 8.0 Hz, 2H), 5.83 (tt, *J* = 56.5, 4.3 Hz, 1H), 2.88 – 2.84 (m, 2H), 2.24 – 2.10 (m, 2H); ^{13}C NMR (125 MHz, $CDCl_3$) δ 144.2, 129.0 (q, *J* = 32.4 Hz), 128.8, 125.8 (q, *J* = 3.7 Hz), 124.3 (q, *J* = 271.9 Hz), 116.4 (t, *J* = 239.3 Hz), 35.5 (t, *J* = 21.3 Hz), 28.3 (t, *J* = 5.9 Hz); ^{19}F NMR (376 MHz, $CDCl_3$) δ -62.5 (s, 3F), -117.2 (dt, *J* = 56.5, 17.0 Hz, 2F); IR (neat): 2976, 2873, 1620, 1408, 1328, 1124, 1067, 847 cm^{-1}; HRMS (EI) exact mass calculated for [$C_{10}H_9F_5^+$]: 224.0624, found 224.0621.

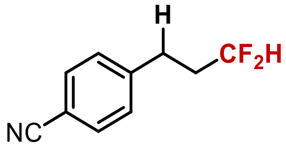

4-(3,3-Difluoropropyl)benzonitrile (10h). Method A. Purified using silica gel chromatography to give 75% yield of **10h** as a colorless oil; ^{1}H NMR (400 MHz, $CDCl_3$) δ 7.61 (d, *J* = 8.3 Hz, 2H), 7.31 (d, *J* = 8.1 Hz, 2H), 5.83 (tt, *J* = 56.4, 4.3Hz, 1H), 2.88 – 2.84 (m, 2H), 2.23 – 2.10 (m, 2H); ^{13}C NMR (100 MHz, $CDCl_3$) δ 145.5, 132.5, 129.2, 118.8, 116.1 (t, *J* = 242.4 Hz), 110.5, 35.1 (t, *J* = 20.2 Hz), 28.4 (t, *J* = 10.1 Hz); ^{19}F NMR (376 MHz, $CDCl_3$) δ -117.2 (dt, *J* = 56.4, 17.0 Hz); IR (neat): 3043, 2975, 2940, 2871, 2229, 1609, 1507, 1443, 1407, 1282, 1180, 1122, 1054, 947, 846, 579 cm^{-1}; HRMS (EI) exact mass calculated for [$C_{10}H_9F_2N^+$]: 181.0703, found 181.0723.

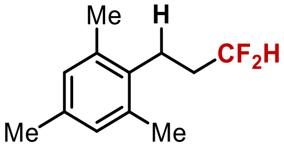

2-(3,3-Difluoropropyl)-1,3,5-trimethylbenzene (10i). Method A. Purified using silica gel chromatography to give 61% yield of **10i** as a colorless oil; ^{1}H NMR (400 MHz, $CDCl_3$) δ 6.85 (s, 2H), 5.87 (tt, *J* = 56.7, 4.2 Hz, 1H), 2.78 – 2.74 (m, 2H), 2.29 (s, 6H), 2.25 (s, 3H), 2.04 – 1.90 (m, 2H); ^{13}C NMR (100 MHz, $CDCl_3$) δ 136.0, 135.7, 133.7, 129.1, 116.9 (t, *J* = 242.4 Hz), 33.5 (t, *J* = 20.2 Hz), 21.5 (t, *J* = 10.1 Hz), 20.8, 19.6; ^{19}F NMR (376 MHz, $CDCl_3$) δ -116.8 (dt, *J* = 56.6, 17.7 Hz); IR (neat): 2968, 2925, 1732, 1614, 1447, 1401, 1329, 1123, 1061, 851 cm^{-1}; HRMS (EI) exact mass calculated for [$C_{12}H_{16}F_2^+$]: 198.1220, found 198.1220.

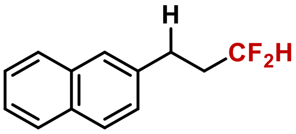

2-(3,3-Difluoropropyl)naphthalene (10j). Method A. Purified using silica gel chromatography to give 61% yield of **10j** as a white solid; m.p. 33 °C; ^{1}H NMR (400 MHz, $CDCl_3$) δ 7.84 – 7.78 (m, 3H), 7.65 (s, 1H), 7.50 – 7.43 (m, 2H), 7.34 (dd, *J* = 8.5, 1.8 Hz, 1H), 5.85 (tt, *J* = 56.7, 4.5 Hz, 1H), 2.98 – 2.94 (m, 2H), 2.32 – 2.18 (m, 2H); ^{13}C NMR (100 MHz, $CDCl_3$) δ 137.4, 133.6, 132.2, 128.4, 127.7, 127.5, 126.9, 126.6, 126.2, 125.6, 116.7 (t, *J* = 242.4 Hz), 35.6 (t, *J* = 20.2 Hz), 28.6 (t, *J* = 10.1 Hz); ^{19}F NMR (376 MHz, $CDCl_3$) δ -117.1 (dt, *J* = 56.6, 17.0 Hz); IR (Film): 3055, 2971, 2860, 1930, 1599, 1508, 1366, 1123, 1055, 953, 825, 748, 479 cm^{-1}; HRMS (EI) exact mass calculated for [$C_{13}H_{12}F_2^+$]: 206.0907, found 206.0915.

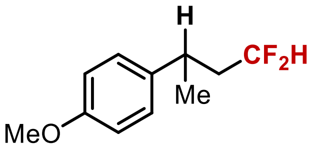

1-(4,4-difluorobutan-2-yl)-4-methoxybenzene (10k). Method A. Purified using silica gel chromatography to give 35% yield of **10k** as a colorless oil; ^{1}H NMR (400 MHz, $CDCl_3$) δ 7.15 (d, *J* = 8.7 Hz, 2H), 6.89 (d, *J* = 8.7 Hz, 2H), 5.78 – 5.42 (m, 1H), 3.82 (s, 3H), 3.01 – 2.91 (m, 1H), 2.16 – 2.04 (m, 2H), 1.32 (d, *J* = 7.0 Hz, 3H); ^{13}C NMR (100 MHz, $CDCl_3$) δ 158.3, 137.1, 127.7, 116.8 (t, *J* = 242.4 Hz), 114.1, 55.3, 42.4 (t, *J* = 20.2 Hz), 33.8 (q, *J* = 10.1 Hz), 22.6; ^{19}F NMR (376 MHz, $CDCl_3$) δ -115.17 (dt, J = 56.5, 13.4 Hz), -115.92 (ddd, J = 56.5, 15.0, 12.0 Hz), -117.07 (ddd, J = 56.9, 21.0, 17.0 Hz), -117.82 (dt, 56.6, 20.2 Hz); IR (neat): 2964, 2933, 2839, 1612, 1513, 1461, 1401, 1298, 1249, 1121, 1035, 831, 554 cm^{-1}; HRMS (EI) exact mass calculated for [$C_{11}H_{14}F_2O^+$]: 200.1013, found 200.1010.

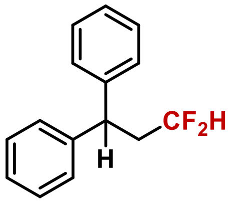

(3,3-Difluoropropane-1,1-diyl)dibenzene (10l). Method A. Purified using silica gel chromatography to give 62% yield of **10l** as a colorless oil; ^{1}H NMR (400 MHz, $CDCl_3$) δ 7.33–7.20 (m, 10H), 5.58 (tt, *J* = 56.7, 5.1 Hz, 1H), 4.18 (t, *J* = 8.1 Hz, 1H), 2.64 – 2.53 (m, 2H); ^{13}C NMR (100 MHz, $CDCl_3$) δ 142.91, 128.8, 127.6, 126.8, 116.6 (t, *J* = 242.4 Hz), 45.3 (t, *J* = 20.2 Hz), 39.8 (t, *J* = 10.1 Hz); ^{19}F NMR (376 MHz, $CDCl_3$) δ -117.0 (dt, *J* = 56.7, 15.8 Hz); IR (Film): 3063, 3029, 2983, 2934, 1951, 1600, 1495, 1451, 1403, 1370, 1120, 1050, 743, 700, 545, 470 cm^{-1}; HRMS (EI) exact mass calculated for [$C_{15}H_{14}F_2^+$]: 232.1064, found 232.1068.

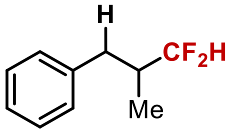

2-(3,3-Difluoropropyl)-1,3,5-trimethylbenzene (10m). Method A. Purified using silica gel chromatography to give 65% yield of **10m** as a colorless oil; ^{1}H NMR (400 MHz, $CDCl_3$) δ 7.35 – 7.19 (m, 5H), 5.67 (td, *J* = 56.8, 3.5 Hz, 1H), 2.92 (dd, *J* = 13.6, 5.7 Hz, 1H), 2.51 (dd, *J* = 13.6, 9.1 Hz, 1H), 2.28 – 2.13 (m, 1H), 1.00 (d, *J* = 6.9 Hz, 3H); ^{13}C NMR (100 MHz, $CDCl_3$) δ 138.8, 129.1, 128.5, 126.4, 118.6 (t, *J* = 242.4 Hz), 39.3 (t, *J* = 20.2 Hz), 36.2 (q, *J* = 10.1 Hz), 11.9 (t, *J* = 10.1 Hz); ^{19}F NMR (376 MHz, $CDCl_3$) δ -123.43 (ddd, J = 276.9, 56.8, 12.6 Hz), -126.42 (ddd, J = 277.1, 56.9, 17.7 Hz).; IR (neat): 3030, 2977, 2944, 1655, 1495, 1459, 1394, 1152, 1055, 991, 746, 701 cm^{-1}; HRMS (EI) exact mass calculated for [$C_{10}H_{12}F_2^+$]: 170.0907, found 170.0914.

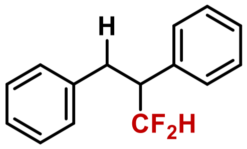

(3,3-difluoropropane-1,2-diyl)dibenzene (10n). Method A. Purified using silica gel chromatography to give 58% yield of **10n** as a colorless oil; ^{1}H NMR (400 MHz, $CDCl_3$) δ 7.32 – 7.27 (m, 3H), 7.24 – 7.13 (m, 5H), 7.06 – 7.03 (m, 2H), 5.91 (td, *J* = 56.5, 3.2 Hz, 1H), 3.35 – 3.22 (m, 2H), 3.06 – 2.89 (m, 1H); ^{13}C NMR (100 MHz, $CDCl_3$) δ 138.4, 136.4 – 136.3 (m), 129.1, 129.1, 128.5, 128.4, 127.6, 126.4, 117.3 (t, *J* = 244.3 Hz), 51.7 (t, *J* = 19.6 Hz), 35.3 (dd, *J* = 5.6, 3.4 Hz); ^{19}F NMR (376 MHz, $CDCl_3$) δ -120.53 (ddd, J = 72.3, 56.5, 16.4 Hz), -122.74 (ddd, J = 277.9, 56.6, 14.7 Hz); IR (neat): 3064, 3031, 2961, 2867, 1951, 1877, 1603, 1496, 1454, 1382, 1129, 1061, 1028, 744, 701, 569 cm^{-1}; HRMS (EI) exact mass calculated for [$C_{15}H_{14}F_2^+$]: 232.1064, found 232.1069.

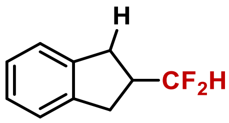

2-(Difluoromethyl)-2,3-dihydro-1H-indene (10o). Method A. Purified using silica gel chromatography to give 67% yield of **10o** as a colorless oil; ^{1}H NMR (400 MHz, $CDCl_3$) δ 7.24 – 7.16 (m, 4H), 5.78 (td, *J* = 56.9, 5.2 Hz, 1H), 3.15 – 3.08 (m, 2H), 3.02 – 2.83 (m, 3H); ^{13}C NMR (100 MHz, $CDCl_3$) δ 141.3, 126.7, 124.6, 118.4 (t, *J* = 242.4 Hz), 42.6 (t, *J* = 20.2 Hz), 32.8 (t, *J* = 10.1 Hz); ^{19}F NMR (376 MHz, $CDCl_3$) δ -119.8 (dd, *J* = 56.6, 13.6 Hz); IR (neat): 3072, 3028, 2954, 2856, 1513, 1065, 746 cm^{-1}; HRMS (EI) exact mass calculated for [$C_{10}H_{10}F_2^+$]: 168.0751, found 168.0754.

Spectroscopic data is in accordance with previous reports.[13]

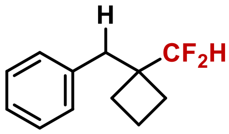

((1-(Difluoromethyl)cyclobutyl)methyl)benzene (10p). Method A. Purified using silica gel chromatography to give 25% yield of **10p** as a colorless oil; ^{1}H NMR (400 MHz, $CDCl_3$) δ 7.33 – 7.20 (m, 5H), 5.55 (t, *J* = 57.2 Hz, 1H), 2.86 (s, 2H), 2.19 – 2.11 (m, 2H), 1.97 – 1.71 (m, 4H); ^{13}C NMR (100 MHz, $CDCl_3$) δ 137.1, 130.1, 128.3, 126.6, 118.5 (t, *J* = 242.4 Hz), 44.7 (t, *J* = 19.7 Hz), 40.3 (t, *J* = 10.1 Hz), 24.8 (t, *J* = 10.1 Hz), 15.0; ^{19}F NMR (376 MHz, $CDCl_3$) δ -131.2 (t, *J* = 57.1 Hz); IR (neat): 3064, 3030, 2947, 2861, 1604, 1495, 1452, 1353, 1253, 1181, 1089, 1056, 1032, 913, 760, 703, 624, 516 cm^{-1}; HRMS (EI) exact mass calculated for [$C_{12}H_{14}F_2^+$]: 196.1064, found 196.1067

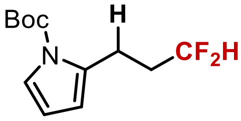

***tert*-Butyl 2-(3,3-difluoropropyl)-1*H*-pyrrole-1-carboxylate (10q)**. Method A. Purified using silica gel chromatography to give 41% yield of **10q** as a colorless oil; ^{1}H NMR (400 MHz, $CDCl_3$) δ 7.20 (dd, *J* = 3.4, 1.8 Hz, 1H), 6.08 (t, *J* = 3.3 Hz, 1H), 6.01 – 5.70 (m, 2H), 3.02 (dd, *J* = 9.0, 6.6 Hz, 2H), 2.24 – 2.10 (m, 2H), 1.59 (s, 9H); ^{13}C NMR (100 MHz, $CDCl_3$) δ 149.3, 133.5, 121.4, 117.1 (t, *J* = 232.3 Hz), 111.6, 110.0, 83.7, 33.5 (t, *J* = 20.2 Hz), 28.0, 21.9 (t, *J* = 10.1 Hz); ^{19}F NMR (376 MHz, $CDCl_3$) δ -116.7 (dt, *J* = 57.0, 17.1 Hz); IR (neat): 3435, 2980, 2936, 1742, 1334, 1128, 1055, 727, 487 cm^{-1}; HRMS (EI) exact mass calculated for [$C_{10}H_{10}F_2^+$]: 168.0751, found 168.0754.

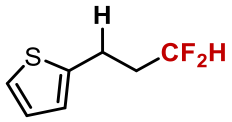

2-(3,3-Difluoropropyl)thiophene (10r). Method A. Purified using silica gel chromatography to give 71% yield of **10r** as a colorless oil; ^{1}H NMR (400 MHz, $CDCl_3$)δ 7.16 (dd, *J* = 5.1, 1.2 Hz, 1H), 6.94 (dd, *J* = 5.1, 3.4 Hz, 1H), 6.83 (dd, *J* = 3.4, 1.1 Hz, 1H), 5.85 (tt, *J* = 56.5, 4.5 Hz, 1H), 3.04 – 3.00 (m, 2H), 2.28 – 2.15 (m, 2H); ^{13}C NMR (100 MHz, $CDCl_3$) δ 142.4, 127.0, 124.8, 123.7, 116.3 (t, *J* = 242.4 Hz), 35.9 (t, *J* = 20.2 Hz), 22.7 (t, *J* = 10.1 Hz); ^{19}F NMR (376 MHz, $CDCl_3$) δ -117.5 (dt, *J* = 56.5, 17.0 Hz); IR (neat): 2923, 2853, 1734, 1461, 1374, 1083, 693, 461 cm^{-1}; HRMS (EI) exact mass calculated for [$C_{10}H_{12}F_2^+$]: 162.0315, found 162.0331.

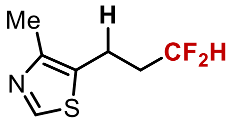

5-(3,3-Difluoropropyl)-4-methylthiazole (10s). Method A. Purified using silica gel chromatography to give 46% yield of **10s** as a yellow oil; ^{1}H NMR (400 MHz, $CDCl_3$) δ 8.61 (s, 1H), 5.86 (tt, *J* = 56.4, 4.3 Hz, 1H), 3.00 – 2.96 (m, 2H), 2.43 (s, 3H), 2.24 – 2.11 (m, 2H); ^{13}C NMR (100 MHz, $CDCl_3$) δ 149.5, 149.4, 129.1, 116.0 (t, *J* = 232.3 Hz), 35.7 (t, *J* = 20.2 Hz), 19.1 (t, *J* = 10.1 Hz), 14.8; ^{19}F NMR (376 MHz, $CDCl_3$) δ -117.6 (dt, *J* = 56.4, 17.0 Hz); IR (neat): 3079, 2929, 2858, 1719, 1545, 1412, 1121, 1063, 841 cm^{-1}; HRMS (EI) exact mass calculated for [$C_{10}H_{12}F_2^+$]: 170.0424, found 177.0433.

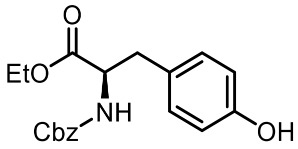

Ethyl ((benzyloxy)carbonyl)-D-tyrosinate. To a round bottom flask containing L-tyrosine (500 mg, 2.76 mmol) and EtOH (20 mL) was slowly added $SOCl_2$ (2 mL) at room temperature. The resulting mixture was stirred at 80 °C for 12 h. The reaction mixture was concentrated under reduced pressure, and the residue was used for following step without further pufification; ^{1}H NMR (300 MHz, DMSO-d_6) δ 9.43 (s, 1H), 8.46 (s, 2H), 7.01 (d, *J* = 8.4 Hz, 2H), 6.71 (d, *J* = 8.4 Hz, 2H), 4.22 – 4.03 (m, 3H), 3.10 – 2.85 (m, 2H), 1.13 (t, *J* = 7.1 Hz, 3H). Ethyl L-tyrosinate (577 mg, 2.8 mmol) and Na_2CO_3 (439 mg, 4.1 mmol) was dissolved in acetone/water, and benzyl chloroformate (0.47 mL, 3.3 mmol) was slowly added to the reaction mixture at 0 °C. The resulting mixture was stirred at room temperature for 24 h. The mixture was poured into water and extracted with ethyl acetate. The organic phase was dried with Mg_2SO_4, filtered, and concentrated under reduced pressure. The crude mixture was purified by a silica gel chromatography as a yellow oil (634 mg, 67 %); ^{1}H NMR (400 MHz, $CDCl_3$) δ 7.38 – 7.29 (m, 5H), 6.96 (d, *J* = 8.0 Hz, 2H), 6.71 (d, *J* = 7.9 Hz, 2H), 5.22 (d, *J* = 8.3 Hz, 1H), 5.13 – 5.06 (m, 2H), 4.90 (s, 1H), 4.59 (q, *J* = 6.8 Hz, 1H), 4.19 – 4.10 (m, 2H), 3.07 – 2.98 (m, 2H), 1.24 (t, *J* = 7.3 Hz, 3H); ^{13}C NMR (100 MHz, $CDCl_3$) δ 171.6, 155.7, 154.7, 136.3, 130.5, 128.6, 128.2, 128.1, 127.8, 115.4, 67.0, 61.5, 54.9, 37.5, 14.2; IR (neat): 3350, 3065, 3032, 2982, 1701, 1516, 1449, 1219, 1104, 1058, 911, 830, 741, 578, 541 cm^{-1} HRMS (EI) exact mass calculated for [$C_{19}H_{21}NO_5^+$]: 343.1420, found 343.1428.

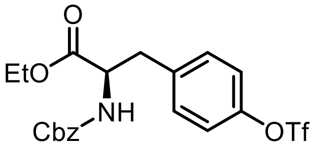

Ethyl (R)-2-(((benzyloxy)carbonyl)amino)-3-(4-(((trifluoromethyl)sulfonyl)oxy)phenyl)propanoate. To a round bottom flask containing ethyl ((benzyloxy)carbonyl)-D-tyrosinate (634 mg,1.8 mmol), diisopropylethylamine (0.34 mL, 2.0 mmol), and DCM (10 mL) was slowly added Tf_2O (0.39 mL, 2.2 mmol) at -15 ^oC. The reaction temperature was increased to room temperature, and the resulting mixture was stirred for 12 h. The mixture was poured into water and extracted with dichloromethane. The organic phase was dried with Mg_2SO_4, filtered, and concentrated under reduced pressure. The crude mixture was purified by a silica gel chromatography (hexane/ethyl acetate = 5/1) as a white solid (710 mg, 81%);m.p. 73 ^oC; ^1H NMR (400 MHz, $CDCl_3$) δ 7.39 – 7.31 (m, 5H), 7.20 – 7.15 (m, 4H), 5.28 (d, *J* = 8.5 Hz, 1H), 5.13 – 5.06 (m, 2H), 4.63 (q, *J* = 6.4 Hz, 1H), 4.18 – 4.11 (m, 2H), 3.14 (qd, *J* = 14.0, 6.1 Hz, 2H), 1.21 (t, *J* = 7.1 Hz, 3H); ^{13}C NMR (125 MHz, $CDCl_3$) δ 171.0, 155.5, 148.6, 136.6, 136.1, 131.2, 128.6, 128.3, 128.2, 121.4, 118.7 (q, *J* = 321.3 Hz), 67.1, 61.8, 54.6, 37.8, 14.1; IR (neat): 3341, 2979, 1743, 1691, 1532, 1501, 1414, 1348, 1253, 1204, 1136, 1057, 1017, 879, 841, 735, 695, 613, 575, 535, 497 cm^{-1}; HRMS (EI) exact mass calculated for [$C_{19}H_{21}NO_5^+$]: 475.0913, found 475.0916.

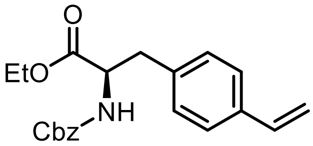

Ethyl (R)-2-(((benzyloxy)carbonyl)amino)-3-(4-vinylphenyl)propanoate. To an Ar-filled round bottom flask containing xx (710 mg, 1.5 mmol), $Pd(OAc)_2$ (17 mg, 5 mol%), SPhos (61 mg, 10 mol%), K_3PO_4 (951 mg, 4.5 mmol), and dioxane/water (6 mL) was slowly added 4,4,5,5-tetramethyl-2-vinyl-1,3,2-dioxaborolane (0.38 mL, 2.2 mmol). The reaction temperature was increased to 80 ^oC, and the resulting mixture was stirred for 24 h. The mixture was poured into water and extracted with ethyl acetate. The organic phase was dried with Mg_2SO_4, filtered, and concentrated under reduced pressure. The crude mixture was purified by a silica gel chromatography (hexane/dichloromethane= 4/1) as a white solid (350 mg, 66%); ^1H NMR (300 MHz, $CDCl_3$) δ 7.35 – 7.31 (m, 7H), 7.06 (d, *J* = 8.1 Hz, 2H), 6.68 (dd, *J* = 17.6, 10.9 Hz, 1H), 5.71 (dd, *J* = 17.6, 0.9 Hz, 1H), 5.24 – 5.21 (m, 2H), 5.10 (s, 2H), 4.63 (q, *J* = 6.4 Hz, 1H), 4.17 (q, *J* = 7.1 Hz, 2H), 3.10 (t, *J* = 5.8 Hz, 2H), 1.24 (t, *J* = 7.1 Hz, 3H); ^{13}C NMR (100 MHz, $CDCl_3$) δ 171.5, 155.6, 136.5, 136.4, 136.3, 135.4, 129.5, 128.5, 128.2, 128.1, 126.4, 113.8, 67.0, 61.6, 54.8, 38.0, 14.1.; IR (neat): 3342, 3086, 3032, 2981, 1725, 1512, 1450, 1400, 1256, 1210, 1057, 1028, 910, 830, 775, 741, 699, 579, 487 cm^{-1}; HRMS (EI) exact mass calculated for [$C_{21}H_{23}NO_4^+$]: 353.1627, found 353.1616.

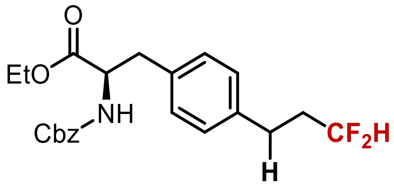

Ethyl (R)-2-(((benzyloxy)carbonyl)amino)-3-(4-(3,3-difluoropropyl)phenyl)propanoate (10t). Method A. Purified using silica gel chromatography to give 50% yield of **10t** as a colorless oil; ^{1}H NMR (400 MHz, $CDCl_3$) δ 7.37 – 7.30 (m, 5H), 7.10 (d, *J* = 8.1 Hz, 2H), 7.04 (d, *J* = 8.0 Hz, 2H), 5.79 (tt, *J* = 56.7, 4.5 Hz, 1H), 5.21 (d, *J* = 8.2 Hz, 1H), 5.13 – 5.06 (m, 2H), 4.65 – 4.60 (m, 1H), 4.17 (q, *J* = 7.0 Hz, 2H), 3.14 – 3.04 (m, 2H), 2.76 – 2.72 (m, 2H), 2.19 – 2.05 (m, 2H), 1.24 (t, *J* = 7.2 Hz, 3H); ^{13}C NMR (100 MHz, $CDCl_3$) δ 171.5, 155.6, 138.7, 136.3, 133.8, 129.6, 128.6, 128.5, 128.2, 128.1, 116.7 (t, *J* = 242.4 Hz), 67.0, 61.5, 54.8, 37.9, 35.6 (t, *J* = 20.2 Hz), 27.9 (t, *J* = 10.1 Hz), 14.1; ^{19}F NMR (376 MHz, $CDCl_3$) -117.1 (dt, *J* = 56.6, 17.1 Hz); IR (neat): 3431, 3343, 2980, 2936, 1725, 1513, 1207, 1055, 741 cm^{-1}; HRMS (FAB) exact mass calculated for [$C_{22}H_{25}F_2NO_4^+$]: 405.1752, found 406.1822.

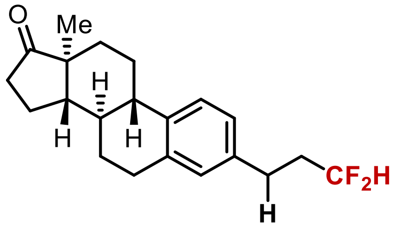

3-(3,3-Difluoropropyl)-13-methyl-6,7,8,9,11,12,13,14,15,16-decahydro-17*H*-cyclopenta[a]phenanthren-17-one (10u). Method A. Purified using silica gel chromatography to give 52% yield of **10u** as a white solid; m.p. 79 °C; ^{1}H NMR (400 MHz, $CDCl_3$) δ 7.24 (d, *J* = 8.1 Hz, 1H), 6.99 (d, *J* = 7.7 Hz, 1H), 6.94 (s, 1H), 5.81 (tt, *J* = 56.7, 4.5 Hz, 1H), 2.90 (dd, *J* = 9.0, 4.2 Hz, 2H), 2.72 (dd, *J* = 9.4, 6.7 Hz, 2H), 2.51 (dd, *J* = 18.7, 8.7 Hz, 1H), 2.45 – 2.40 (m, 1H), 2.31 – 2.26 (m, 1H), 2.20 – 1.94 (m, 6H), 1.68 – 1.41 (m, 6H), 0.91 (s, 3H); ^{13}C NMR (126 MHz, $CDCl_3$) δ 221.1, 138.0, 137.5, 136.9, 129.1, 125.9, 125.8, 116.9 (t, *J* = 239.4 Hz), 50.6, 48.1, 44.4, 38.3, 36.0, 35.8 (t, *J* = 21.0 Hz), 31.7, 29.5, 28.0 (t, *J* = 6.0 Hz), 26.6, 25.9, 21.7, 14.0; ^{19}F NMR (376 MHz, $CDCl_3$) δ -117.1 (dt, *J* = 57.0, 17.1 Hz); IR (neat): 2928, 2865, 1736, 1373, 1121, 1053, 822 cm^{-1}; HRMS (EI) exact mass calculated for [$C_{21}H_{26}F_2O^+$]: 332.1952, found 332.1948.

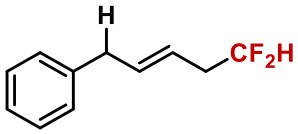

(*E*)-(5,5-Difluoropent-2-en-1-yl)benzene (10v). Method B. Purified using silica gel chromatography to give **10v** as a colorless oil (correct yield was measured by ^{1}H NMR spectroscopy with CH+Br$_2$ as an internal standard because of highly volatile nature of the product); ^{1}H NMR (300 MHz, CDCl$_3$) δ 7.34 – 7.27 (m, 2H), 7.25 – 7.15 (m, 3H), 6.01 – 5.37 (m, 3H), 3.39 (d, *J* = 6.7 Hz, 2H), 2.71 – 2.48 (m, 2H); ^{13}C NMR (125 MHz) δ 139.9, 135.2, 128.5, 128.5, 126.2, 121.2 (t, *J* = 6.7 Hz), 116.4 (t, *J* = 241.7 Hz), 39.0, 37.6 (t, *J* = 21.7 Hz); ^{19}F NMR (282 MHz, CDCl$_3$) δ -115.6 – -116.0 (m, 2F); IR (Film): 3025, 2924, 2853, 1456, 1097, 968, 801, 746, 699, 467 cm^{-1}; HRMS (EI) exact mass calculated for [M$^+$, $C_{11}H_{12}F_2^+$]: 182.0907, found 182.0878.

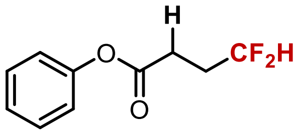

Phenyl 4,4-difluorobutanoate (10w). Method B. Purified using silica gel chromatography to give 36% yield of **10w** as a colorless oil; ^{1}H NMR (300 MHz, CDCl$_3$) δ 7.44 – 7.35 (m, 2H), 7.29 – 7.19 (m, 1H), 7.11 – 7.05 (m, 2H), 6.01 (tt, *J* = 56.5, 4.1 Hz, 1H), 2.78 (t, *J* = 7.4 Hz, 2H), 2.29 (ttd, *J* = 17.5, 7.4, 4.1 Hz, 2H); ^{13}C NMR (75 MHz, CDCl$_3$) δ 170.6, 150.4, 129.5, 126.0, 121.4, 115.9 (t, *J* = 239.3 Hz), 29.3 (t, *J* = 22.3 Hz), 26.9 (t, *J* = 6.0 Hz); ^{19}F NMR (282 MHz, CDCl$_3$) δ -117.7 (dt, *J* = 56.6, 17.4 Hz); IR (Film): 2925, 2853, 1760, 1594, 1493, 1442, 1380, 1198, 1164, 1147, 1121, 1069, 945, 810, 754, 691, 499, 453 cm^{-1}; HRMS (EI) exact mass calculated for [M$^+$, $C_{10}H_{10}F_2O_2^+$]: 200.0649, found 200.0644.

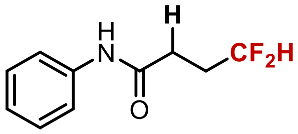

4,4-Difluoro-N-phenylbutanamide (10x). Method B. Purified using silica gel chromatography to give 40% yield of **10x** as a white solid; ^{1}H NMR (300 MHz, CDCl$_3$) δ 7.49 (d, *J* = 7.8 Hz, 2H), 7.32 (t, *J* = 7.9 Hz, 3H), 7.12 (t, *J* = 7.4 Hz, 1H), 5.99 (tt, *J* = 56.7, 4.1 Hz, 1H), 2.55 (t, *J* = 7.3 Hz, 2H), 2.40 – 2.15 (m, 2H); ^{13}C NMR (75 MHz, CDCl$_3$) δ 169.1, 137.5, 129.1, 124.6, 120.0, 116.3 (t, *J* = 239.0 Hz), 29.9 – 28.8 (m, 2C); ^{19}F NMR (282 MHz, CDCl$_3$) δ -117.5 (dt, *J* = 56.7, 17.6 Hz); IR (Film): 3307, 1660, 1600, 1543, 1500, 1446, 1409, 1380, 1316, 1265, 1122, 1067, 956, 912, 759, 723, 694, 504, 477, 450 cm^{-1}; HRMS (EI) exact mass calculated for [M$^+$, $C_{10}H_{11}F_2NO^+$]: 199.0809, found 199.0815.

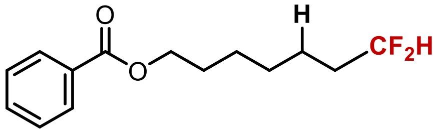

7,7-Difluoroheptyl benzoate (12a). Method C. Purified using silica gel chromatography to give 56% yield of **12a** as a colorless oil; ^{1}H NMR (300 MHz, $CDCl_3$) δ 8.10 – 7.98 (m, 2H), 7.62 – 7.52 (m, 1H), 7.49 – 7.38 (m, 2H), 5.80 (tt, J = 56.9, 4.5 Hz, 1H), 4.32 (t, J = 6.6 Hz, 2H), 1.95 – 1.70 (m, 4H), 1.57 – 1.39 (m, 6H); ^{13}C NMR (75 MHz, $CDCl_3$) δ 166.6, 132.9, 130.4, 129.5, 128.3, 117.3 (t, J = 238.7 Hz), 64.9, 34.0 (t, J = 20.7 Hz), 28.7, 28.5, 25.8, 22.0 (t, J = 5.4 Hz); ^{19}F NMR (282 MHz, $CDCl_3$) δ -115.8 (dt, J = 57.1, 17.8 Hz); IR (Film): 2931, 2862, 1719, 1602, 1453, 1401, 1315, 1275, 1113, 1069, 960, 839, 712, 557, 462 cm^{-1}; HRMS (EI) exact mass calculated for [M$^+$, $C_{14}H_{18}F_2O_2{}^+$]: 256.1275, found 256.1289.

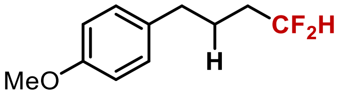

1-(4,4-Difluorobutyl)-4-methoxybenzene (12b). Method C. Purified using silica gel chromatography to give 48% yield of **12b** as a colorless oil; ^{1}H NMR (300 MHz, $CDCl_3$) δ 7.13 – 7.03 (m, 2H), 6.90 – 6.73 (m, 2H), 5.79 (tt, J = 56.7, 4.2 Hz, 1H), 3.79 (s, 3H), 2.62 (t, J = 7.2 Hz, 2H), 1.95 – 1.66 (m, 4H); ^{13}C NMR (75 MHz, $CDCl_3$) δ 157.9, 133.3, 129.3, 117.3 (t, J = 238.9 Hz), 113.8, 55.3, 34.2, 33.4 (t, J = 20.8 Hz), 23.9 (t, J = 5.2 Hz); ^{19}F NMR (282 MHz, $CDCl_3$) δ -115.7 (dt, J = 56.8, 17.1 Hz); IR (Film): 2956, 2864, 2839, 2061, 1884, 1613, 1583, 1513, 1463, 1442, 1403, 1371, 1300, 1248, 1179, 1121, 1037, 977, 833, 749, 696, 558, 516, 426 cm^{-1}; HRMS (EI) exact mass calculated for [M$^+$, $C_{11}H_{14}F_2O$ $^+$]: 200.1013, found 200.1009.

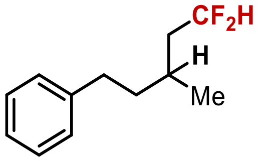

(5,5-Difluoro-3-methylpentyl)benzene (12c). Method C. Purified using silica gel chromatography to give 64% yield of **12c** as a colorless oil; ^{1}H NMR (300 MHz, $CDCl_3$) δ 7.33 – 7.26 (m, 2H), 7.21-7.17 (m, 3H), 5.87 (tdd, J = 56.9, 5.3, 4.3 Hz, 1H), 2.64 (qdd, J = 13.7, 10.1, 6.0 Hz, 2H), 2.05 – 1.44 (m, 5H), 1.04 (d, J = 6.3 Hz, 3H); ^{13}C NMR (75 MHz, $CDCl_3$) δ 142.2, 128.4, 128.3, 125.8, 117.1 (t, J = 238.6 Hz), 40.8 (t, J = 19.9 Hz), 38.8, 33.1, 27.6 (t, J = 5.2 Hz), 19.6; ^{19}F NMR (282 MHz, $CDCl_3$) δ -114.5 (dtd, J = 28.3, 16.6, 10.4 Hz); IR (Film): 2921, 2851, 1007, 788, 447 cm^{-1}; HRMS (EI) exact mass calculated for [M$^+$, $C_{12}H_{16}F_2{}^+$]: 198.1220, found 198.1219.

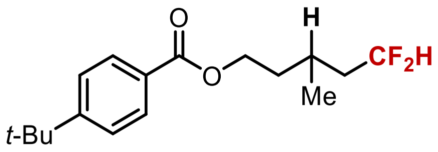

5,5-difluoro-3-methylpentyl 4-(tert-butyl)benzoate (12d). Method C. Purified using silica gel chromatography to give 72% yield of **12d** as a colorless oil; ^{1}H NMR (300 MHz, $CDCl_3$) δ 7.96 (d, *J* = 8.6 Hz, 2H), 7.46 (d, *J* = 8.6 Hz, 2H), 5.90 (tt, *J* = 56.8, 4.7 Hz, 1H), 4.42 – 4.22 (m, 2H), 2.08 – 1.57 (m, 5H), 1.34 (s, 9H), 1.07 (d, *J* = 6.5 Hz, 3H); ^{13}C NMR (75 MHz, $CDCl_3$) δ 166.6, 156.6, 129.4, 127.4, 125.4, 116.8 (t, *J* = 238.8 Hz), 62.4, 40.8 (t, *J* = 20.1 Hz), 35.5, 35.1, 31.1, 25.2 (t, *J* = 5.4 Hz), 19.5; ^{19}F NMR (282 MHz, $CDCl_3$) δ -113.1 – -115.4 (m); IR (Film): 2964, 2930, 2872, 1720, 1611, 1572, 1464, 1407, 1367, 1276, 1189, 1119, 1039, 973, 856, 776, 708, 547 cm^{-1}; HRMS (EI) exact mass calculated for [M^+, $C_{17}H_{24}F_2O_2^+$]: 298.1744, found 298.1744.

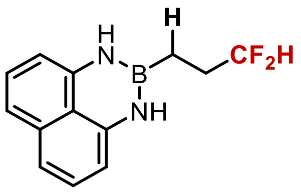

2-(3,3-Difluoropropyl)-2,3-dihydro-1H-naphtho[1,8-de][1,3,2]diazaborinine (12e). Method C. Purified using silica gel chromatography to give 58% yield of **12e** as a blue solid; ^{1}H NMR (300 MHz, $CDCl_3$) δ 7.08 (m, 4H), 6.32 (dd, *J* = 7.2, 1.0 Hz, 2H), 6.10 – 5.47 (m, 3H), 2.09 – 1.83 (m, 2H), 1.07 – 0.79 (m, 2H); ^{11}B NMR (96 MHz, $CDCl_3$) δ 31.54 (s, 1B); ^{13}C NMR (75 MHz, $CDCl_3$) δ 140.7, 136.2, 127.5, 119.5, 117.8 (t, *J* = 239.6 Hz), 117.7, 105.7, 29.4 (t, *J* = 21.5 Hz); ^{19}F NMR (282 MHz, $CDCl_3$) δ -116.5 (dt, *J* = 57.1, 17.3 Hz); IR (Film): 3441, 3392, 3052, 2926, 1601, 1511, 1443, 1412, 1372, 1344, 1270, 1235, 1186, 1167, 1116, 1053, 889, 821, 766, 641, 600, 456 cm^{-1}; HRMS (EI) exact mass calculated for [M^+, $C_{13}H_{13}BF_2N_2^+$]: 246.1140, found 246.1154.

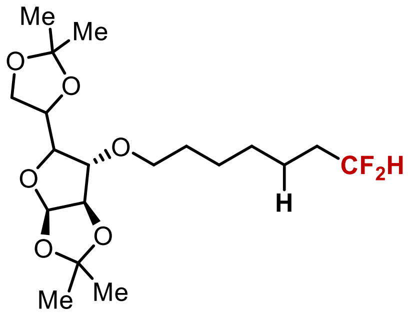

(3aR,6S,6aR)-6-((7,7-Difluoroheptyl)oxy)-5-(2,2-dimethyl-1,3-dioxolan-4-yl)-2,2-dimethyltetrahydrofuro[2,3-d][1,3]dioxole (12f). Method C. Purified using silica gel chromatography to give 72% yield of **12f** as a colorless oil; ^{1}H NMR (300 MHz, $CDCl_3$) δ 5.87 (d, *J* = 3.7 Hz, 1H), 5.79 (tt, *J* = 56.9, 4.5 Hz, 1H), 4.52 (d, *J* = 3.7 Hz, 1H), 4.29 (dt, *J* = 7.6, 6.0 Hz, 1H), 4.15 – 4.03 (m, 2H), 3.98 (dd, *J* = 8.5, 5.9 Hz, 1H), 3.84 (d, *J* = 3.1 Hz, 1H), 3.66 – 3.42 (m, 2H), 1.81 (qdd, *J* = 13.3, 9.8, 5.5 Hz, 2H), 1.58 – 1.51 (m, 2H), 1.49 (s, 3H), 1.48 – 1.44 (m, 1H), 1.42 (s, 3H), 1.40 – 1.36 (m, 4H), 1.34 (s, 3H), 1.31 (s, 3H), 1.28– 1.23 (m, 1H); ^{13}C NMR (75 MHz, $CDCl_3$) δ 117.3 (t, *J* = 238.7 Hz), 111.7, 108.9, 105.2, 82.5, 82.1, 81.2, 72.5, 70.4, 67.2, 34.0 (t, *J* = 20.6 Hz), 29.5, 28.8, 26.8, 26.8, 26.2, 25.8, 25.4, 22.0 (t, *J* = 5.4 Hz); ^{19}F NMR (282 MHz, $CDCl_3$) δ -115.8 (dt, *J* = 57.0, 17.6 Hz); IR (Film): 2986, 2937, 2867, 1458, 1376, 1254, 1216, 1165, 1126, 1078, 1022, 886, 850, 637, 512 cm^{-1}; HRMS (EI) exact mass calculated for [M^+, $C_{19}H_{32}F_2O_6^+$]: 394.2167, found 394.2147.

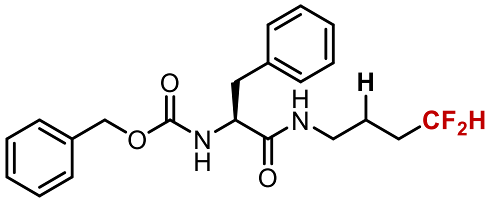

Benzyl (S)-(1-((4,4-difluorobutyl)amino)-1-oxo-3-phenylpropan-2-yl)carbamate (12g). Method C. Purified using silica gel chromatography to give 60% yield of **12g** as a white solid; ^{1}H NMR (300 MHz, $CDCl_3$) δ 7.37 – 7.29 (m, 7H), 7.24 – 7.15 (m, 3H), 6.05 – 5.39 (m, 3H), 5.07 (s, 2H), 4.36 (dd, J = 14.3, 7.7 Hz, 1H), 3.29 – 2.92 (m, 4H), 1.74 – 1.56 (m, 2H), 1.54 – 1.42 (m, 2H); ^{13}C NMR (75 MHz, $CDCl_3$) δ 170.9, 156.0, 136.4, 136.0, 129.2, 128.7, 128.5, 128.3, 128.0, 127.1, 116.7 (t, *J* = 239.1 Hz), 67.1, 56.5, 38.6, 38.6, 31.1 (t, *J* = 21.3 Hz), 21.9 (t, *J* = 5.3 Hz); ^{19}F NMR (282 MHz, $CDCl_3$) δ -116.1 (dt, *J* = 56.6, 17.5 Hz); IR (Film): 3301, 3035, 2929, 1689, 1654, 1533, 1453, 1384, 1286, 1242, 1119, 1077, 1036, 913, 847, 749, 699, 664, 572, 520, 481, 429 cm^{-1}; HRMS (EI) exact mass calculated for [M^+, $C_{21}H_{24}F_2N_2O_3^+$]: 390.1755, found 309.1760.

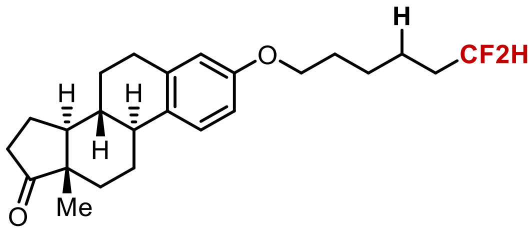

(8R,9S,13S,14S)-3-((6,6-difluorohexyl)oxy)-13-methyl-6,7,8,9,11,12,13,14,15,16-decahydro-17H-cyclopenta[a]phenanthren-17-onecarbamate (12h). Method C. Purified using silica gel chromatography to give 44% yield of **12h** as a white solid; ^{1}H NMR (300 MHz, $CDCl_3$) δ 7.20 (d, *J* = 8.6 Hz, 1H), 6.71 (dd, *J* = 8.6, 2.7 Hz, 1H), 6.64 (d, *J* = 2.6 Hz, 1H), 5.81 (tt, *J* = 56.9, 4.5 Hz, 1H), 3.94 (t, *J* = 6.3 Hz, 2H), 2.96 – 2.80 (m, 2H), 2.57 – 2.45 (m, 1H), 2.44 – 2.34 (m, 1H), 2.31 – 2.10 (m, 2H), 2.10 – 1.72 (m, 7H), 1.69 – 1.38 (m, 10H), 0.91 (s, 3H); ^{13}C NMR (75 MHz, $CDCl_3$) δ 221.0, 157.0, 137.7, 132.0, 126.3, 117.3 (t, *J* = 238.8 Hz), 114.5, 112.0, 67.4, 50.3, 48.0, 44.0, 38.3, 35.8, 34.0 (t, *J* = 20.7 Hz), 31.5, 29.6, 29.1, 26.5, 25.9, 25.6, 21.8 (t, *J* = 5.5 Hz), 21.6, 13.8; ^{19}F NMR (282 MHz, $CDCl_3$) δ -115.8 (dt, *J* = 56.9, 17.6 Hz); IR (Film): 3301, 3035, 2929, 1689, 1654, 1533, 1453, 1384, 1286, 1242, 1119, 1077, 1036, 913, 847, 749, 699, 664, 572, 520, 481 cm^{-1}; HRMS (EI) exact mass calculated for [M$^+$, $C_{24}H_{32}F_2O_2^+$]: 390.2370, found 390.2348.

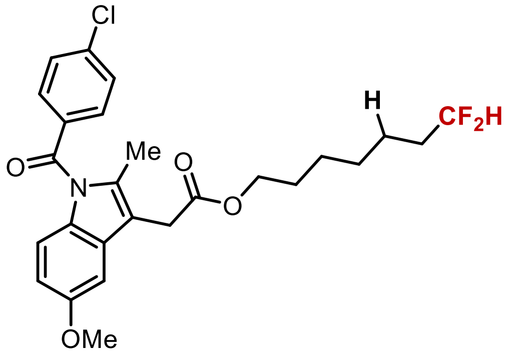

7,7-Difluoroheptyl 2-(1-(4-chlorobenzoyl)-5-methoxy-2-methyl-1H-indol-3-yl)acetate (12i). Method C. Purified using silica gel chromatography to give 30% yield of **12i** as a yellow oil; ^{1}H NMR (300 MHz, $CDCl_3$) δ 7.66 (d, *J* = 8.5 Hz, 2H), 7.46 (d, *J* = 8.5 Hz, 2H), 6.97 (d, *J* = 2.5 Hz, 1H), 6.86 (d, *J* = 9.0 Hz, 1H), 6.66 (dd, *J* = 9.0, 2.5 Hz, 1H), 5.77 (tt, *J* = 56.9, 4.5 Hz, 1H), 4.10 (t, *J* = 6.6 Hz, 2H), 3.83 (s, 3H), 3.66 (s, 2H), 2.39 (s, 3H), 1.88 – 1.68 (m, 2H), 1.62 (t, *J* = 6.7 Hz, 2H), 1.44 – 1.27 (m, 6H); ^{13}C NMR (75 MHz, $CDCl_3$) δ 170.9, 168.3, 156.0, 139.2, 135.9, 133.9, 131.1, 130.8, 130.6, 129.1, 117.3 (t, *J* = 238.8 Hz), 114.9, 112.7, 111.5, 101.4, 64.9, 55.7, 33.9 (t, *J* = 20.7 Hz), 30.4, 28.6, 28.4, 25.6, 21.9 (t, *J* = 5.4 Hz), 13.3; ^{19}F NMR (282 MHz, $CDCl_3$) δ -115.8 (dt, *J* = 57.0, 17.7 Hz); IR (Film): 2936, 2862, 1734, 1685, 1595, 1478, 1399, 1359, 1321, 1261, 1224, 1168, 1143, 1089, 1067, 1038, 1017, 997, 925, 837, 806, 755, 691, 563, 482 cm^{-1}; HRMS (EI) exact mass calculated for [M$^+$, $C_{26}H_{28}ClF_2NO_4^+$]: 491.1675, found 491.1682.

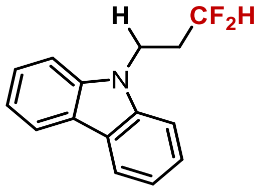

9-(3,3-Difluoropropyl)-9H-carbazole (12j). Method C. Purified using silica gel chromatography to give 66% yield of **12j** as a white solid; ^1H NMR (300 MHz, $CDCl_3$) δ 8.12 (d, *J* = 7.7 Hz, 2H), 7.53 – 7.47 (m, 2H), 7.42 (d, *J* = 8.1 Hz, 2H), 7.30 – 7.24 (m, 2H), 5.79 (tt, *J* = 56.0, 4.4 Hz, 1H), 4.53 (t, *J* = 6.9 Hz, 2H), 2.55 – 2.24 (m, 2H); ^{13}C NMR (75 MHz, $CDCl_3$) δ 139.9, 126.0, 123.1, 120.5, 119.4, 115.5 (t, *J* = 239.2 Hz), 108.2, 36.4 (t, *J* = 6.9 Hz), 33.5 (t, *J* = 21.5 Hz); ^{19}F NMR (282 MHz, $CDCl_3$) δ -117.8 (dt, *J* = 56.0, 17.1 Hz); IR (Film): 3055, 2926, 2854, 1597, 1484, 1457, 1381, 1353, 1331, 1242, 1201, 1154, 1121, 1096, 1060, 961, 858, 755, 727, 694, 617, 587, 502, 425 cm^{-1}; HRMS (EI) exact mass calculated for [M^+, $C_{15}H_{13}F_2N^+$]: 245.1016, found 245.1034.

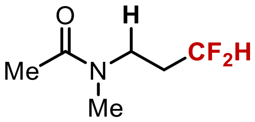

N-(3,3-Difluoropropyl)-N-methylacetamide (12k). Method C. 1H NMR analysis [integration of methyl resonances at 3.02 (major) and 2.92 (minor) ppm] of the unpurified reaction. Purified using silica gel chromatography to give 48% yield of **12k** as a colorless oil; ^1H NMR (300 MHz, $CDCl_3$) δ 5.88 (tt, *J* = 56.3, 4.3 Hz, 1H), 3.51 (t, *J* = 7.0 Hz, 2H), 3.02 (s, 3H), 2.07 (s, 3H), 2.10 – 2.00 (m, 2H); ^{13}C NMR (75 MHz, $CDCl_3$) δ 170.8, 116.1 (t, *J* = 239.1 Hz), 41.8 (t, *J* = 6.6 Hz), 36.6, 32.2 (t, *J* = 20.9 Hz), 21.81; ^{19}F NMR (282 MHz, $CDCl_3$) δ -116.0 (dt, *J* = 56.3, 17.5 Hz); IR (Film): 2925, 2854, 1724, 1648, 1458, 1407, 1378, 1311, 1217, 1120, 1066, 753, 594, 568, 475 cm^{-1}; HRMS (EI) exact mass calculated for [M^+, $C_6H_{11}F_2NO^+$]: 151.0809, found 151.0812.

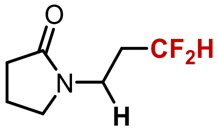

1-(3,3-Difluoropropyl)pyrrolidin-2-one (12l). Method C. Purified using silica gel chromatography to give 44% yield of **12l** as a colorless oil; ^1H NMR (300 MHz, $CDCl_3$) δ 5.88 (tt, *J* = 56.2, 4.4 Hz, 1H), 3.48 – 3.38 (m, 4H), 2.38 (t, *J* = 8.1 Hz, 2H), 2.20 – 1.92 (m, 4H); ^{13}C NMR (75 MHz, $CDCl_3$) δ 175.3, 115.9 (t, *J* = 239.4 Hz), 47.3, 36.5 (t, *J* = 6.7 Hz), 32.1 (t, *J* = 21.1 Hz), 30.8, 18.0; ^{19}F NMR (282 MHz, $CDCl_3$) δ -116.2 (dt, *J* = 56.2, 17.2 Hz); IR (Film): 2924, 2853, 1677, 1499, 1467, 1431, 1379, 1293, 1190, 1100, 951, 919, 624, 472 cm^{-1}; HRMS (EI) exact mass calculated for [M^+, $C_7H_{11}F_2NO^+$]: 163.0809, found 163.0806.

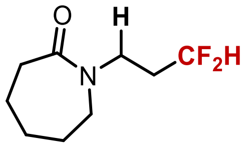

1-(3,3-Difluoropropyl)azepan-2-one (12m). Method C. Purified using silica gel chromatography to give 60% yield of **12m** as a colorless oil; ^{1}H NMR (300 MHz, $CDCl_3$) δ 5.87 (tt, *J* = 56.3, 4.4 Hz, 1H), 3.55 – 3.47 (m, 2H), 3.37 – 3.31 (m, 2H), 2.59 – 2.37 (m, 2H), 2.15 – 1.96 (m, 2H), 1.75 – 1.54 (m, 6H); ^{13}C NMR (75 MHz, $CDCl_3$) δ 176.0, 116.1 (t, *J* = 239.1 Hz), 50.2, 42.6 (t, *J* = 6.8 Hz), 37.1, 33.0 (t, *J* = 20.8 Hz), 29.9, 28.5, 23.2; ^{19}F NMR (282 MHz, $CDCl_3$) δ -115.7 (dt, *J* = 56.3, 17.3 Hz); IR (Film): 2931, 2857, 1632, 1494, 1444, 1370, 1331, 1264, 1233, 1197, 1118, 979, 847, 723, 624, 577, 479, 451 cm^{-1}; HRMS (EI) exact mass calculated for [M^+, $C_9H_{15}F_2NO^+$]: 191.1122, found 191.1131.

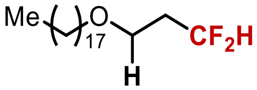

1-(3,3-Difluoropropoxy)octadecane (12n). Method C. Purified using silica gel chromatography to give 30% yield of **12n** as a white solid; ^{1}H NMR (300 MHz, $CDCl_3$) δ 5.96 (tt, *J* = 57.0, 4.8 Hz, 1H), 3.55 (t, *J* = 6.1 Hz, 2H), 3.41 (t, *J* = 6.6 Hz, 2H), 2.21 – 1.90 (m, 2H), 1.67 – 1.48 (m, 4H), 1.25 (s, 28H), 0.88 (t, *J* = 6.7 Hz, 3H); ^{13}C NMR (125 MHz, $CDCl_3$) δ 116.0 (t, *J* = 238.0 Hz), 71.4 , 64.4 (t, *J* = 6.8 Hz), 34.8 (t, *J* = 21.3 Hz), 31.9, 29.7 – 29.6 (m, 8C), 29.6, 29.6, 29.6, 29.4, 29.4, 26.1, 22.7, 14.1; ^{19}F NMR (282 MHz, $CDCl_3$) δ -117.9 (dt, *J* = 56.9, 16.5 Hz); IR (Film): 2925, 2855, 1735, 1464, 1401, 1375, 1260, 1119, 979, 802, 722 cm^{-1}; HRMS (EI) exact mass calculated for [M^+, $C_{21}H_{42}F_2O^+$]: 348.3204, found 348.3226.

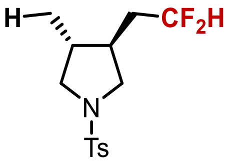

(3R,4R)-3-(2,2-Difluoroethyl)-4-methyl-1-tosylpyrrolidine (14). Method C. Isolated as a 2.5:1 mixture of diastereomers. Purified using silica gel chromatography to give 88% yield of **14** as a colorless oil; ^{1}H NMR (300 MHz, $CDCl_3$) δ 7.73 (d, *J* = 8.3 Hz, 2H), 7.35 (d, *J* = 8.4 Hz, 2H), 5.79 (tt, *J* = 56.3, 4.2 Hz, 1H), 3.65 – 3.34 (m, 2H), 3.11 – 2.76 (m, 2H), 2.45 (s, 3H), 2.30 – 1.53 (m, 4H), 0.98 – 0.76 (d, *J* = 6.5 Hz, 3H); ^{13}C NMR (75 MHz, $CDCl_3$) δ 143.49, 143.47, 133.9, 133.7, 129.7, 129.7, 127.4, 127.3, 116.3 (t, *J* = 239.8 Hz), 116.0 (t, *J* = 239.6 Hz), 54.5, 54.0, 53.2, 50.7, 39.6 (t, *J* = 4.4 Hz), 38.9, 36.1 (t, *J* = 21.2 Hz), 35.7 (t, *J* = 4.6 Hz), 35.3, 32.4 (t, *J* = 21.2 Hz), 21.5, 15.7, 13.3; ^{19}F NMR (282 MHz, $CDCl_3$) δ -114.2 – -117.2 (m, 2F); IR (Film): 2964, 2927, 1598, 1480, 1438, 1407, 1385, 1342, 1307, 1215, 1163, 1120, 1095, 1045, 976, 816, 708, 665, 592, 549, 492, 427 cm^{-1}; HRMS (EI) exact mass calculated for [M^+, $C_{14}H_{19}F_2NO_2S^+$]: 303.1105, found 303.1102.

Spectroscopic data is in accordance with previous reports.[13]

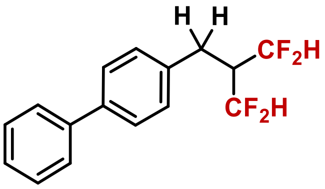

4-(2-(Difluoromethyl)-3,3-difluoropropyl)-1,1'-biphenyl (**16a**). Method D. Purified using silica gel chromatography to give 43% yield of **16** as a white solid; ^{1}H NMR (400 MHz, $CDCl_3$) δ 7.60 – 7.56 (m, 4H), 7.47 – 7.44 (m, 2H), 7.38 – 7.30 (m, 3H), 6.11 – 5.80 (m, 2H), 3.02 (d, *J* = 7.2 Hz, 2H), 2.76 – 2.56 (m, 1H); ^{13}C NMR (100 MHz, $CDCl_3$) δ 140.7, 140.1, 136.1, 129.6, 128.9, 127.7, 127.5, 127.2, 114.7 (tt, *J* = 243.3, 6.0 Hz), 48.8 (p, *J* = 19.8 Hz), 27.7 (p, *J* = 4.2 Hz); ^{19}F NMR (376 MHz, $CDCl_3$) δ -123.8 – -124.0 (m); IR (Film): 3031, 2997, 2945, 1955, 1599, 1489, 1452, 1403, 1274, 1144, 1086, 931, 849, 763, 696, 658, 555, 496 cm^{-1}; HRMS (EI) exact mass calculated for [M^+, $C_{16}H_{14}F_4^+$]: 282.1032, found 282.1036.

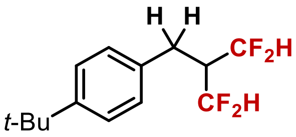

1-(*tert*-Butyl)-4-(2-(difluoromethyl)-3,3-difluoropropyl)benzene (**16b**). Method D. Purified using preparative thin layer chromatography to give **16b** as a colorless oil; ^{1}H NMR (400 MHz, $CDCl_3$) δ 7.37 (d, *J* = 7.9 Hz, 2H), 7.17 (d, *J* = 7.9 Hz, 2H), 6.07 – 5.78 (m, 2H), 2.95 (d, *J* = 7.3 Hz, 2H), 2.71 – 2.54 (m, 1H), 1.34 (s, 9H); ^{13}C NMR (100 MHz, $CDCl_3$) δ 150.1, 133.9, 128.9, 125.9, 114.7 (tt, *J* = 243.6, 6.1 Hz), 48.7 (p, *J* = 19.7 Hz), 34.6, 31.5, 27.6 (p, *J* = 4.3 Hz); ^{19}F NMR (376 MHz, $CDCl_3$) δ -123.9 – -124.2 (m); IR (Film): 2965, 2909, 2873, 1514, 1457, 1404, 1389, 1269, 1142, 1063, 1144, 1086, 931, 844, 573 cm^{-1}; HRMS (EI) exact mass calculated for [M^+, $C_{14}H_{18}F_4^+$]: 262.1345, found 262.1341.

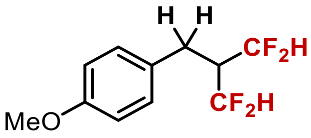

1-(2-(Difluoromethyl)-3,3-difluoropropyl)-4-methoxybenzene (**16c**). Method D. Purified using preparative thin layer chromatography to give **16c** as a colorless oil; ^{1}H NMR (400 MHz, $CDCl_3$) δ 7.14 (d, *J* = 7.7 Hz, 2H), 6.86 (d, *J* = 7.5 Hz, 2H), 6.03 – 5.74 (m, 2H), 3.80 (s, 3H), 2.91 (d, *J* = 7.1 Hz, 2H), 2.62 – 2.52 (m, 1H); ^{13}C NMR (100 MHz, $CDCl_3$) δ 158.7, 130.2, 128.9, 114.7 (tt, *J* = 242.3, 5.8 Hz), 114.4, 55.4, 48.9 (p, *J* = 19.5 Hz), 27.2 (p, *J* = 4.2 Hz); ^{19}F NMR (376 MHz, $CDCl_3$) δ -124.0 (dd, *J* = 55.7, 14.8 Hz); IR (Film): 2996, 2961, 2941, 2840, 2601, 2059, 1890, 1673, 1612, 1512, 1458, 1386, 1299, 1249, 1180, 1136, 1057, 1029, 838, 772, 715, 688, 648, 563, 523, 498, 428 cm^{-1}; HRMS (EI) exact mass calculated for [M^+, $C_{11}H_{12}F_4O^+$]: 236.0824, found 236.0809.

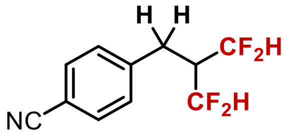

4-(2-(Difluoromethyl)-3,3-difluoropropyl)benzonitrile (**16d**). Method D. Purified using preparative thin layer chromatography to give **16d** as a colorless oil; ^{1}H NMR (400 MHz, $CDCl_3$) δ 7.63 (d, *J* = 6.6 Hz, 2H), 7.36 (d, *J* = 8.0 Hz, 2H), 6.07 – 5.77 (m, 2H), 3.03 (d, *J* = 6.8 Hz, 2H), 2.70 – 2.60 (m, 1H); ^{13}C NMR (100 MHz, $CDCl_3$) δ 143.0, 132.7, 130.0, 118.7, 114.4 (tt, *J* = 242.1, 6.5 Hz), 111.3, 48.7 (p, *J* = 19.7 Hz), 28.0 (p, *J* = 3.9 Hz); ^{19}F NMR (376 MHz, $CDCl_3$) δ -121.9 – -124.7 (m); IR (Film): 3042, 2991, 2230, 1610, 1508, 1452, 1406, 1141, 1063, 1027, 932, 886, 853, 820, 563 cm^{-1}; HRMS (EI) exact mass calculated for [M^+, $C_{11}H_9F_4N^+$]: 231.0671, found 231.0676.

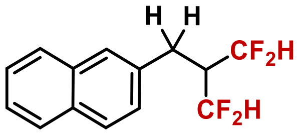

2-(2-(Difluoromethyl)-3,3-difluoropropyl)naphthalene (**16e**). Method D. Purified using preparative thin layer chromatography to give **16e** as a white solid; ^{1}H NMR (400 MHz, $CDCl_3$) δ 7.84 – 7.79 (m, 3H), 7.69 (s, 1H), 7.52 – 7.45 (m, 2H), 7.35 (dd, *J* = 8.5, 1.8 Hz, 1H), 6.09 – 5.79 (m, 2H), 3.14 (d, *J* = 7.2 Hz, 2H), 2.80 – 2.65 (m, 1H); ^{13}C NMR (100 MHz, $CDCl_3$) δ 134.4, 133.7, 132.5, 128.8, 127.9, 127.8, 127.7, 127.1, 126.6, 126.1, 114.7 (tt, *J* = 242.1, 6.2 Hz), 48.7 (p, *J* = 19.5 Hz), 28.3 (p, *J* = 4.2 Hz); ^{19}F NMR (376 MHz, $CDCl_3$) δ -123.7 – -123.9 (m); IR (Film): 3057, 2927, 2855, 1738, 1601, 1510, 1377, 1270, 1192, 1065, 1027, 961, 937, 898, 861, 817, 749, 650, 479 cm^{-1}; HRMS (EI) exact mass calculated for [M^+, $C_{14}H_{12}F_4^+$]: 256.0875, found 256.0898.

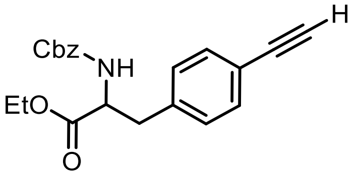

Ethyl 2-(((benzyloxy)carbonyl)amino)-3-(4-ethynylphenyl)propanoate. To a round bottom flask containing ethyl (R)-2-(((benzyloxy)carbonyl)amino)-3-(4-(((trifluoromethyl)sulfonyl)oxy)phenyl)propanoate (812 mg, 1.7 mmol), $Pd(PPh_3)_4$ (196 mg, 0.17 mmol), CuI (32 mg, 0.17 mmol), and TEA (17 mL) were added ethynyltrimethylsilane (353 uL, 2.6 mmol). The reaction temperature was increased to 80 °C, and the resulting mixture was stirred for 12 h. The mixture was poured into water and extracted with dichloromethane. The organic phase was dried with Mg_2SO_4, filtered, and concentrated under reduced pressure. The crude mixture was diluted in THF (17 mL), and treated with 1 M TBAF in THF (5.1 mL, 5.1 mmol) at 0 °C. The reaction temperature was increased to 26 °C, and the resulting mixture was stirred for 30 min. The mixture was poured into water and extracted with ethyl acetate. The organic phase was dried with Mg_2SO_4, filtered, and concentrated under reduced pressure. The crude mixture was purified by a reverse-phase C18 column chromatography (acetonitrile/H_2O = 2/1) to afford desired product as a colorless oil (312 mg, 52%); ^1H NMR (400 MHz, $CDCl_3$) δ 7.40 – 7.32 (m, 7H), 7.07 (d, J = 7.9 Hz, 2H), 5.24 (d, J = 8.1 Hz, 1H), 5.13 – 5.06 (m, 2H), 4.63 (q, J = 6.4 Hz, 1H), 4.16 (q, J = 7.2 Hz, 2H), 3.17 – 3.04 (m, 3H), 1.23 (t, J = 7.2 Hz, 3H); ^{13}C NMR (125 MHz, $CDCl_3$) δ 171.4, 155.7, 136.9, 136.4, 132.4, 129.5, 128.7, 128.4, 128.2, 121.0, 83.5, 77.4, 67.1, 61.8, 54.8, 38.4, 14.2; IR (neat): 3290, 3032, 2981, 2939, 1722, 1509, 1451, 1374, 1343, 1255, 1211, 1056, 1026, 852, 824, 740, 698, 615, 549 cm^{-1}; HRMS (EI) exact mass calculated for [M^+, $C_{21}H_{21}NO_4^+$]: 351.1471, found 351.1453.

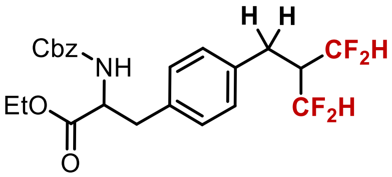

Ethyl 2-(((Benzyloxy)carbonyl)amino)-3-(4-(2-(difluoromethyl)-3,3-difluoropropyl)phenyl)propanoate (**16f**). Method D. Purified using preparative thin layer chromatography to give **16f** as a white solid; ^1H NMR (400 MHz, $CDCl_3$) δ 7.37 – 7.32 (m, 5H), 7.12 (d, J = 7.9 Hz, 2H), 7.07 (d, J = 7.9 Hz, 2H), 6.03 – 5.73 (m, 2H), 5.23 (d, J = 8.3 Hz, 1H), 5.09 (s, 2H), 4.62 (q, J = 6.5 Hz, 1H), 4.17 (dd, J = 7.0, 1.9 Hz, 2H), 3.09 (qd, J = 13.9, 5.9 Hz, 2H), 2.92 (d, J = 7.2 Hz, 2H), 2.64 – 2.51 (m, 1H), 1.23 (t, J = 7.4 Hz, 3H); ^{13}C NMR (100 MHz, $CDCl_3$) δ 171.4, 155.6, 136.2, 135.7, 134.6, 129.9, 129.2, 128.6, 128.3, 128.1, 114.5 (tt, J = 243.6, 6.2 Hz), 67.0, 61.6, 54.8, 48.6 (p, J = 19.5 Hz), 37.9, 27.5 (p, J = 4.1 Hz), 14.1; ^{19}F NMR (376 MHz, $CDCl_3$) δ -123.9 (dd, J = 56.0, 14.8 Hz); IR (Film): 3434, 3345, 3032, 2985, 2935, 1723, 1590, 1514, 1452, 1375, 1343, 1257, 1210, 1140, 1061, 1027, 931, 886, 858, 776, 742, 699, 652, 581, 566, 528, 496 cm^{-1}; HRMS (FAB) exact mass calculated for [$M+H^+$, $C_{23}H_{26}F_4NO_4^+$]: 456.1798, found 456.1779.

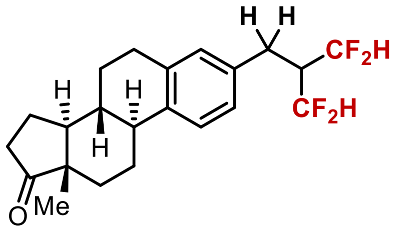

(*8R,9S,13S,14S*)-3-(2-(Difluoromethyl)-3,3-difluoropropyl)-13-methyl-6,7,8,9,11,12,13,14,15,16-decahydro-17H-cyclopenta[a]phenanthren-17-one (**16g**). Method D. The reaction was performed in DMF instead of MeCN. Purified using preparative thin layer chromatography to give **16g** as a white solid; m.p. 110 °C; ^{1}H NMR (400 MHz, $CDCl_3$) δ 7.25 (d, *J* = 9.1 Hz, 1H), 7.01 (d, *J* = 8.0 Hz, 1H), 6.96 (s, 1H), 6.05 – 5.77 (m, 2H), 2.90 (d, *J* = 7.5 Hz, 4H), 2.67 – 2.40 (m, 3H), 2.32 – 2.26 (m, 1H), 2.20 – 1.96 (m, 4H), 1.69 – 1.40 (m, 6H), 0.92 (s, 3H); ^{13}C NMR (100 MHz, $CDCl_3$) δ 221.0, 138.6, 137.1, 134.3, 129.6, 126.4, 125.9, 114.6 (tt, *J* = 243.6, 6.1 Hz), 50.5, 48.6 (p, *J* = 19.6 Hz), 48.0, 44.3, 38.1, 35.9, 31.6, 29.3, 27.4 (p, *J* = 4.0 Hz), 26.5, 25.7, 21.6, 13.9; ^{19}F NMR (376 MHz, $CDCl_3$) δ -123.8 – -124.1 (m); IR (Film): 2947, 2930, 2878, 2835, 1731, 1612, 1500, 1470, 1454, 1418, 1404, 1383, 1359, 1339, 1325, 1286, 1258, 1227, 1216, 1196, 1148, 1127, 1102, 1056, 1005, 970, 917, 906, 890, 838, 825, 782, 755, 721, 621, 574, 561, 503, 444, 413 cm^{-1}; HRMS (EI) exact mass calculated for [M^+, $C_{22}H_{26}F_4O^+$]: 382.1920, found 382.1932.

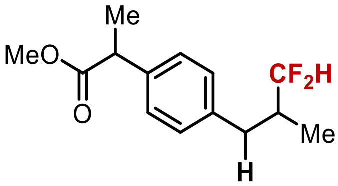

Methyl 2-(4-(-3,3-difluoro-2-methylpropyl)phenyl)propanoate (21). Method A. Purified using silica gel chromatography to give 40% yield of **21** as a colorless oil; ^{1}H NMR (400 MHz, $CDCl_3$)δ 7.23 (d, *J* = 8.2 Hz, 2H), 7.13 (d, *J* = 8.2 Hz, 2H), 5.64 (td, *J* = 56.8, 3.5 Hz, 1H), 3.71 (q, *J* = 7.2 Hz, 1H), 3.66 (s, 3H), 2.86 (dd, *J* = 13.7, 5.7 Hz, 1H), 2.46 (dd, *J* = 13.7, 9.0 Hz, 1H), 2.25 – 2.09 (m, 1H), 1.49 (d, *J* = 7.2 Hz, 3H), 0.97 (d, *J* = 6.9 Hz, 3H); ^{13}C NMR (100 MHz, $CDCl_3$) δ 175.1, 138.6, 137.6, 129.4, 127.6, 118.5 (t, *J* = 242.4 Hz), 52.1, 45.0, 39.2 (t, *J* = 20.2 Hz), 35.8 (dd, *J* = 6.0, 3.8 Hz), 18.6, 12.0 (t, *J* = 10.1 Hz); ^{19}F NMR (376 MHz, $CDCl_3$) δ -123.49 (ddd, J = 276.9, 56.6, 12.4 Hz), -126.52 (ddd, J = 277.1, 56.9, 17.7 Hz); IR (neat): 2981, 2951, 2887, 1737, 1583, 1344, 1063, 859, 547 cm^{-1}; HRMS (EI) exact mass calculated for [$C_{14}H_{16}F_2O_2^+$]: 256.1275, found 256.1283.

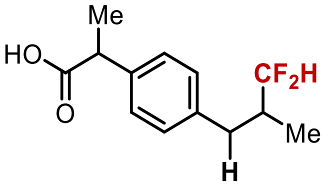

2-(4-(3,3-Difluoro-2-methylpropyl)phenyl)propanoic acid (22). To a solution of **21** (15 mg, 0.06 mmol) in THF/MeOH/H_2O (2 mL, 1/1/1) was added LiOH·H_2O (7 mg, 0.2 mmol) at room temperature, and the resulting mixture was stirred for 12 h. The mixture was poured into water and extracted with ethyl acetate. The organic phase was dried with Mg_2SO_4, filtered, and concentrated under reduced pressure. The residue was purified using silica gel chromatography to give **22** as a colorless oil (14 mg, 99%); ^{1}H NMR (400 MHz, $CDCl_3$) δ 7.26 (d, *J* = 8.0 Hz, 2H), 7.14 (d, *J* = 8.0 Hz, 2H), 5.64 (td, *J* = 56.8, 3.5 Hz, 1H), 3.72 (q, *J* = 7.2 Hz, 1H), 2.86 (dd, *J* = 13.7, 5.7 Hz, 1H), 2.46 (dd, *J* = 13.7, 9.1 Hz, 1H), 2.23 – 2.09 (m, 1H), 1.51 (d, *J* = 7.2 Hz, 3H), 0.97 (*d*, J = 6.9 Hz, 3H); ^{13}C NMR (100 MHz, $CDCl_3$) δ 180.4, 138.0, 137.8, 129.5, 127.8, 118.5 (t, *J* = 242.4 Hz), 44.9, 39.2 (t, *J* = 20.2 Hz), 35.8 (dd, *J* = 6.0, 3.8 Hz), 18.1, 12.0 (t, *J* = 10.1 Hz); ^{19}F NMR (376 MHz, $CDCl_3$) δ -123.51 (ddd, J = 276.9, 56.6, 12.6 Hz), -126.42 (ddd, J = 277.1, 56.9, 17.6 Hz); IR (neat): 2981, 2951, 2887, 1737, 1583, 1344, 1063, 859, 547 cm^{-1}; HRMS (EI) exact mass calculated for [$C_{14}H_{16}F_2O_2^+$]: 256.1275, found 256.1283.

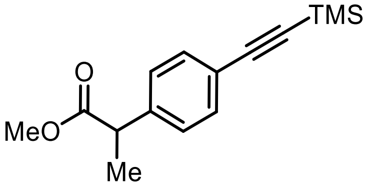

Methyl 2-(4-((trimethylsilyl)ethynyl)phenyl)propanoate. To an Ar-filled round bottom flask containing methyl 2-(4-bromophenyl)propanoate (168 mg, 0.69 mmol), Pd(PPh_3)$_2$$Cl_2$ (48 mg, 0.07 mmol), CuI (13 mg, 0.07 mmol), and TEA (6.9 mL) were added ethynyltrimethylsilane (143 uL, 1.7 mmol). The reaction temperature was increased to 80 ^{o}C, and the resulting mixture was stirred for 24 h. The mixture was poured into water and extracted with ethyl acetate. The organic phase was dried with Mg_2SO_4, filtered, and concentrated under reduced pressure. The crude mixture was purified by a silica gel chromatography (hexane/ethyl acetate = 25/1) to give 88% yield of product as a colorless oil; ^{1}H NMR (400 MHz, $CDCl_3$) δ 7.41 (d, *J* = 8.2 Hz, 2H), 7.22 (d, *J* = 8.2 Hz, 2H), 3.70 (q, *J* = 7.1 Hz, 1H), 3.65 (s, 3H), 1.48 (d, *J* = 7.2 Hz, 3H), 0.24 (s, 9H); ^{13}C NMR (100 MHz, $CDCl_3$) δ 174.7, 141.0, 132.3, 127.5, 122.1, 104.9, 94.4, 52.3, 45.4, 18.5, 0.1; IR (Film): 2958, 2158, 1740, 1505, 1456, 1435.51, 1412, 1377, 1334, 1251, 1208, 1166, 1116, 1069, 1018, 968, 864, 844, 761, 700, 640, 548 cm^{-1}; HRMS (EI) exact mass calculated for [M$^+$, $C_{15}H_{20}O_2Si^+$]: 260.1233, found 260.1231.

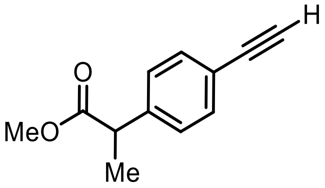

Methyl 2-(4-ethynylphenyl)propanoate (25). To a round bottom flask containing methyl 2-(4-((trimethylsilyl)ethynyl)phenyl)propanoate (158 mg, 0.6 mmol) in THF (6 mL) were added dropwise 1 M TBAF in THF (1.8 mL, 1.8 mmol) at 0 °C. The reaction temperature was increased to 26 °C, and the resulting mixture was stirred for 30 min. The mixture was poured into water and extracted with ethyl acetate. The organic phase was dried with Mg_2SO_4, filtered, and concentrated under reduced pressure. The crude mixture was purified by a silica gel chromatography (hexane/ethyl acetate = 25/1) to give 70% yield of (**25**) as a colorless oil; ^{1}H NMR (400 MHz, $CDCl_3$) δ 7.45 (d, *J* = 8.3 Hz, 2H), 7.26 (d, *J* = 8.3 Hz, 2H), 3.72 (q, *J* = 7.2 Hz, 1H), 3.66 (s, 3H), 3.06 (s, 1H), 1.49 (d, *J* = 7.4 Hz, 3H); ^{13}C NMR (100 MHz, $CDCl_3$) δ 174.7, 141.4, 132.5, 127.7, 121.1, 83.5, 77.4, 52.3, 45.4, 18.6; IR (Film): 3288, 3032, 2983, 2952, 2879, 2108, 1916, 1737, 1608, 1504, 1455, 1436, 1414, 1377, 1335, 1255, 1211, 1168, 1118, 1070, 1014, 967, 842, 782, 657, 558, 519, 438 cm^{-1}; HRMS (EI) exact mass calculated for [M^+, $C_{12}H_{12}O_2^+$]: 188.0837, found 188.0840.

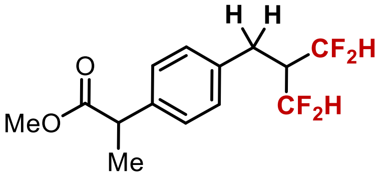

Methyl 2-(4-(2-(difluoromethyl)-3,3-difluoropropyl)phenyl)propanoate (26). Five reactions were performed in parallel according to Method D. The crude mixture was combined and purified using silica gel chromatography to give 18% average yield of **26** as a colorless oil; ^{1}H NMR (400 MHz, $CDCl_3$) δ 7.26 (d, *J* = 7.7 Hz, 2H), 7.18 (d, *J* = 7.7 Hz, 2H), 6.05 – 5.75 (m, 2H), 3.72 (q, *J* = 7.2 Hz, 1H), 3.67 (s, 3H), 2.94 (d, *J* = 7.2 Hz, 2H), 2.69 – 2.50 (m, 1H), 1.49 (d, *J* = 7.2 Hz, 3H); ^{13}C NMR (100 MHz, $CDCl_3$) δ 175.0, 139.5, 135.9, 129.5, 128.1, 114.6 (tt, *J* = 243.6, 6.1 Hz), 52.2, 48.7 (p, *J* = 19.6 Hz), 45.2, 27.7 (p, *J* = 4.1 Hz), 18.7; ^{19}F NMR (376 MHz, $CDCl_3$) δ -123.9 – -124.1 (m); IR (Film): 2986, 2954 1738, 1515, 1455, 1406, 1384, 1336, 1258, 1210, 1167, 1141, 1062, 1026, 969, 930, 859, 692, 497 cm^{-1}; HRMS (EI) exact mass calculated for [M^+, $C_{14}H_{16}F_4O_2^+$]: 292.1086, found 292.1100.

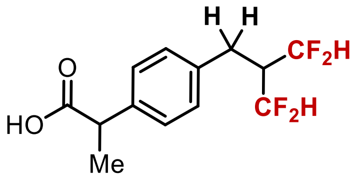

2-(4-(2-(Difluoromethyl)-3,3-difluoropropyl)phenyl) propanoic acid (27). Method D. To a round bottom flask containing **26** (56 mg, 0.2 mmol) and THF/H_2O (2 mL, 10/1) was added LiOH·H_2O (25 mg, 0.6 mmol). The resulting mixture was stirred for 12 h. The mixture was poured into 1 N HCl and extracted with ethyl acetate. The organic phase was dried with Mg_2SO_4, filtered, and concentrated under reduced pressure. The crude mixture was purified by a silica gel chromatography (DCM/MeOH = 25/1) to give 89% yield of **27** as a colorless oil**.** ^{1}H NMR (400 MHz, $CDCl_3$) δ 7.28 (d, *J* = 8.0 Hz, 2H), 7.19 (d, *J* = 7.8 Hz, 2H), 6.05 – 5.74 (m, 2H), 3.73 (q, *J* = 7.2 Hz, 1H), 2.94 (d, *J* = 7.1 Hz, 2H), 2.68 – 2.50 (m, 1H), 1.51 (d, *J* = 7.1 Hz, 3H); ^{13}C NMR (100 MHz, $CDCl_3$) δ 180.6, 138.7, 136.3, 129.5, 128.3, 114.6 (tt, *J* = 243.4, 6.1 Hz), 48.7 (p, *J* = 19.6 Hz), 45.1, 27.6 (p, *J* = 4.1 Hz), 18.2;^{19}F NMR (376 MHz, $CDCl_3$) δ -123.8 – -124.0 (m). IR (Film): 2988, 2941, 2726, 2636, 1709, 1515, 1457, 1409, 1387, 1270, 1233, 1189, 1141, 1061, 1027, 931, 863, 688, 661, 586, 556, 527, 499 cm^{-1}; HRMS (EI) exact mass calculated for [M^+, $C_{13}H_{14}F_4O_2^+$]: 278.0930, found 278.0924.

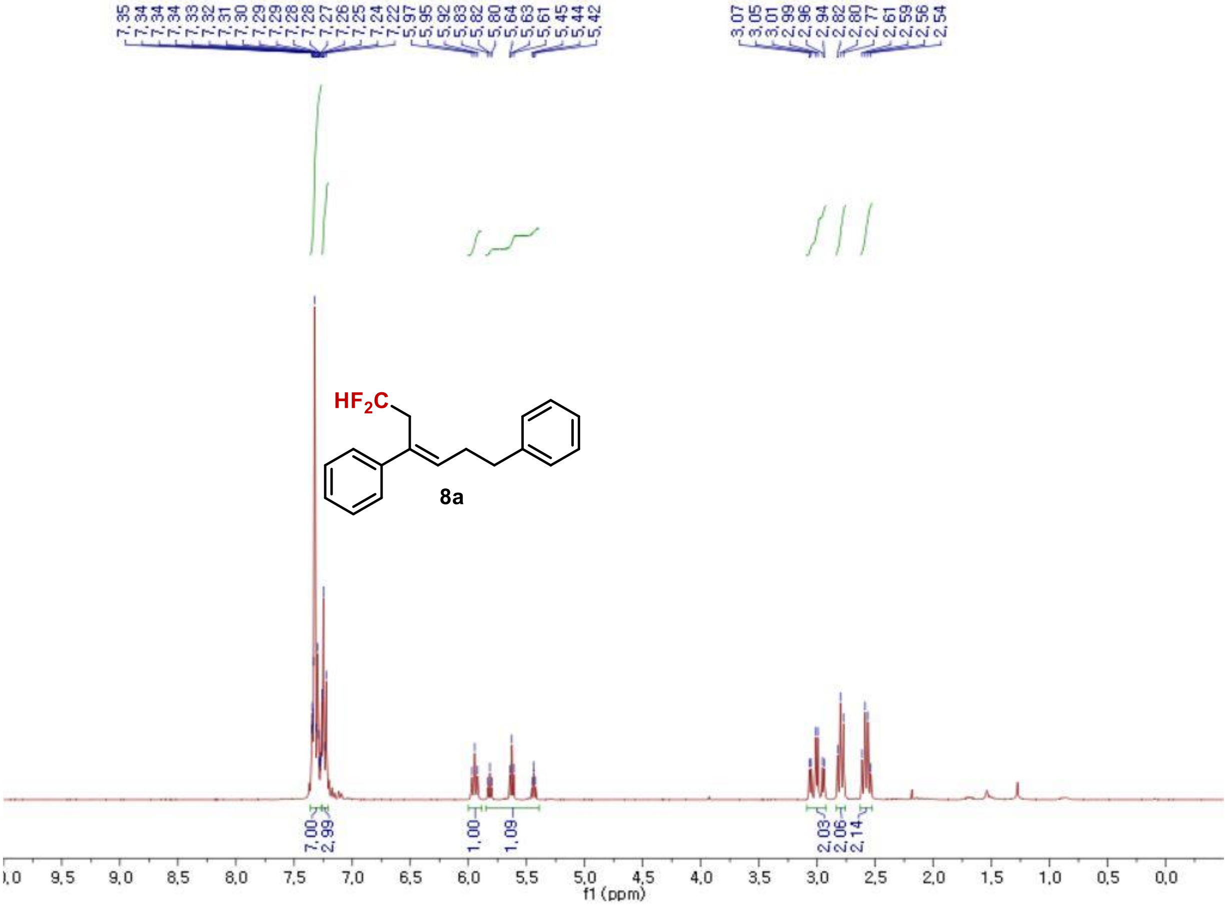

Supplementary Figure 8. ^{1}H NMR Spectrum of (6,6-Difluorohex-3-ene-1,4-diyl)dibenzene (**8a**)

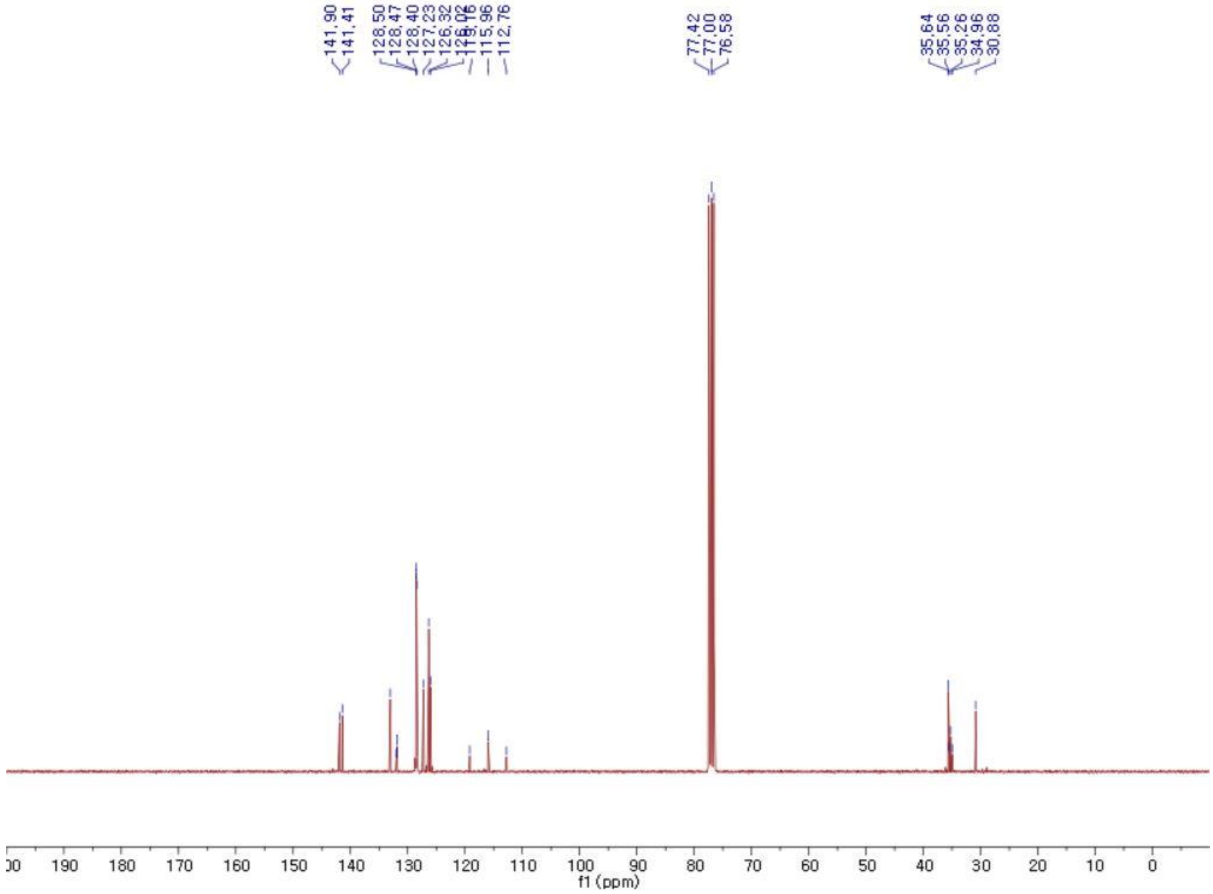

Supplementary Figure 9. ^{13}C NMR Spectrum of (6,6-Difluorohex-3-ene-1,4-diyl)dibenzene (**8a**)

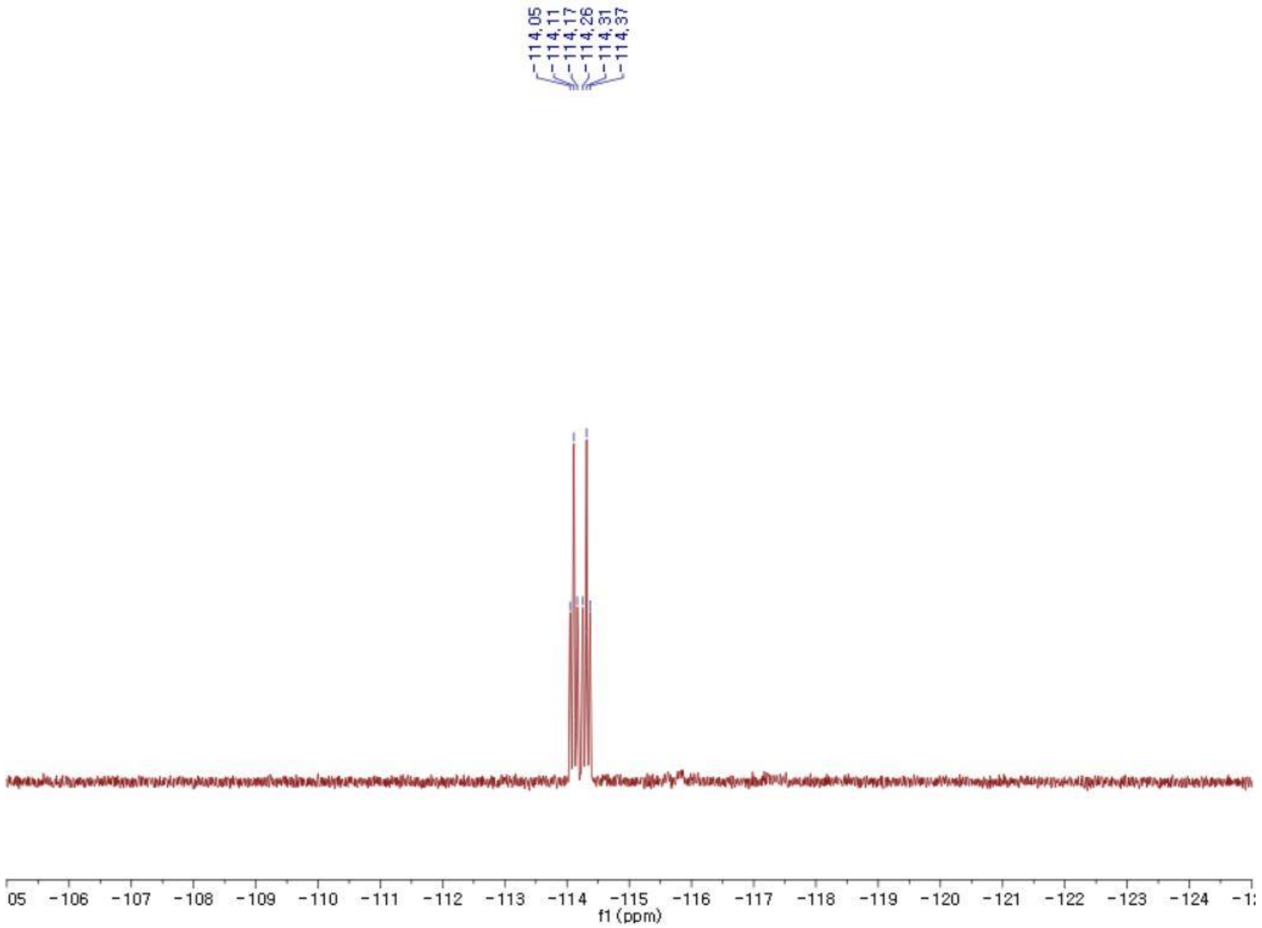

Supplementary Figure 10. ^{19}F NMR Spectrum of (6,6-Difluorohex-3-ene-1,4-diyl)dibenzene (**8a**)

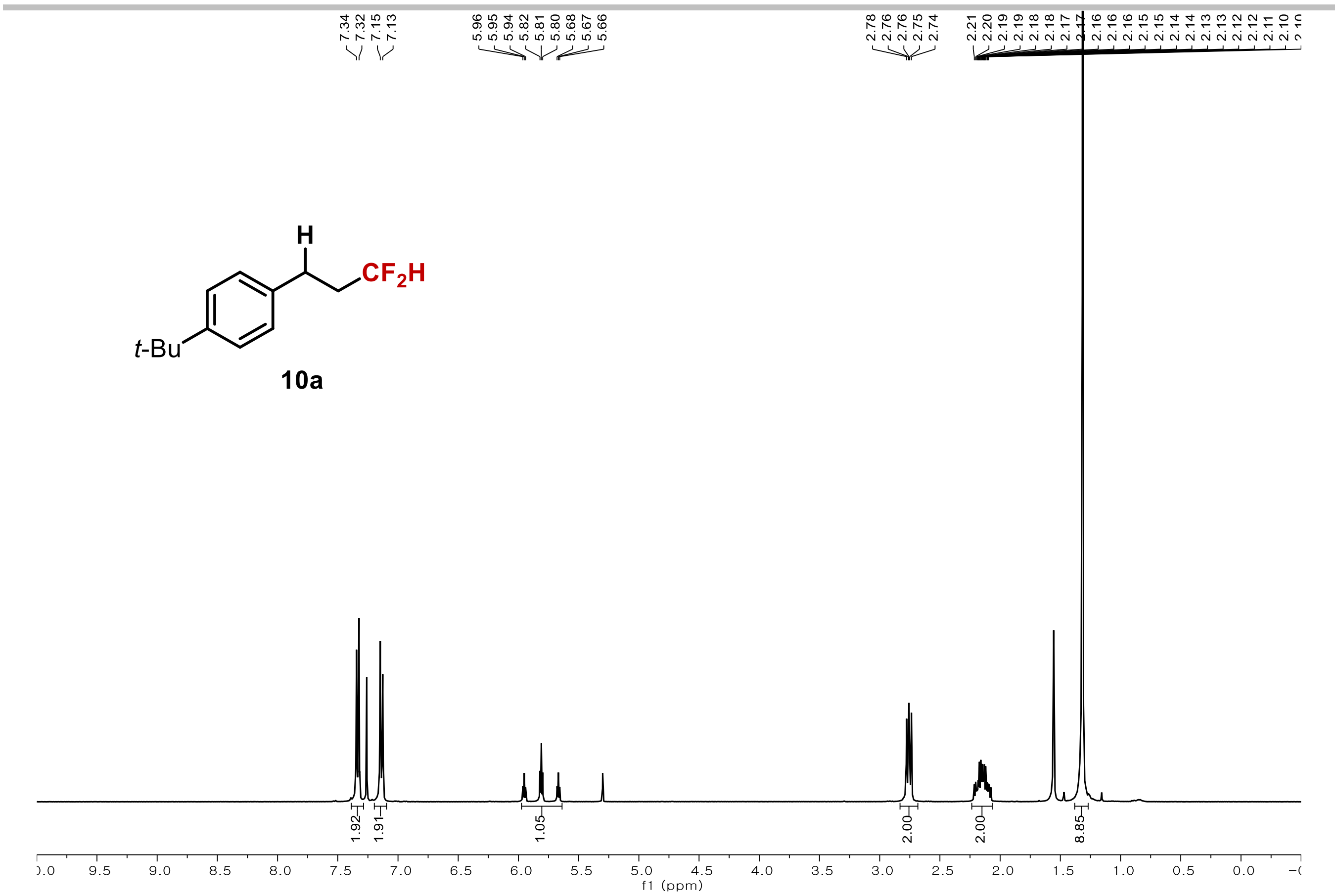

Supplementary Figure 11. ^{1}H NMR Spectrum of 1-(tert-Butyl)-4-(3,3-difluoropropyl)benzene (**10a**)

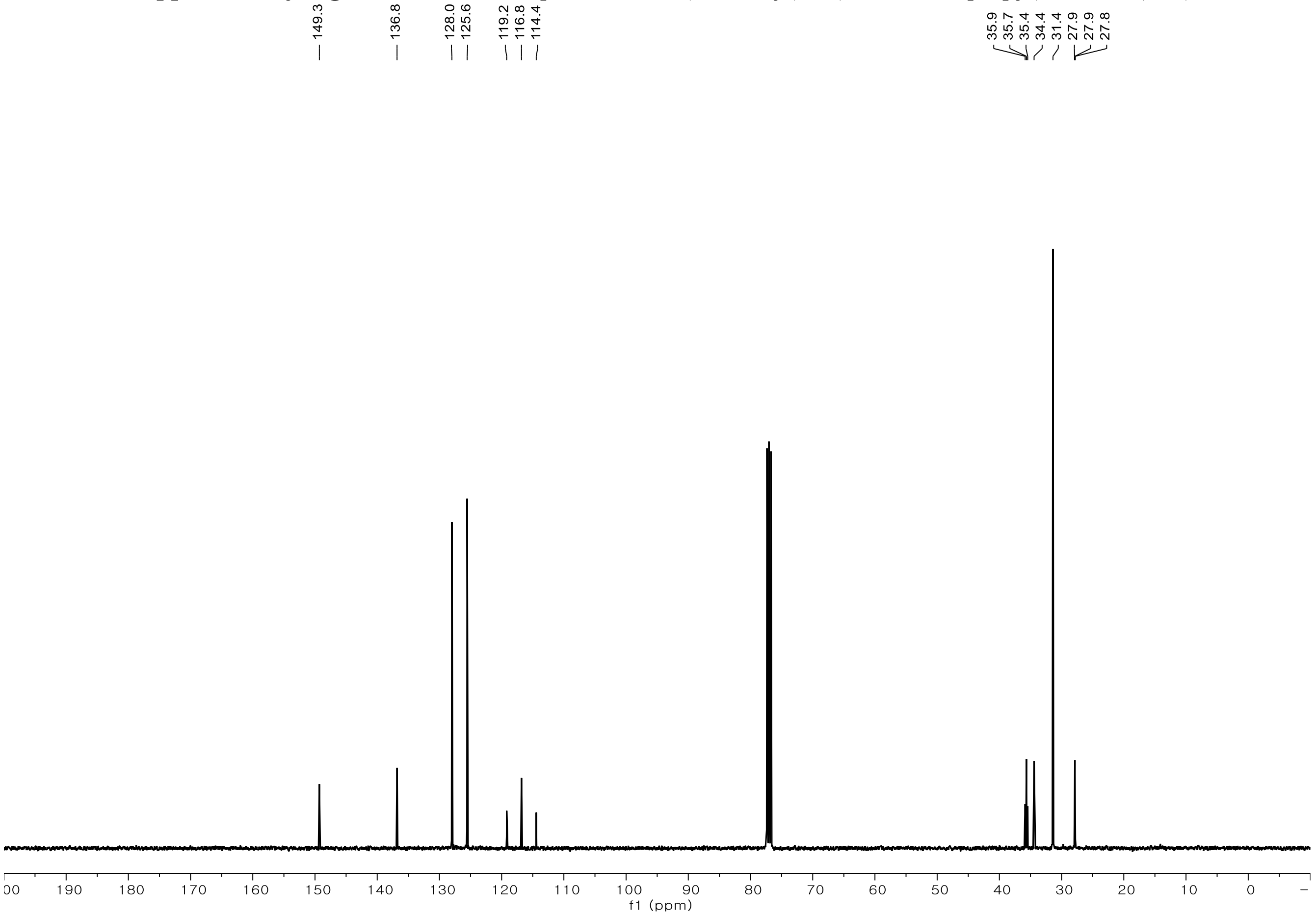

Supplementary Figure 12. ^{13}C NMR Spectrum of 1-(tert-Butyl)-4-(3,3-difluoropropyl)benzene (**10a**)

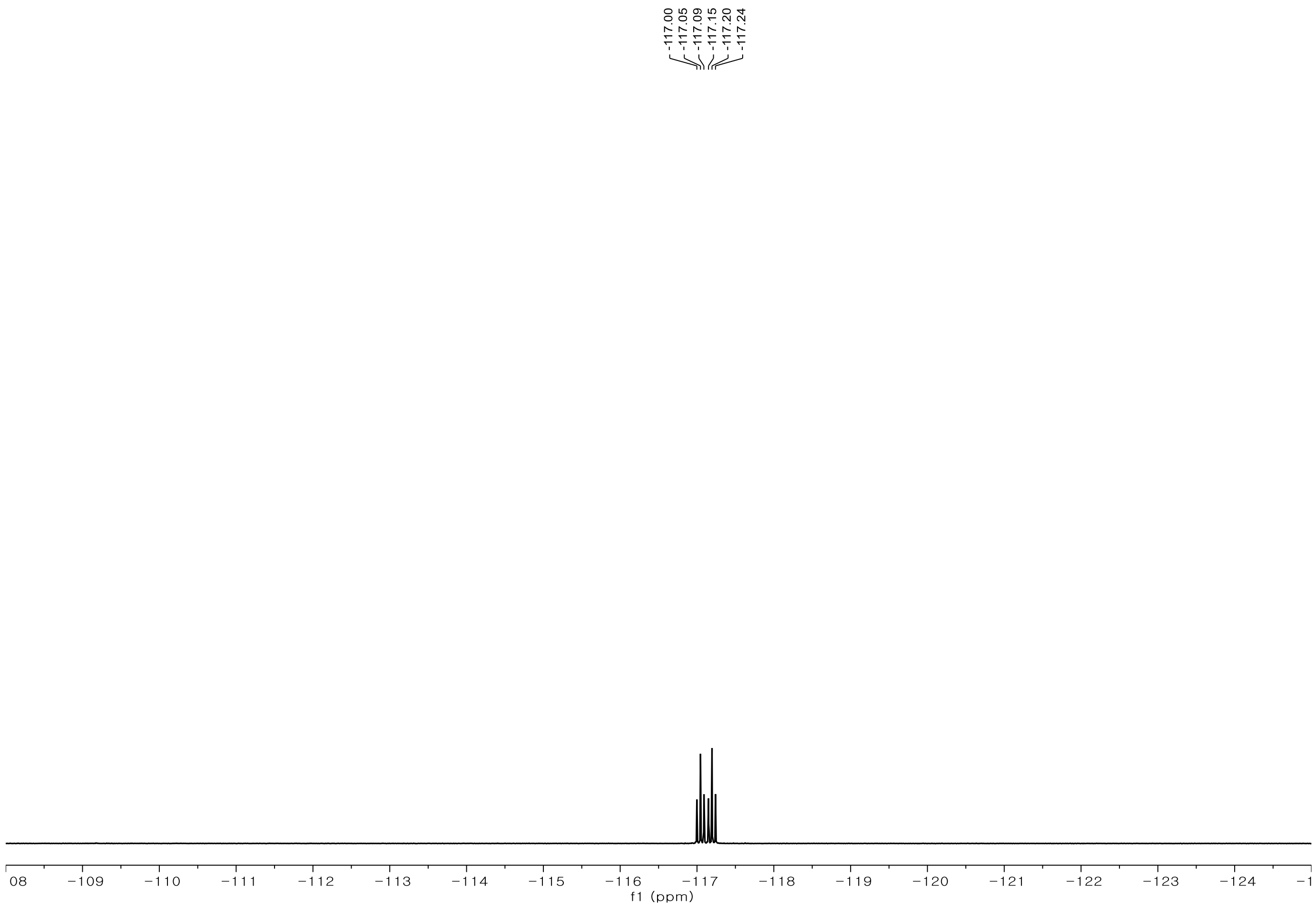

Supplementary Figure 13. ^{19}F NMR Spectrum of 1-(tert-Butyl)-4-(3,3-difluoropropyl)benzene (**10a**)

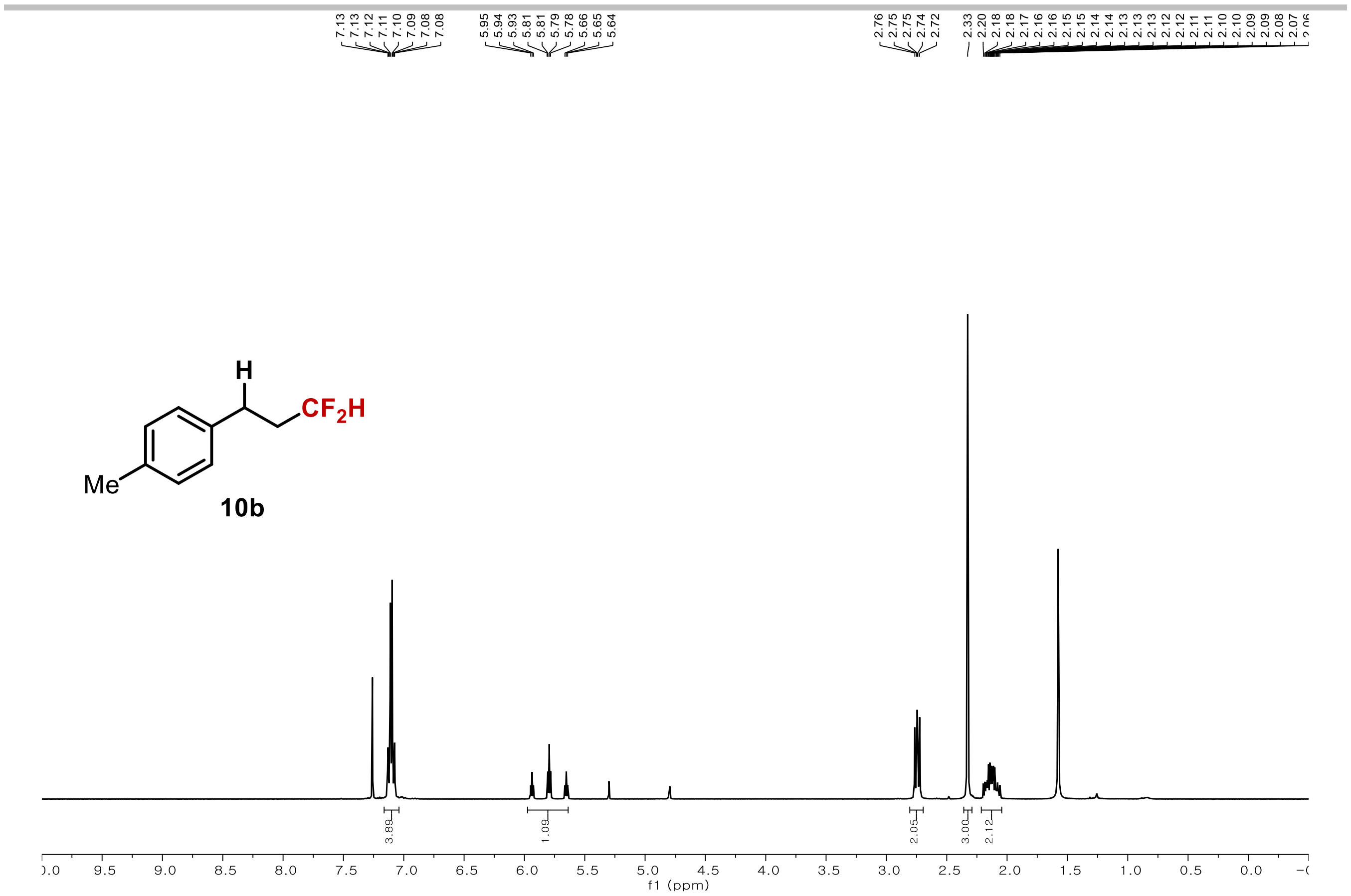

Supplementary Figure 14. ^{1}H NMR Spectrum of 1-(3,3-Difluoropropyl)-4-methylbenzene (**10b**)

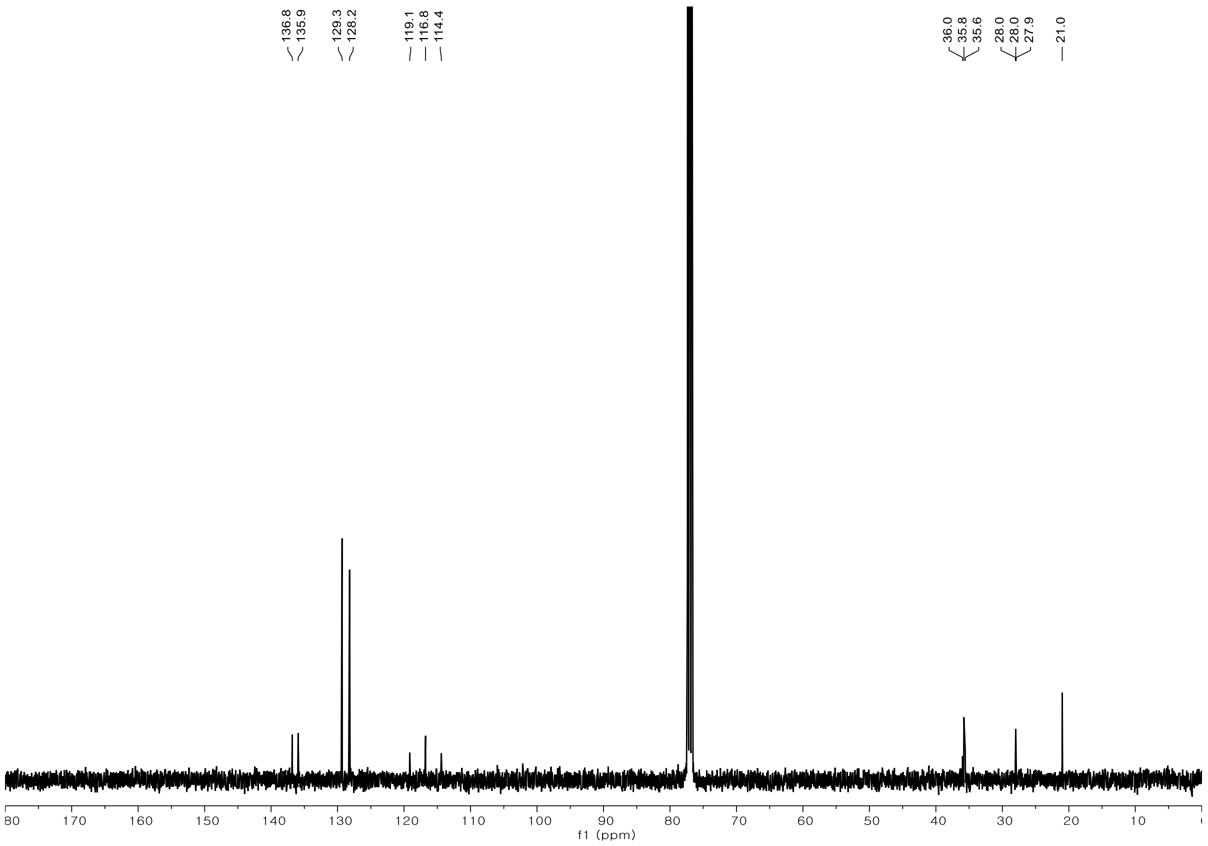

Supplementary Figure 15. ^{13}C NMR Spectrum of 1-(3,3-Difluoropropyl)-4-methylbenzene (**10b**)

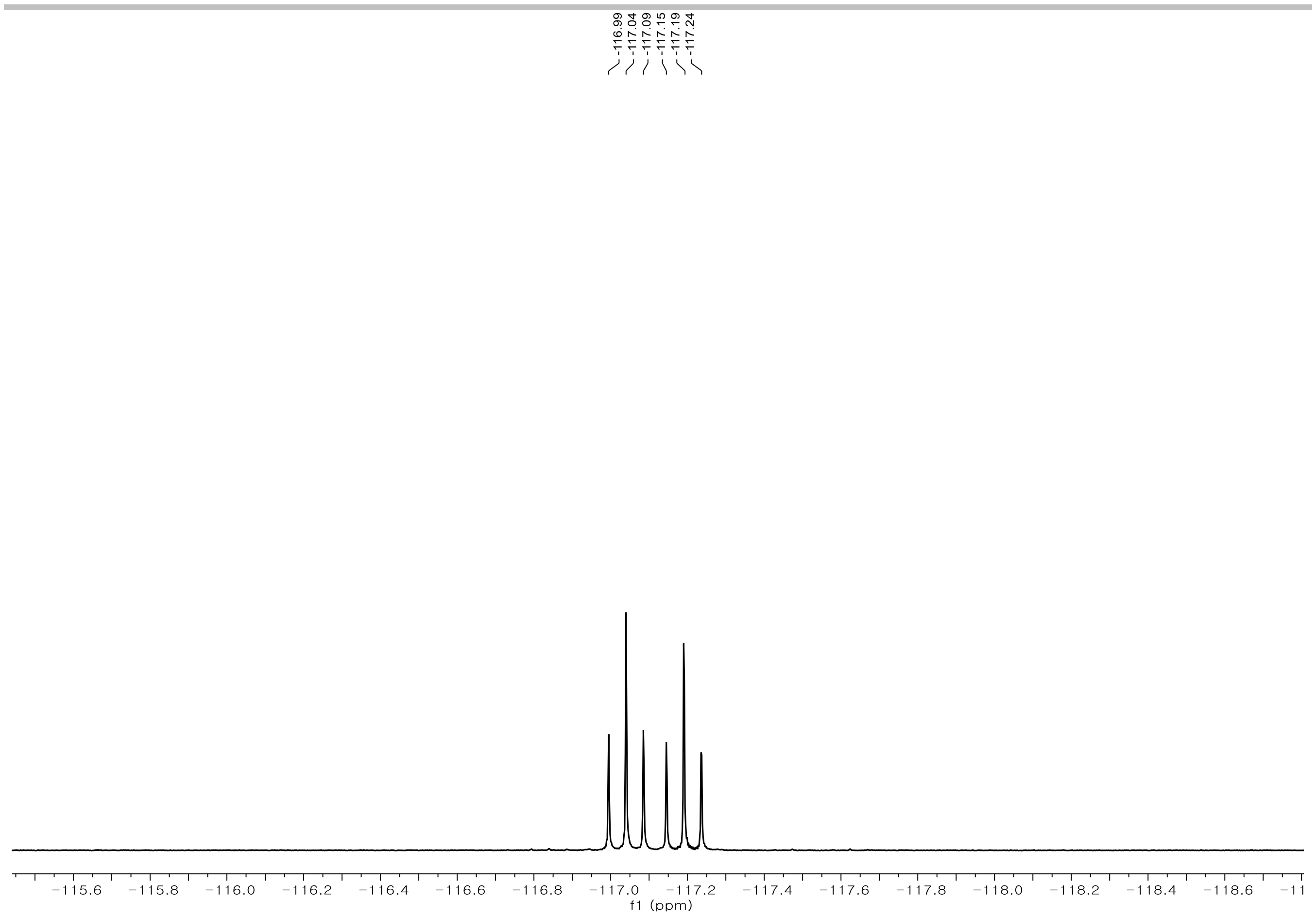

Supplementary Figure 16. ^{19}F NMR Spectrum of 1-(3,3-Difluoropropyl)-4-methylbenzene (**10b**)

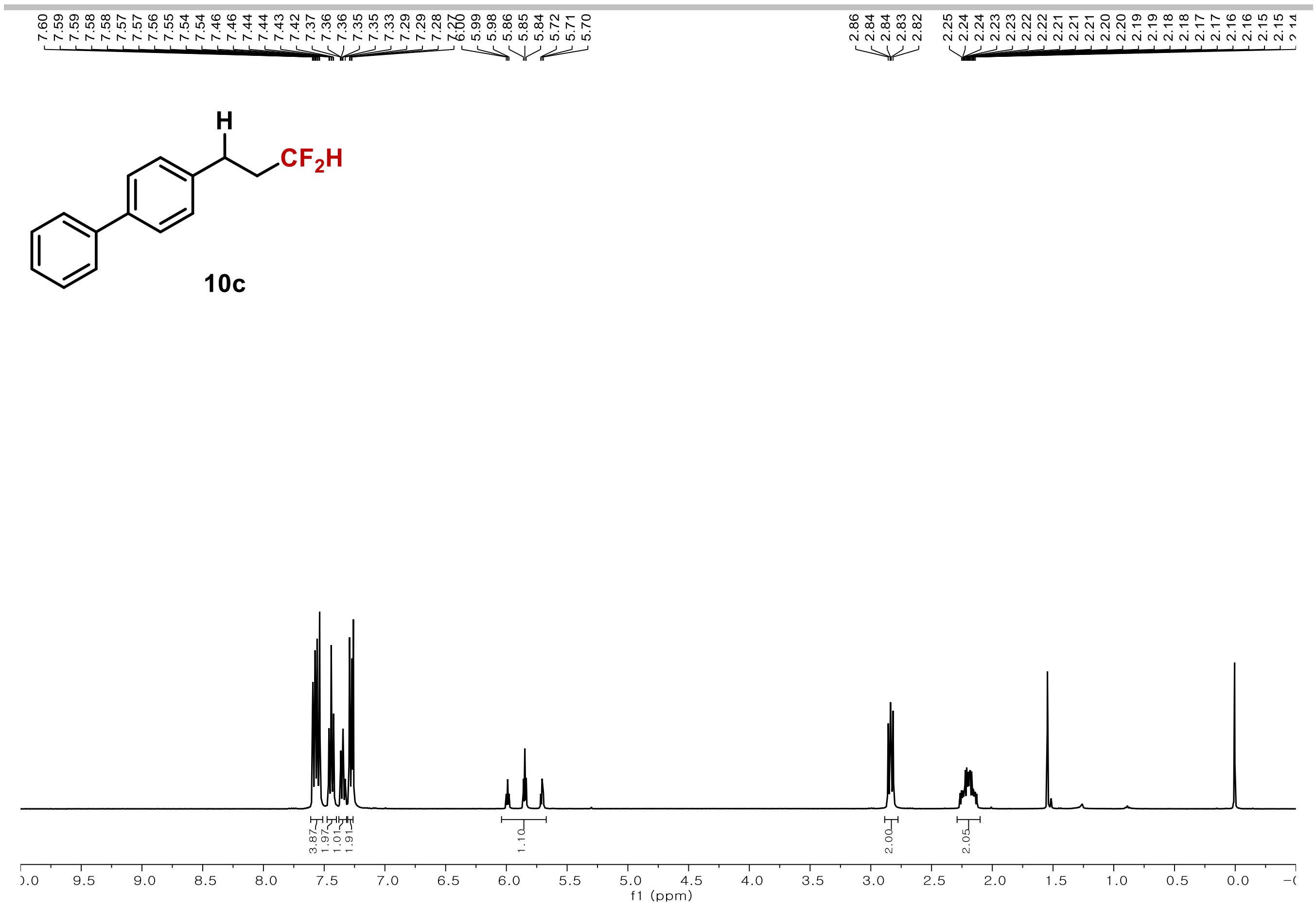

Supplementary Figure 17. ^{1}H NMR Spectrum of 4-(3,3-Difluoropropyl)-1,1'-biphenyl (**10c**)

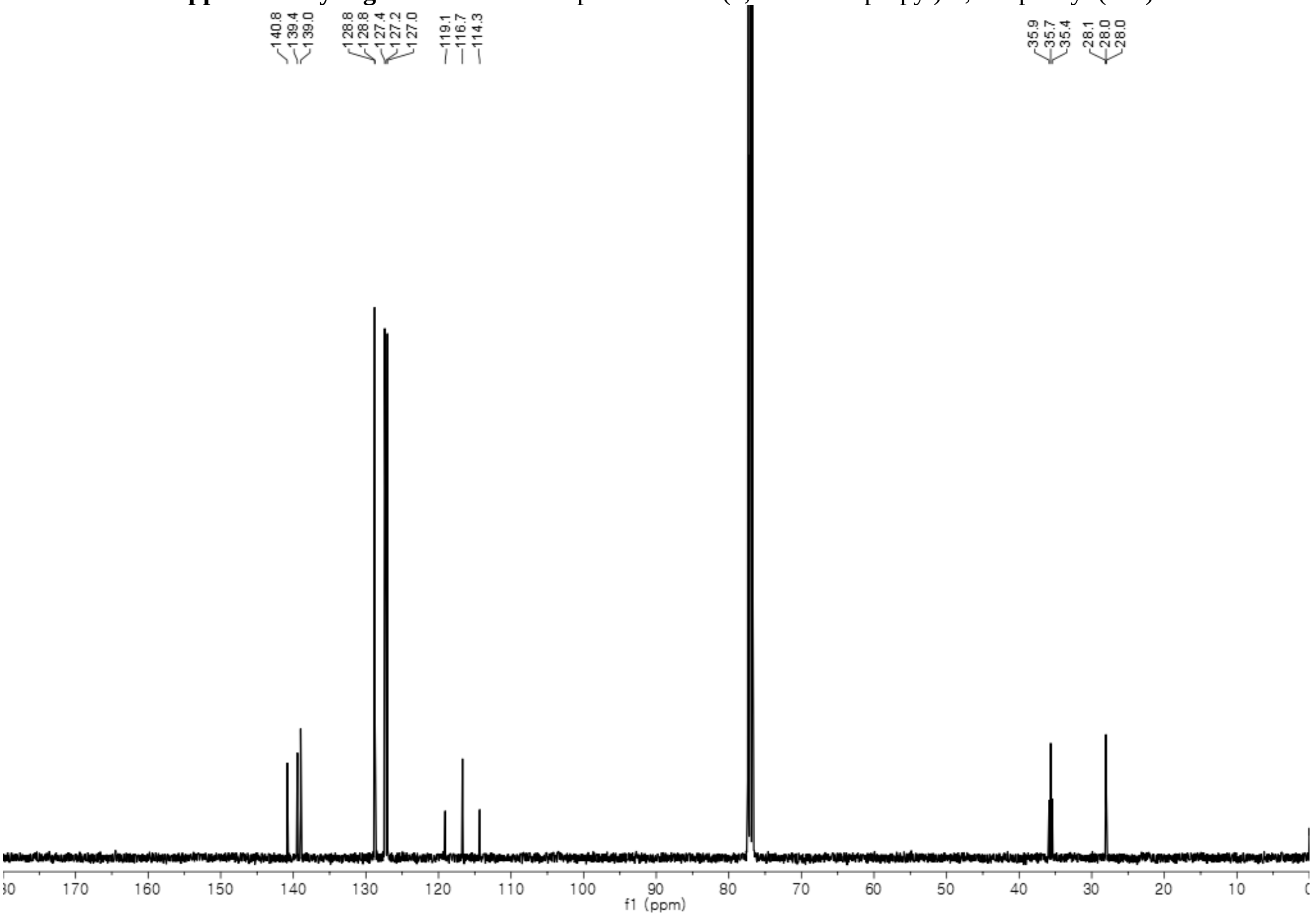

Supplementary Figure 18. ^{13}C NMR Spectrum of 4-(3,3-Difluoropropyl)-1,1'-biphenyl (**10c**)

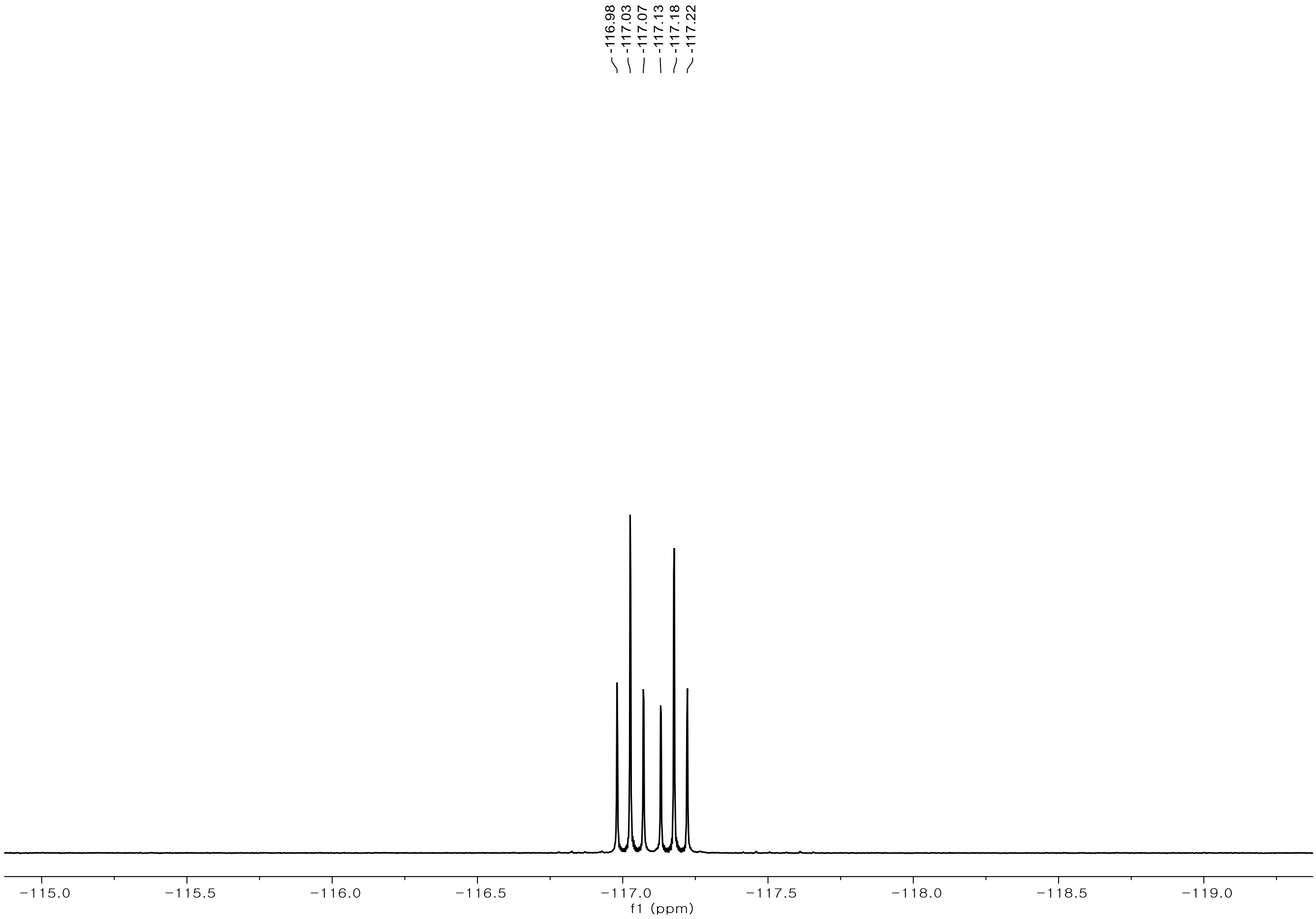

Supplementary Figure 19. ^{19}F NMR Spectrum of 4-(3,3-Difluoropropyl)-1,1'-biphenyl (**10c**)

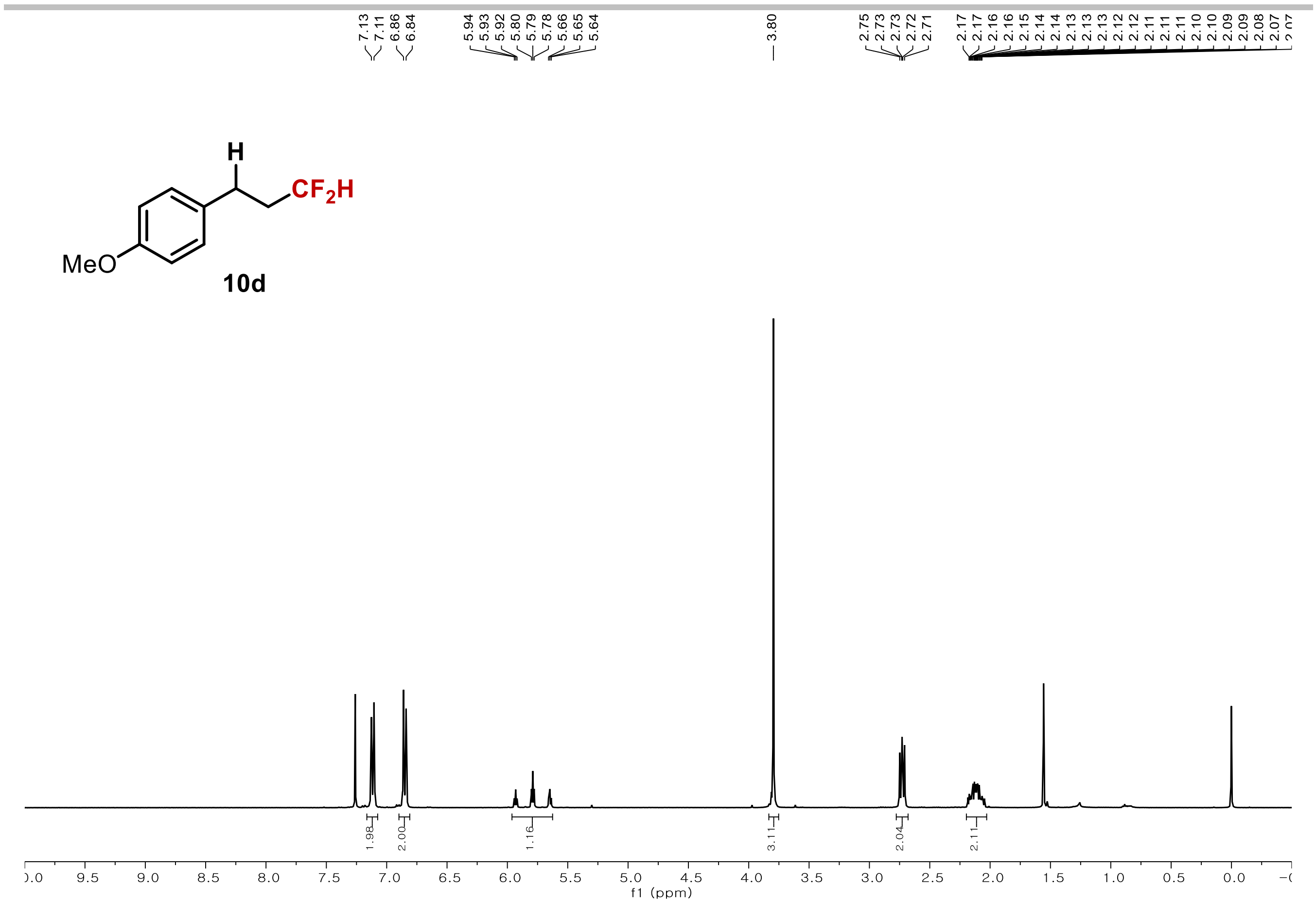

Supplementary Figure 20. ^{1}H NMR Spectrum of 1-(3,3-Difluoropropyl)-4-methoxybenzene (**10d**)

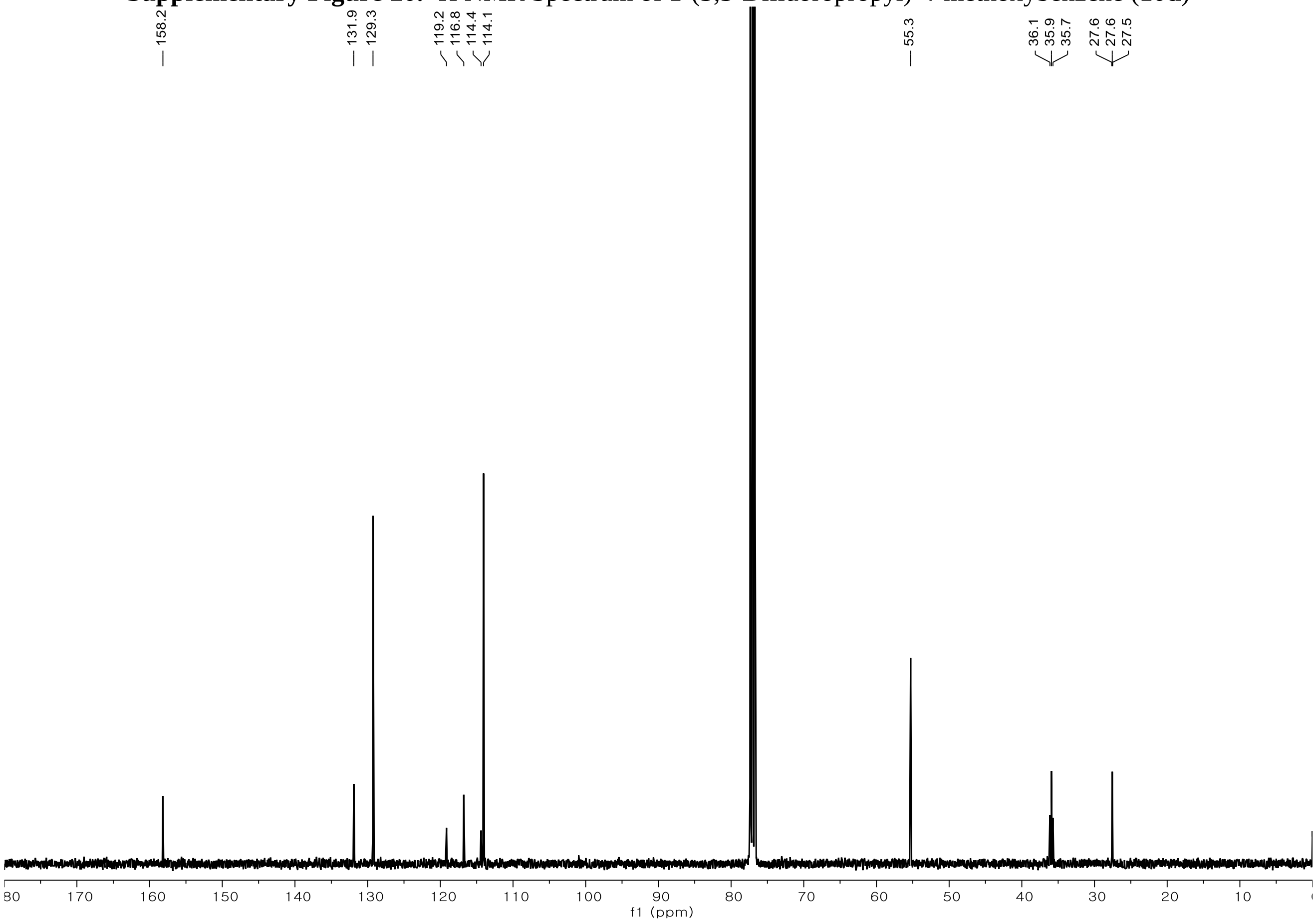

Supplementary Figure 21. ^{13}C NMR Spectrum of 1-(3,3-Difluoropropyl)-4-methoxybenzene (**10d**)

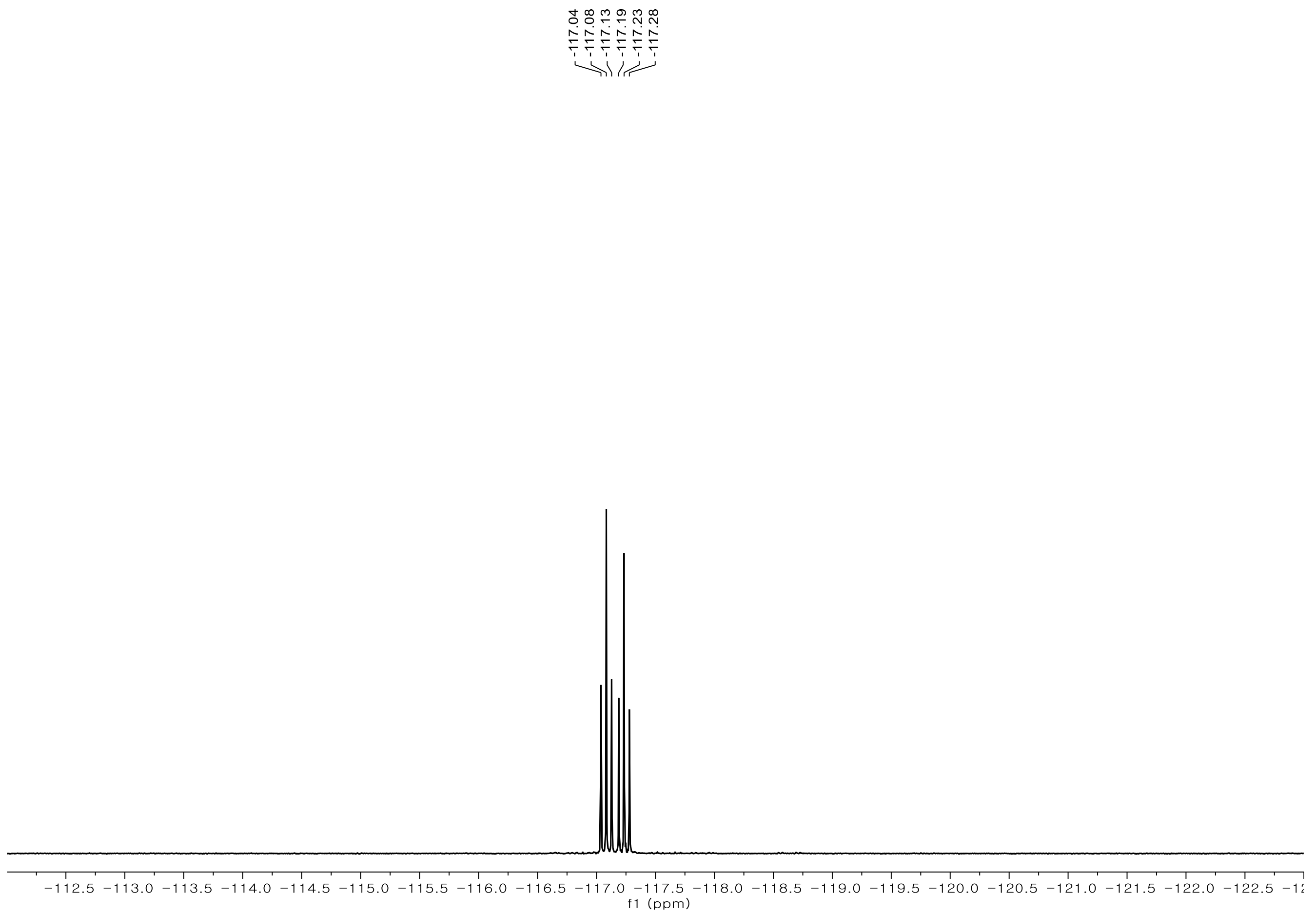

Supplementary Figure 22. ^{19}F NMR Spectrum of 1-(3,3-Difluoropropyl)-4-methoxybenzene (**10d)**

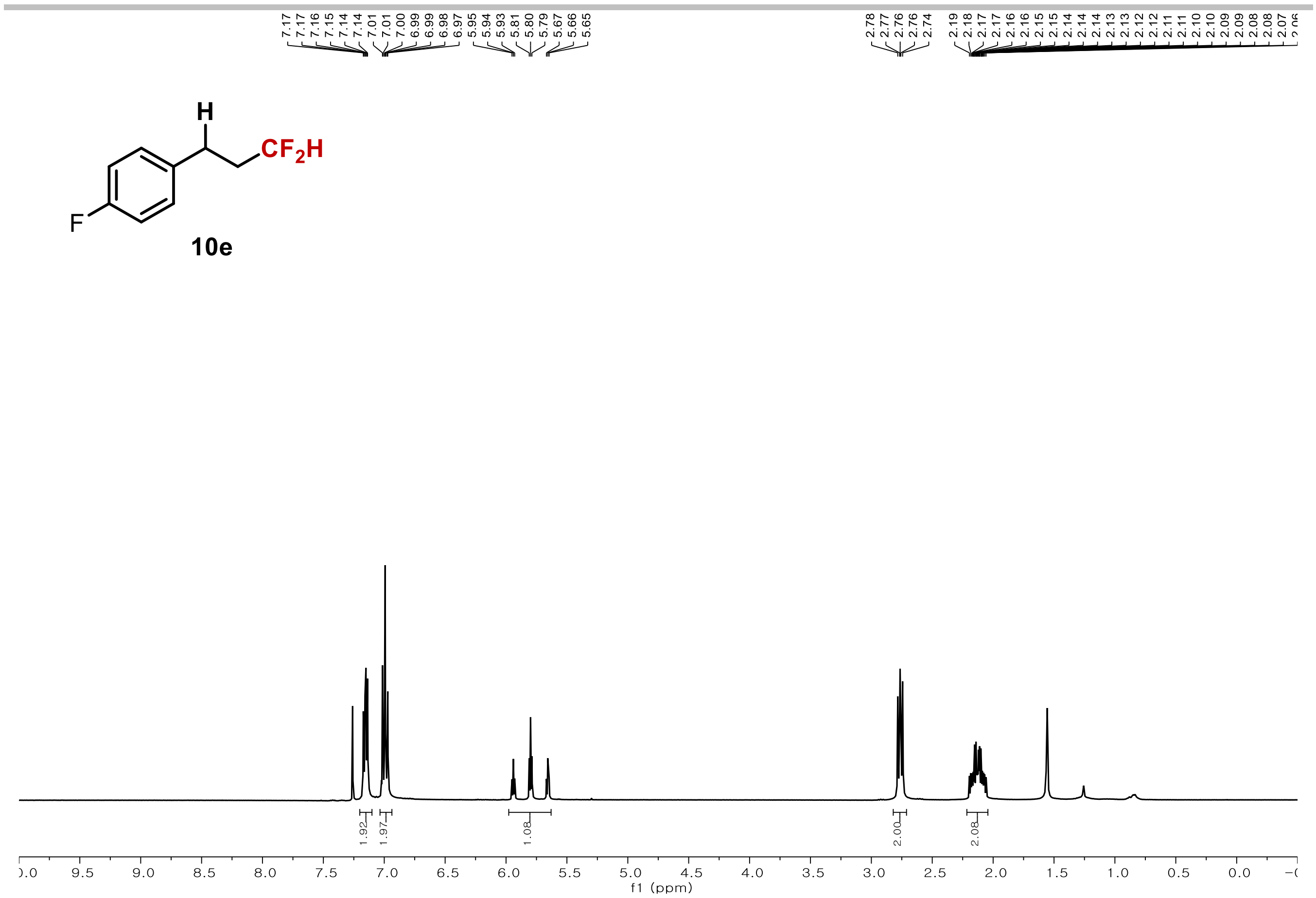

Supplementary Figure 23. ^{1}H NMR Spectrum of 1-(3,3-Difluoropropyl)-4-fluorobenzene (**10e**)

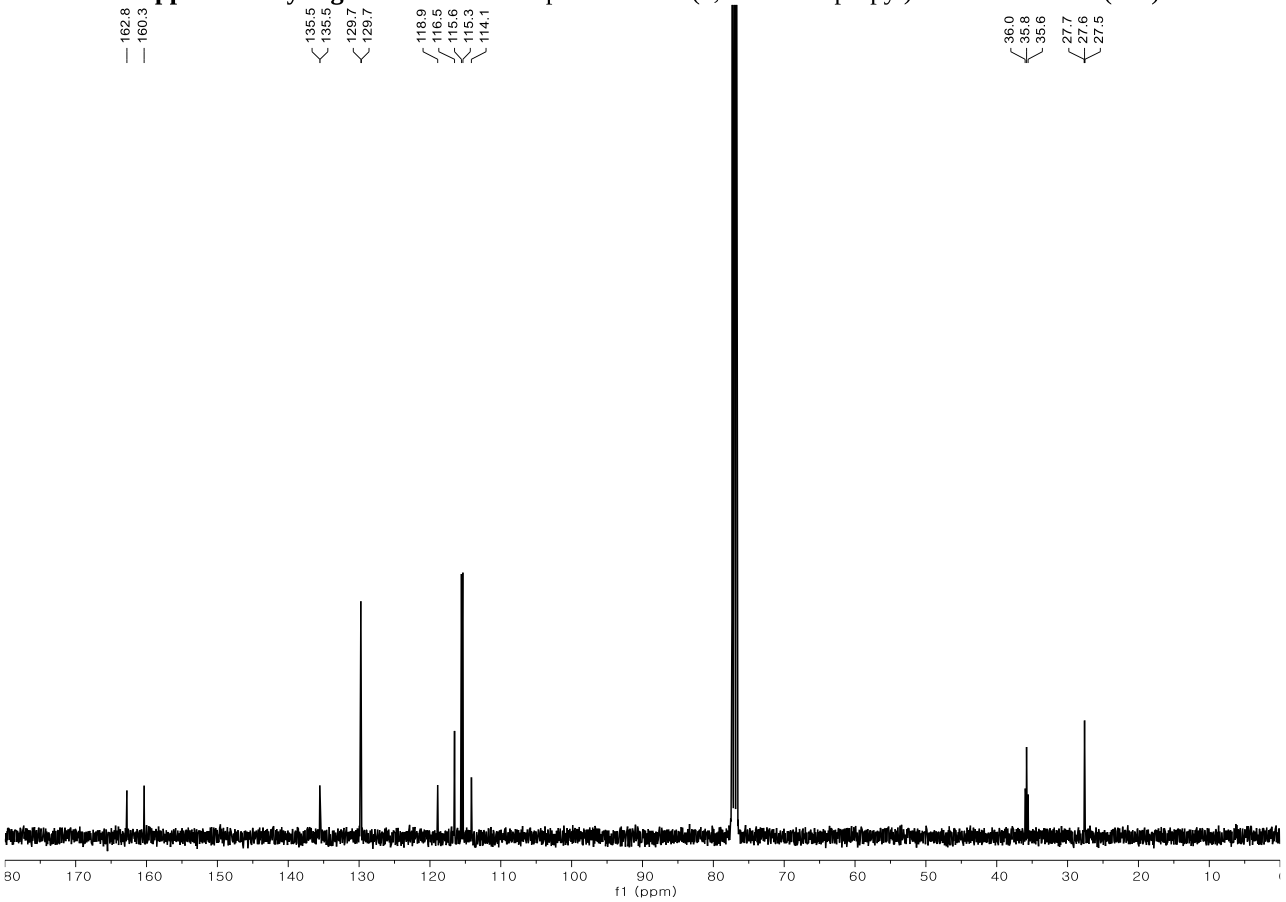

Supplementary Figure 24. ^{13}C NMR Spectrum of 1-(3,3-Difluoropropyl)-4-fluorobenzene (**10e**)

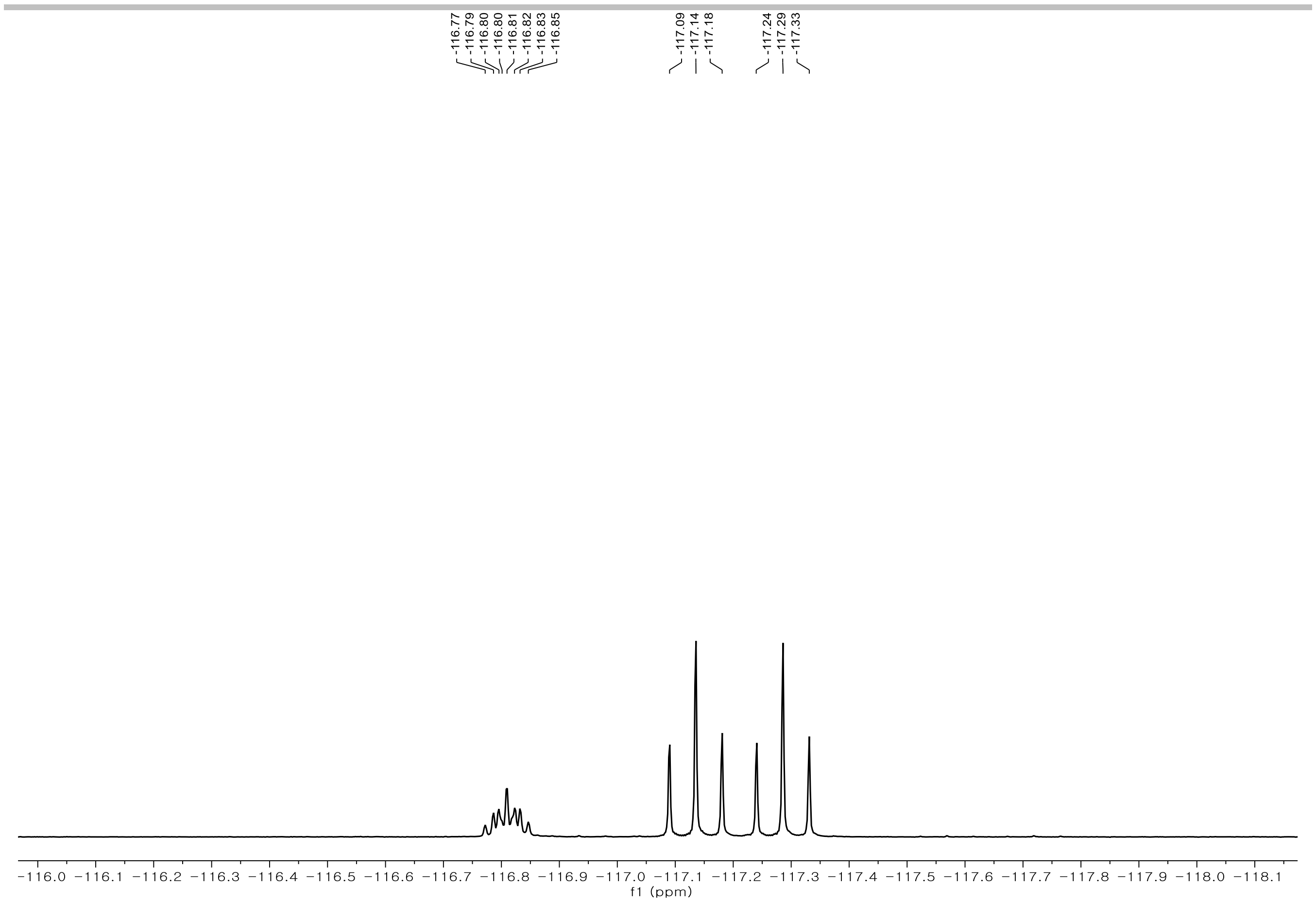

Supplementary Figure 25. ^{19}F NMR Spectrum of 1-(3,3-Difluoropropyl)-4-fluorobenzene (**10e**)

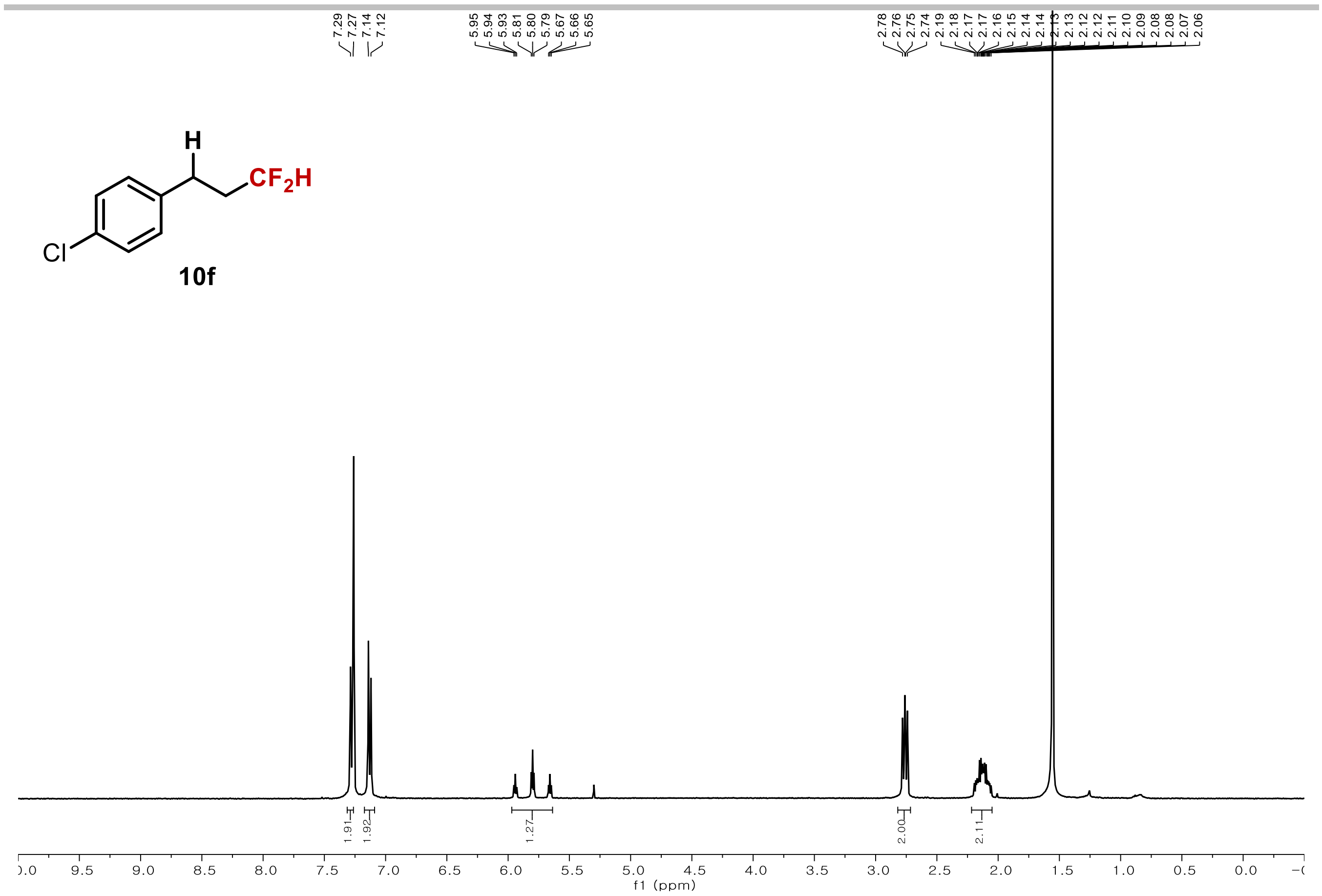

Supplementary Figure 26. ^{1}H NMR Spectrum of 1-Chloro-4-(3,3-difluoropropyl)benzene (**10f**)

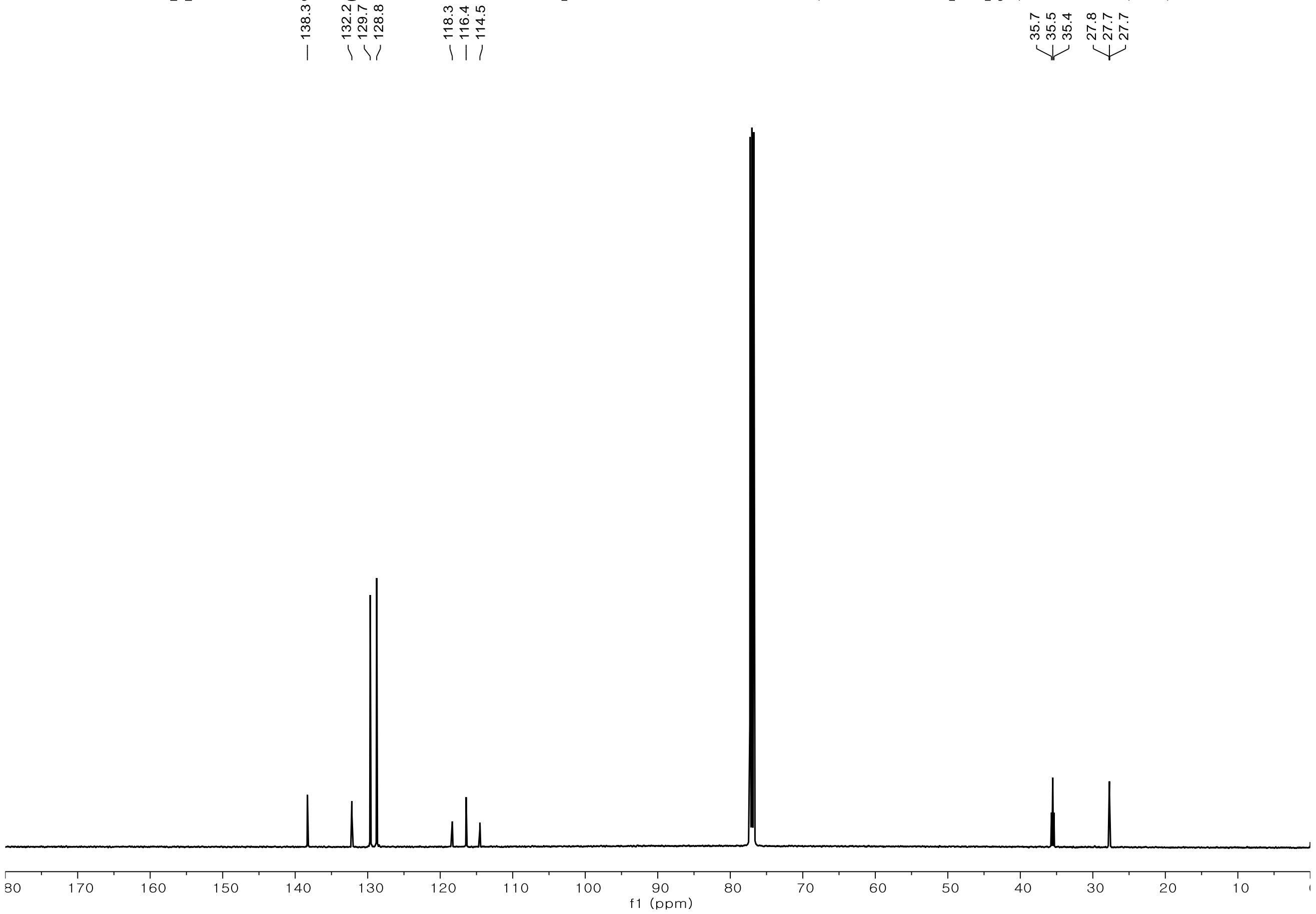

Supplementary Figure 27. ^{13}C NMR Spectrum of 1-Chloro-4-(3,3-difluoropropyl)benzene (**10f**)

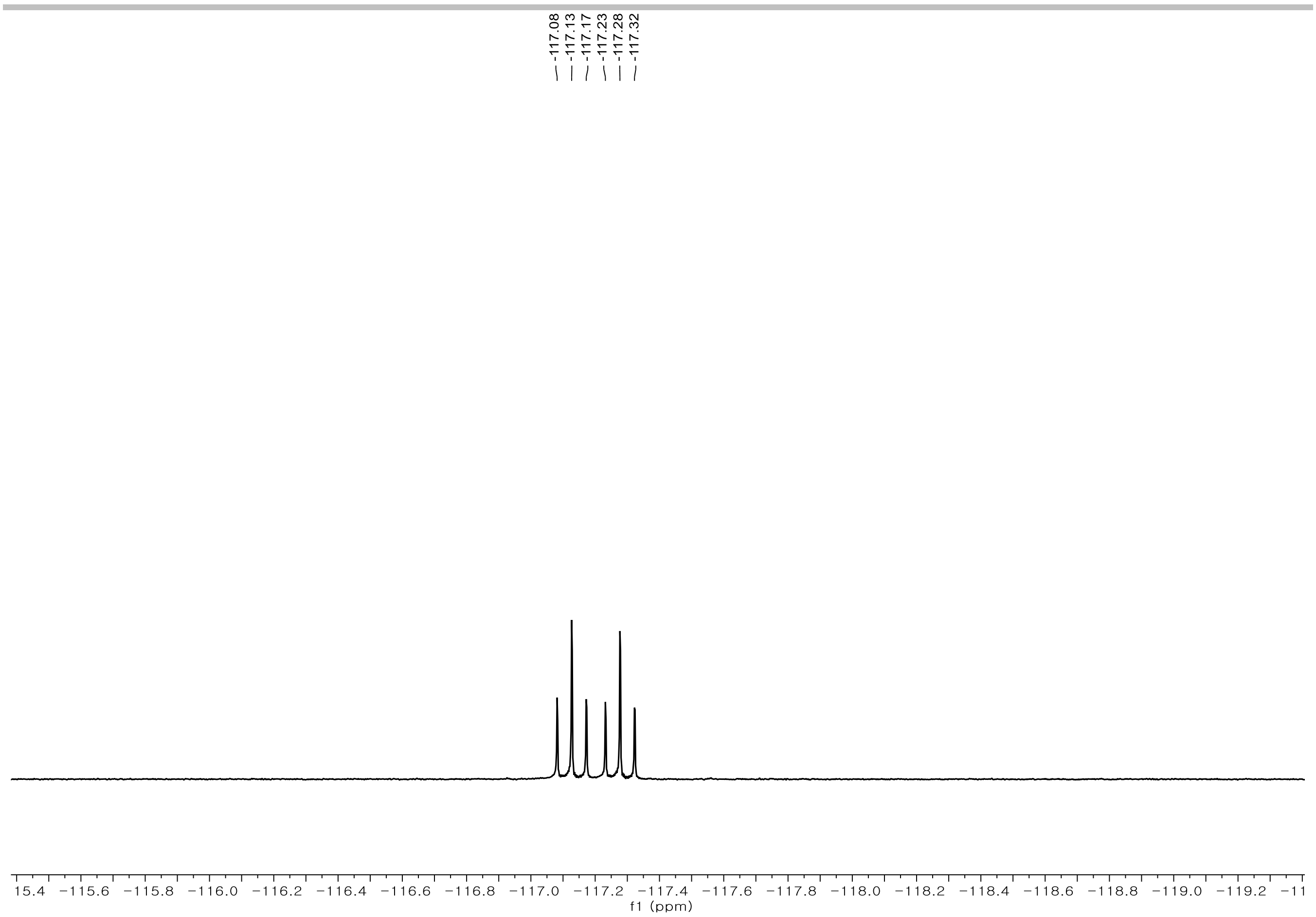

Supplementary Figure 28. ^{19}F NMR Spectrum of 1-Chloro-4-(3,3-difluoropropyl)benzene (**10f**)

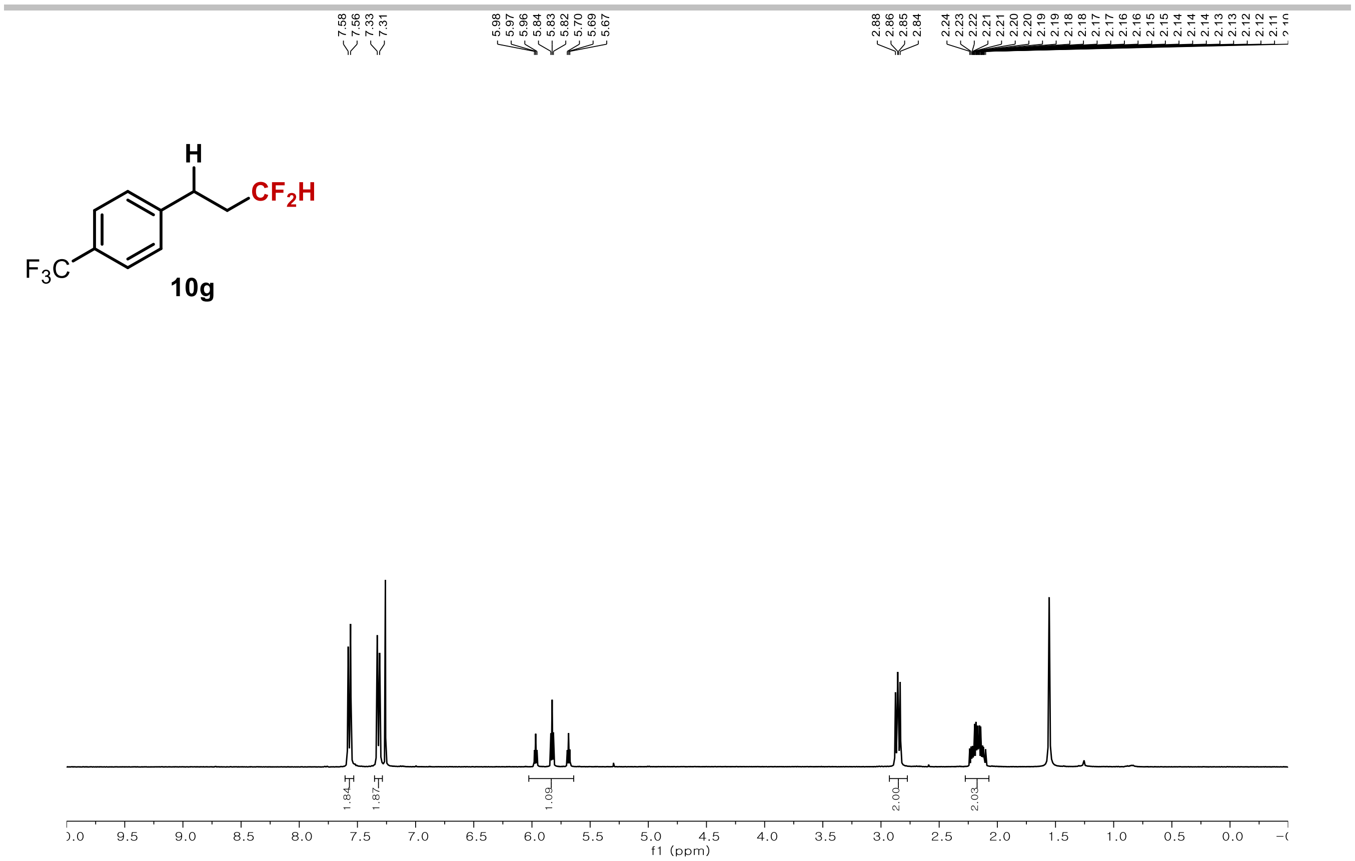

Supplementary Figure 29. ^{1}H NMR Spectrum of 1-(3,3-Difluoropropyl)-4-(trifluoromethyl)benzene (**10g**)

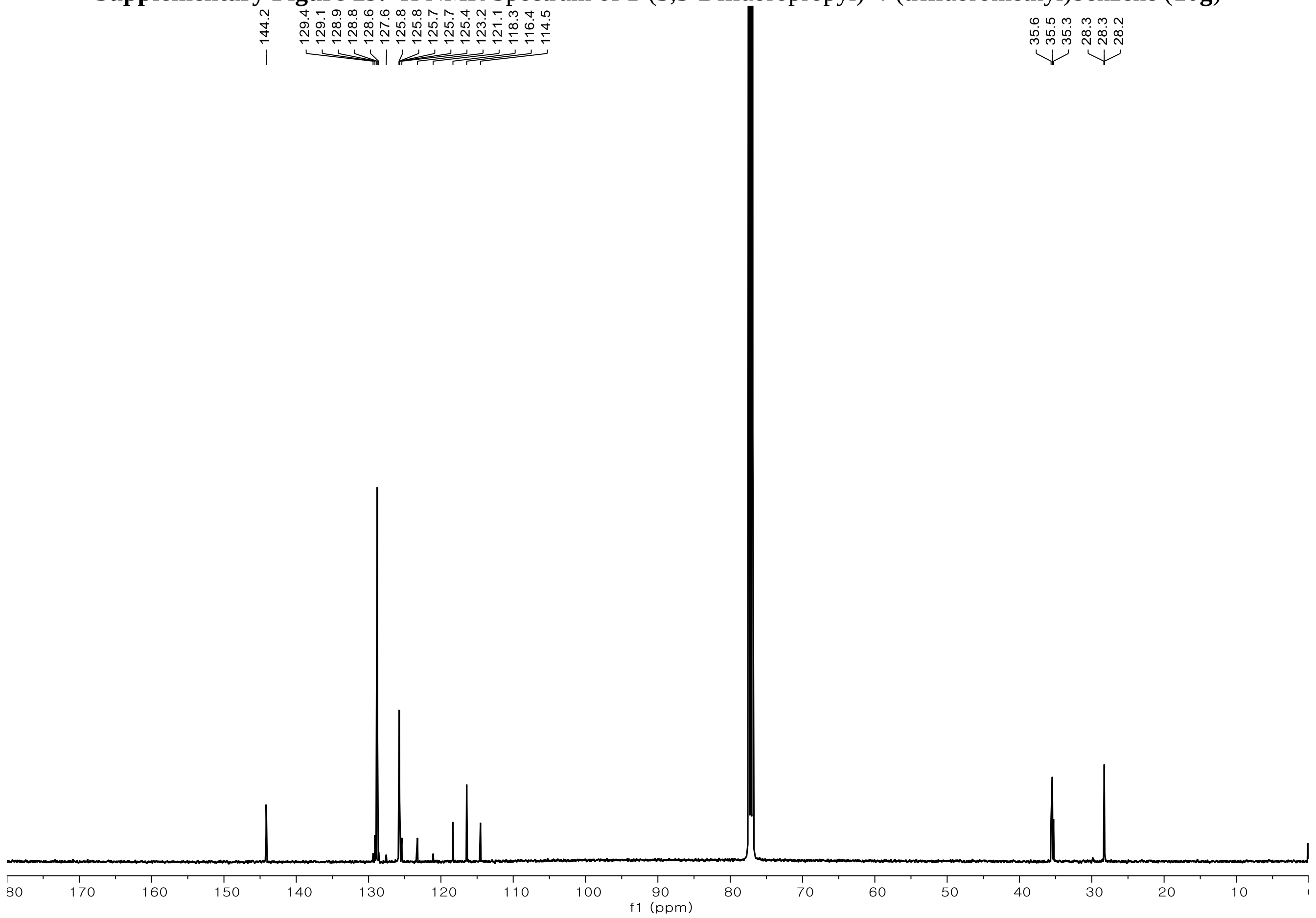

Supplementary Figure 30. ^{13}C NMR Spectrum of 1-(3,3-Difluoropropyl)-4-(trifluoromethyl)benzene (**10g**)

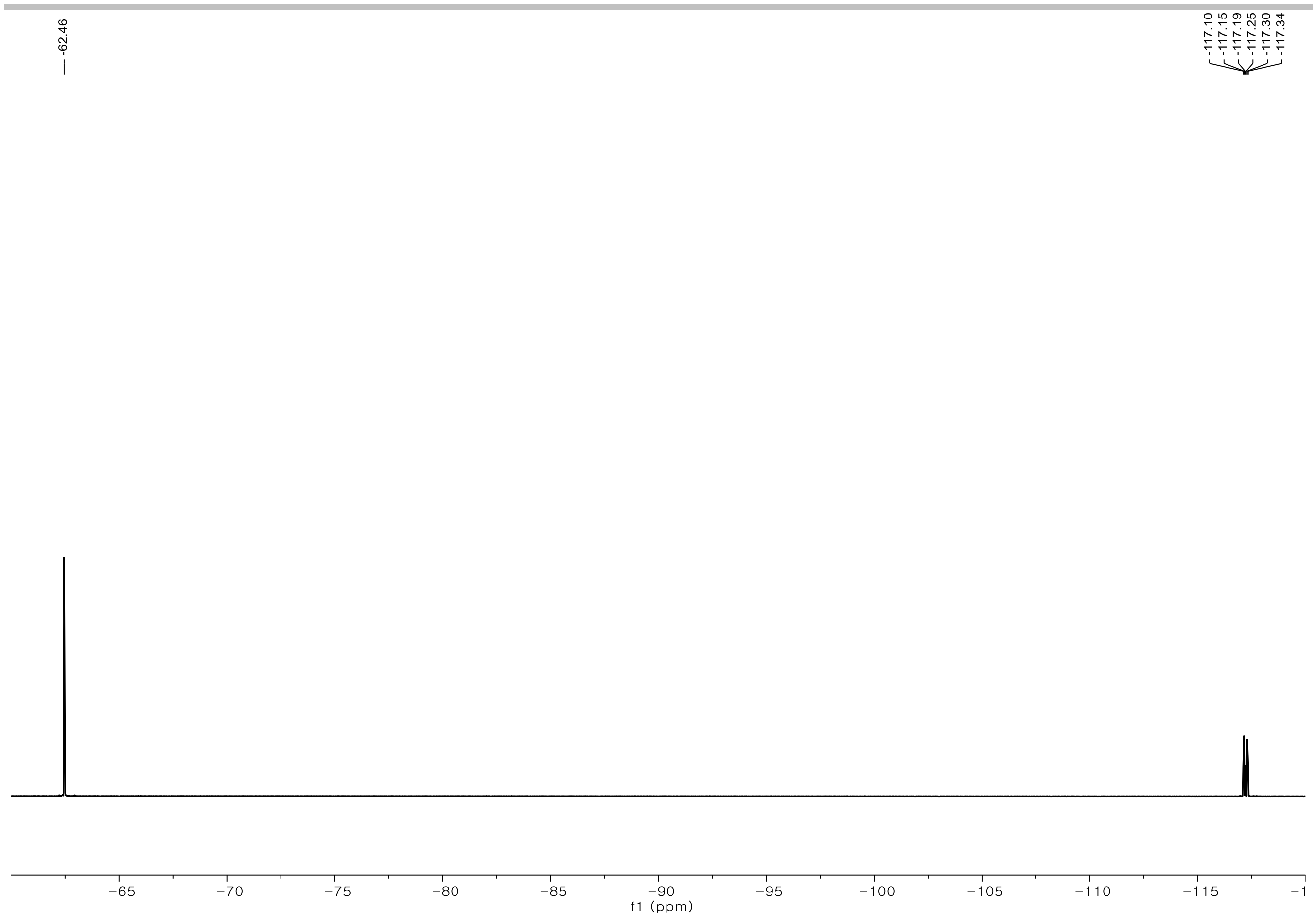

Supplementary Figure 31. ^{19}F NMR Spectrum of 1-(3,3-Difluoropropyl)-4-(trifluoromethyl)benzene (**10g**)

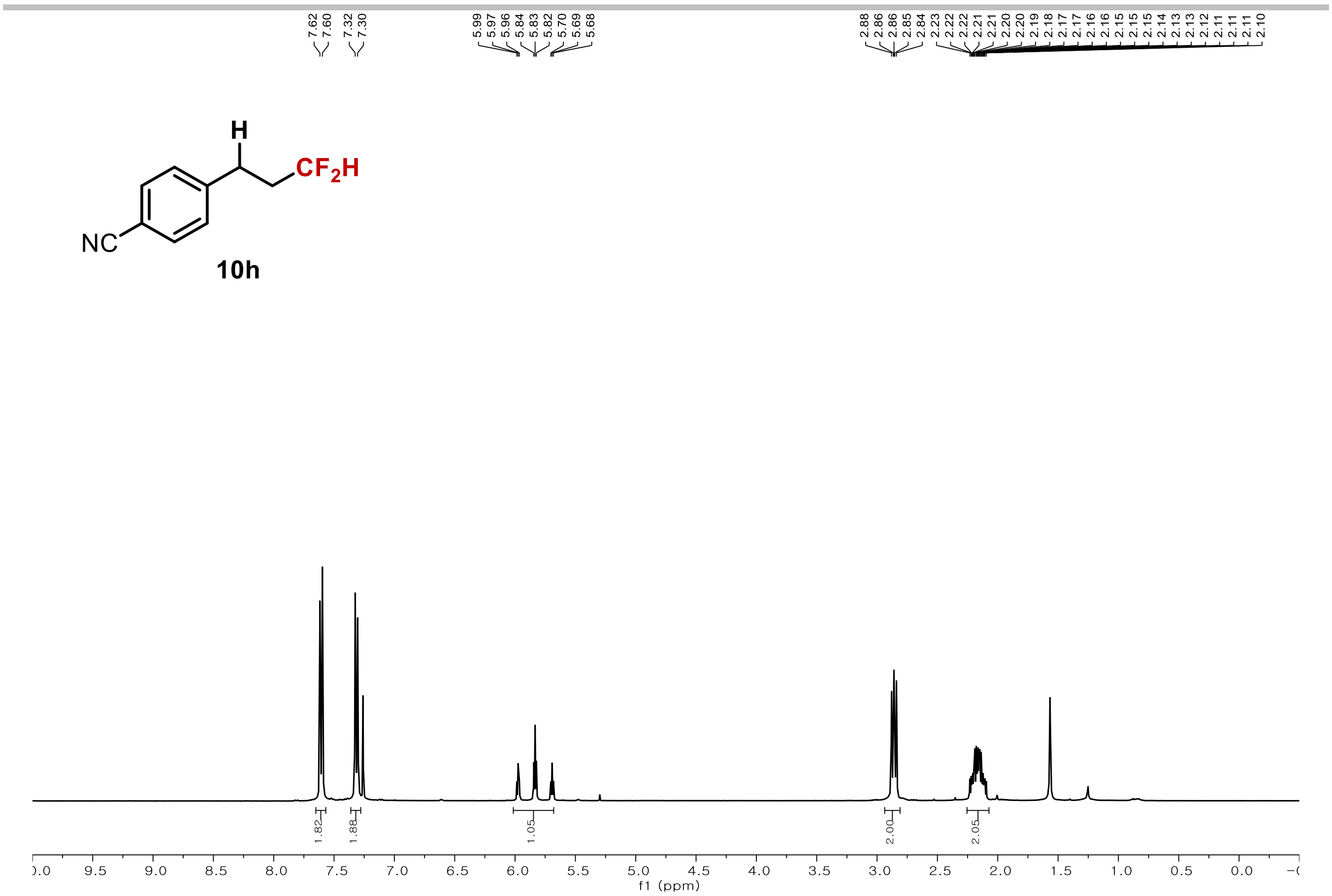

Supplementary Figure 32. ^{1}H NMR Spectrum of 4-(3,3-Difluoropropyl)benzonitrile (**10h**)

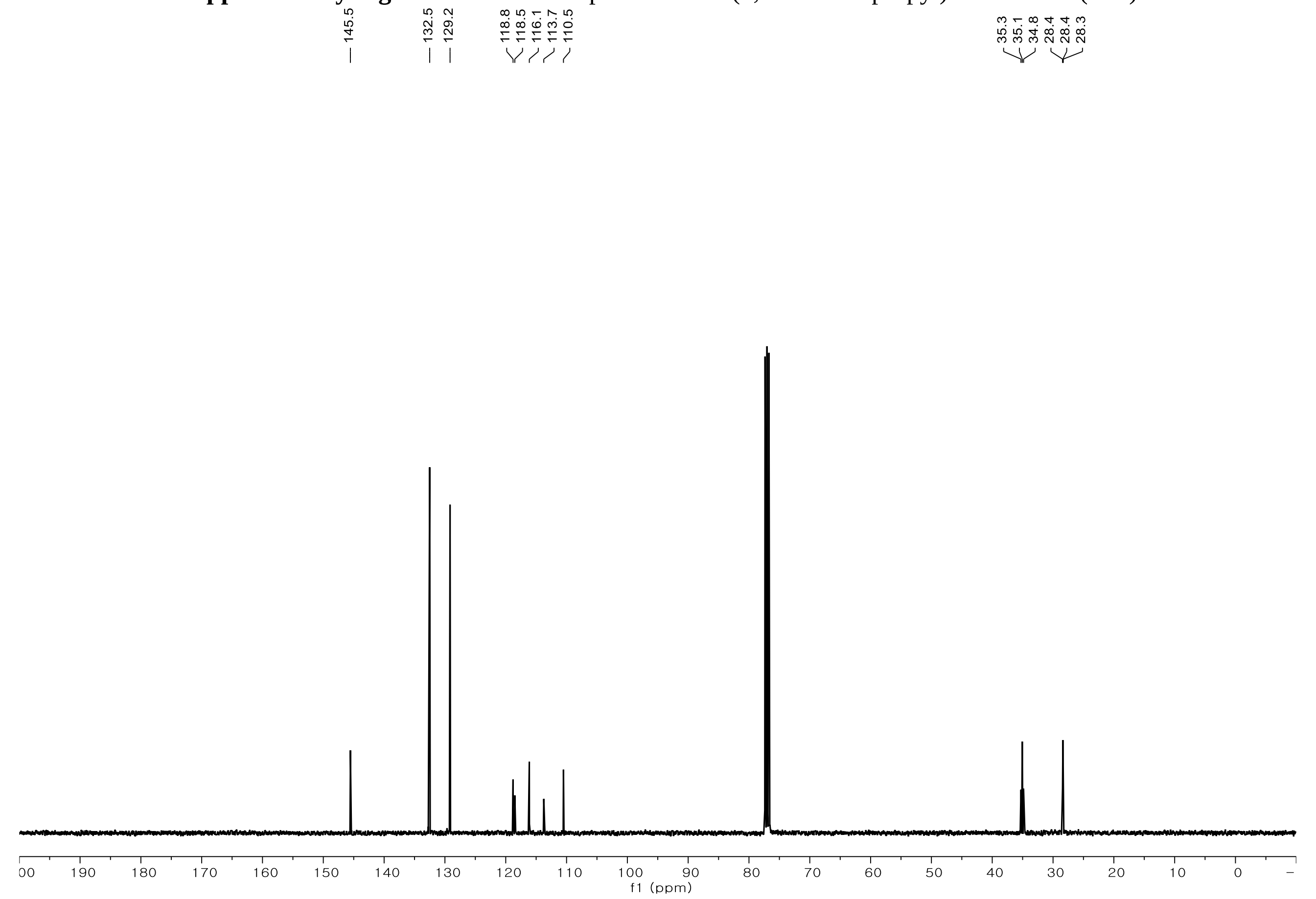

Supplementary Figure 33. ^{13}C NMR Spectrum of 4-(3,3-Difluoropropyl)benzonitrile (**10h**)

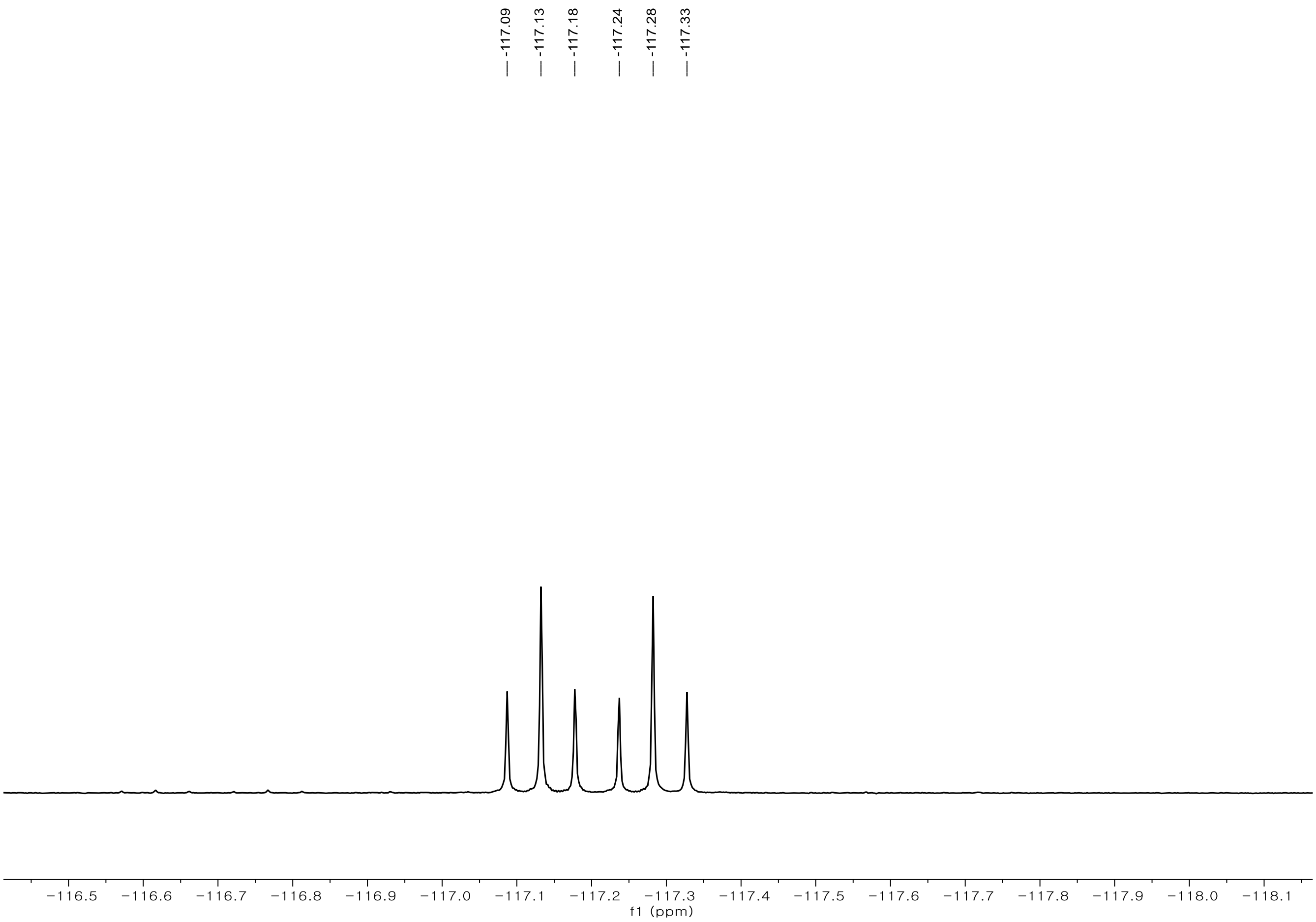

Supplementary Figure 34. ^{19}F NMR Spectrum of 4-(3,3-Difluoropropyl)benzonitrile (**10h**)

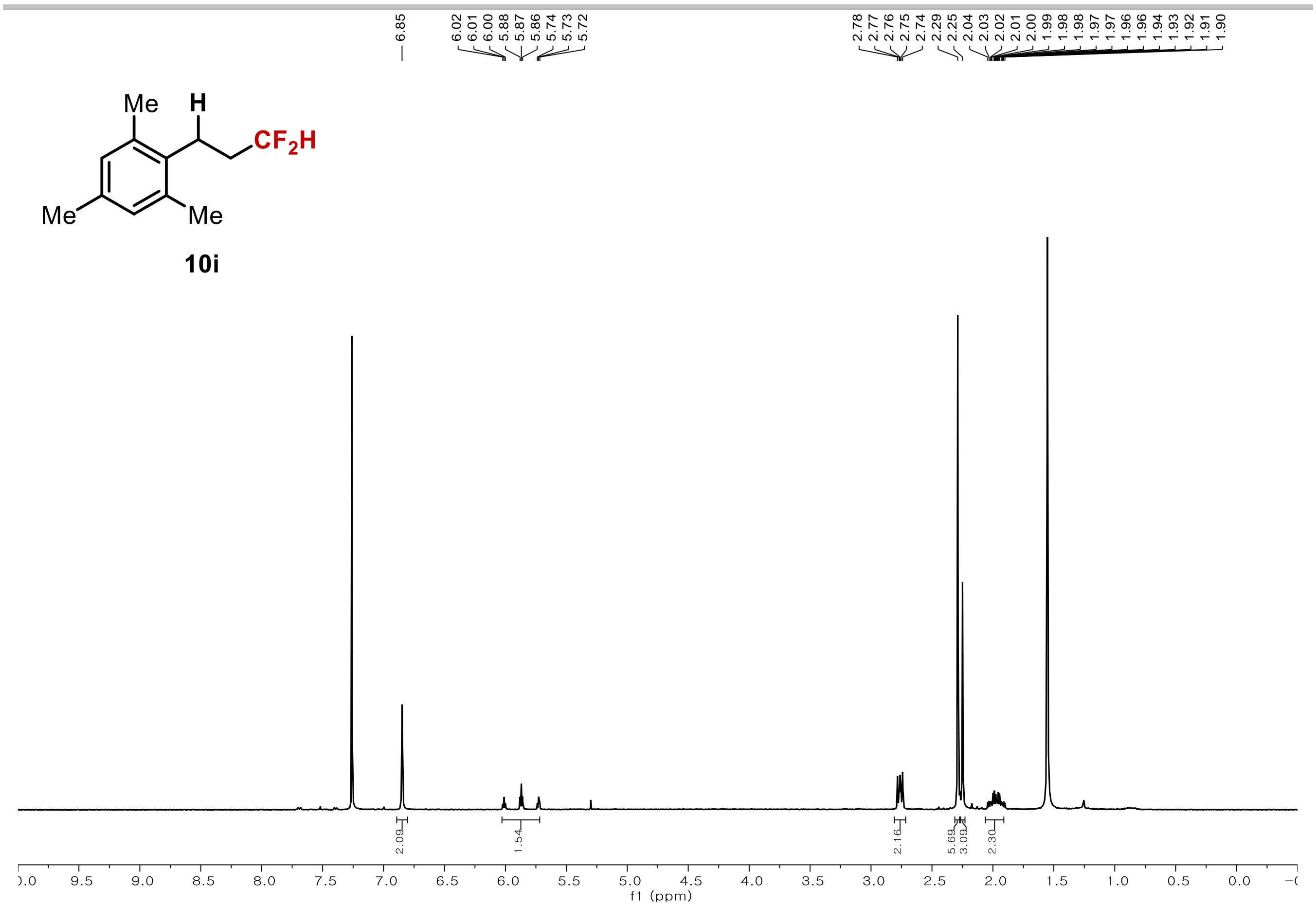

Supplementary Figure 35. ^{1}H NMR Spectrum of 2-(3,3-Difluoropropyl)-1,3,5-trimethylbenzene (**10i**)

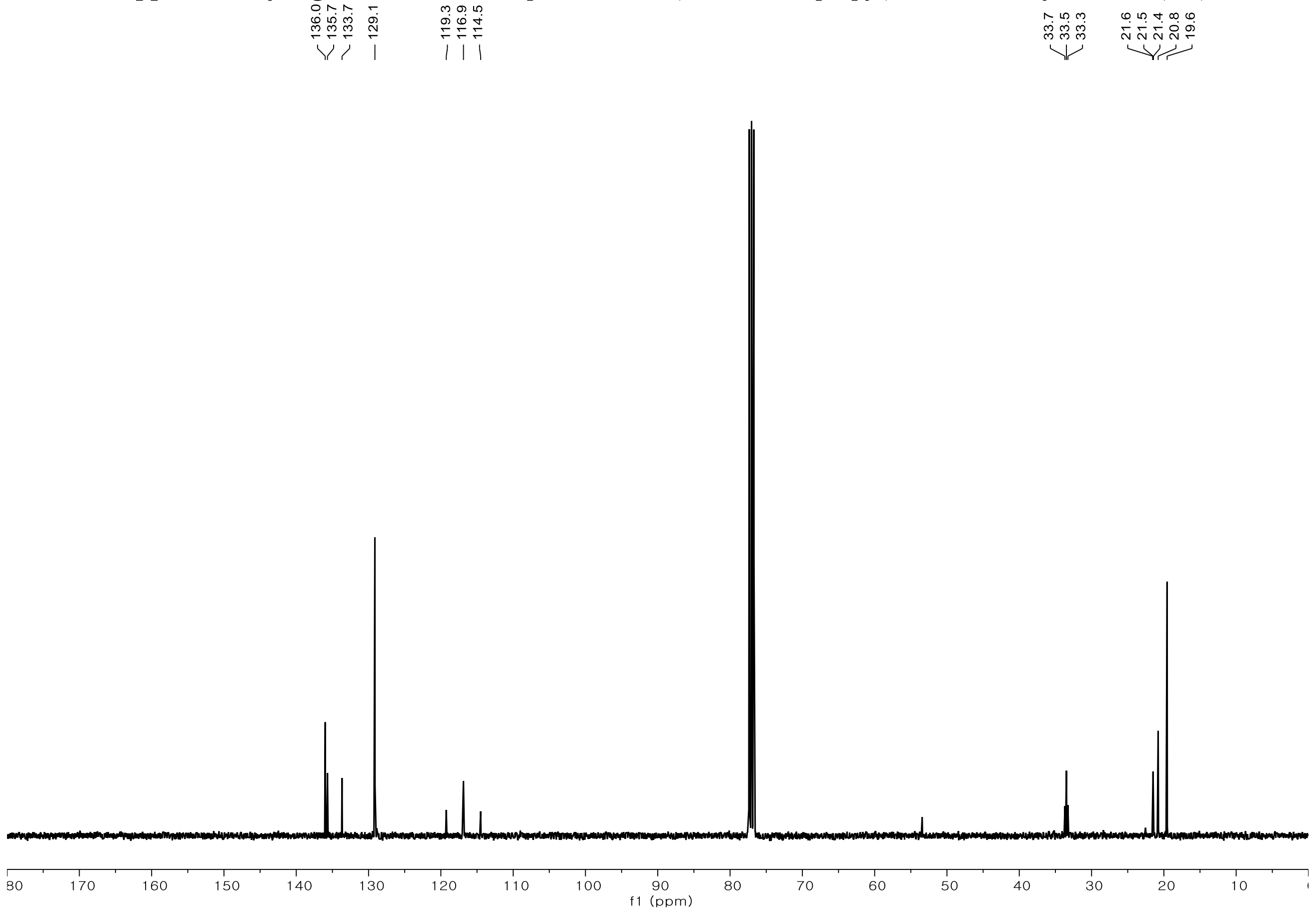

Supplementary Figure 36. ^{13}C NMR Spectrum of 2-(3,3-Difluoropropyl)-1,3,5-trimethylbenzene (**10i**)

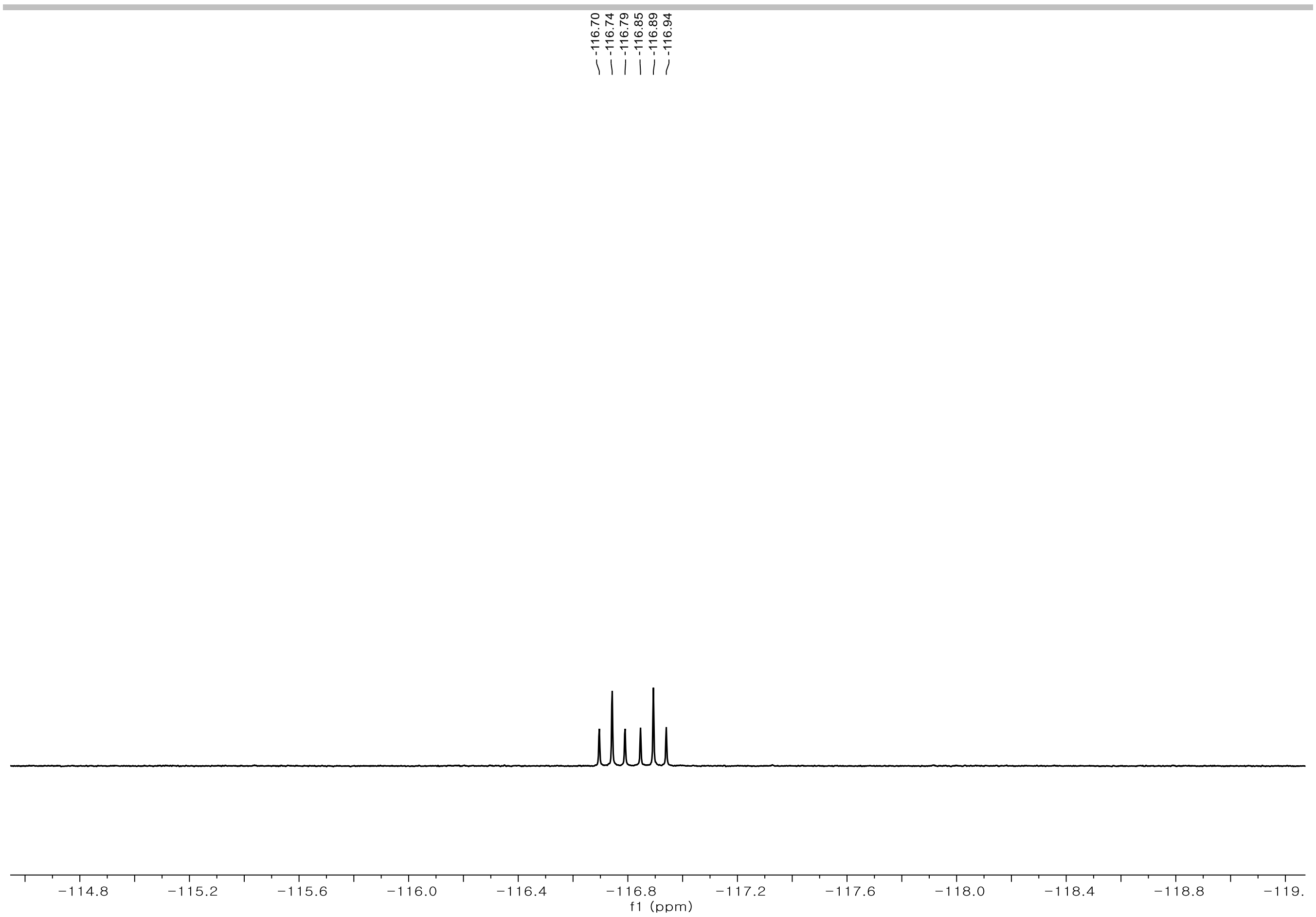

Supplementary Figure 37. ^{19}F NMR Spectrum of 2-(3,3-Difluoropropyl)-1,3,5-trimethylbenzene (**10i**)

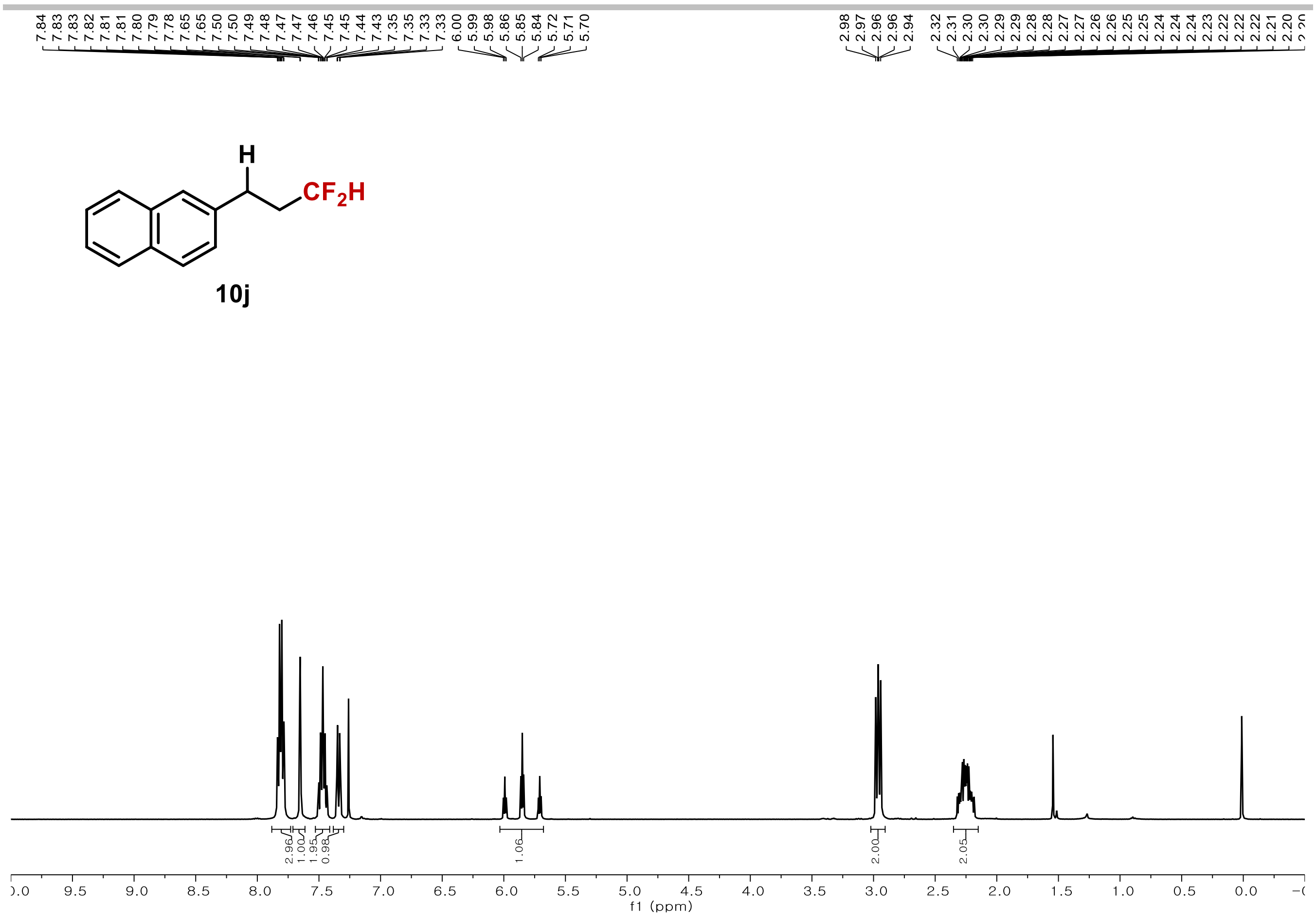

Supplementary Figure 38. ^{1}H NMR Spectrum of 2-(3,3-Difluoropropyl)naphthalene (**10j**)

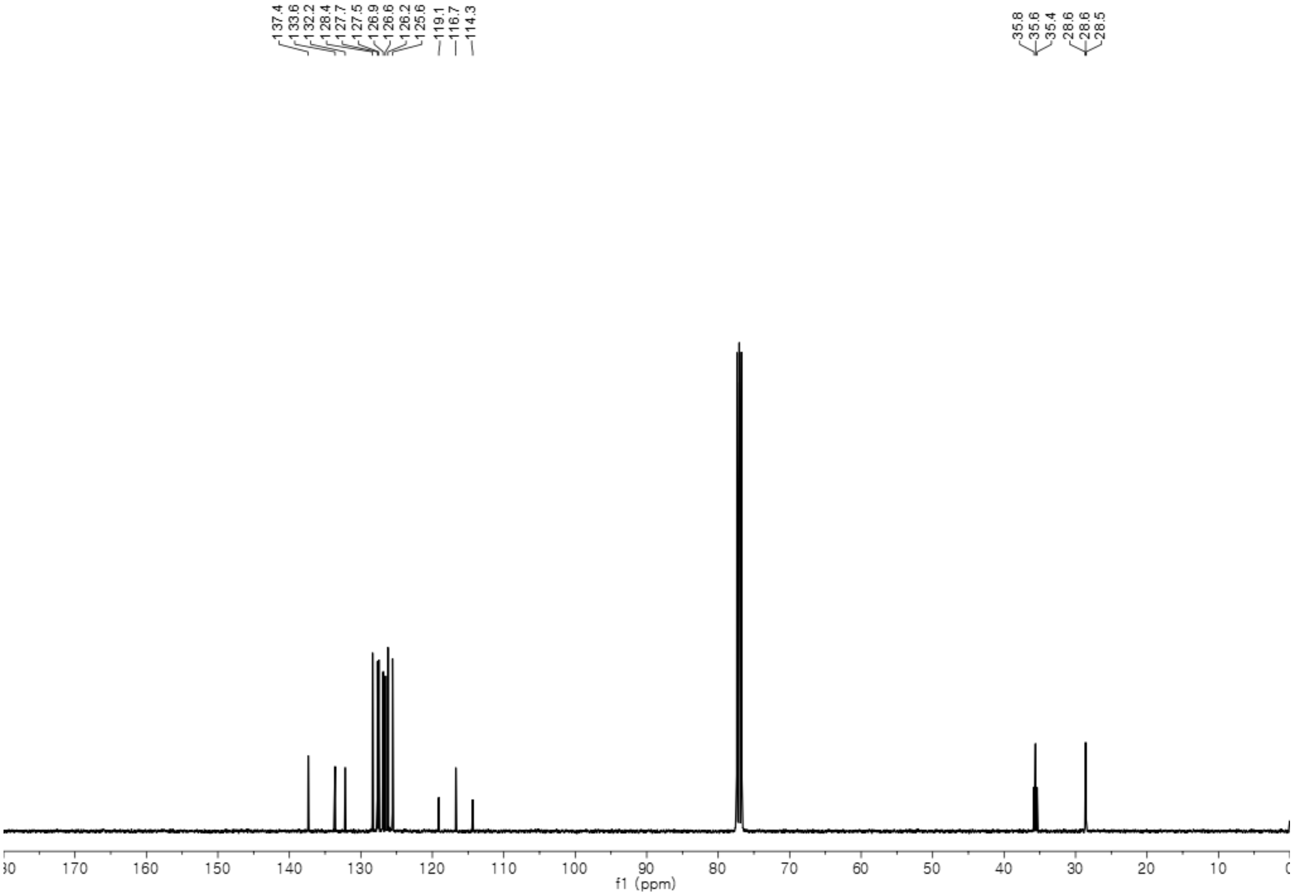

Supplementary Figure 39. ^{13}C NMR Spectrum of 2-(3,3-Difluoropropyl)naphthalene (**10j**)

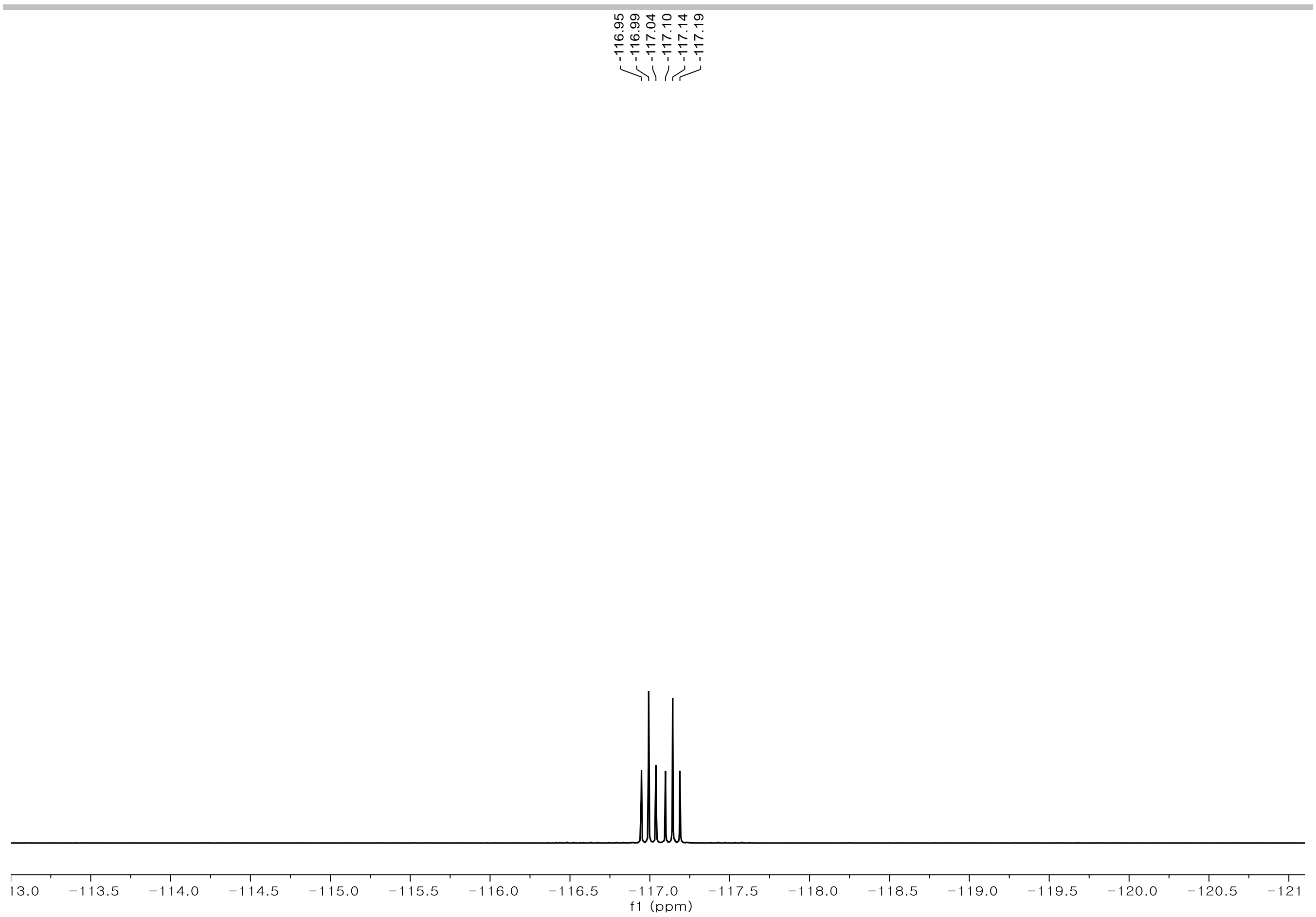

Supplementary Figure 40. ^{19}F NMR Spectrum of 2-(3,3-Difluoropropyl)naphthalene (**10j**)

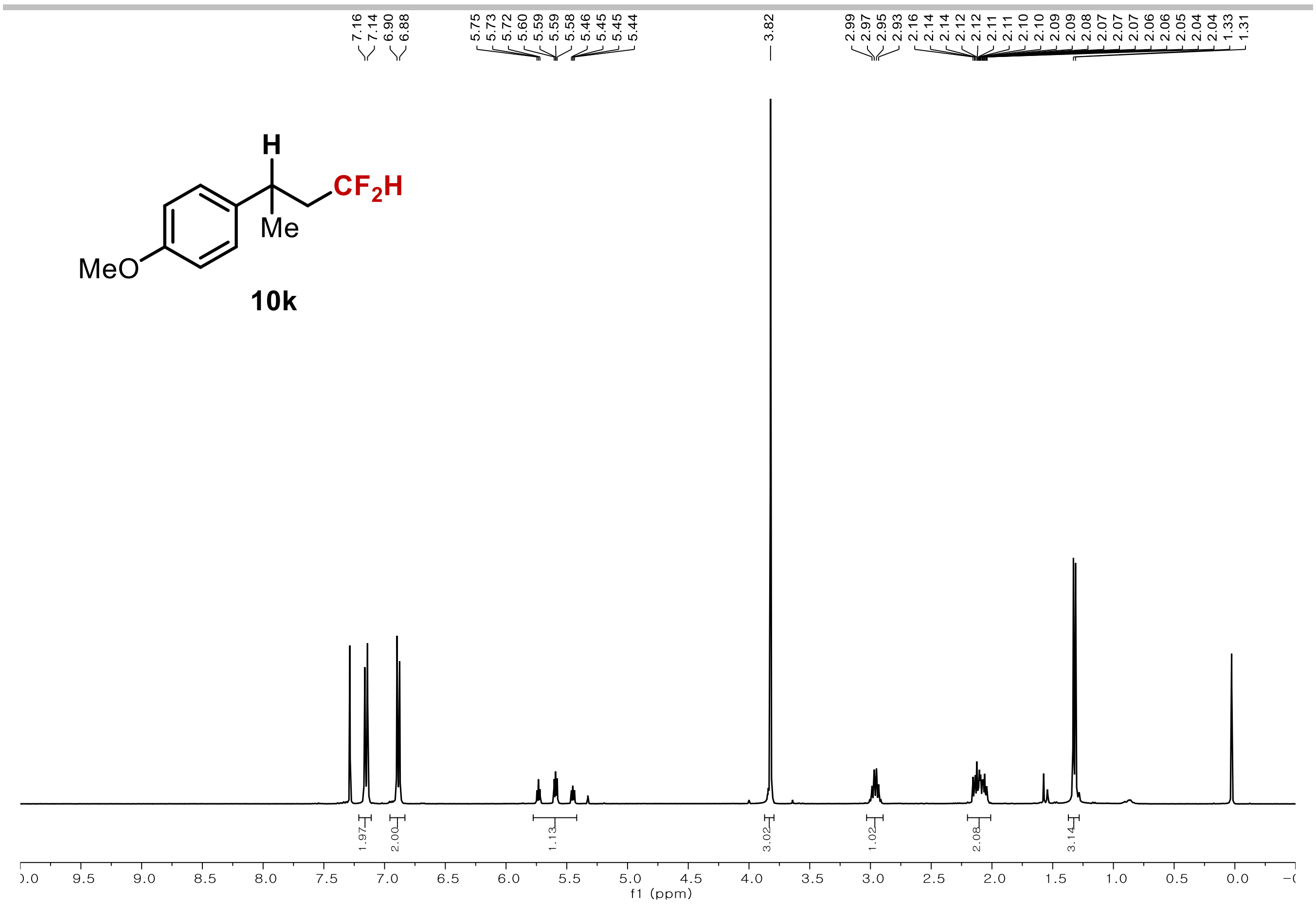

Supplementary Figure 41. ^{1}H NMR Spectrum of 1-(4,4-difluorobutan-2-yl)-4-methoxybenzene (**10k**)

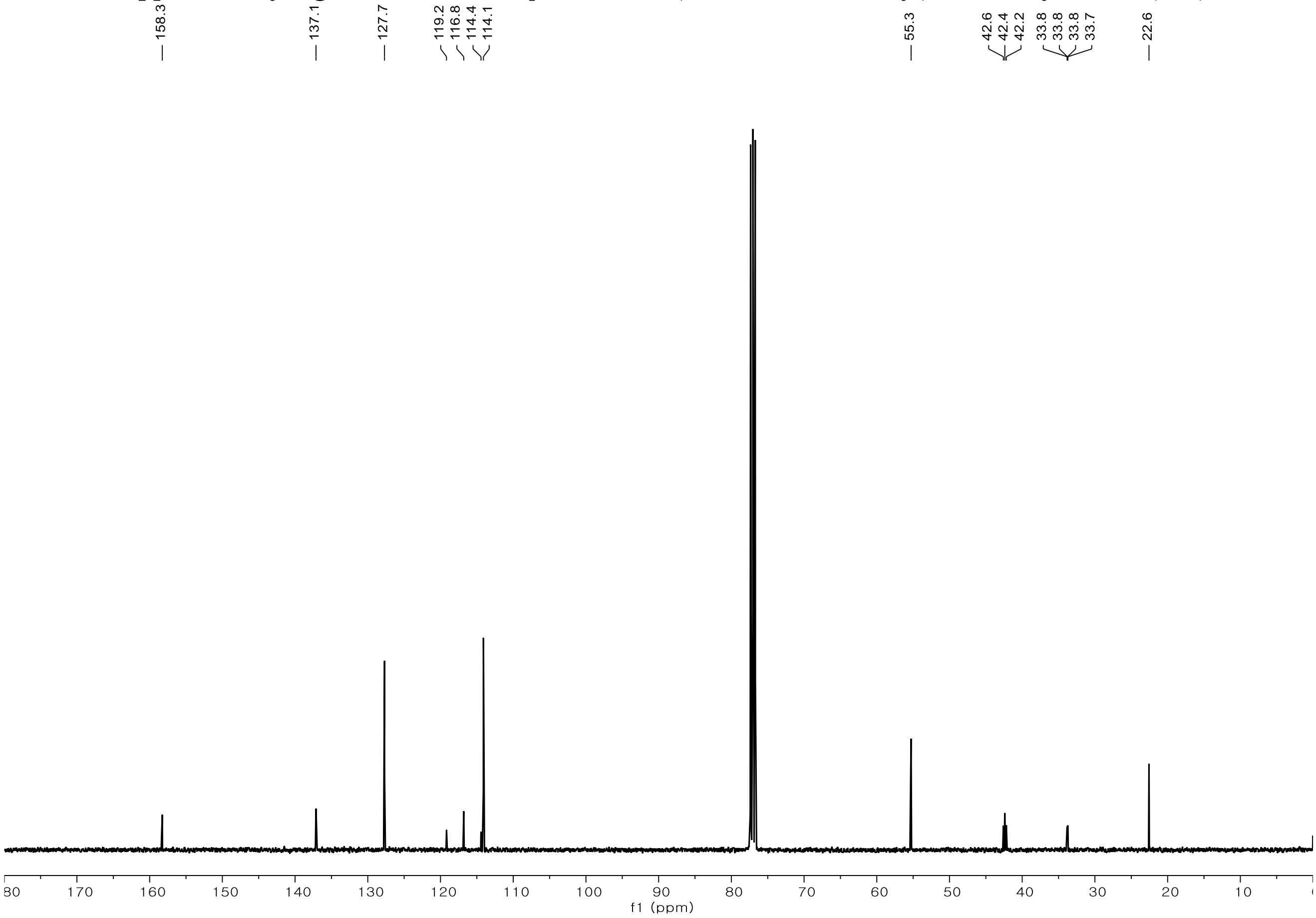

Supplementary Figure 42. ^{13}C NMR Spectrum of 1-(4,4-difluorobutan-2-yl)-4-methoxybenzene (**10k**)

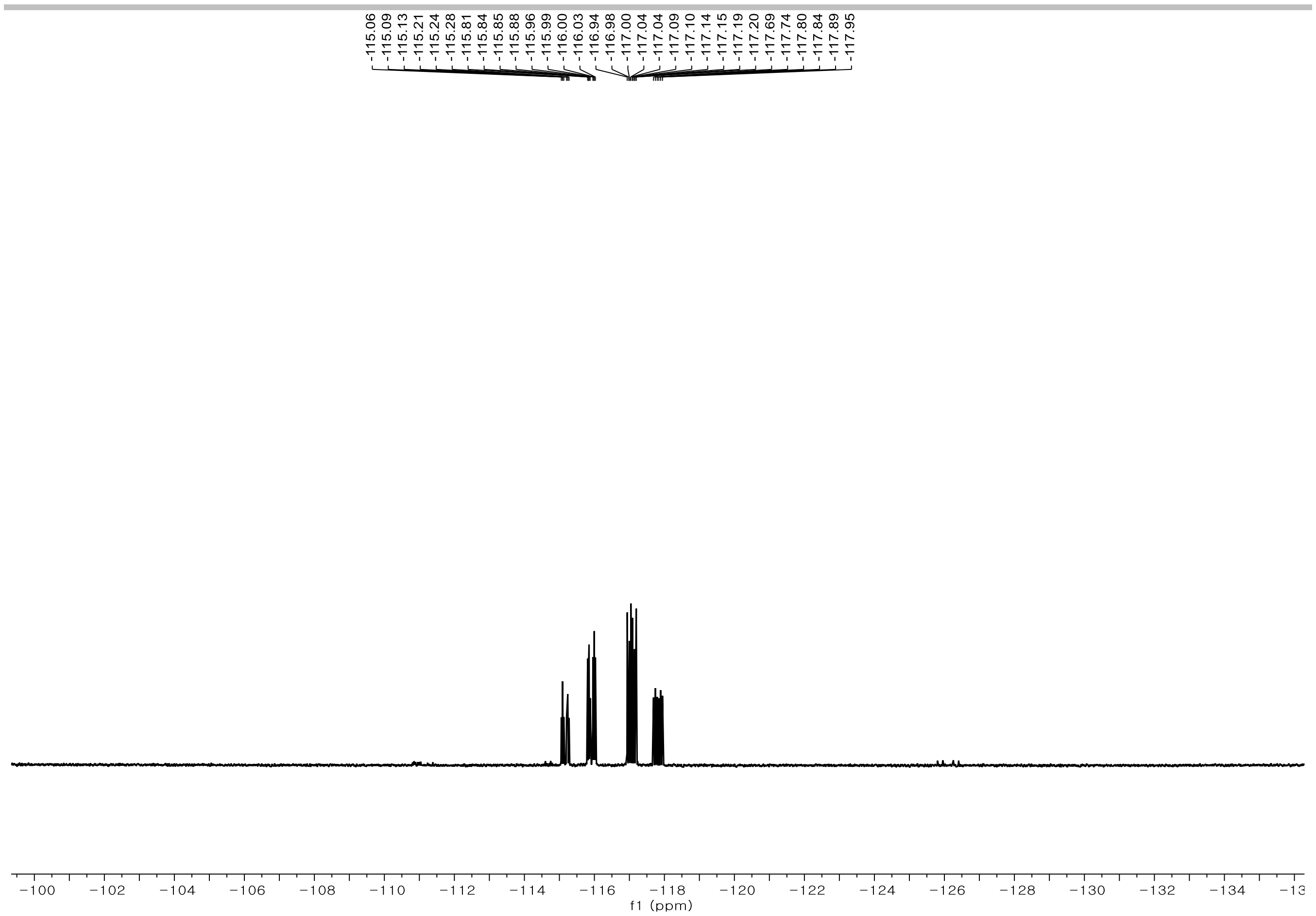

Supplementary Figure 43. ^{19}F NMR Spectrum of 1-(4,4-difluorobutan-2-yl)-4-methoxybenzene (**10k**)

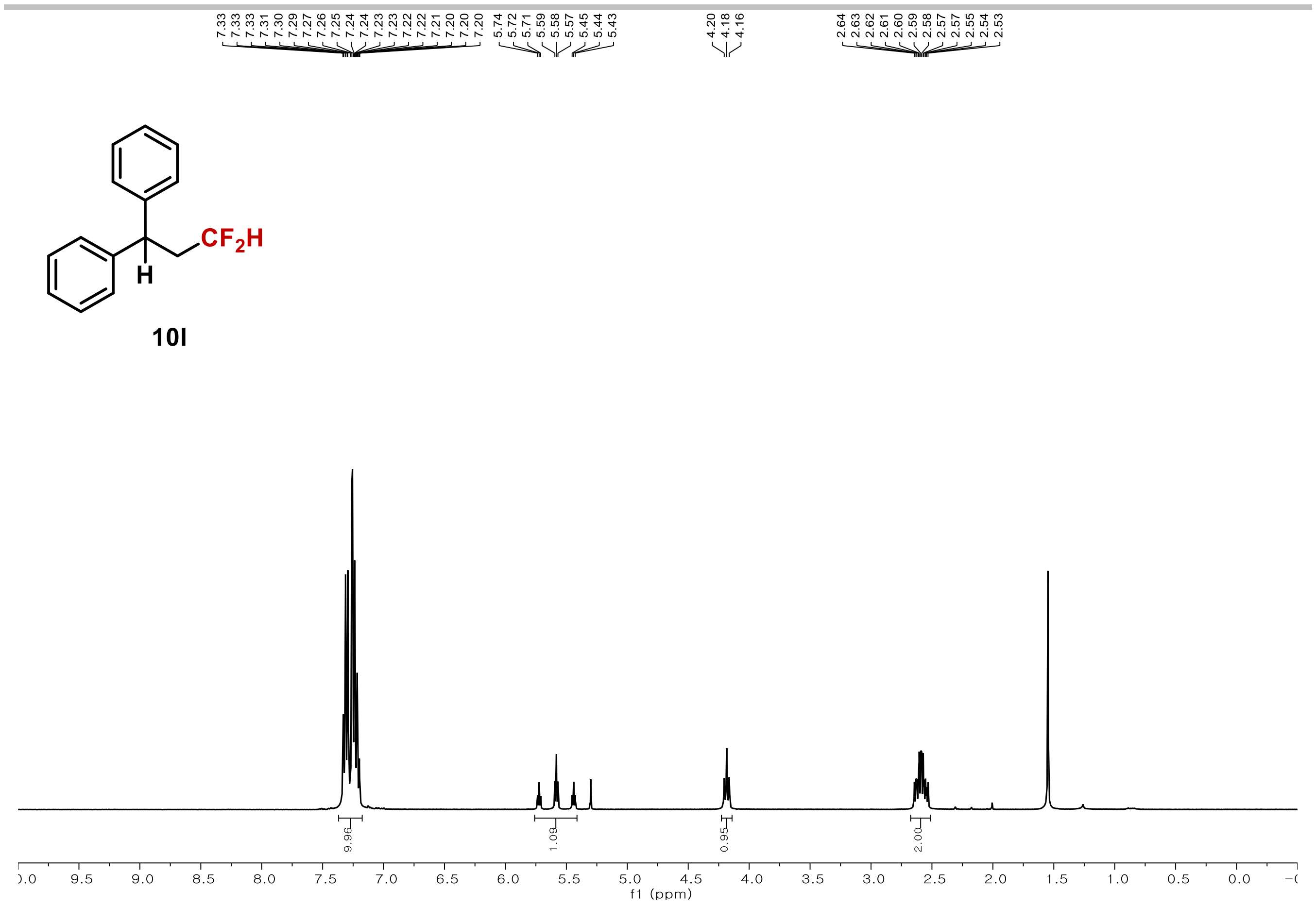

Supplementary Figure 44. ^{1}H NMR Spectrum of (3,3-Difluoropropane-1,1-diyl)dibenzene (**10l**)

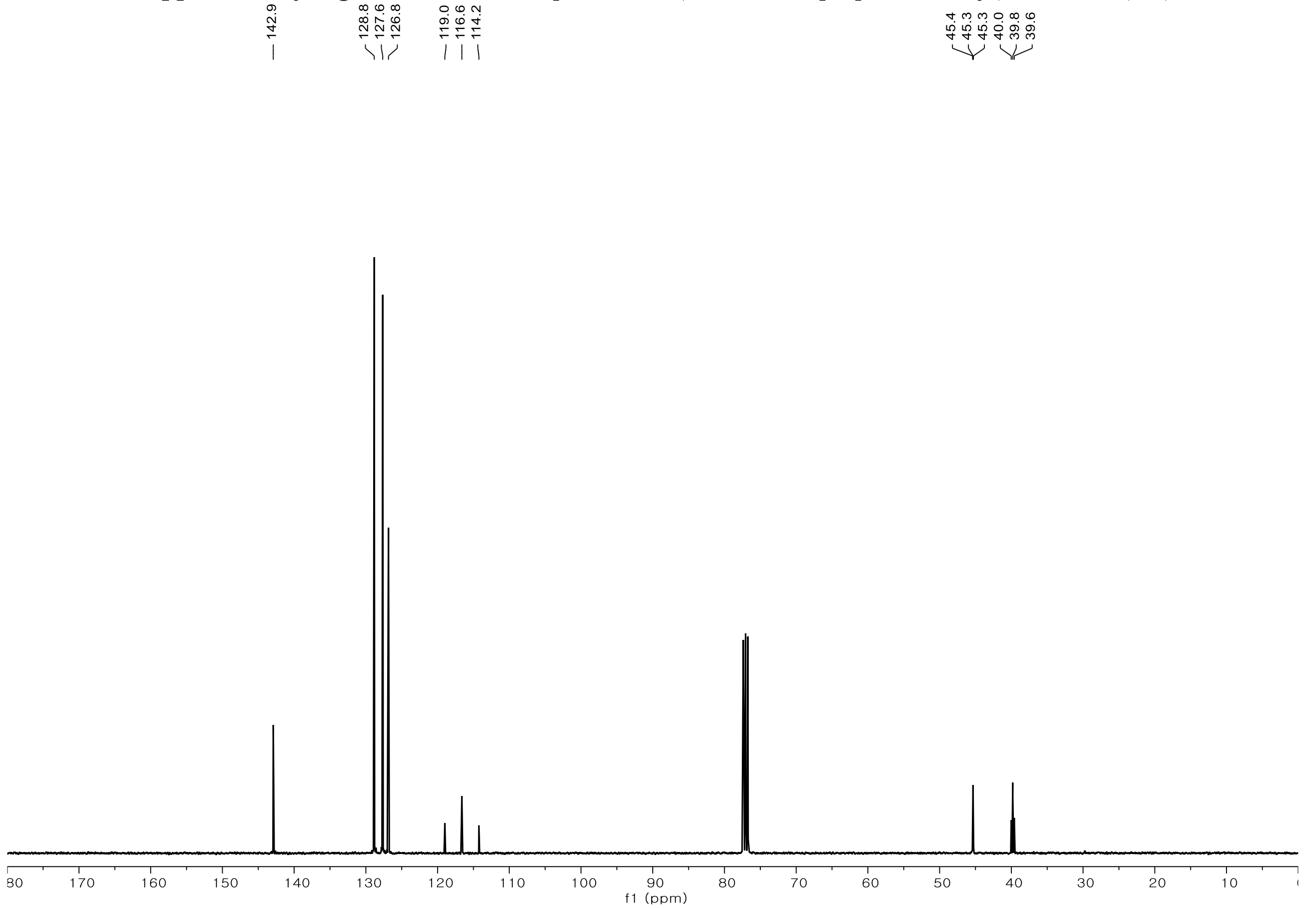

Supplementary Figure 45. ^{13}C NMR Spectrum of (3,3-Difluoropropane-1,1-diyl)dibenzene (**10l**)

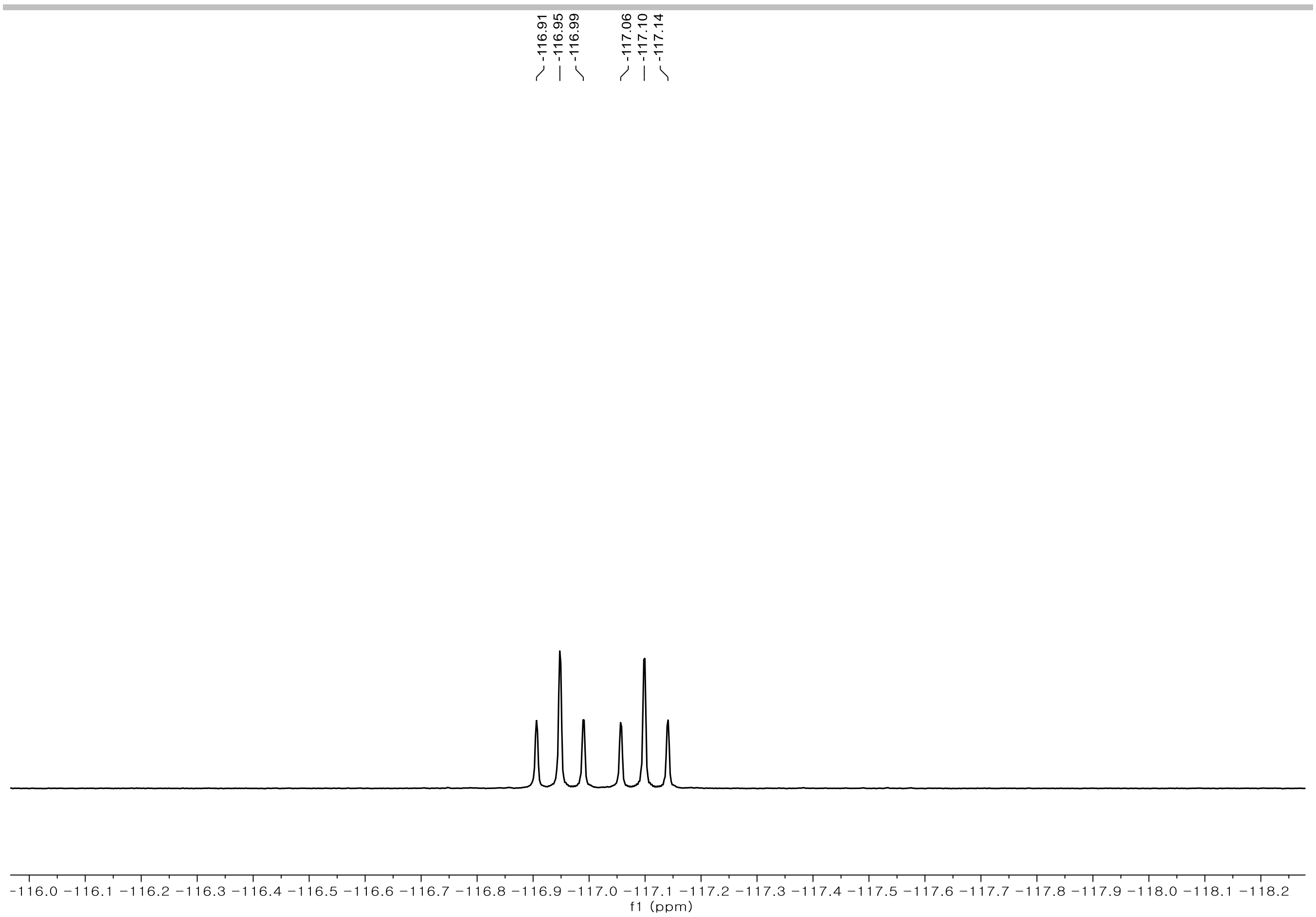

Supplementary Figure 46. ^{19}F NMR Spectrum of (3,3-Difluoropropane-1,1-diyl)dibenzene (**10l**)

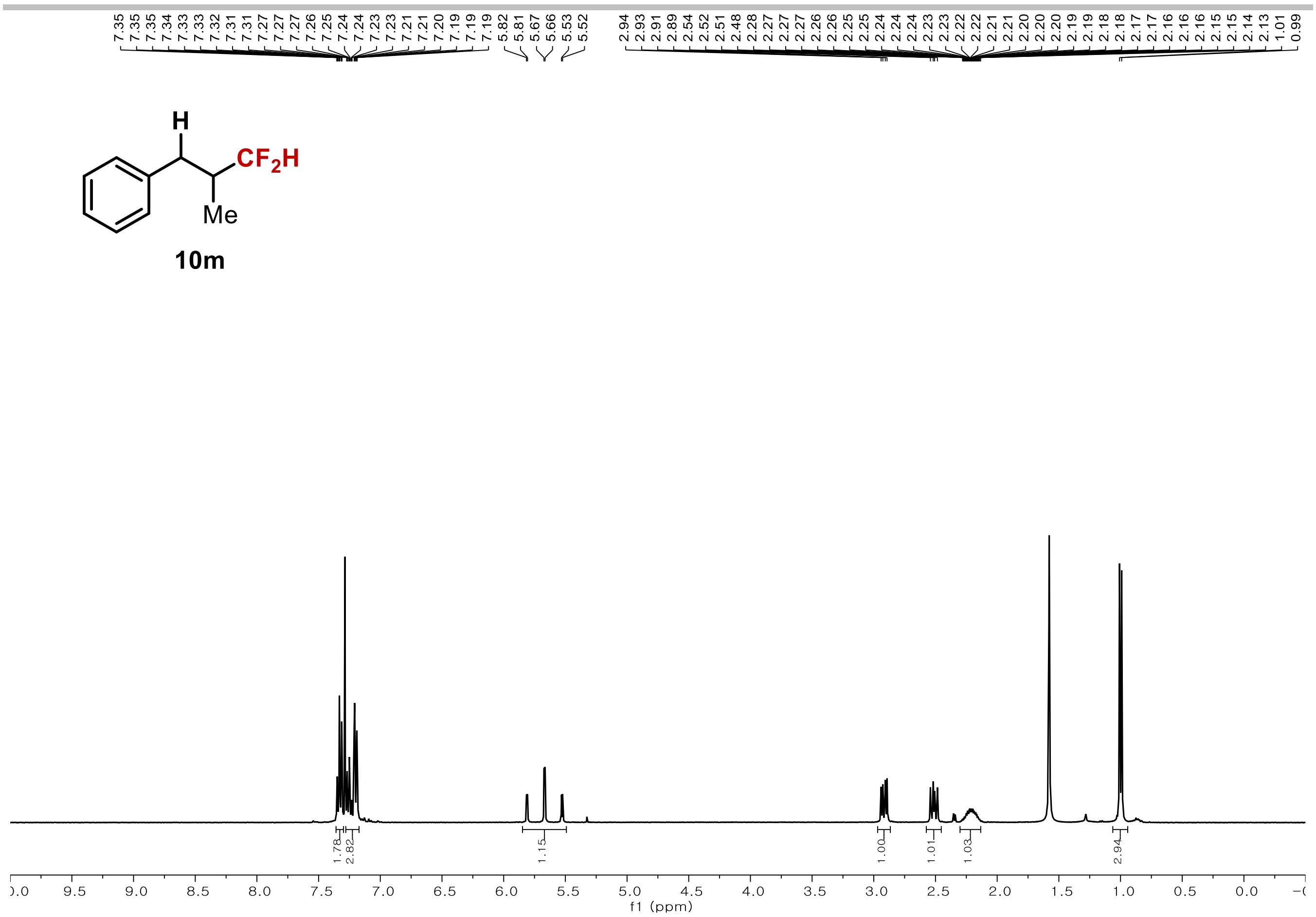

Supplementary Figure 47. ^{1}H NMR Spectrum of 2-(3,3-Difluoropropyl)-1,3,5-trimethylbenzene (**10m**)

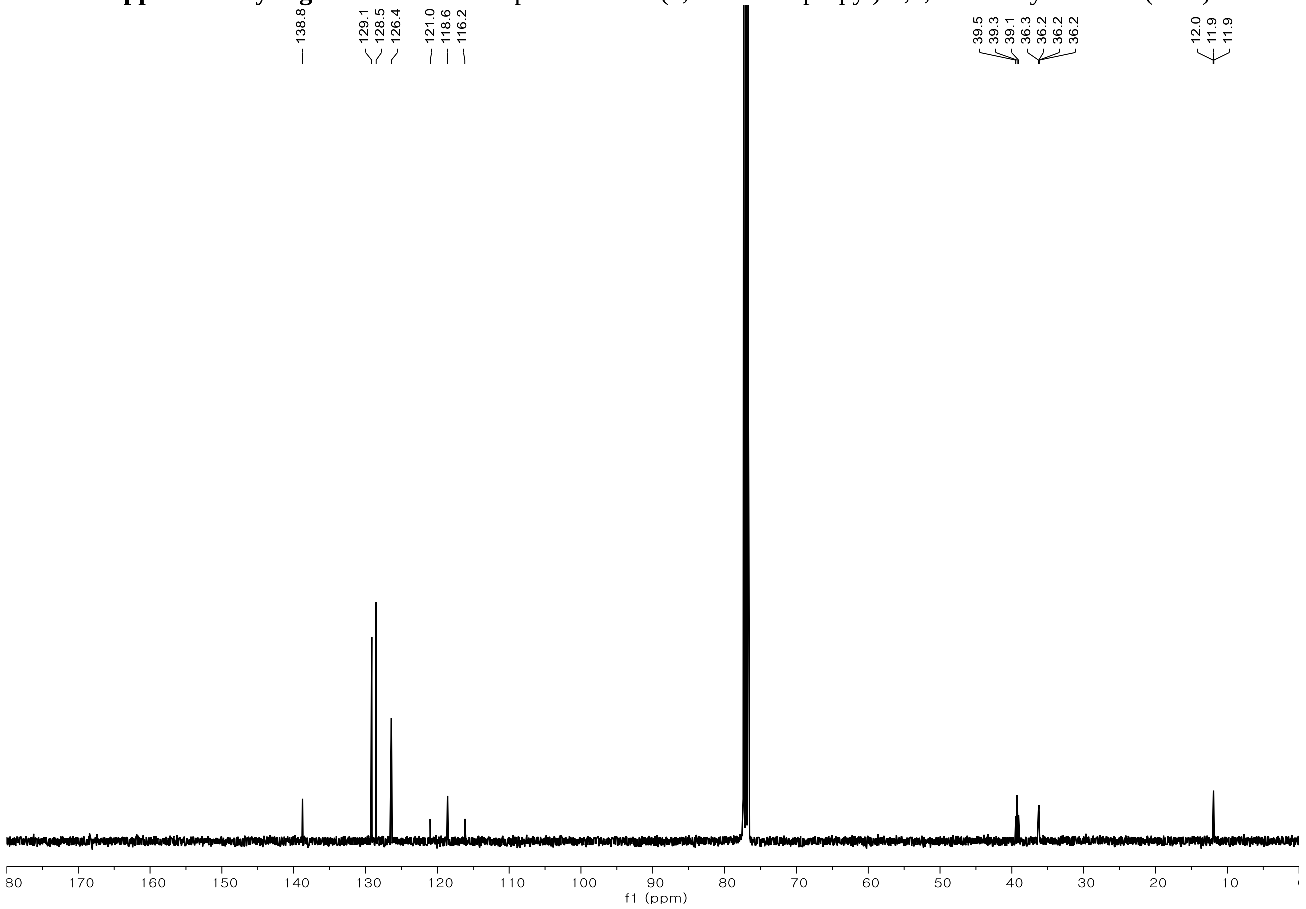

Supplementary Figure 48. ^{13}C NMR Spectrum of 2-(3,3-Difluoropropyl)-1,3,5-trimethylbenzene (**10m**)

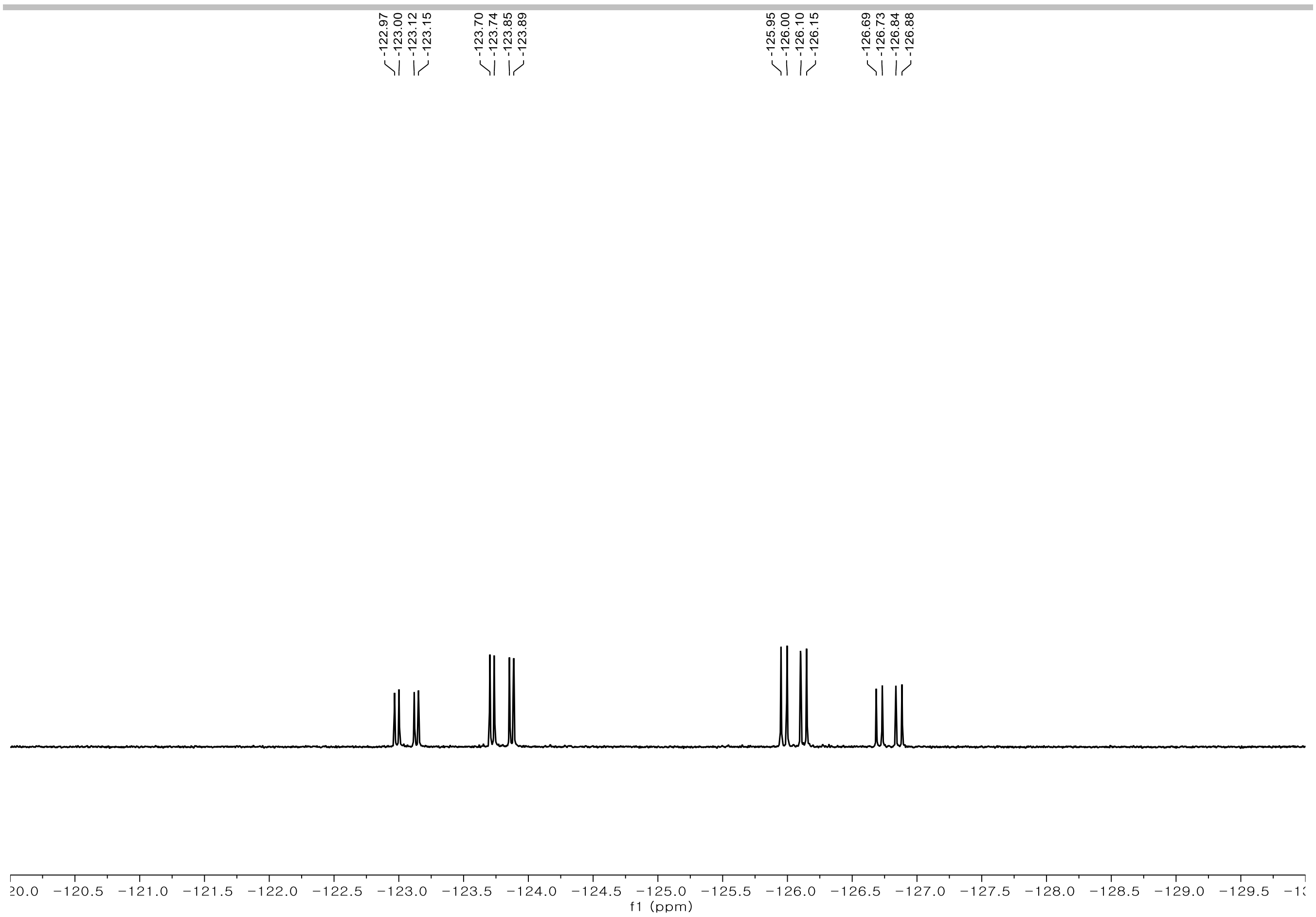

Supplementary Figure 49. ^{19}F NMR Spectrum of 2-(3,3-Difluoropropyl)-1,3,5-trimethylbenzene (**10m**)

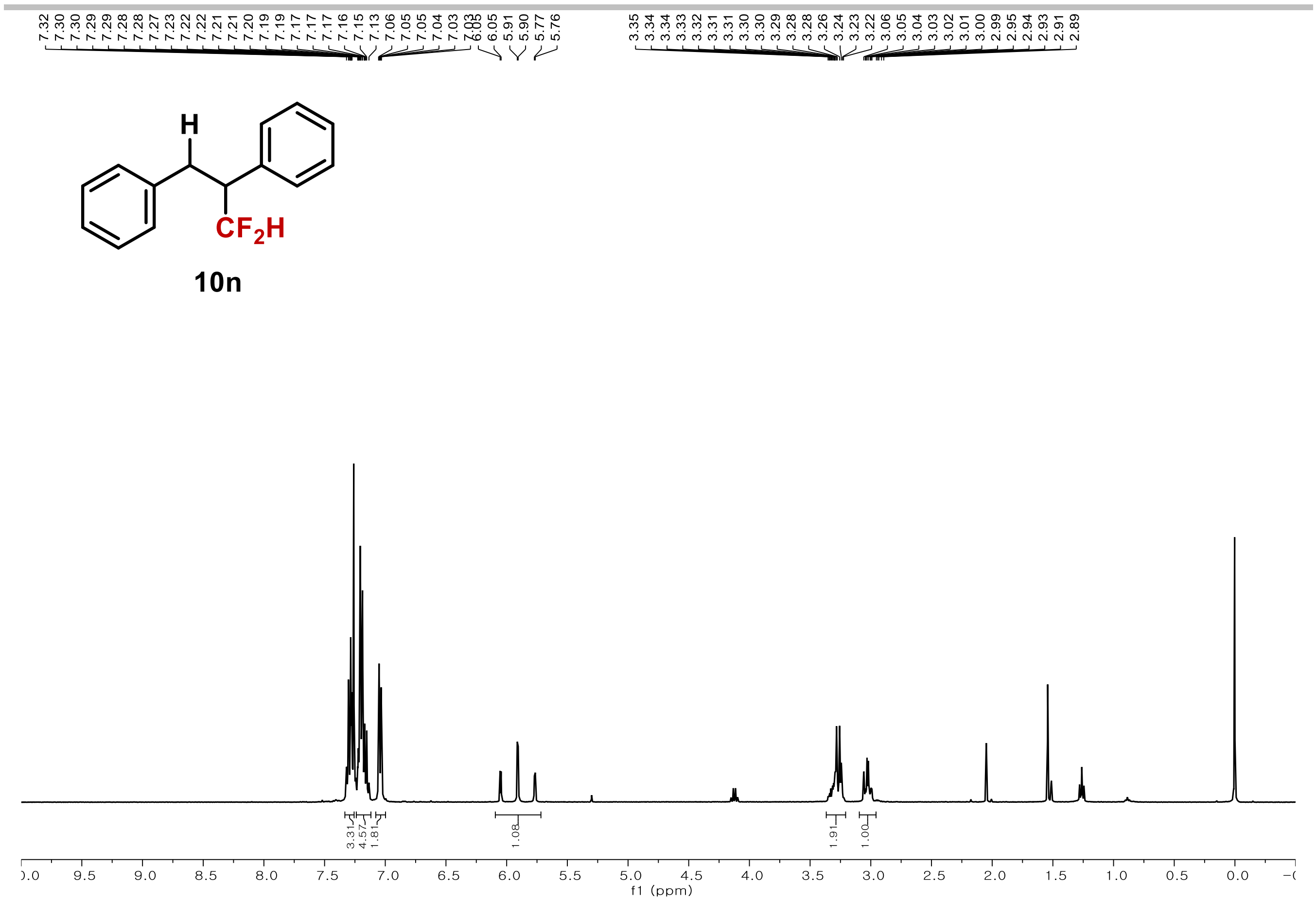

Supplementary Figure 50. ^{1}H NMR Spectrum of (3,3-difluoropropane-1,2-diyl)dibenzene (**10n**)

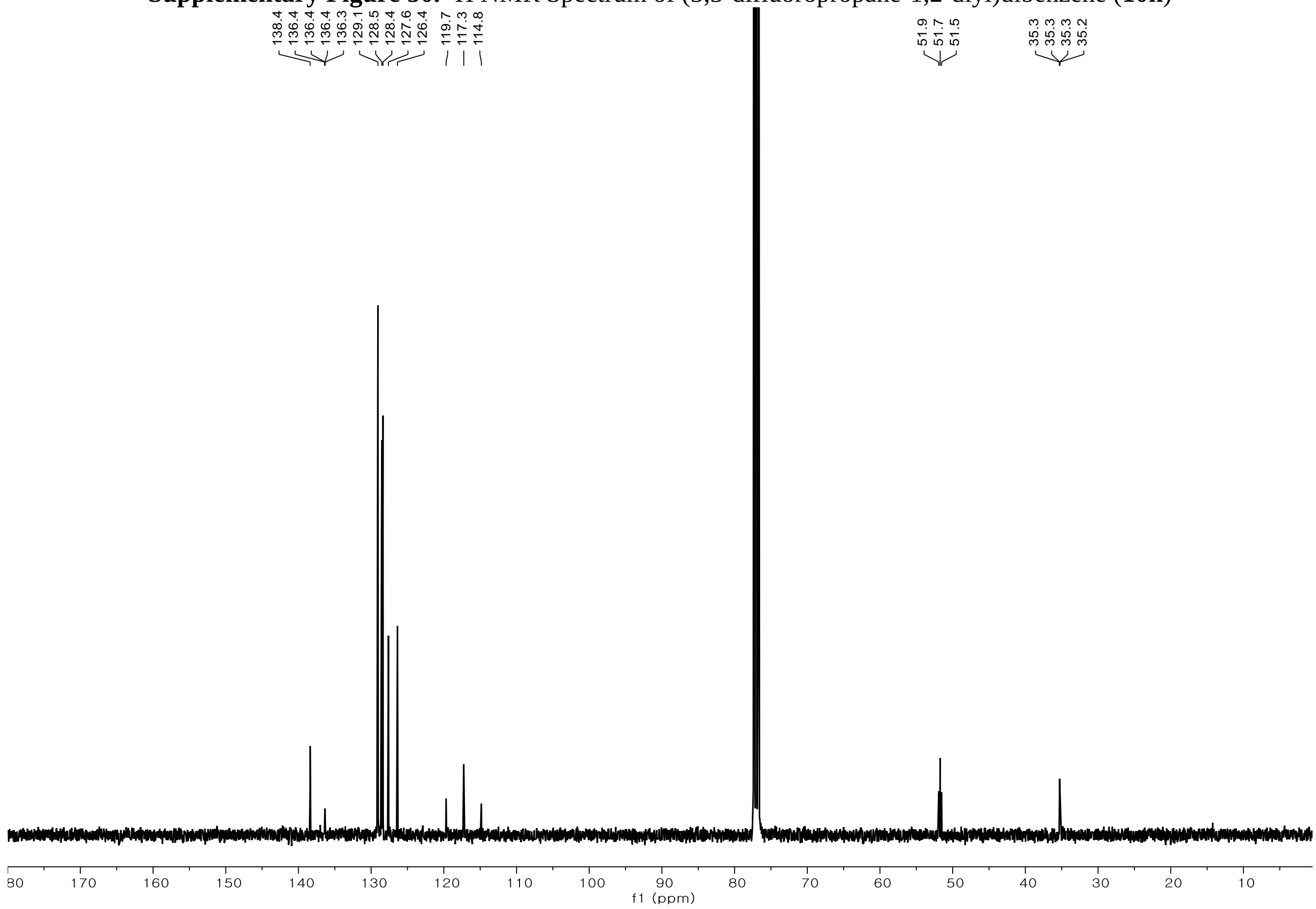

Supplementary Figure 51. ^{13}C NMR Spectrum of (3,3-difluoropropane-1,2-diyl)dibenzene (**10n**)

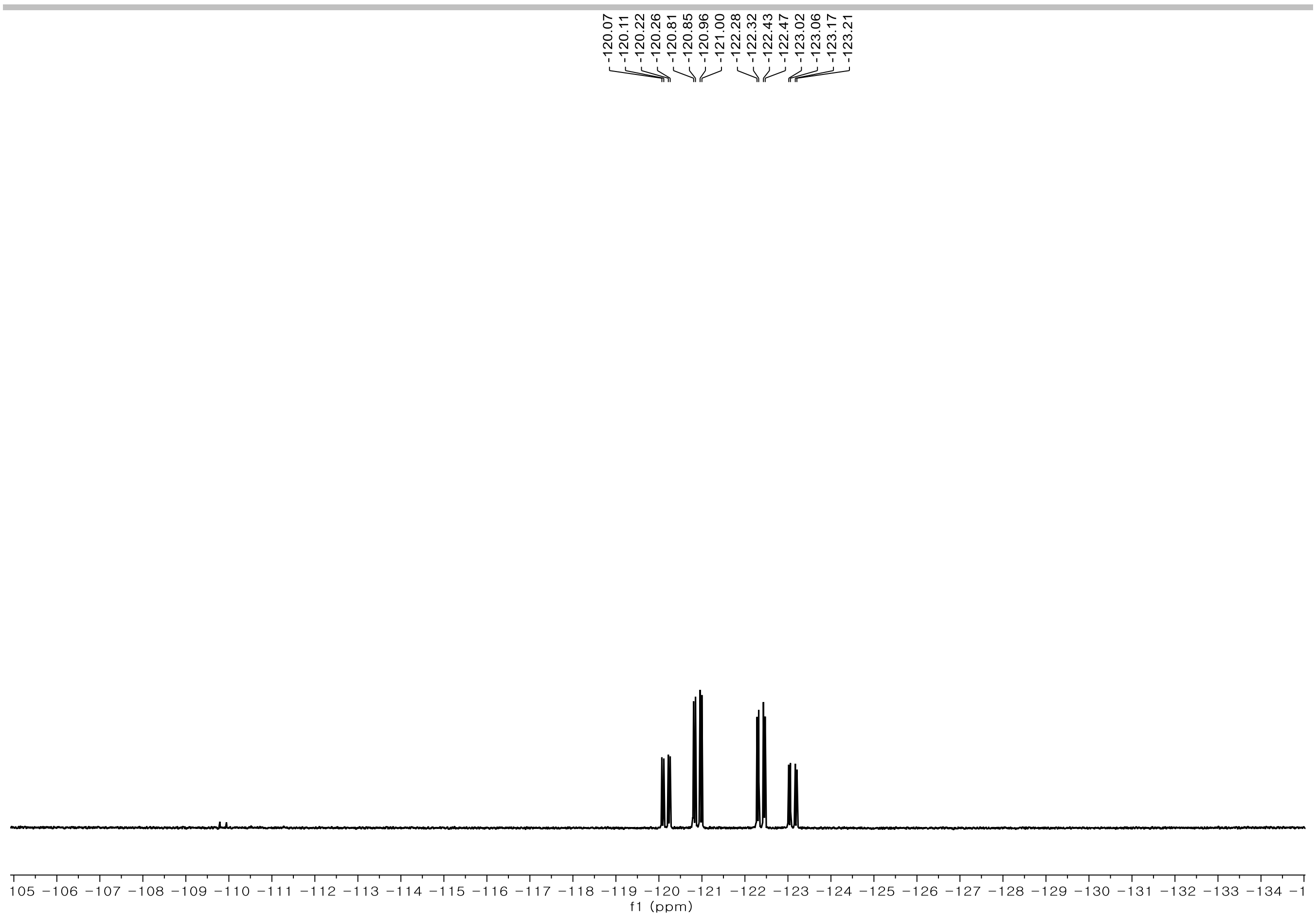

Supplementary Figure 52. ^{19}F NMR Spectrum of (3,3-difluoropropane-1,2-diyl)dibenzene (**10n**)

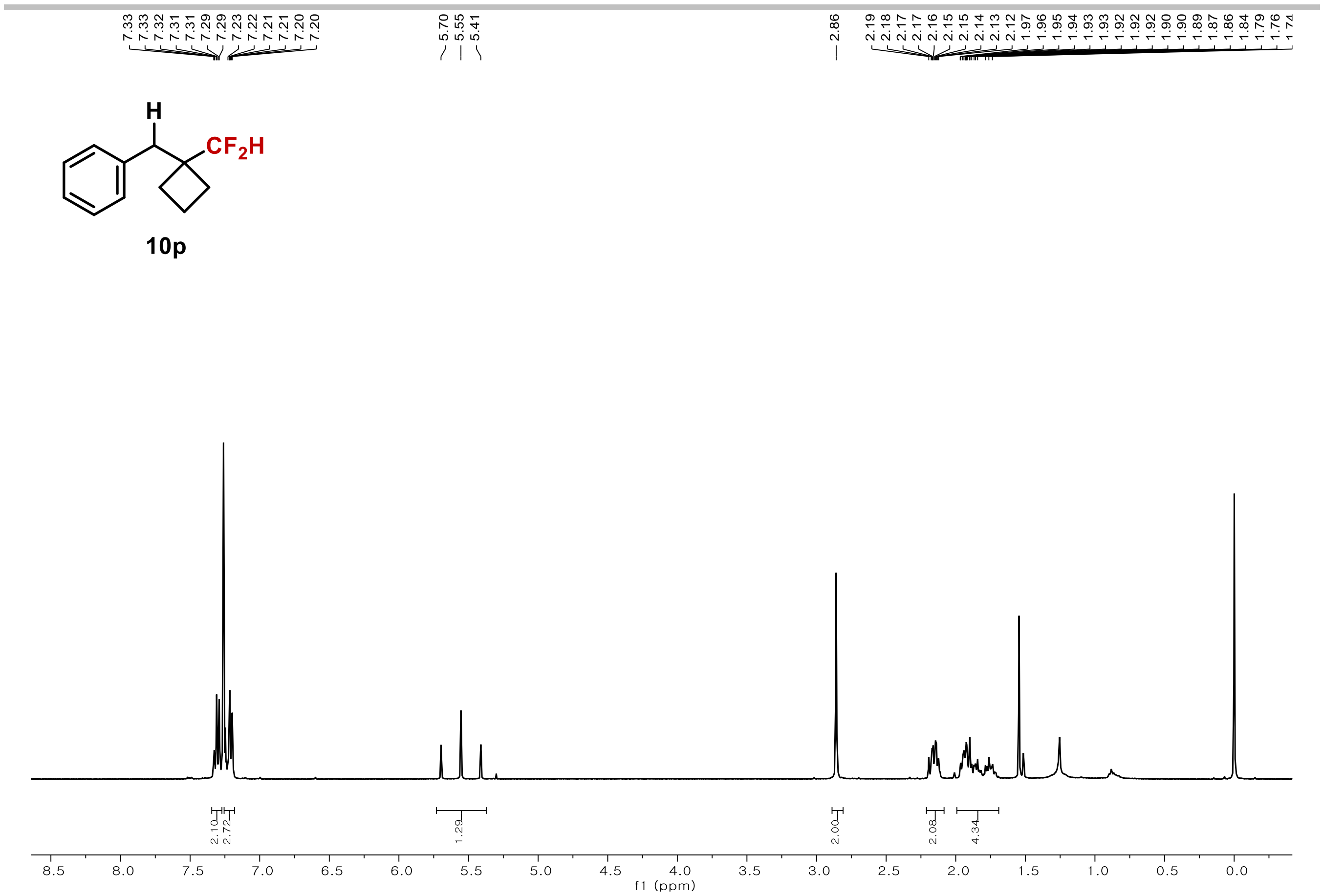

Supplementary Figure 53. ^{1}H NMR Spectrum of ((1-(Difluoromethyl)cyclobutyl)methyl)benzene (**10p**)

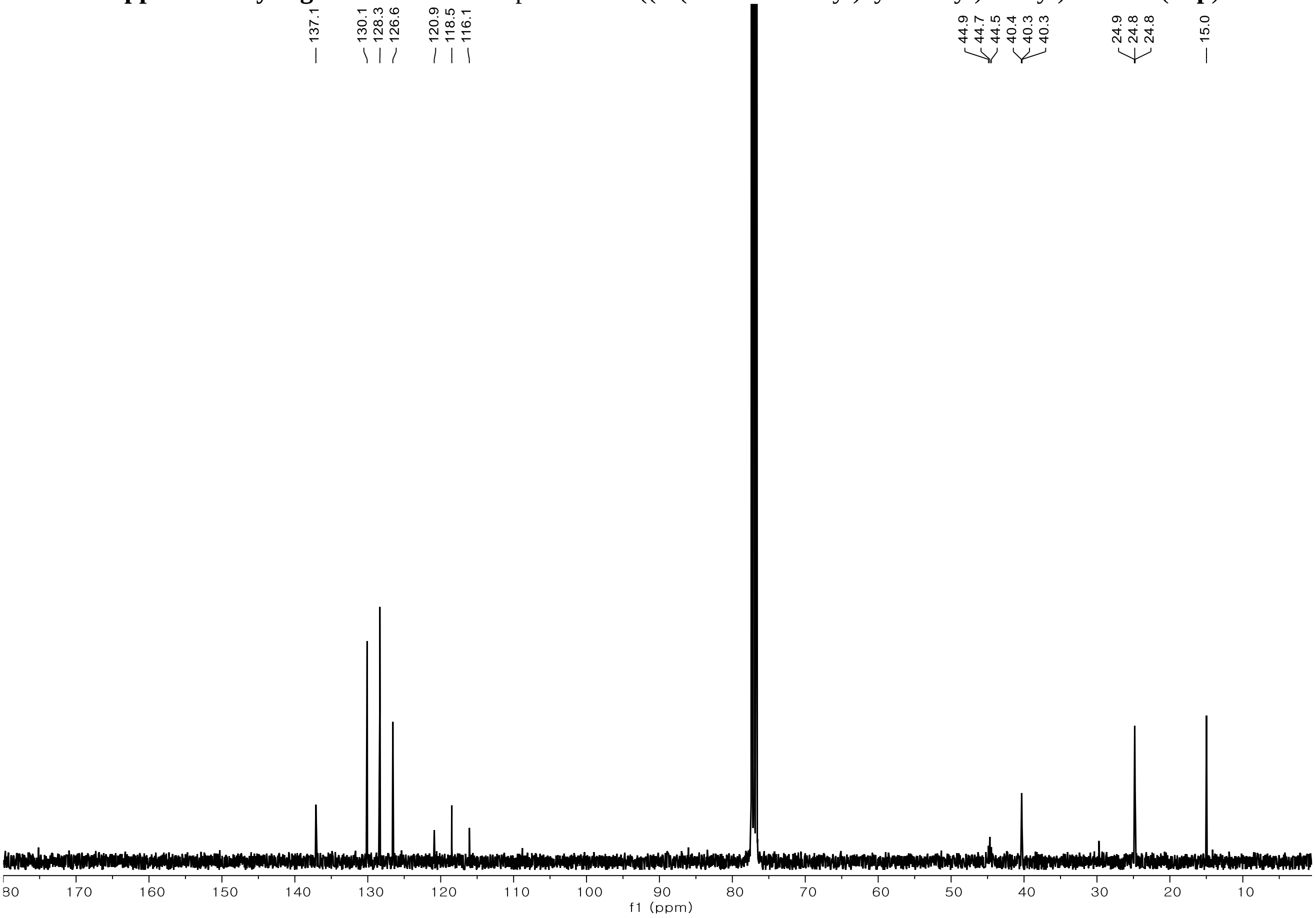

Supplementary Figure 54. ^{13}C NMR Spectrum of ((1-(Difluoromethyl)cyclobutyl)methyl)benzene (**10p**)

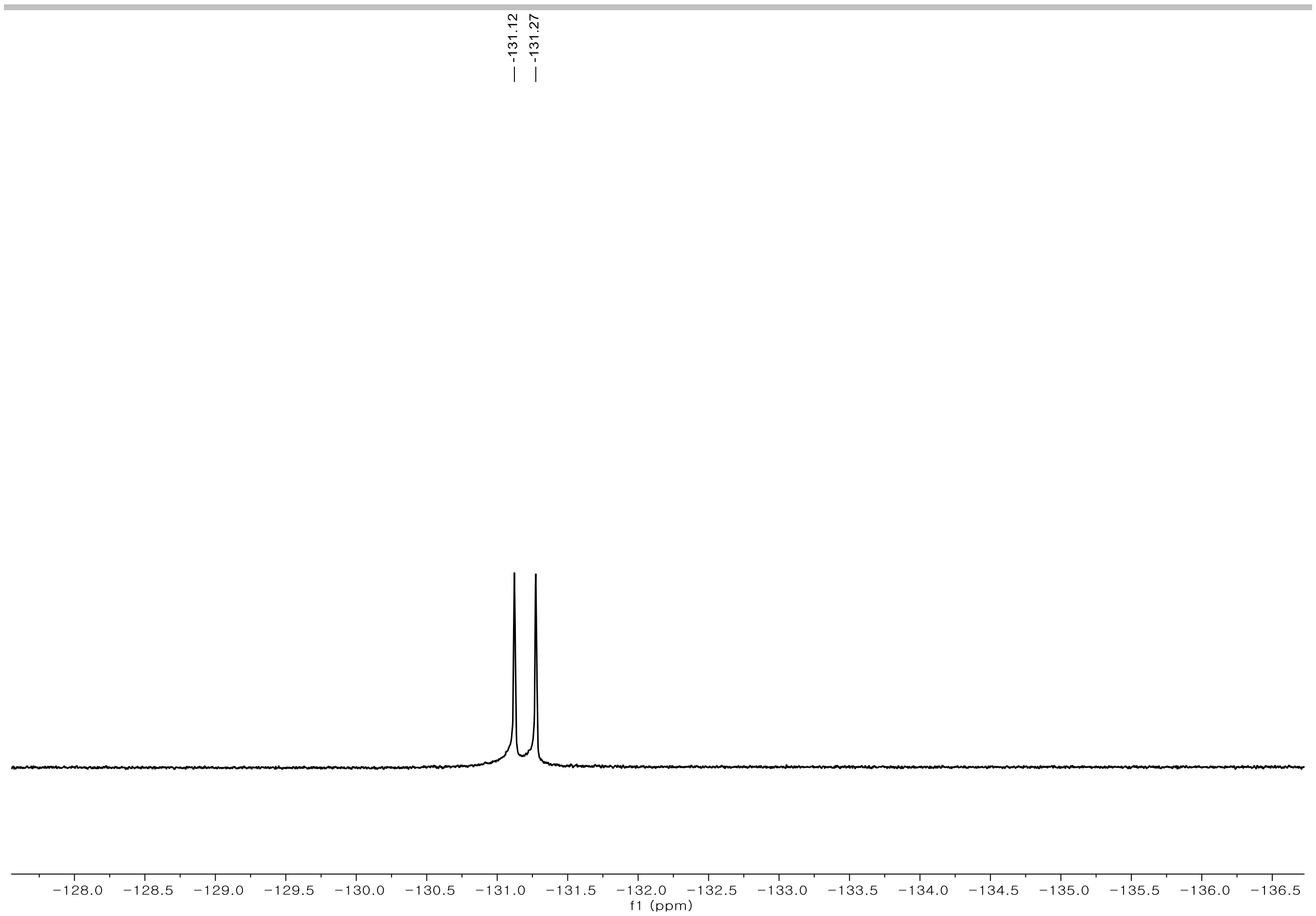

Supplementary Figure 55. ^{19}F NMR Spectrum of ((1-(Difluoromethyl)cyclobutyl)methyl)benzene (**10p**)

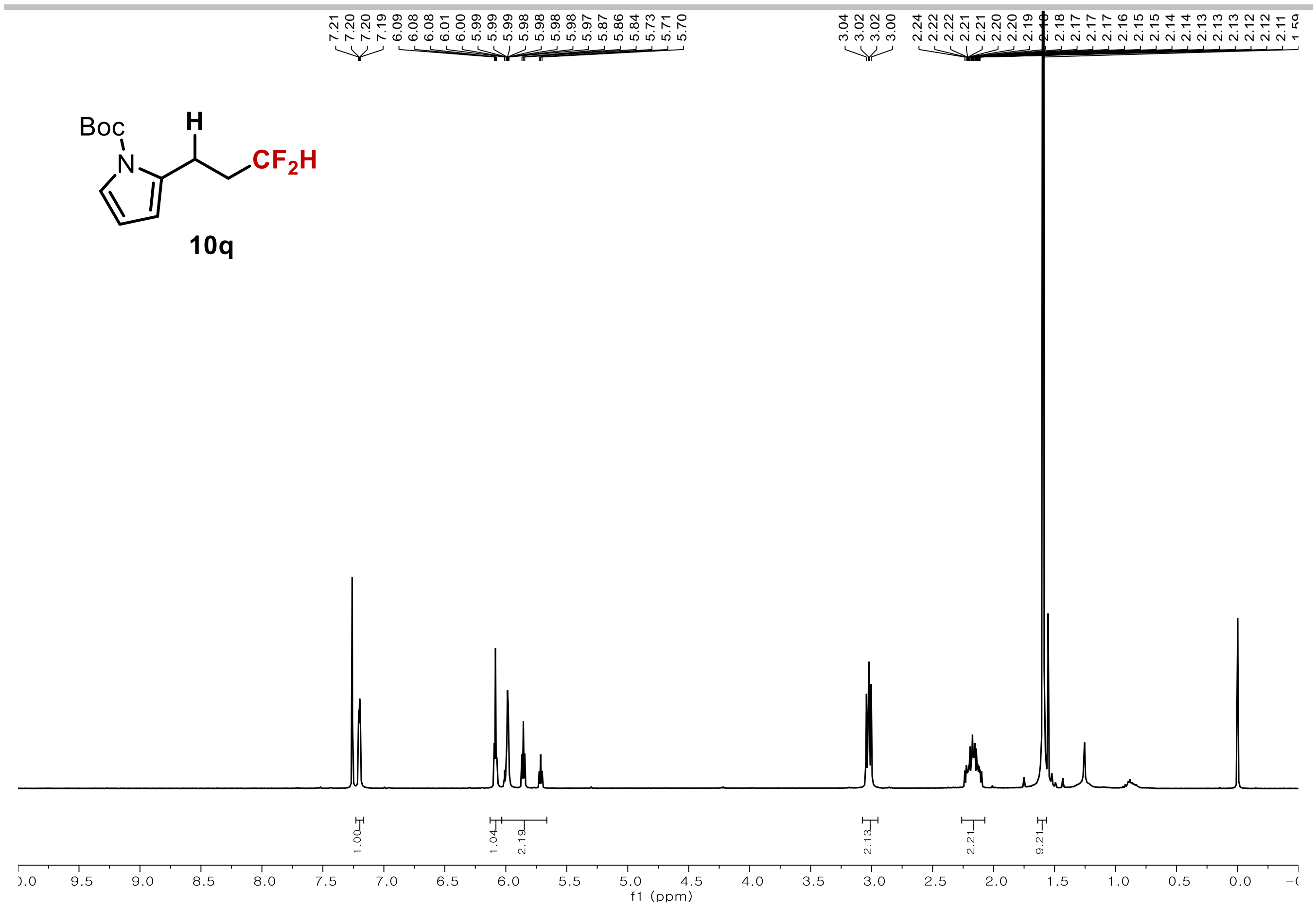

Supplementary Figure 56. ^{1}H NMR Spectrum of *tert*-Butyl 2-(3,3-difluoropropyl)-1*H*-pyrrole-1-carboxylate (**10q**)

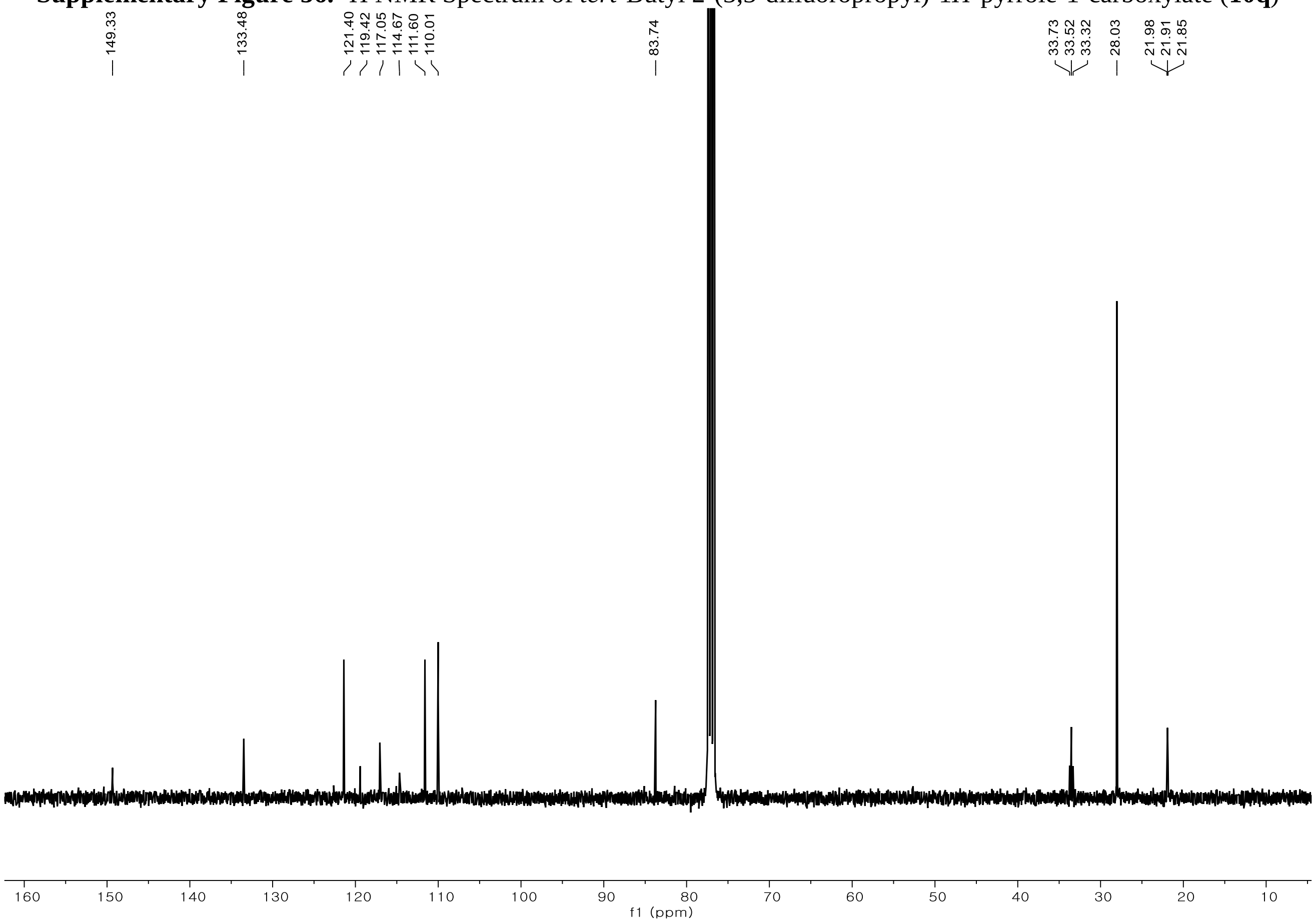

Supplementary Figure 57. ^{13}C NMR Spectrum of *tert*-Butyl 2-(3,3-difluoropropyl)-1*H*-pyrrole-1-carboxylate (**10q**)

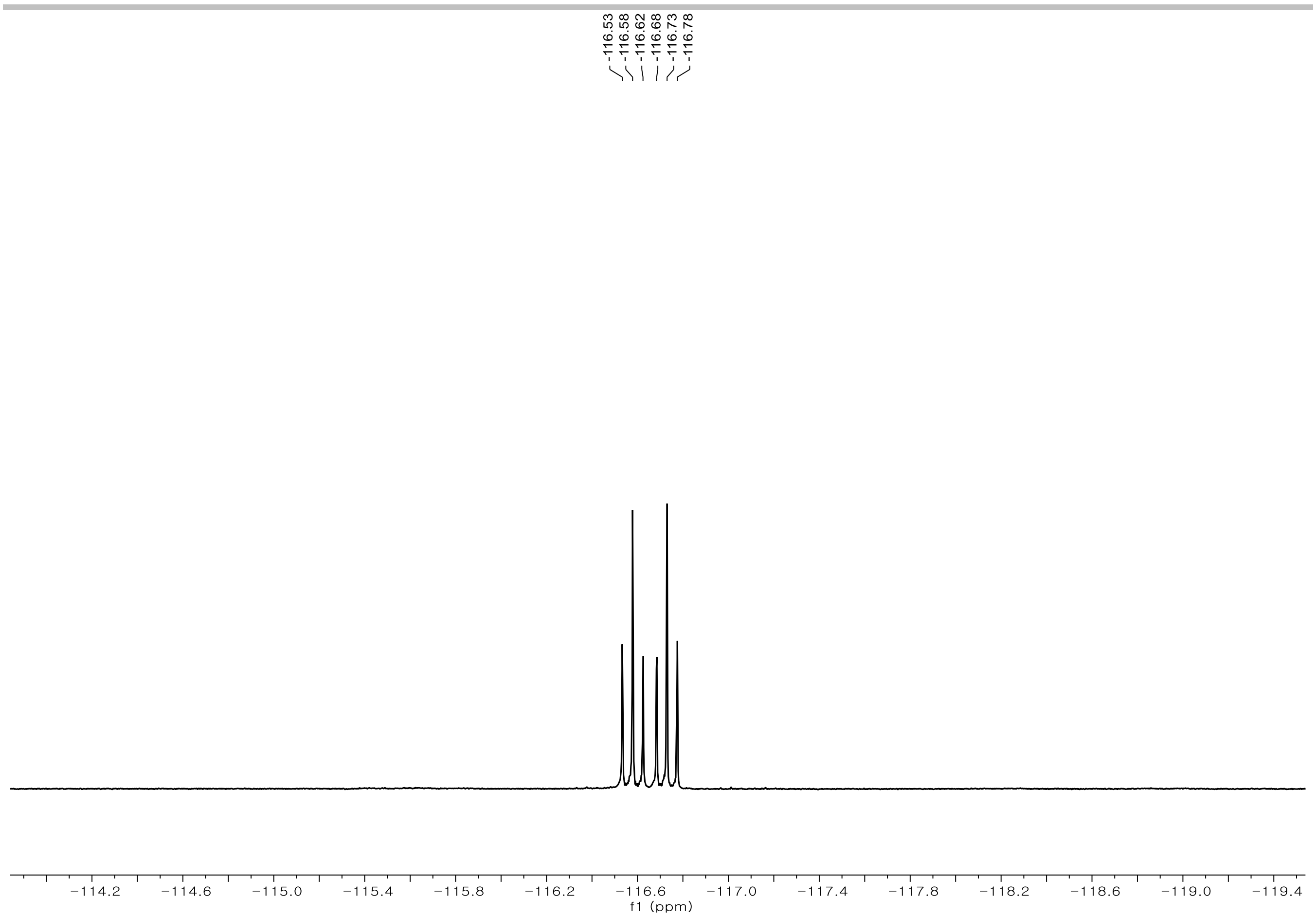

Supplementary Figure 58. ^{19}F NMR Spectrum of *tert*-Butyl 2-(3,3-difluoropropyl)-1*H*-pyrrole-1-carboxylate (**10q**)

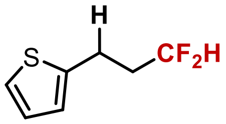

10r

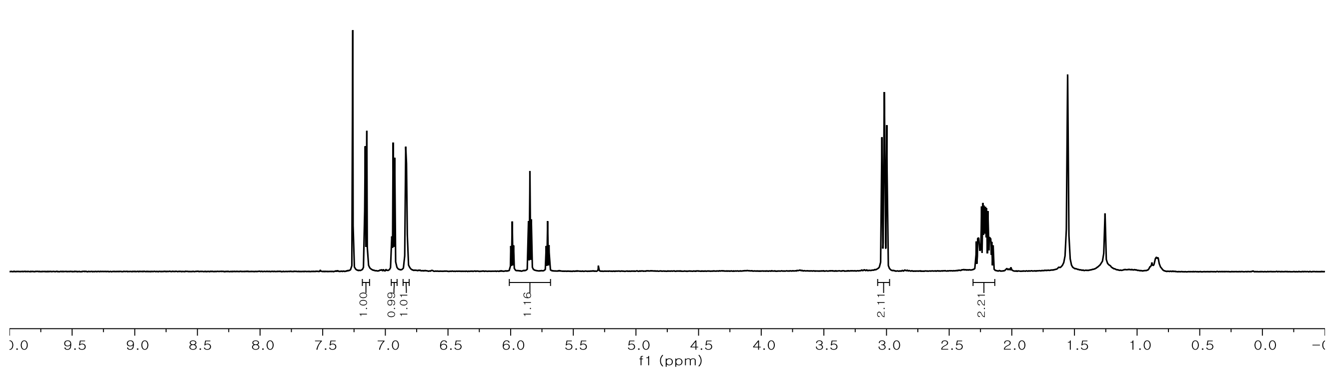

Supplementary Figure 59. ^{1}H NMR Spectrum of 2-(3,3-Difluoropropyl)thiophene (**10r**)

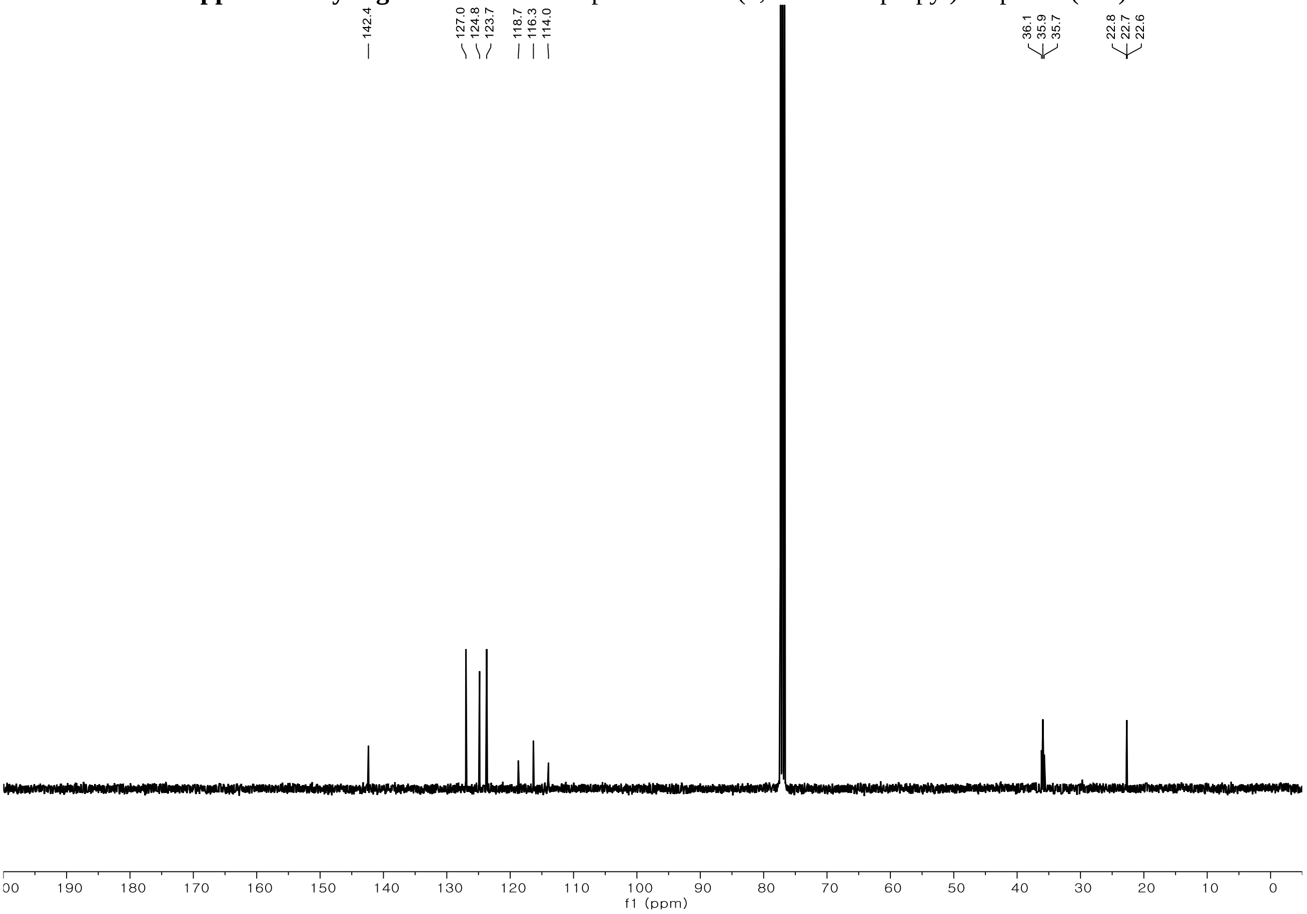

Supplementary Figure 60. ^{13}C NMR Spectrum of 2-(3,3-Difluoropropyl)thiophene (**10r**)

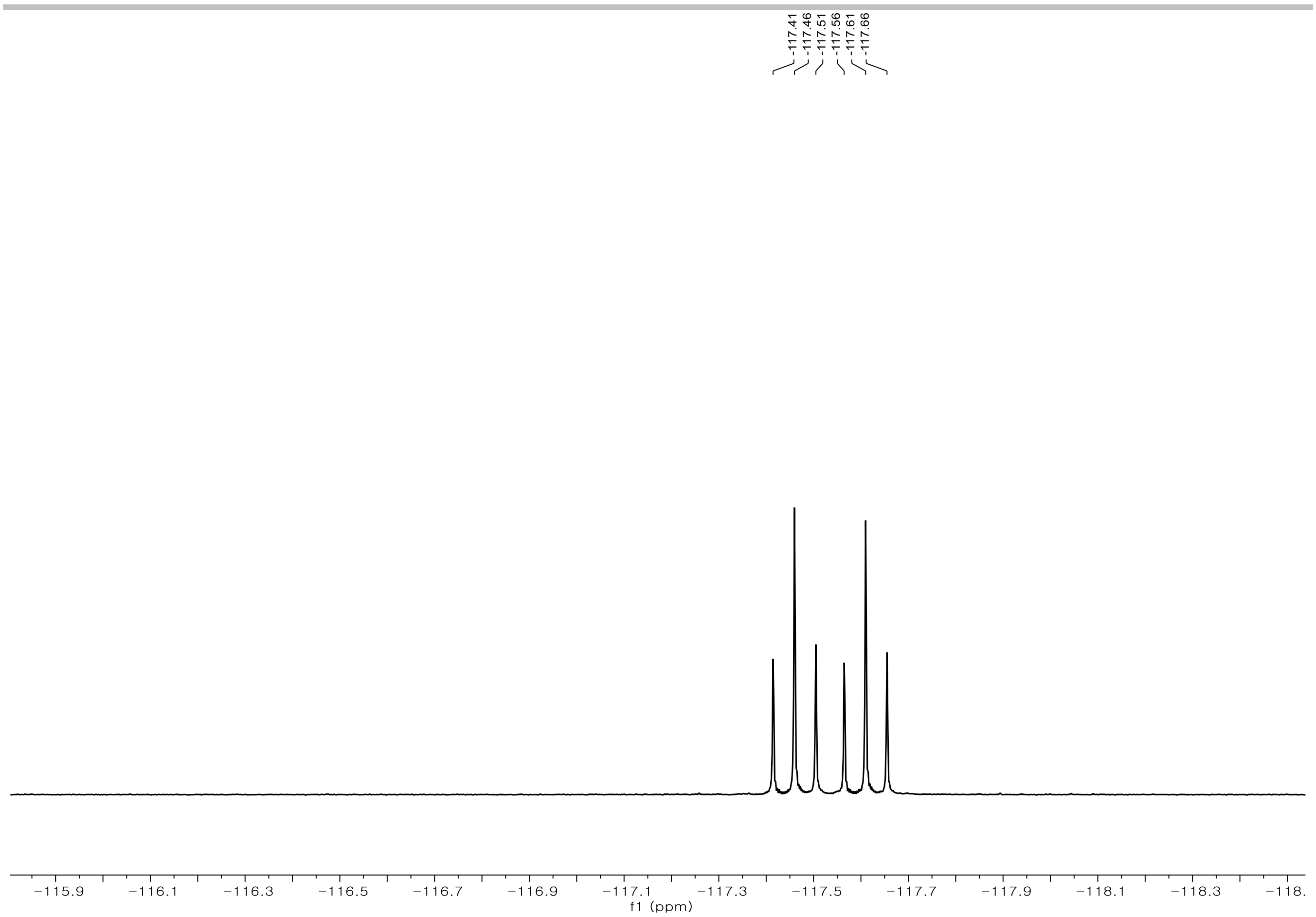

Supplementary Figure 61. ^{19}F NMR Spectrum of 2-(3,3-Difluoropropyl)thiophene (**10r**)

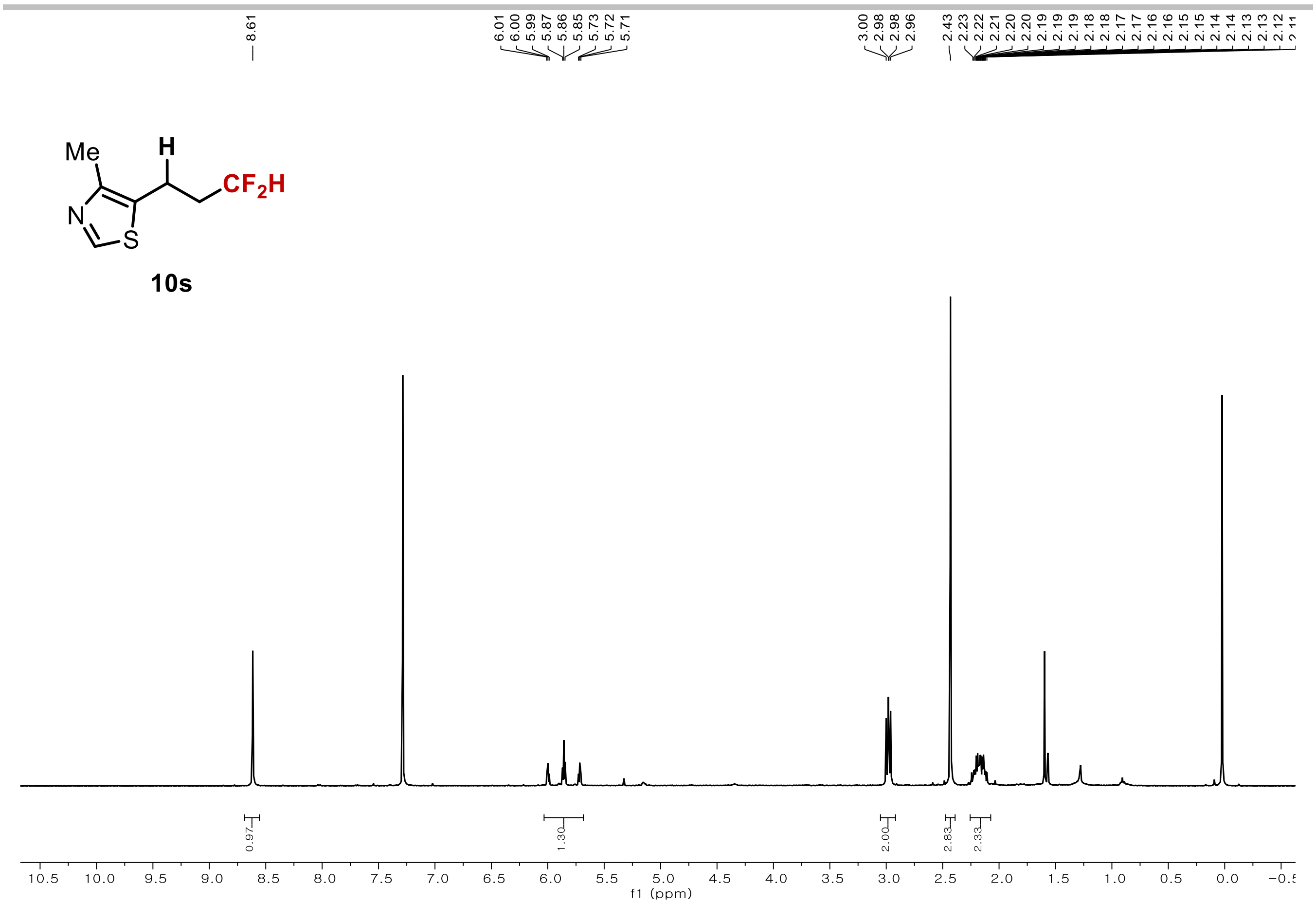

Supplementary Figure 62. ^{1}H NMR Spectrum of 5-(3,3-Difluoropropyl)-4-methylthiazole (**10s**)

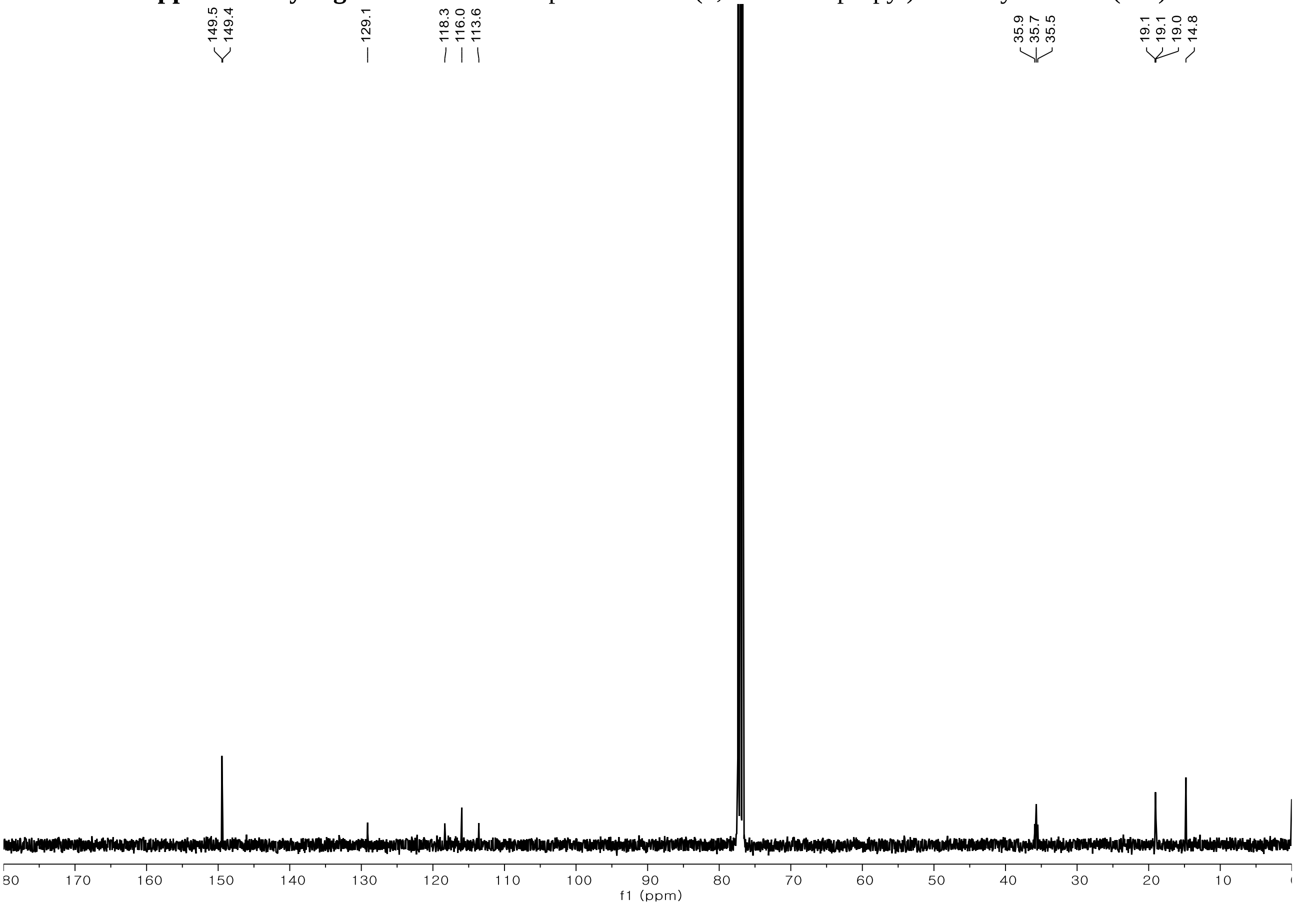

Supplementary Figure 63. ^{13}C NMR Spectrum of 5-(3,3-Difluoropropyl)-4-methylthiazole (**10s**)

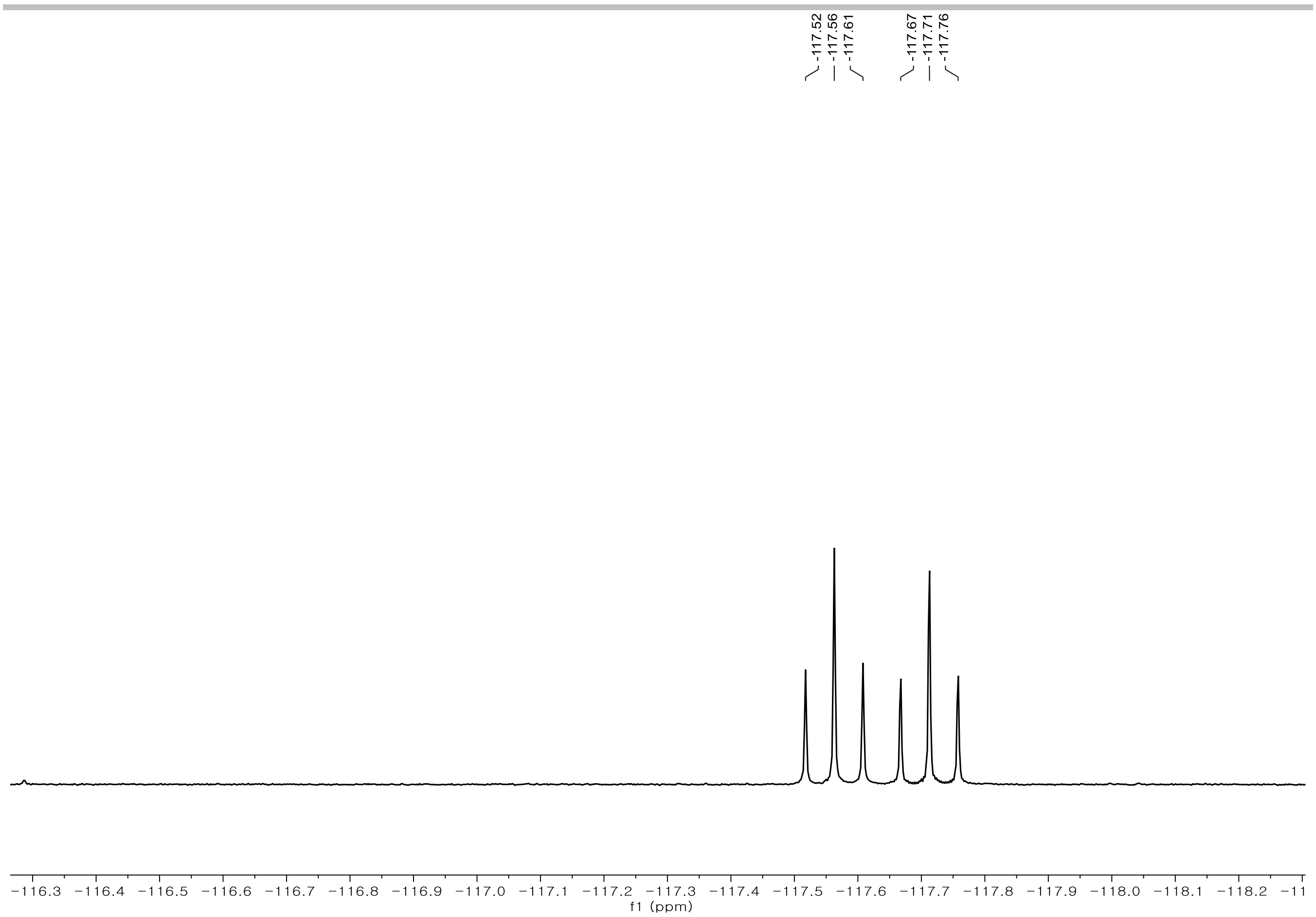

Supplementary Figure 64. ^{19}F NMR Spectrum of 5-(3,3-Difluoropropyl)-4-methylthiazole (**10s**)

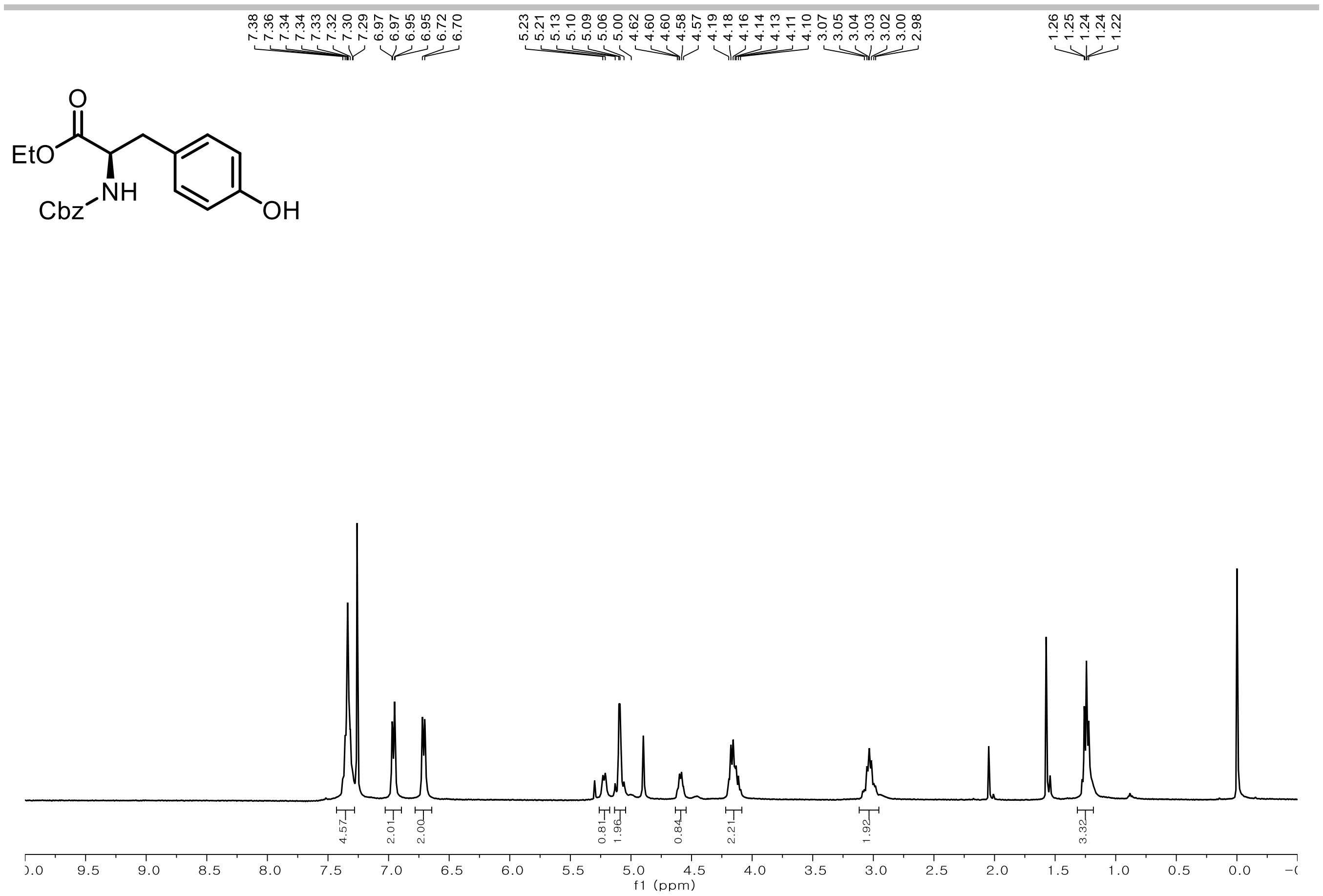

Supplementary Figure 65. ^{1}H NMR Spectrum of Ethyl ((benzyloxy)carbonyl)-D-tyrosinate

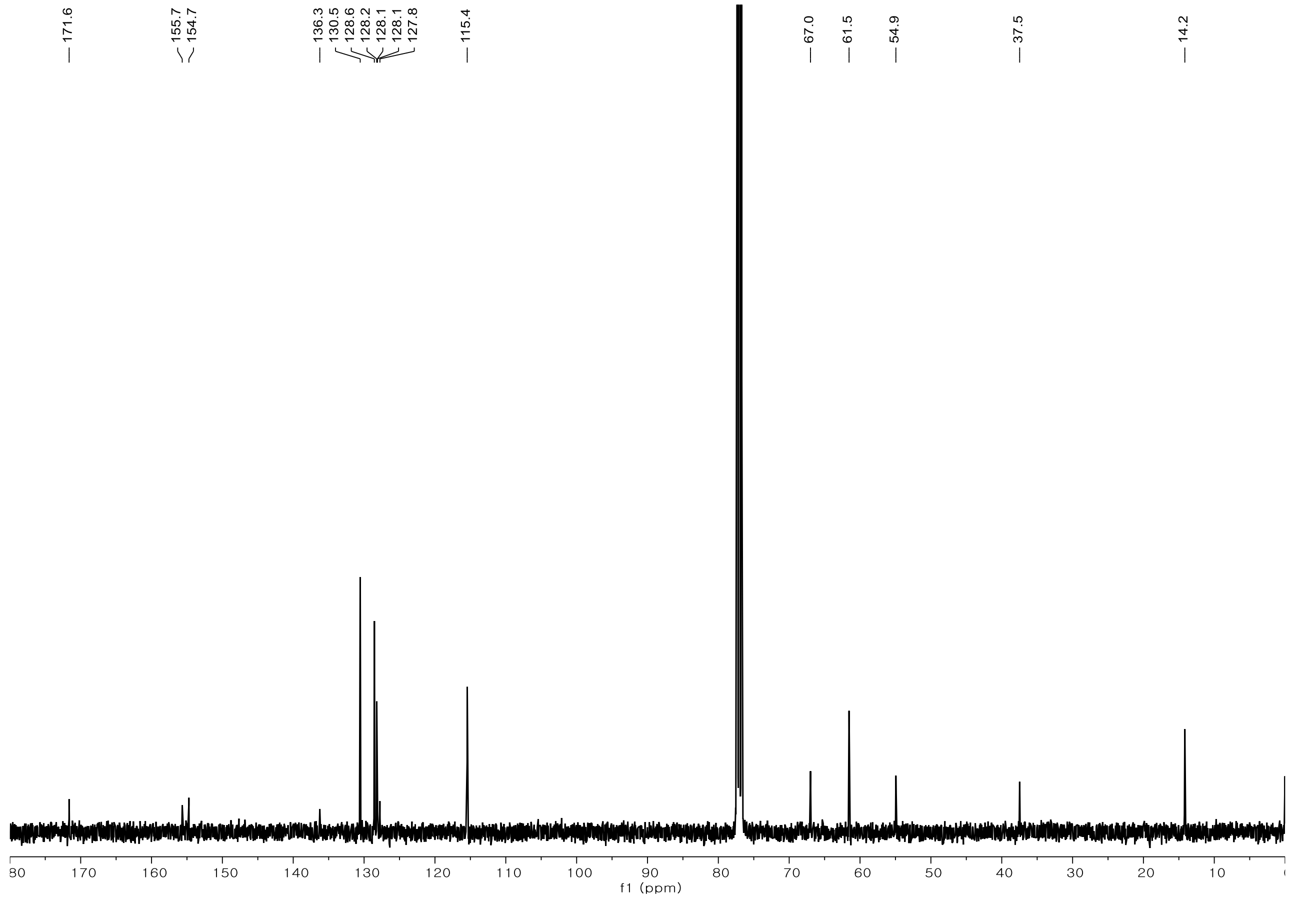

Supplementary Figure 66. ^{13}C NMR Spectrum of Ethyl ((benzyloxy)carbonyl)-D-tyrosinate

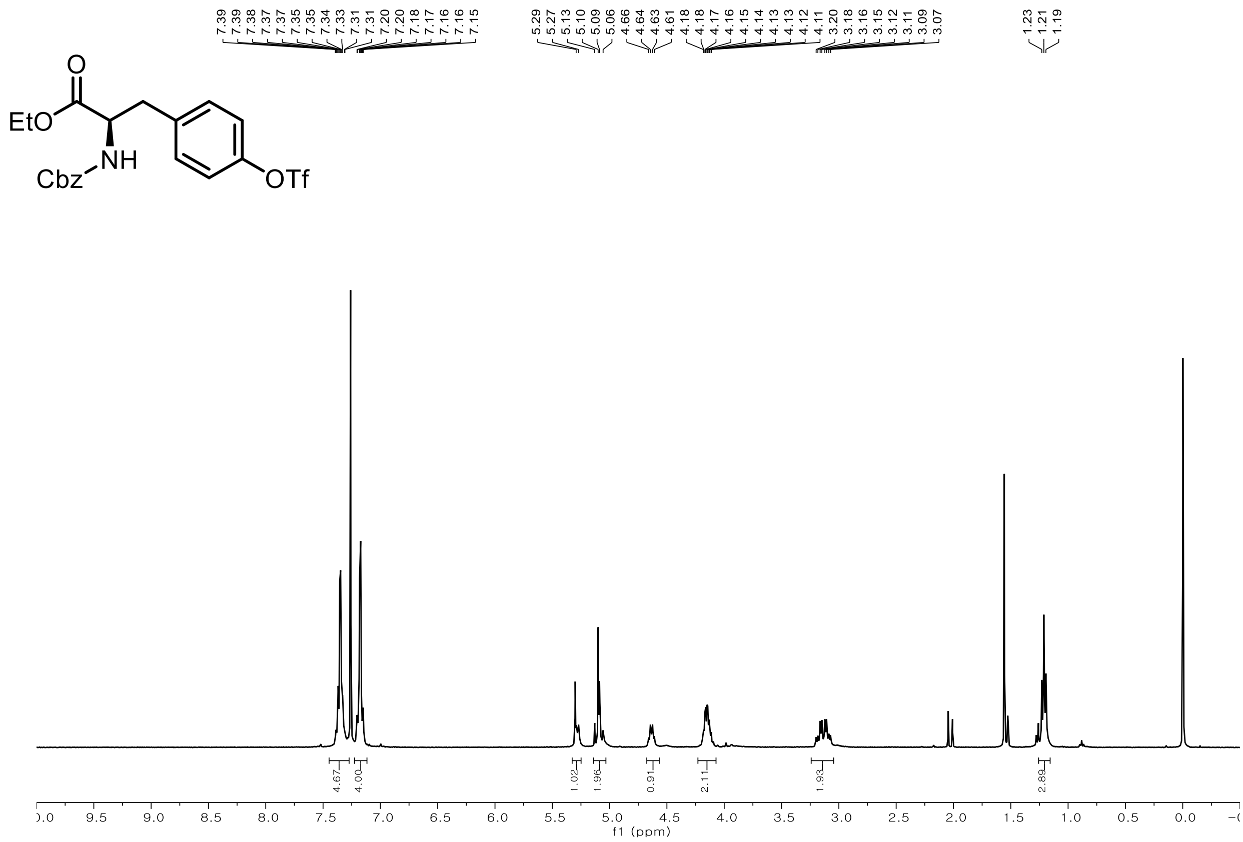

Supplementary Figure 67. ^{1}H NMR Spectrum of Ethyl (R)-2-(((benzyloxy)carbonyl)amino)-3-(4-(((trifluoromethyl)sulfonyl)oxy)phenyl)propanoate

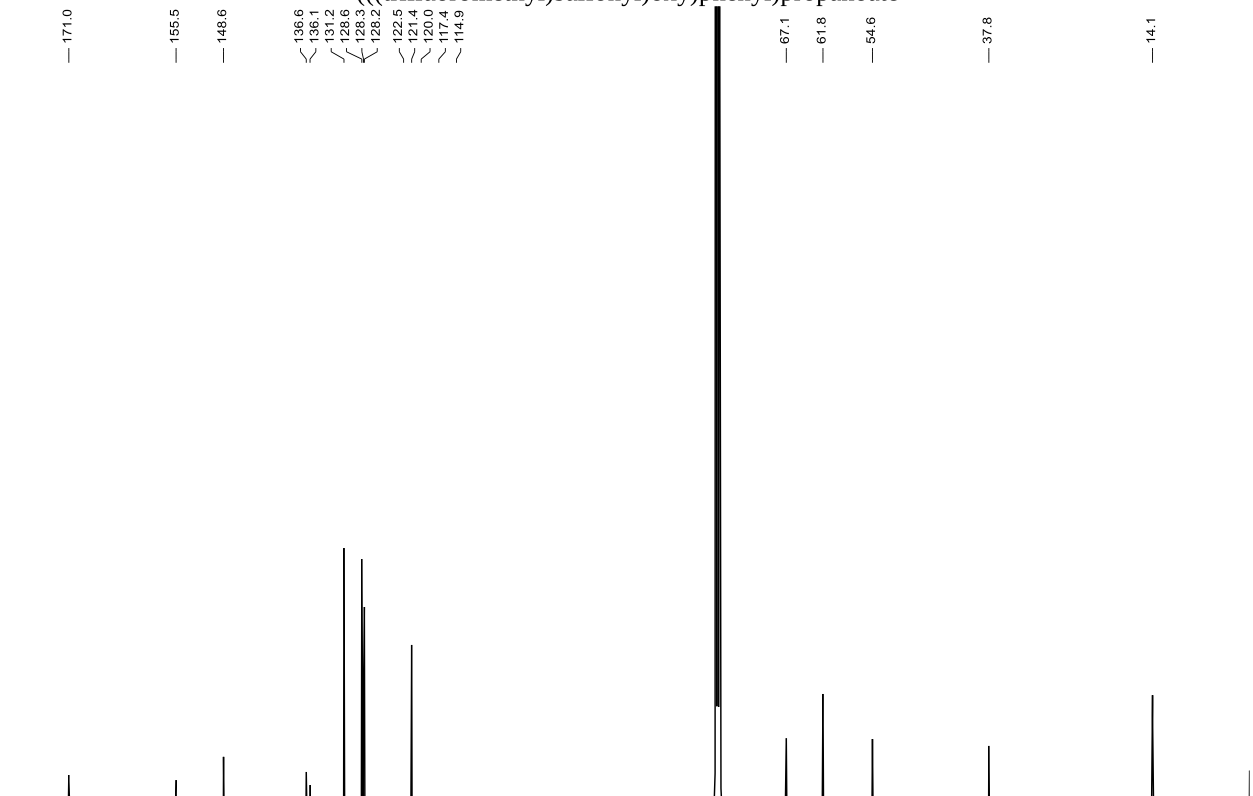

Supplementary Figure 68. ^{13}C NMR Spectrum of Ethyl (R)-2-(((benzyloxy)carbonyl)amino)-3-(4-(((trifluoromethyl)sulfonyl)oxy)phenyl)propanoate

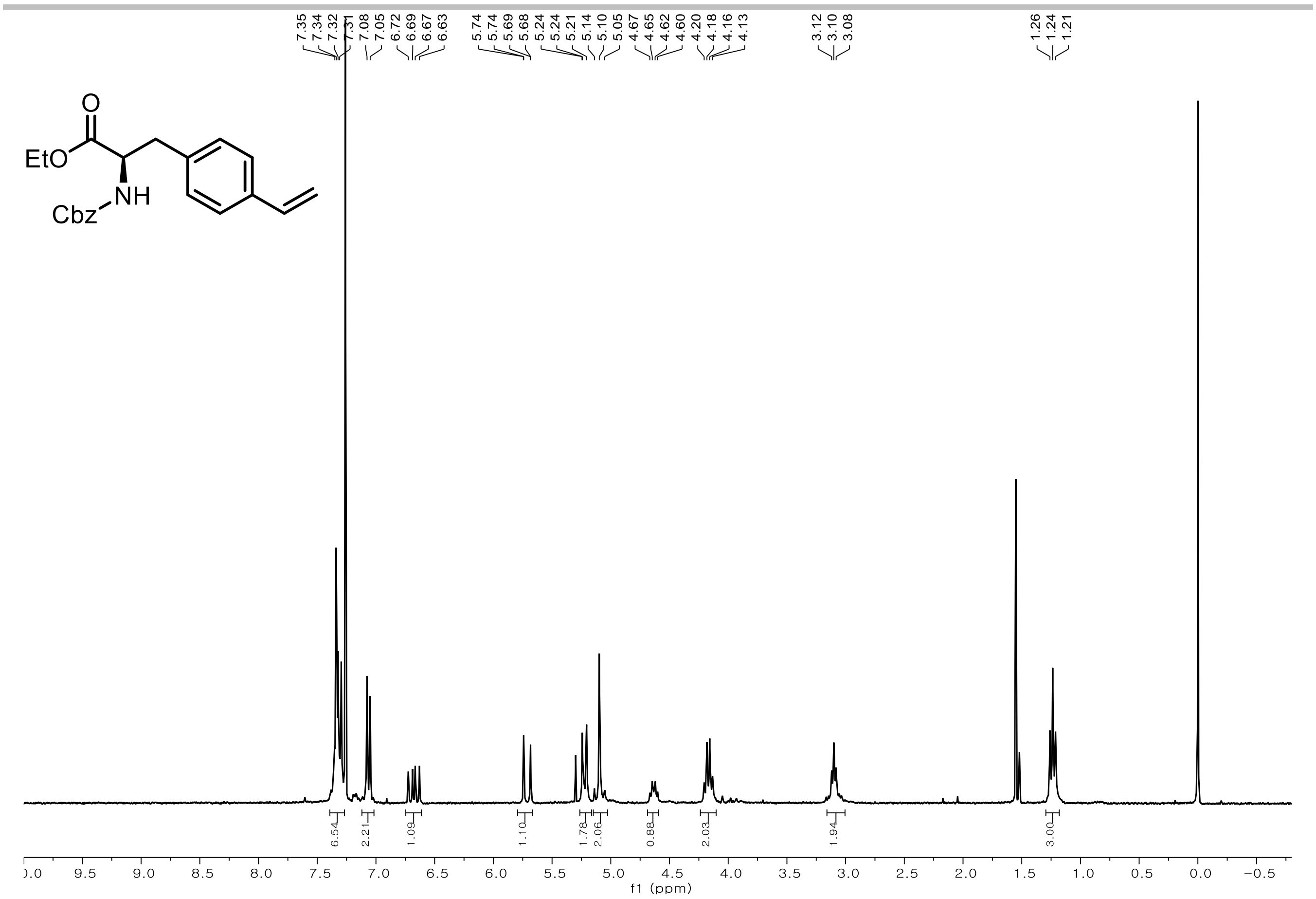

Supplementary Figure 69. ^{1}H NMR Spectrum of Ethyl (R)-2-(((benzyloxy)carbonyl)amino)-3-(4-vinylphenyl)propanoate

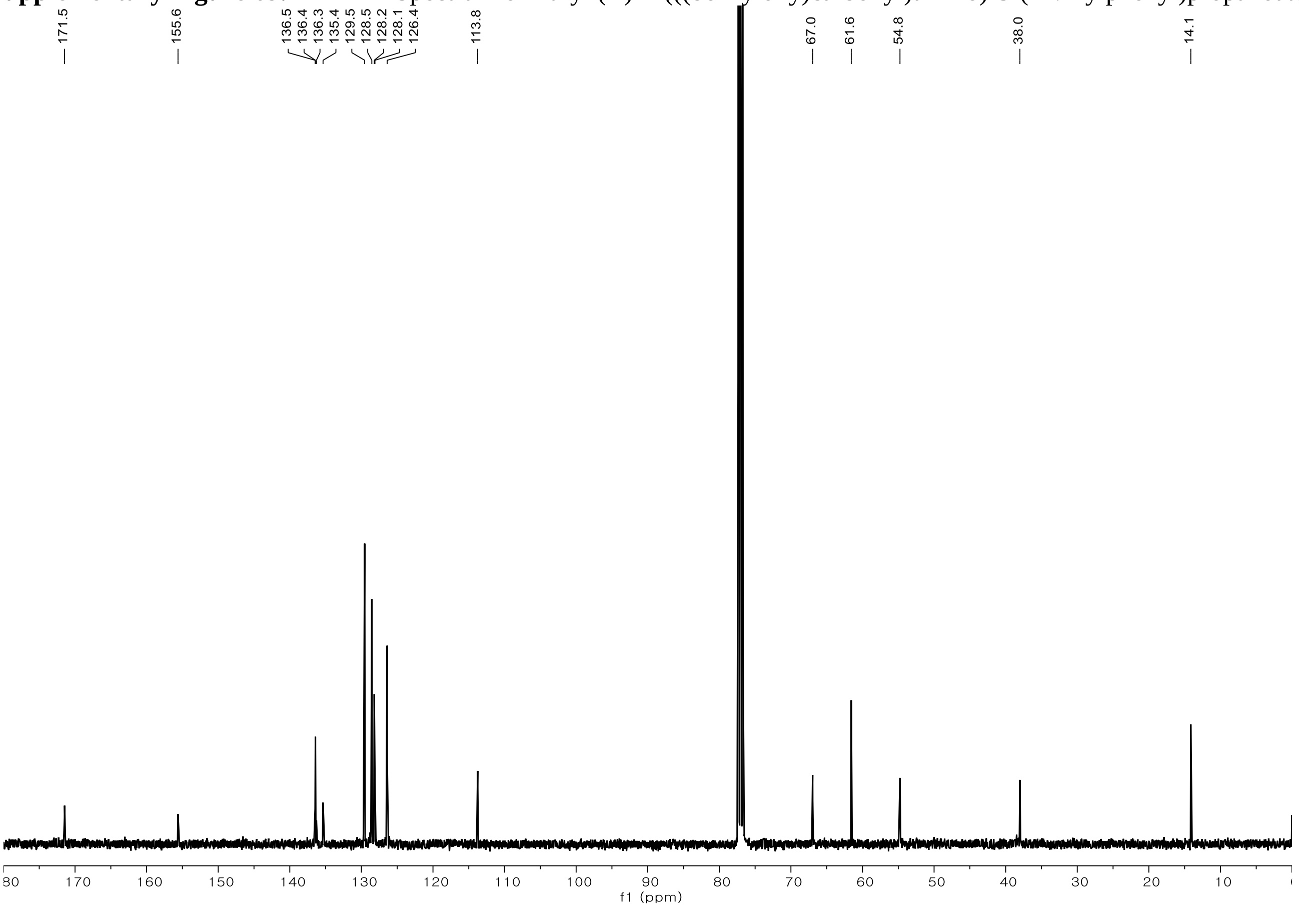

Supplementary Figure 70. ^{13}C NMR Spectrum of Ethyl (R)-2-(((benzyloxy)carbonyl)amino)-3-(4-vinylphenyl)propanoate

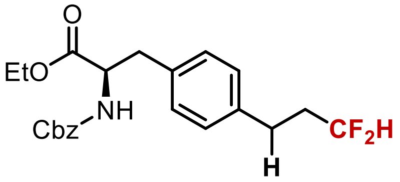

10t

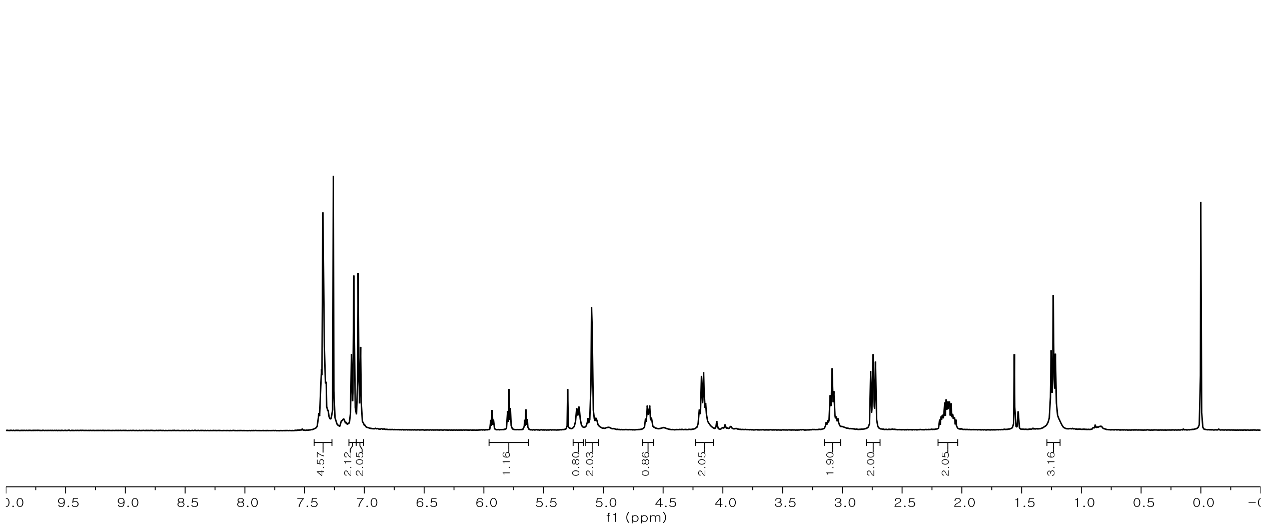

Supplementary Figure 71. ^{1}H NMR Spectrum of Ethyl (R)-2-(((benzyloxy)carbonyl)amino)-3-(4-(3,3-difluoropropyl)phenyl)propanoate (**10t**)

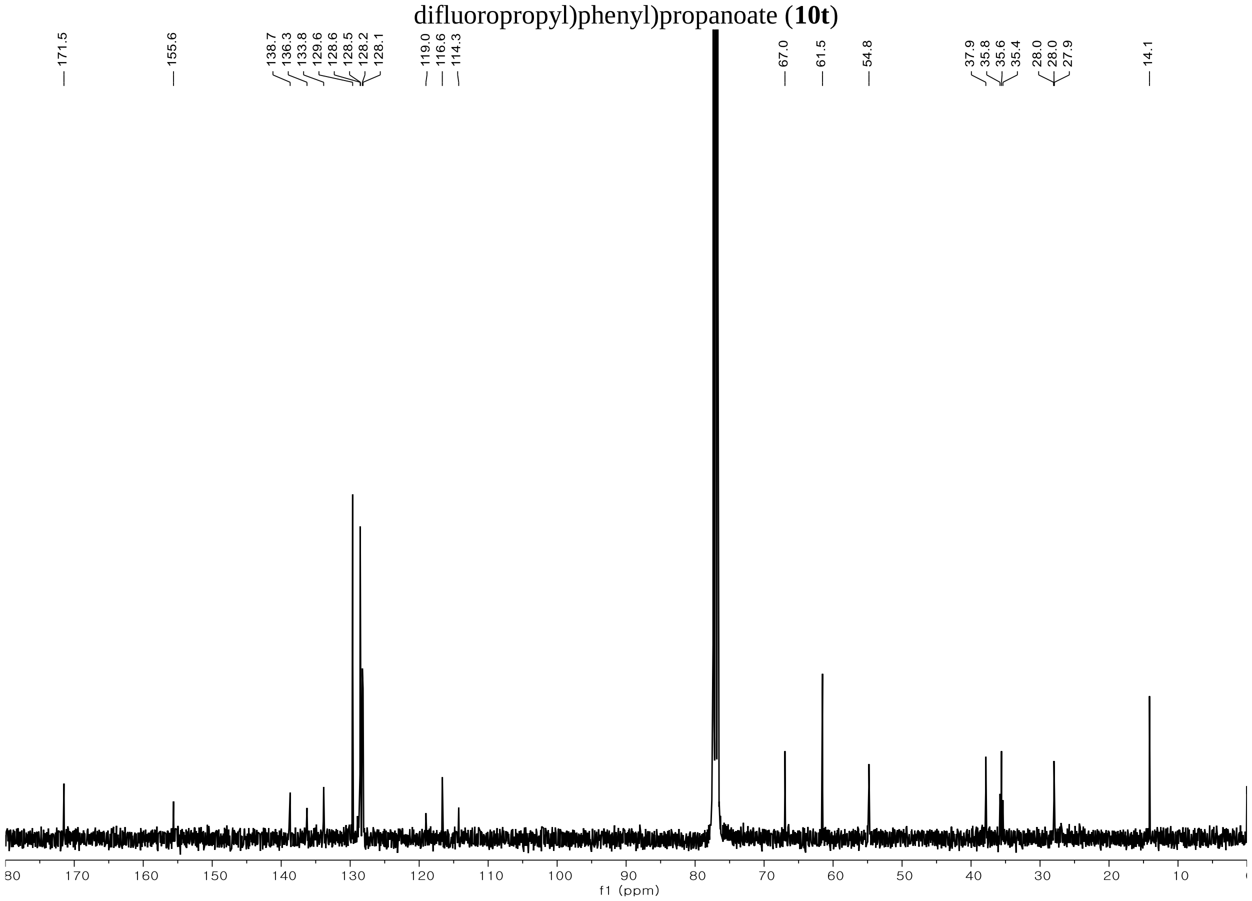

Supplementary Figure 72. ^{13}C NMR Spectrum of Ethyl (R)-2-(((benzyloxy)carbonyl)amino)-3-(4-(3,3-difluoropropyl)phenyl)propanoate (**10t**)

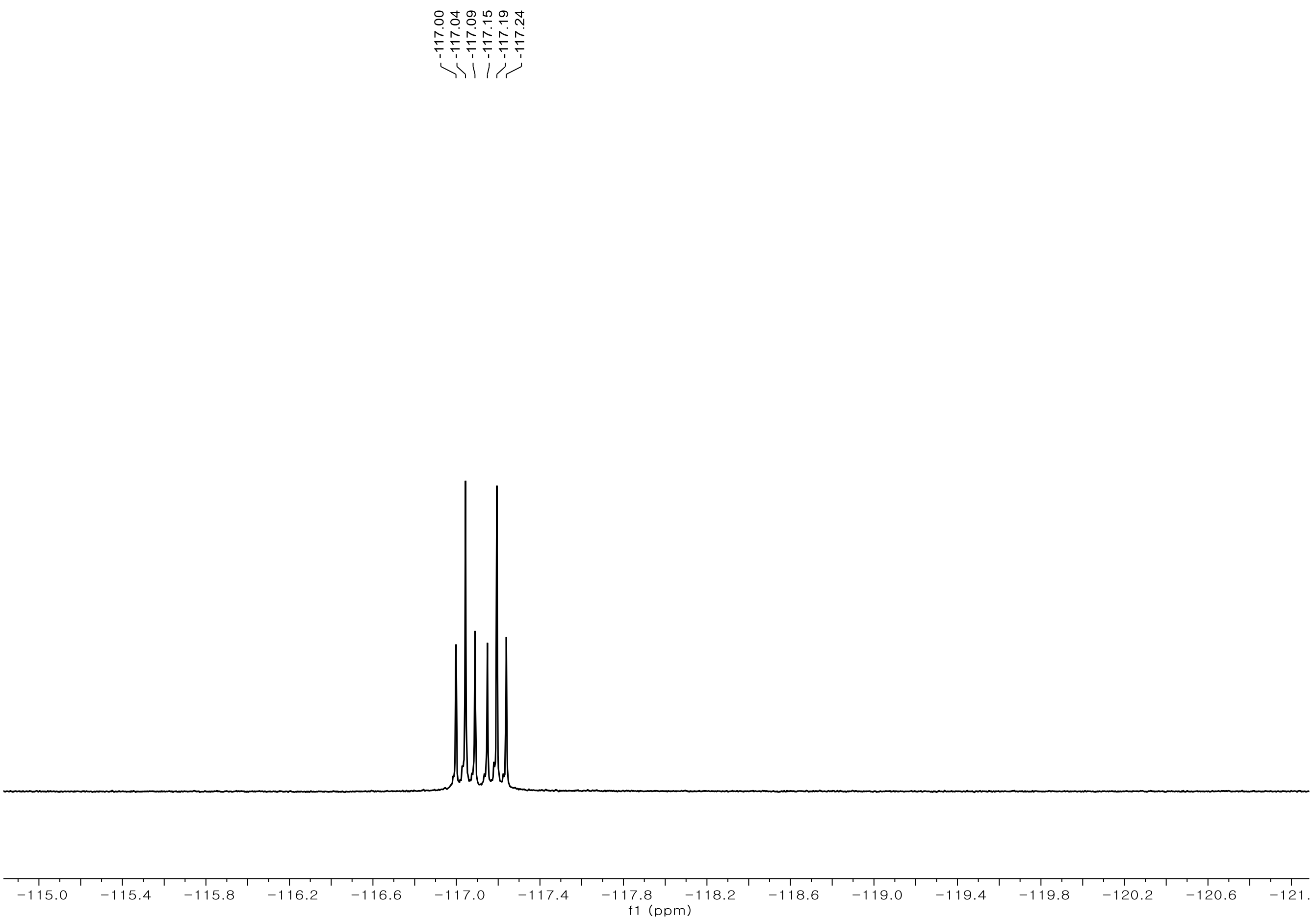

Supplementary Figure 73. ^{19}F NMR Spectrum of Ethyl (R)-2-(((benzyloxy)carbonyl)amino)-3-(4-(3,3-difluoropropyl)phenyl)propanoate (**10t**)

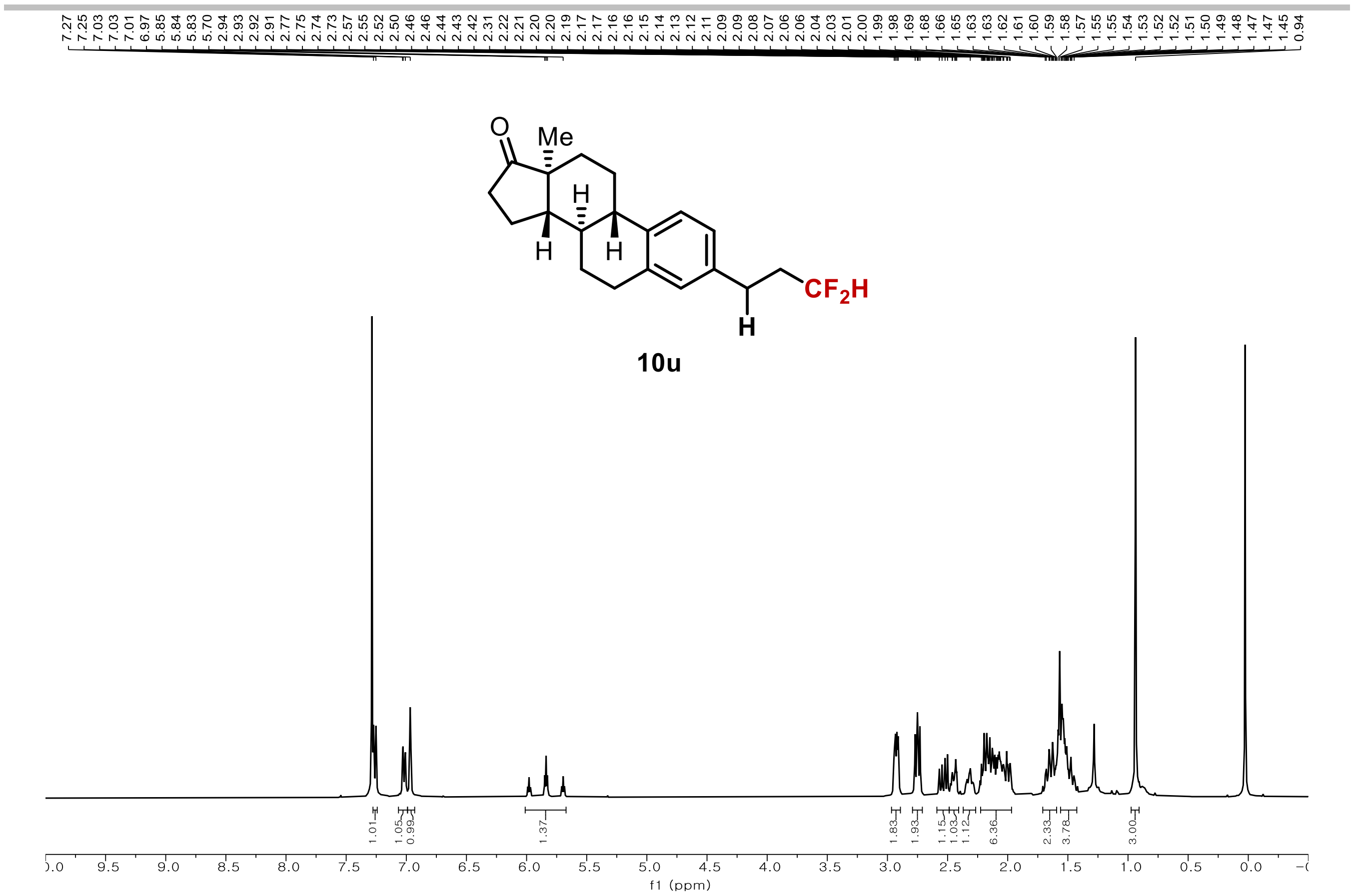

Supplementary Figure 74. ^{1}H NMR Spectrum of 3-(3,3-Difluoropropyl)-13-methyl-6,7,8,9,11,12,13,14,15,16-decahydro-17*H*-cyclopenta[a]phenanthren-17-one (**10u**)

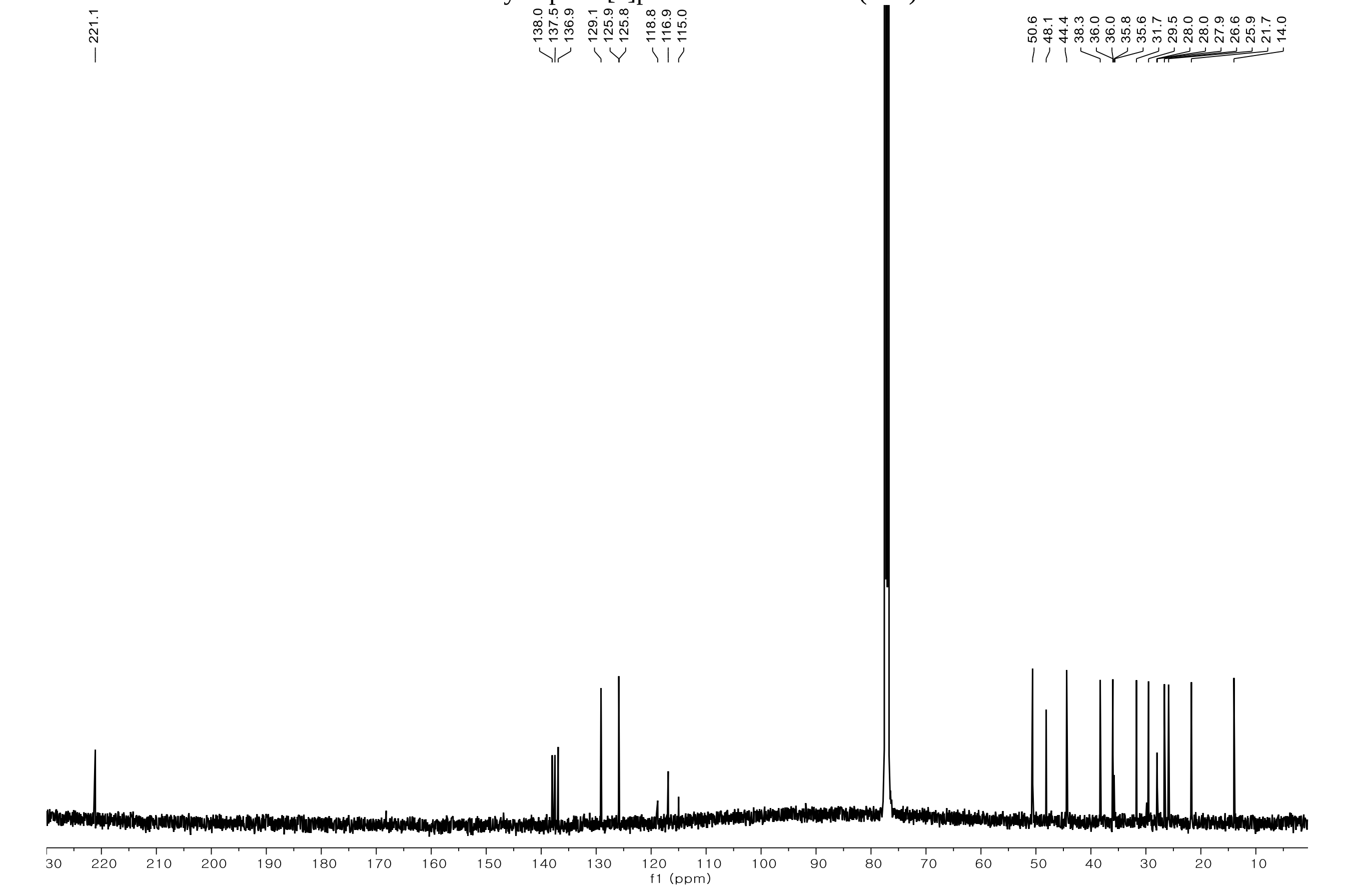

Supplementary Figure 75. ^{13}C NMR Spectrum of 3-(3,3-Difluoropropyl)-13-methyl-6,7,8,9,11,12,13,14,15,16-decahydro-17*H*-cyclopenta[a]phenanthren-17-one (**10u**)

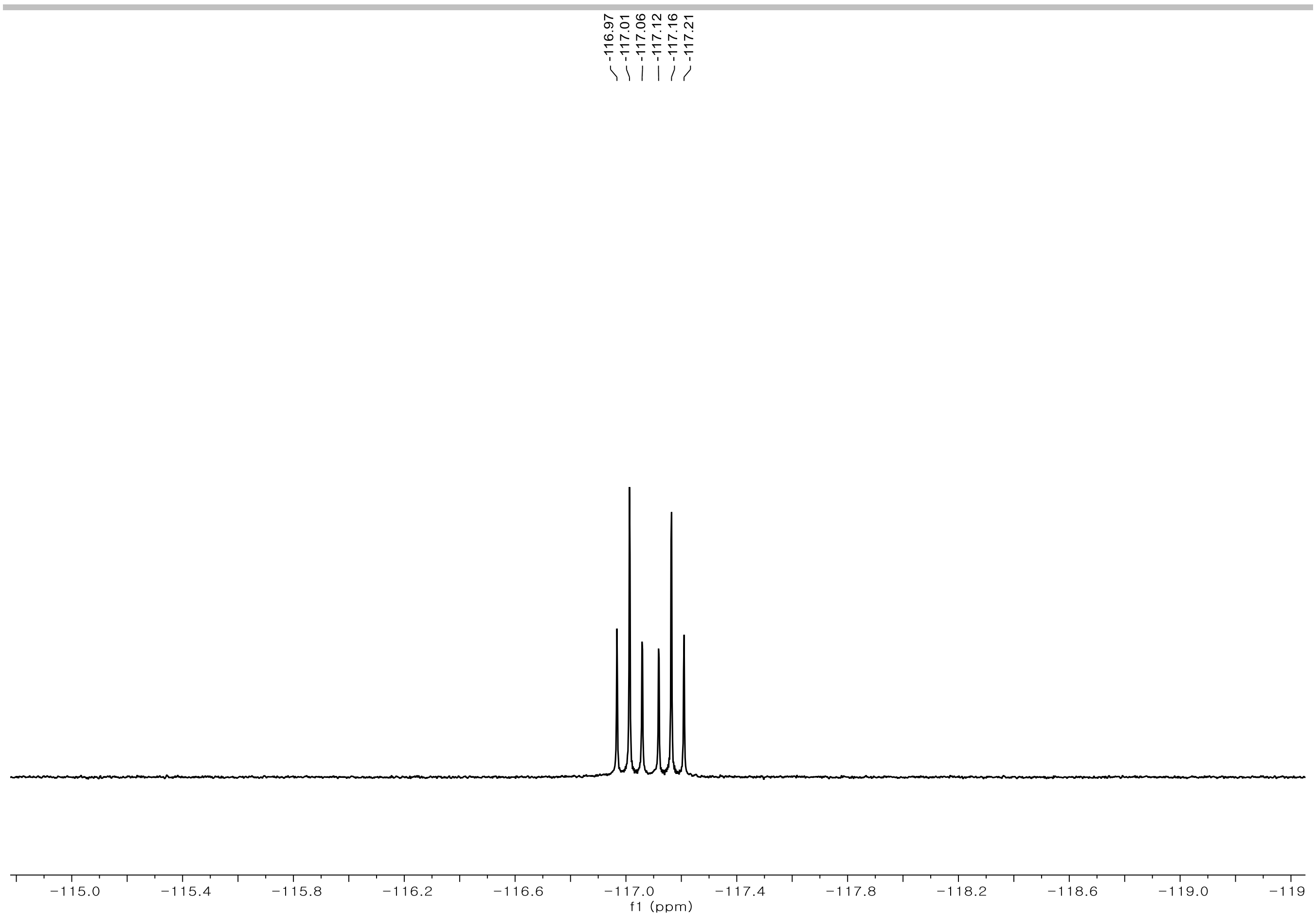

Supplementary Figure 76. ^{19}F NMR Spectrum of 3-(3,3-Difluoropropyl)-13-methyl-6,7,8,9,11,12,13,14,15,16-decahydro-17*H*-cyclopenta[a]phenanthren-17-one (**10u**)

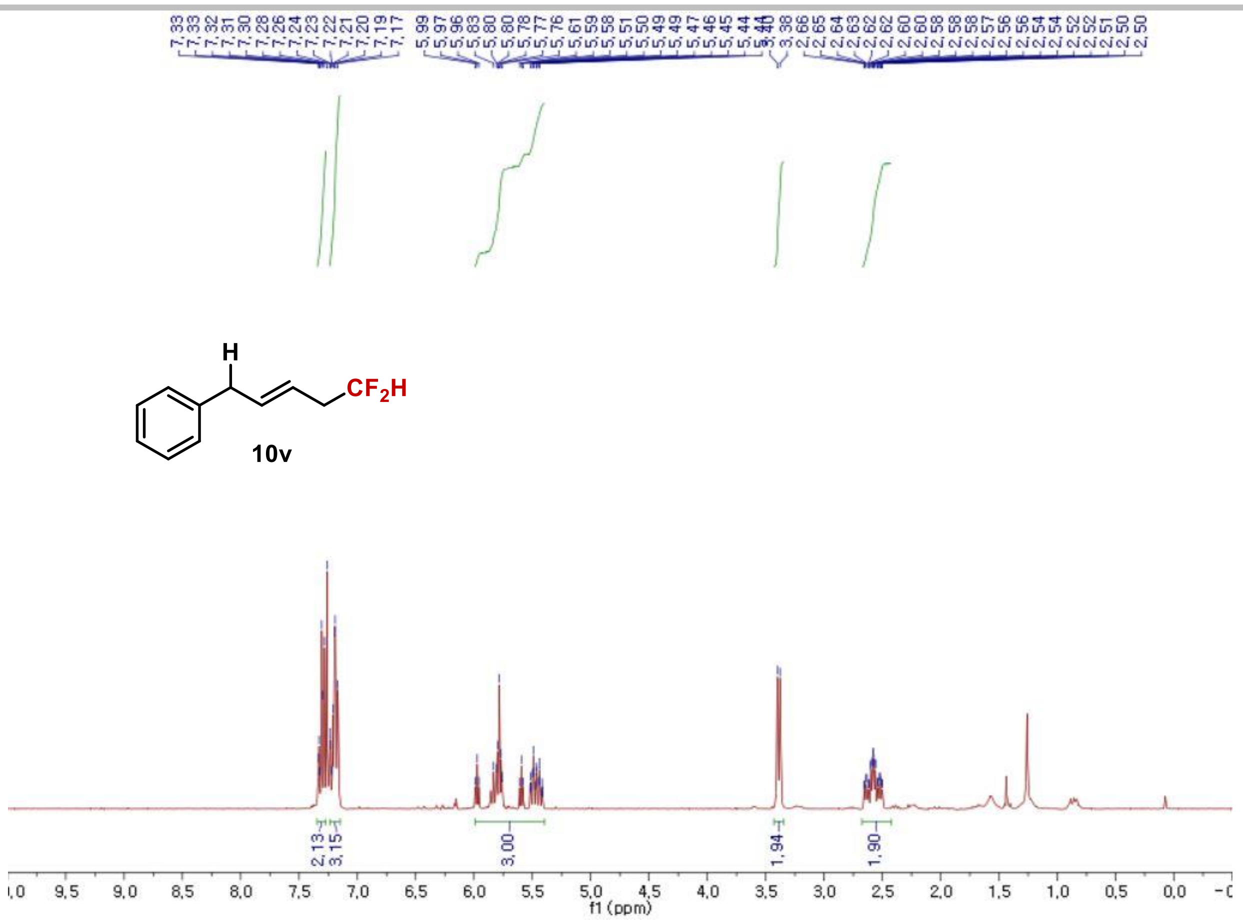

Supplementary Figure 77. ^{1}H NMR Spectrum of (*E*)-(5,5-Difluoropent-2-en-1-yl)benzene (**10v**)

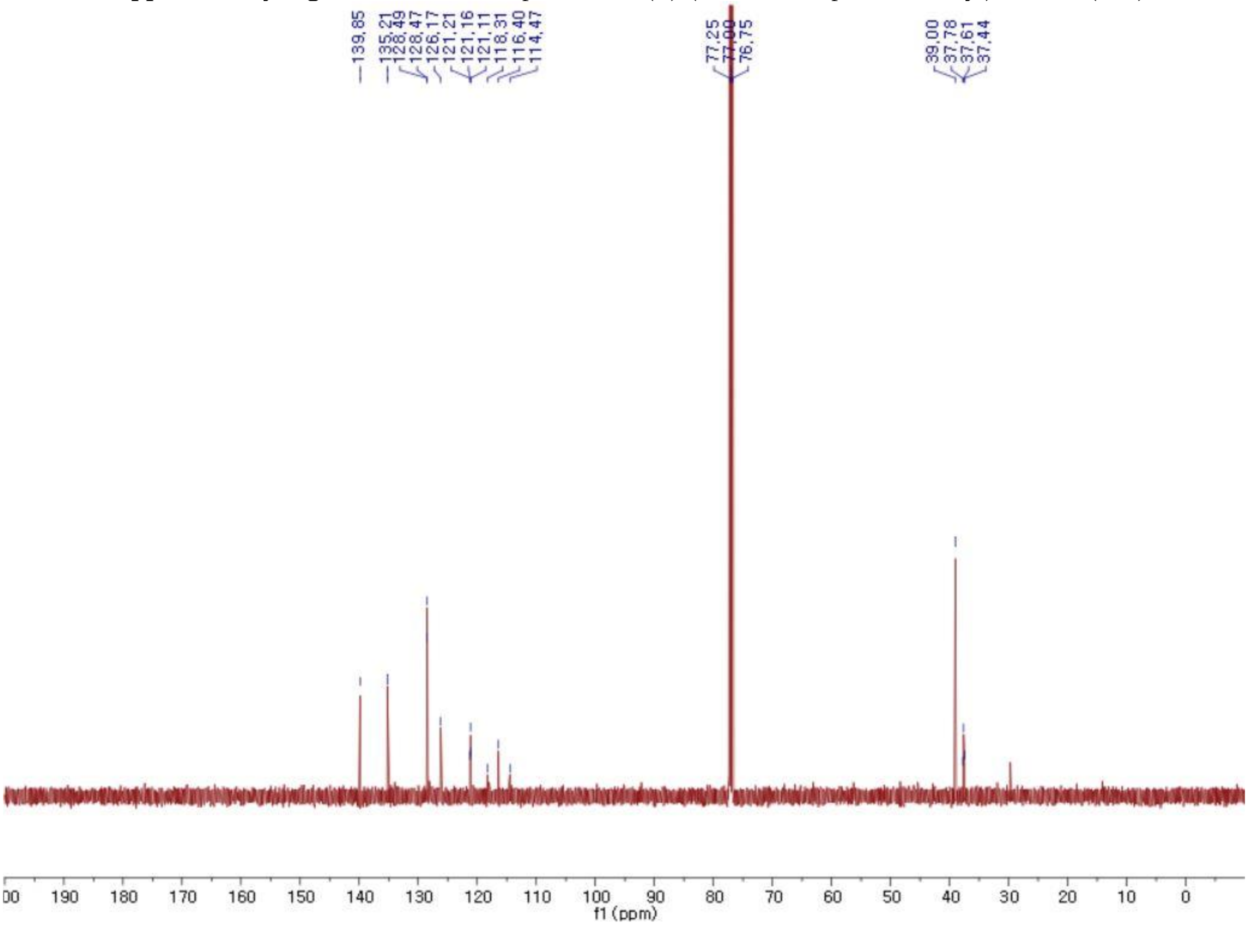

Supplementary Figure 78. ^{13}C NMR Spectrum of (*E*)-(5,5-Difluoropent-2-en-1-yl)benzene (**10v**)

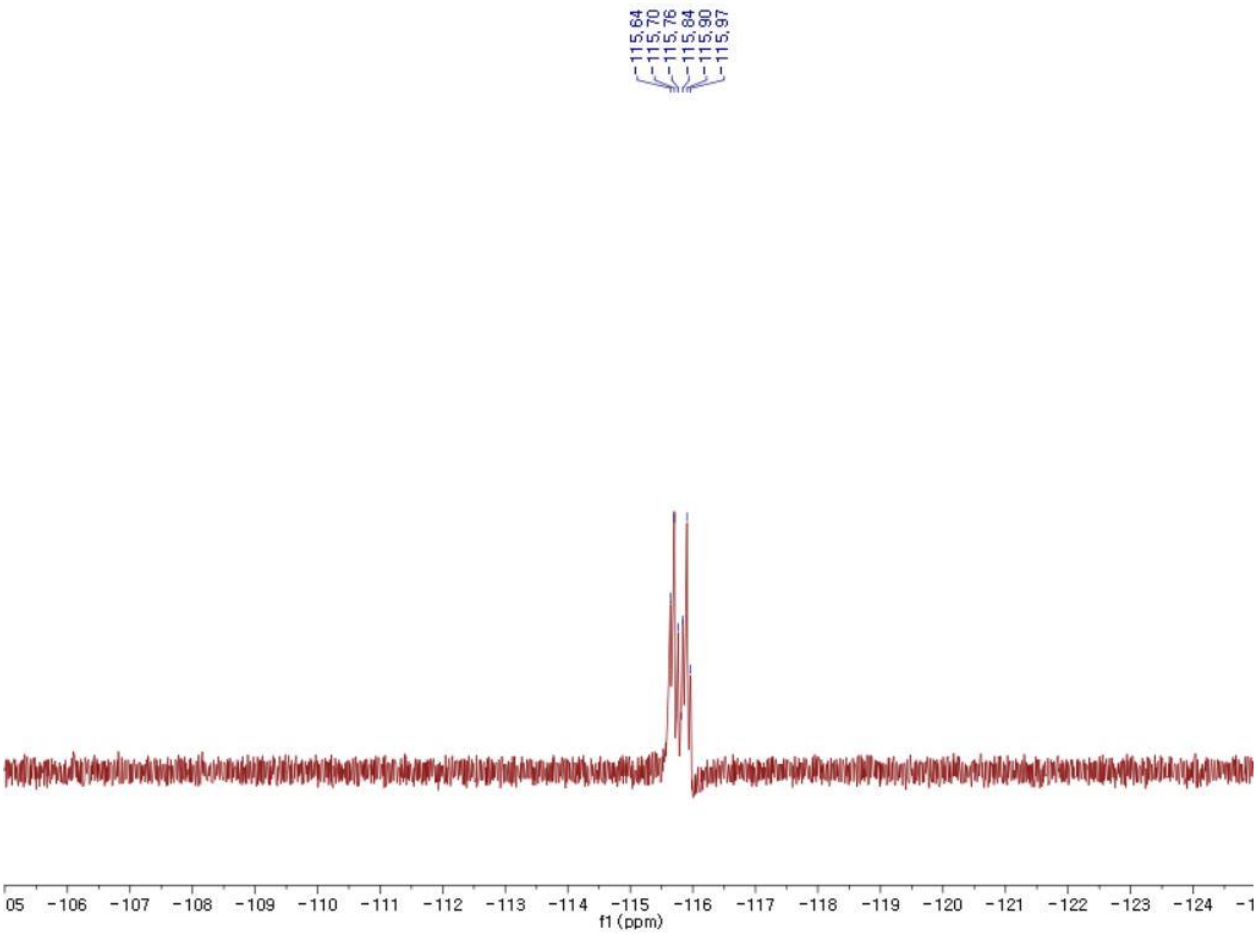

Supplementary Figure 79. ^{19}F NMR Spectrum of (*E*)-(5,5-Difluoropent-2-en-1-yl)benzene (**10v**)

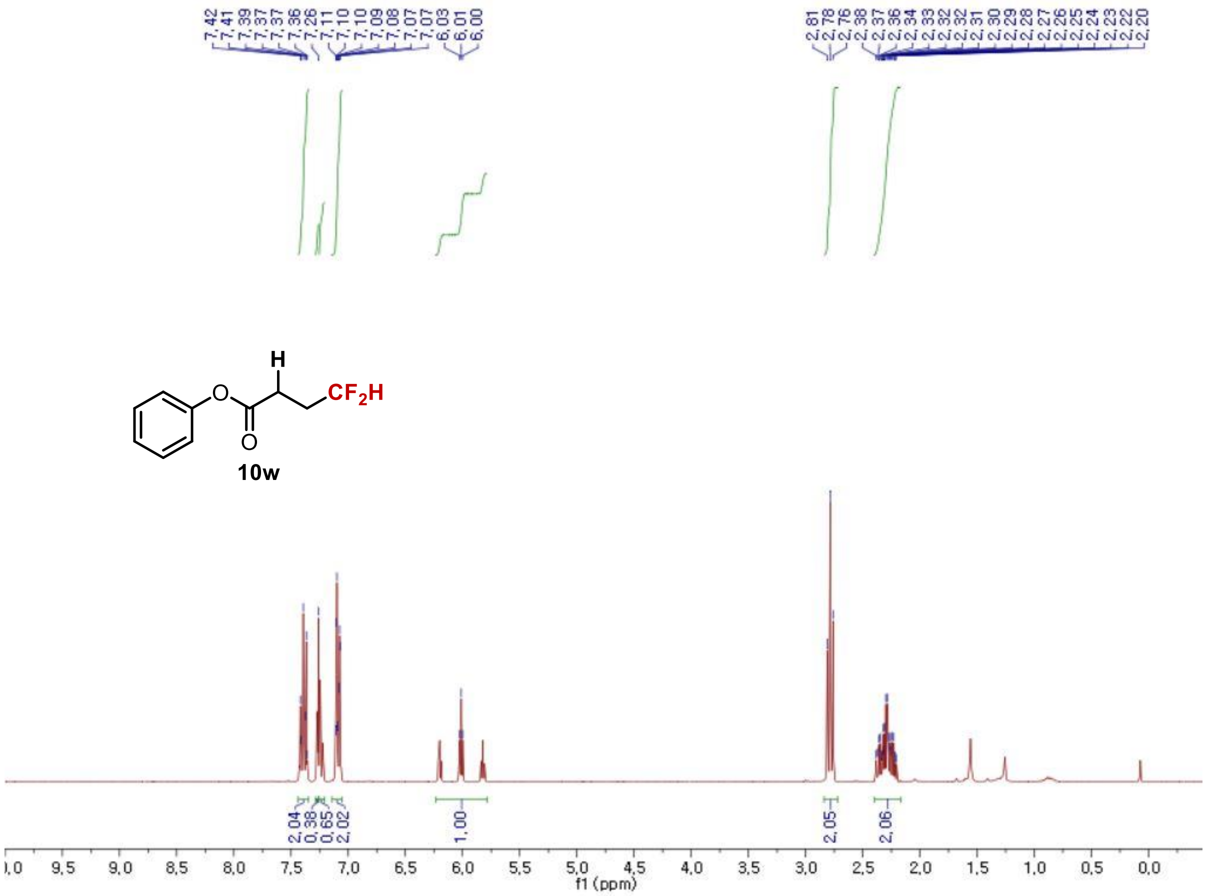

Supplementary Figure 80. ^{1}H NMR Spectrum of Phenyl 4,4-difluorobutanoate (**10w**)

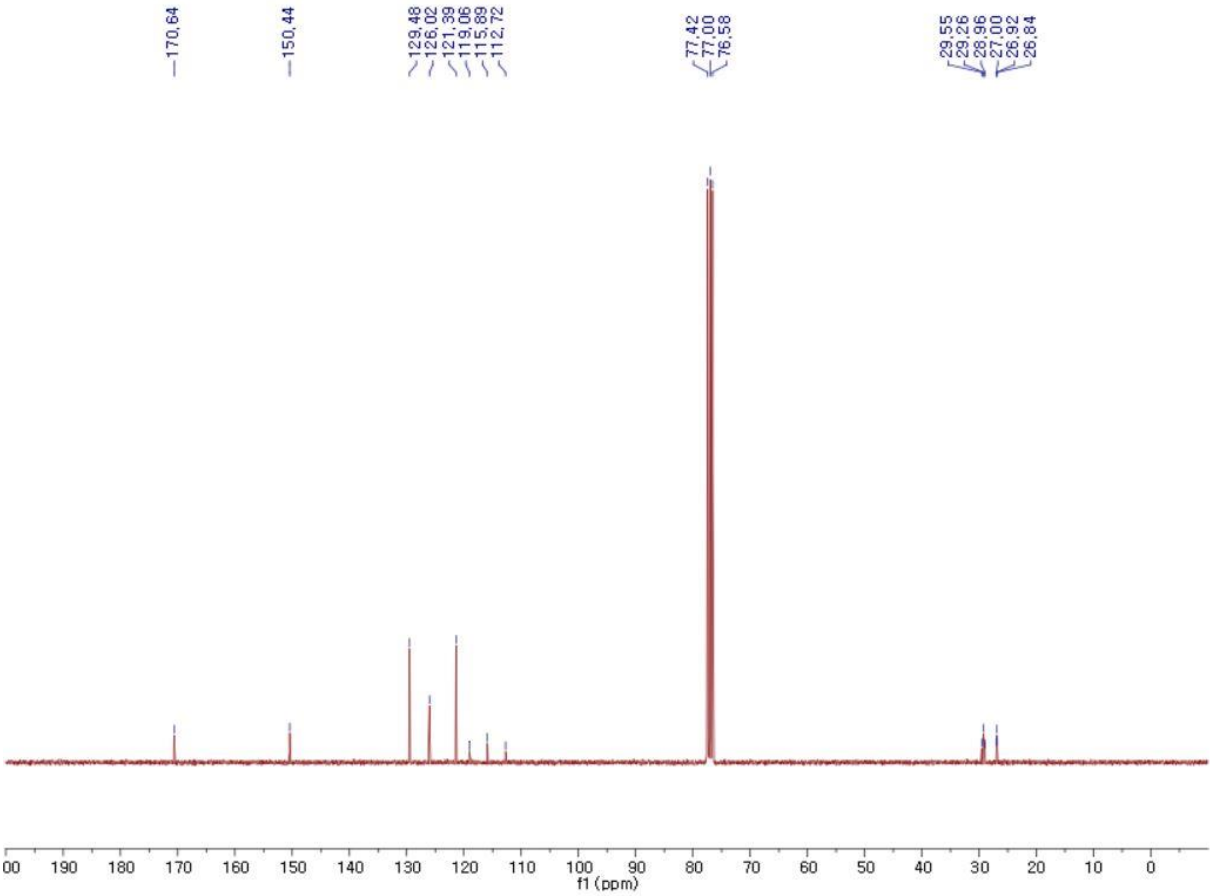

Supplementary Figure 81. ^{13}C NMR Spectrum of Phenyl 4,4-difluorobutanoate (**10w**)

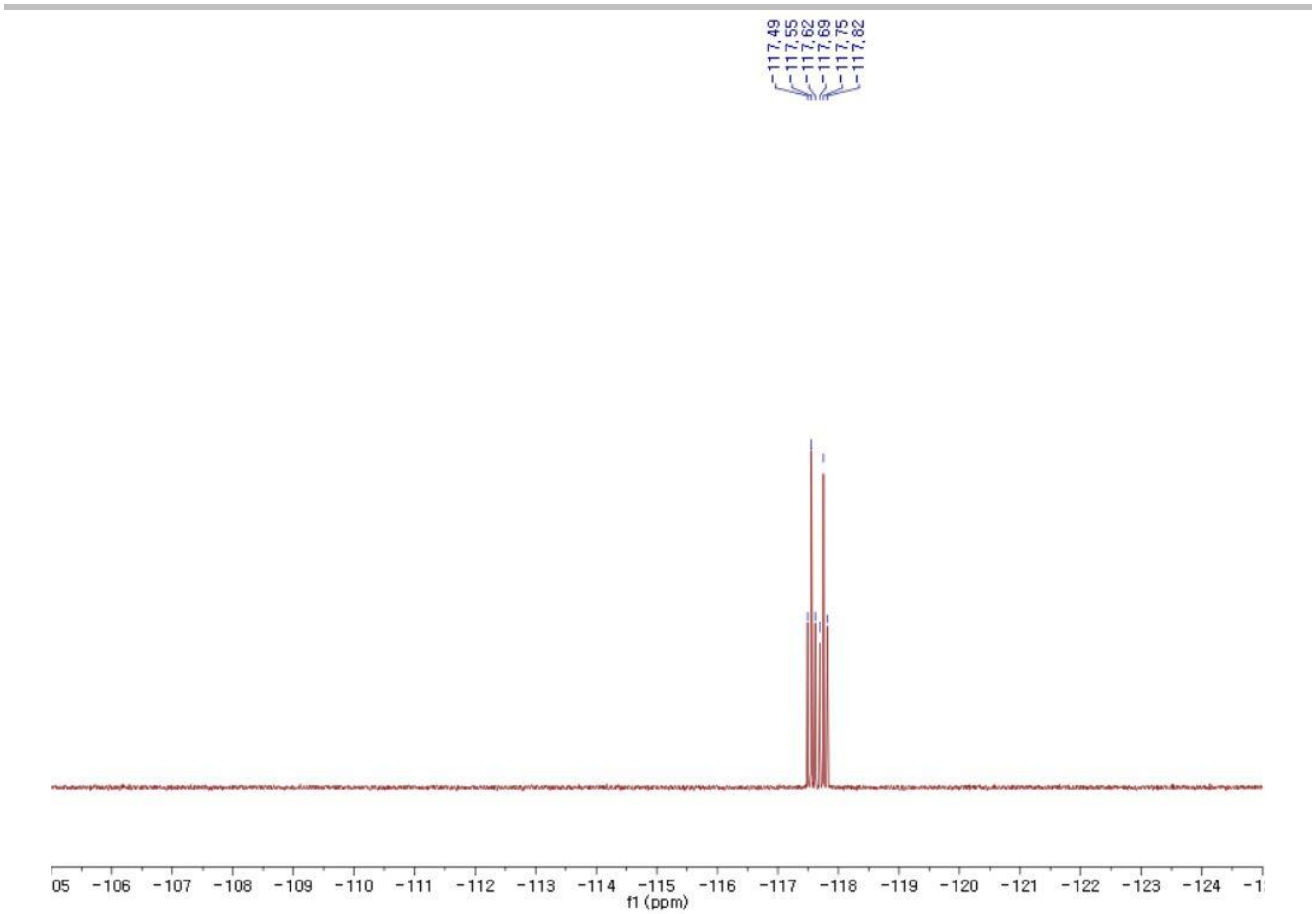

Supplementary Figure 82. ^{19}F NMR Spectrum of Phenyl 4,4-difluorobutanoate (**10w**)

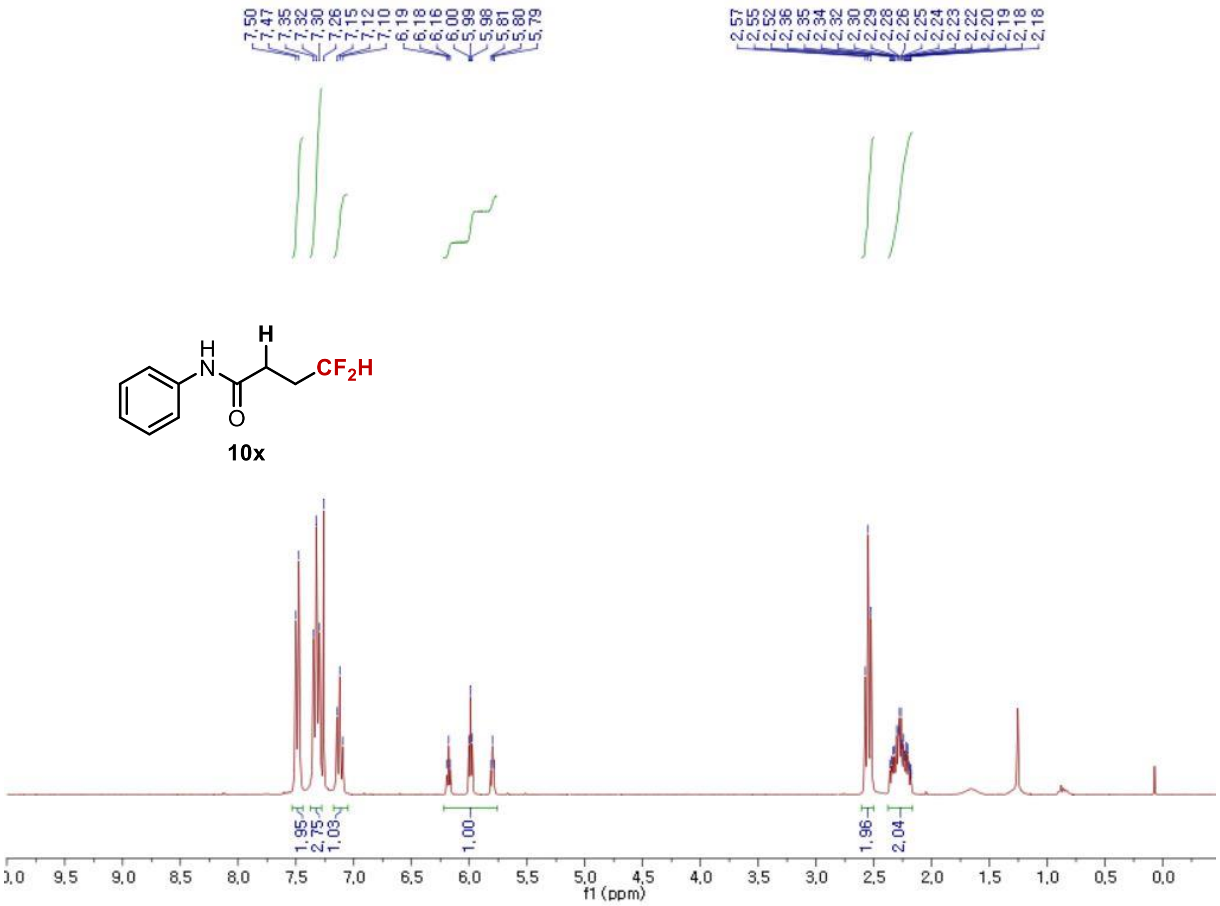

Supplementary Figure 83. ^{1}H NMR Spectrum of 4,4-Difluoro-N-phenylbutanamide (**10x**)

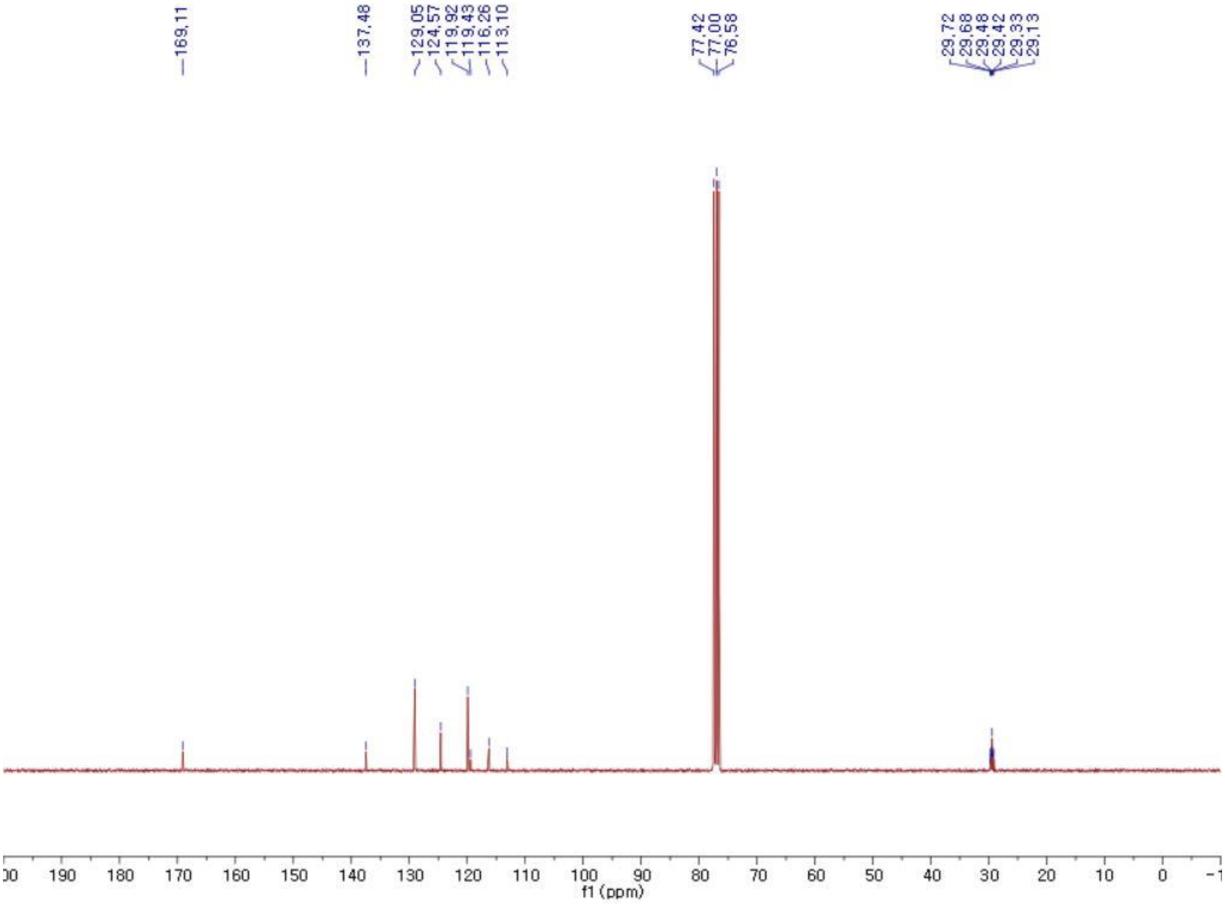

Supplementary Figure 84. ^{13}C NMR Spectrum of 4,4-Difluoro-N-phenylbutanamide (**10x**)

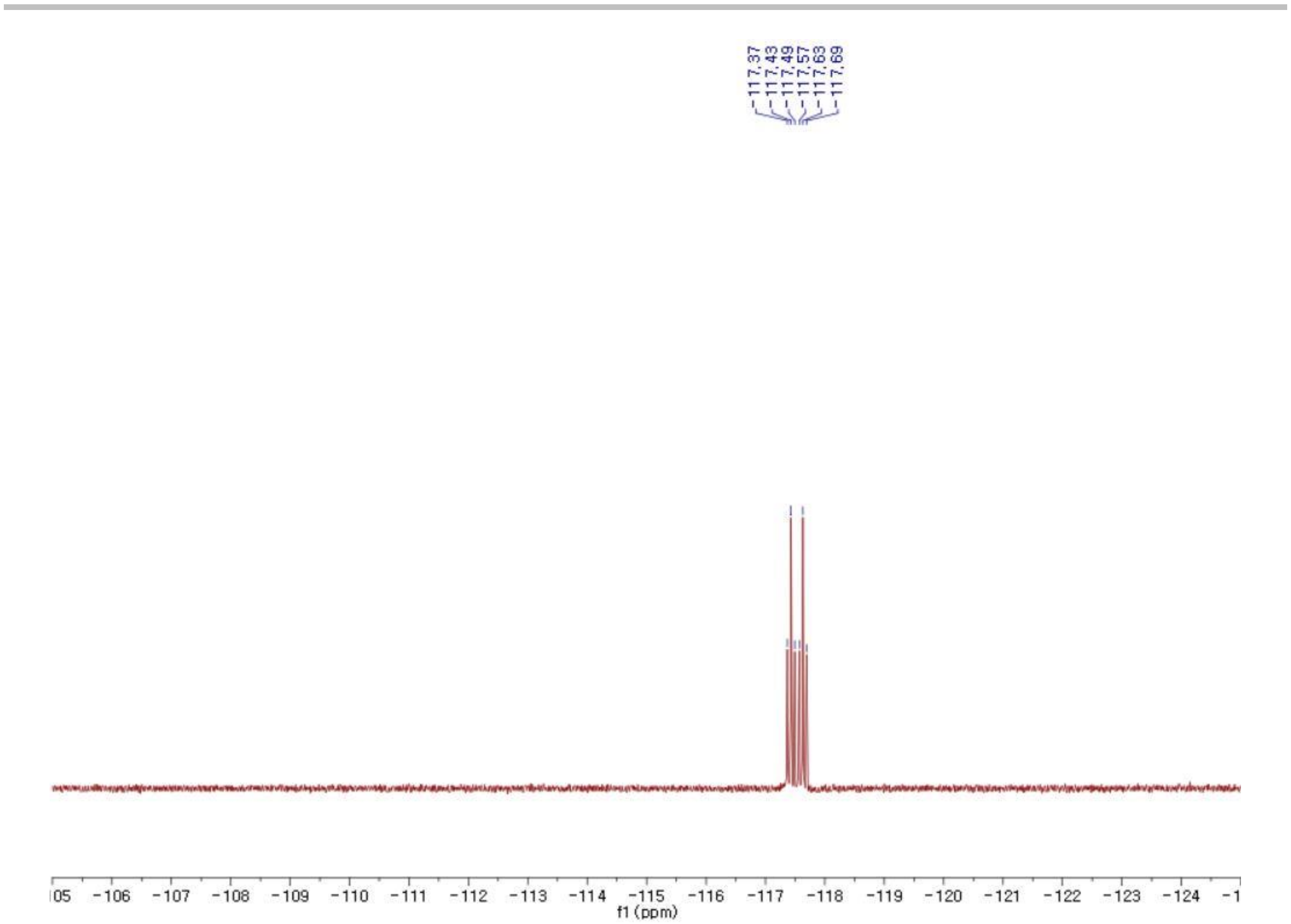

Supplementary Figure 85. ^{19}F NMR Spectrum of 4,4-Difluoro-N-phenylbutanamide (**10x**)

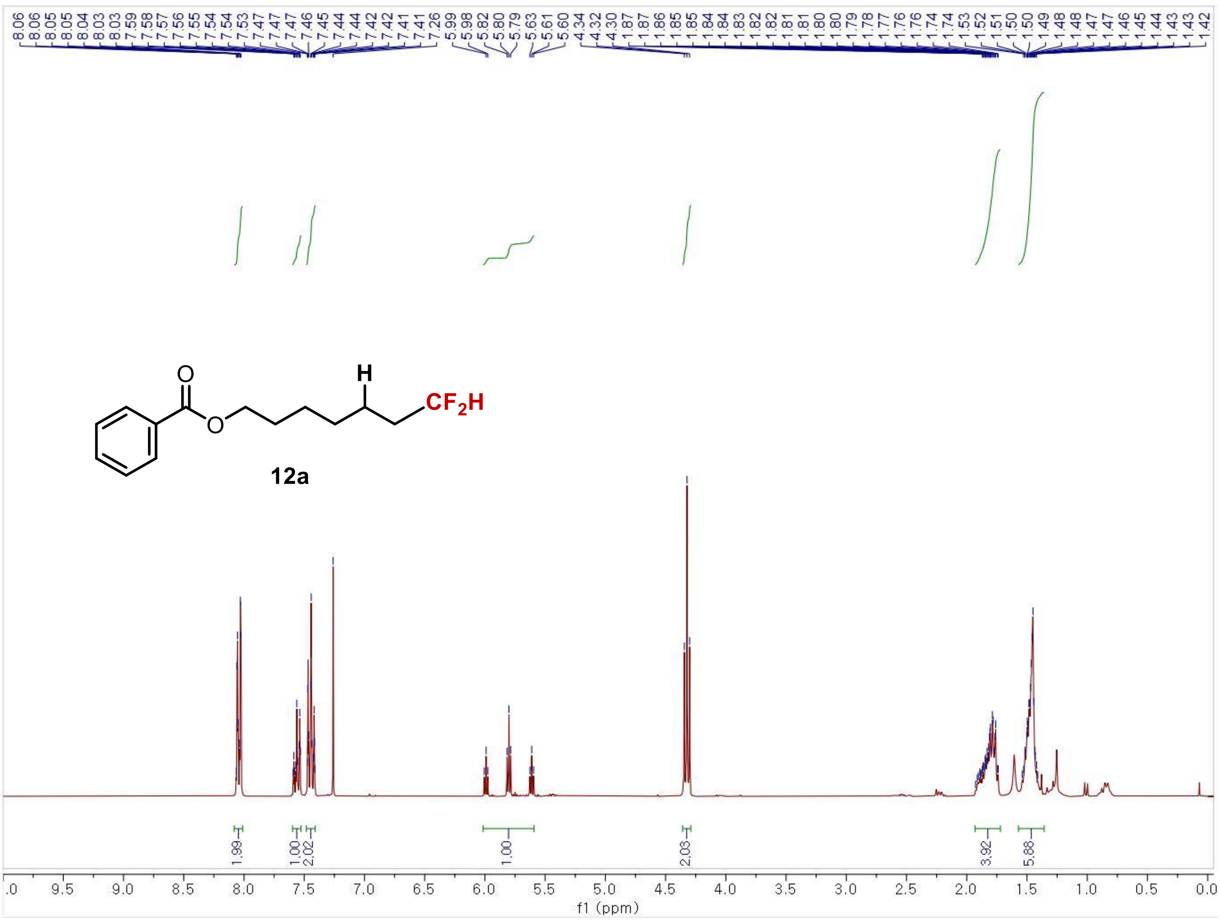

Supplementary Figure 86. ^{1}H NMR Spectrum of 7,7-Difluoroheptyl benzoate (**12a**)

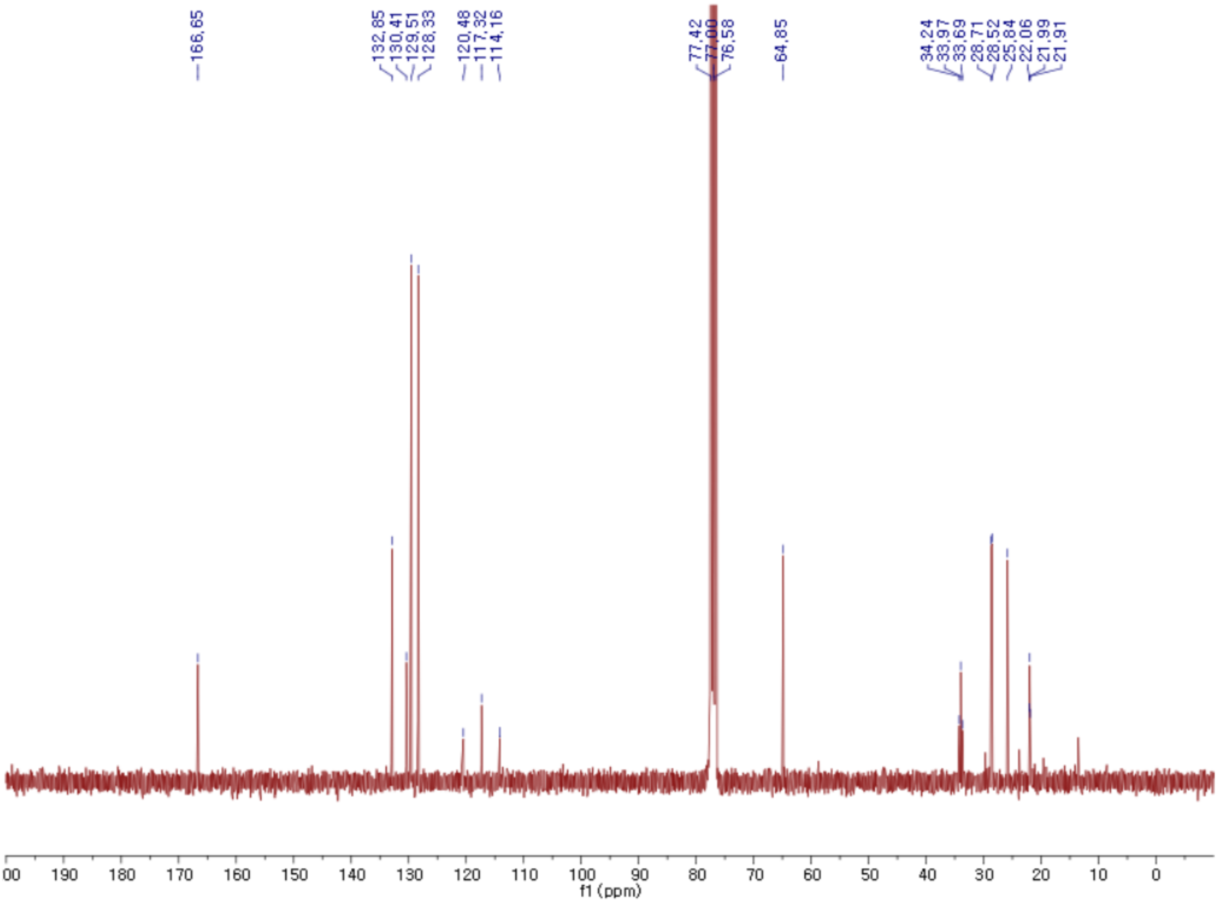

Supplementary Figure 87. ^{13}C NMR Spectrum of 7,7-Difluoroheptyl benzoate (**12a**)

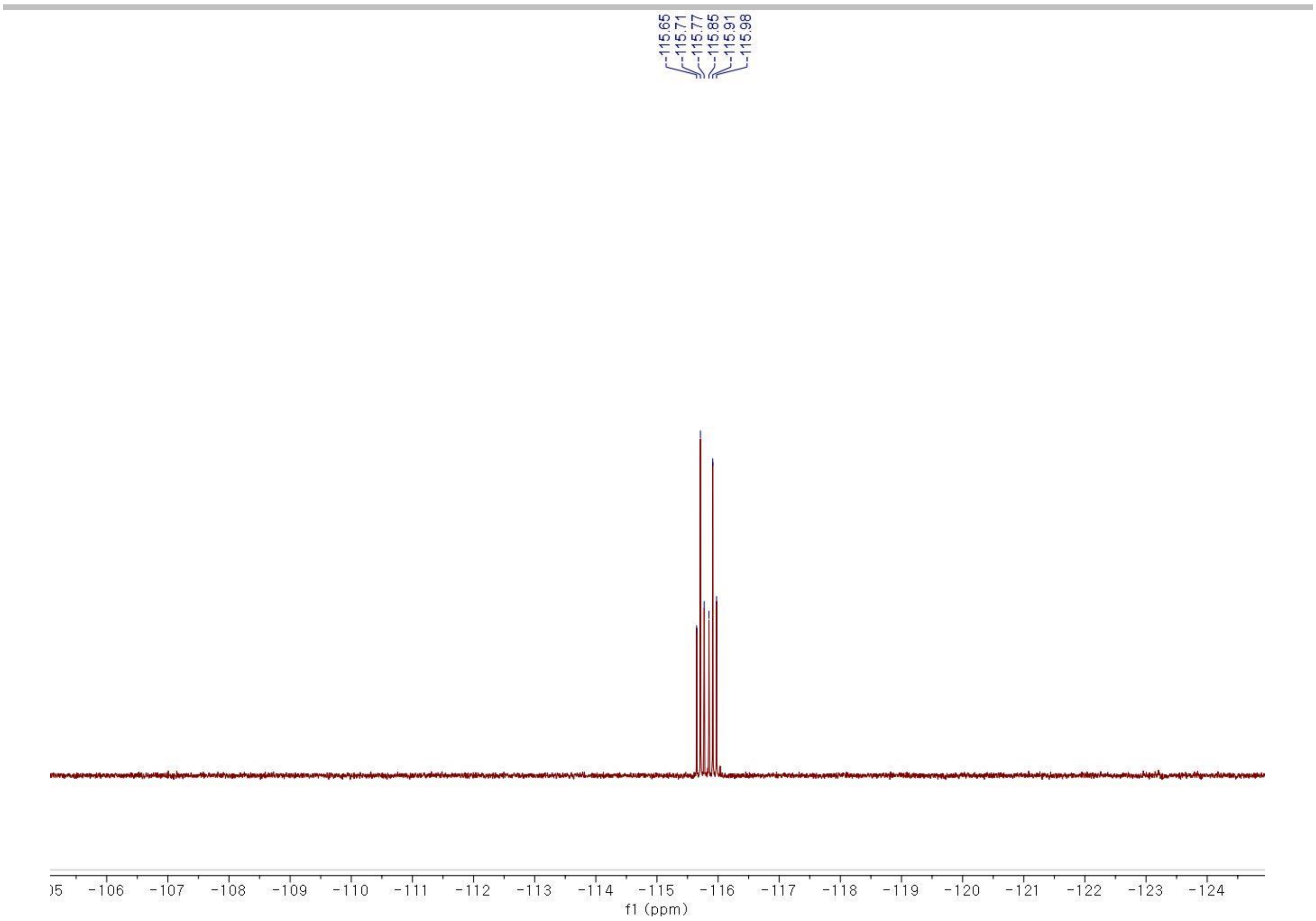

Supplementary Figure 88. ^{19}F NMR Spectrum of 7,7-Difluoroheptyl benzoate (**12a**)

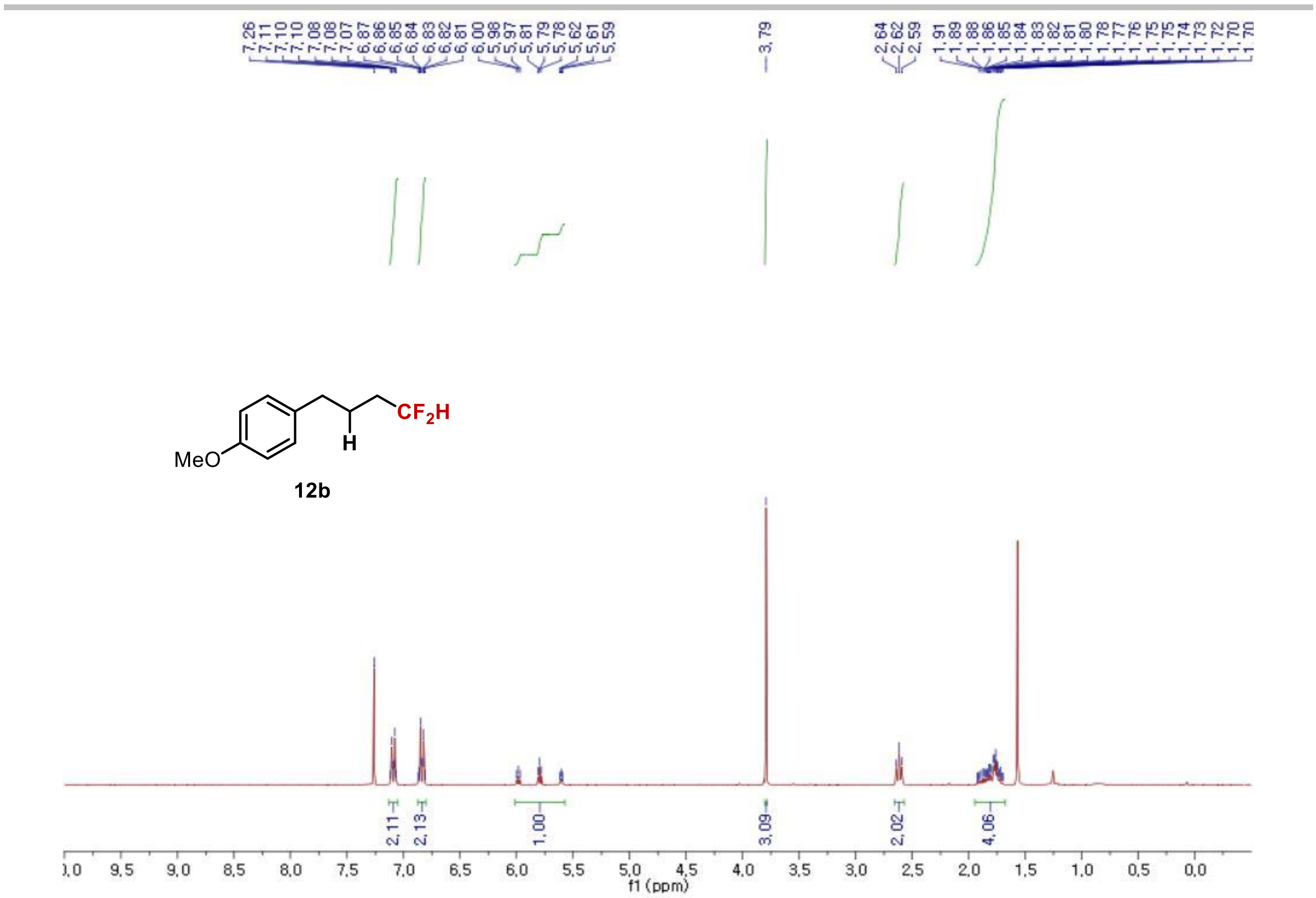

Supplementary Figure 89. ^{1}H NMR Spectrum of 1-(4,4-Difluorobutyl)-4-methoxybenzene (**12b**)

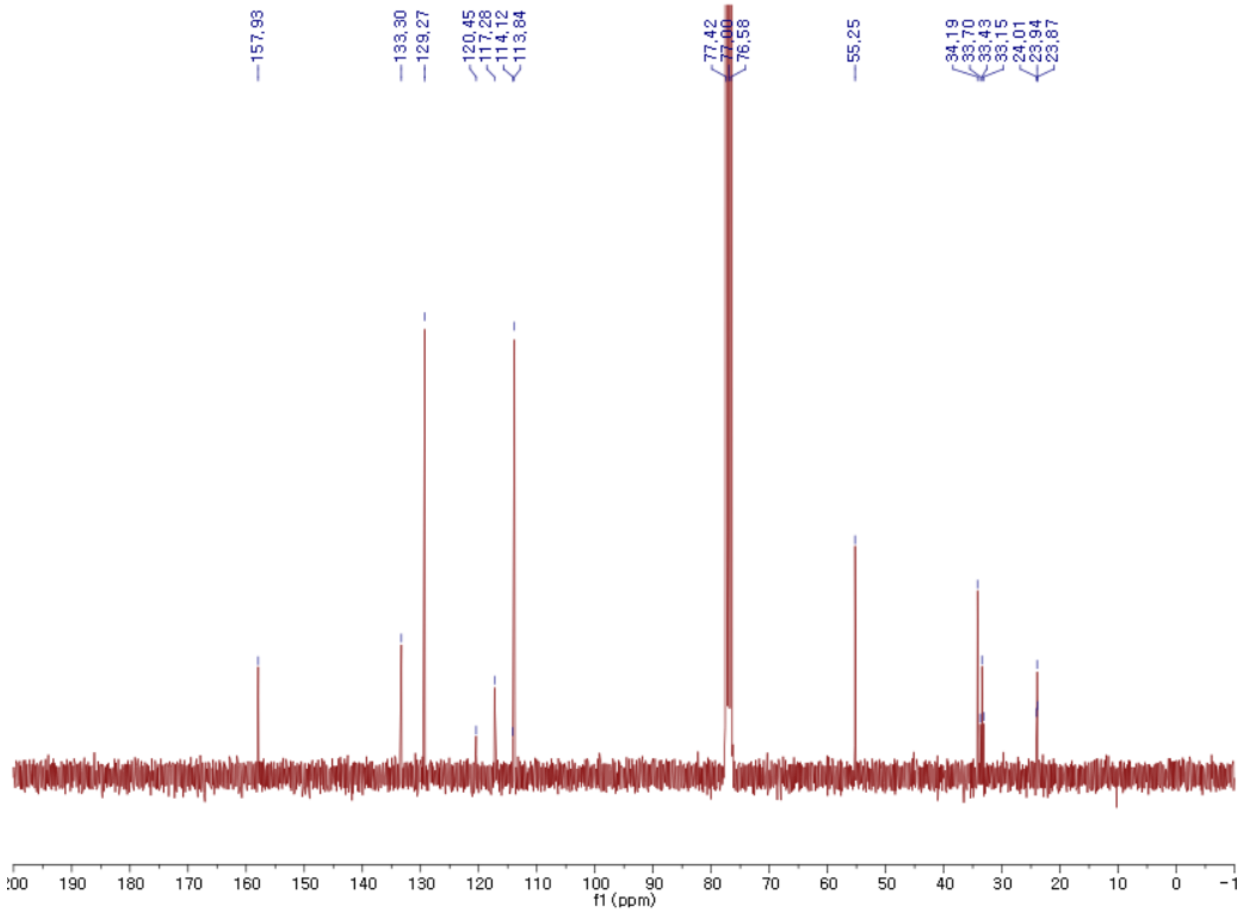

Supplementary Figure 90. ^{13}C NMR Spectrum of 1-(4,4-Difluorobutyl)-4-methoxybenzene (**12b**)

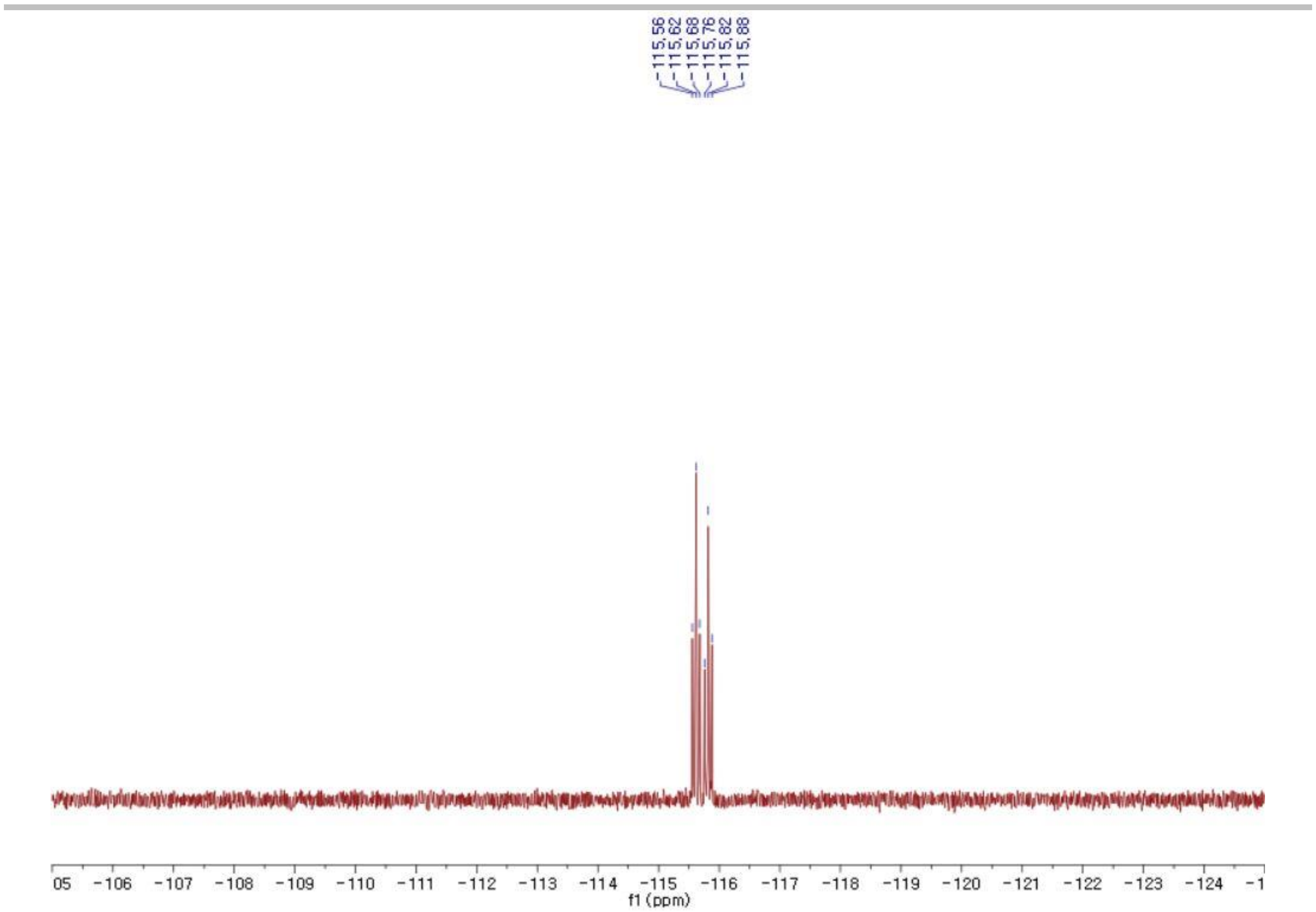

Supplementary Figure 91. ^{19}F NMR Spectrum of 1-(4,4-Difluorobutyl)-4-methoxybenzene (**12b**)

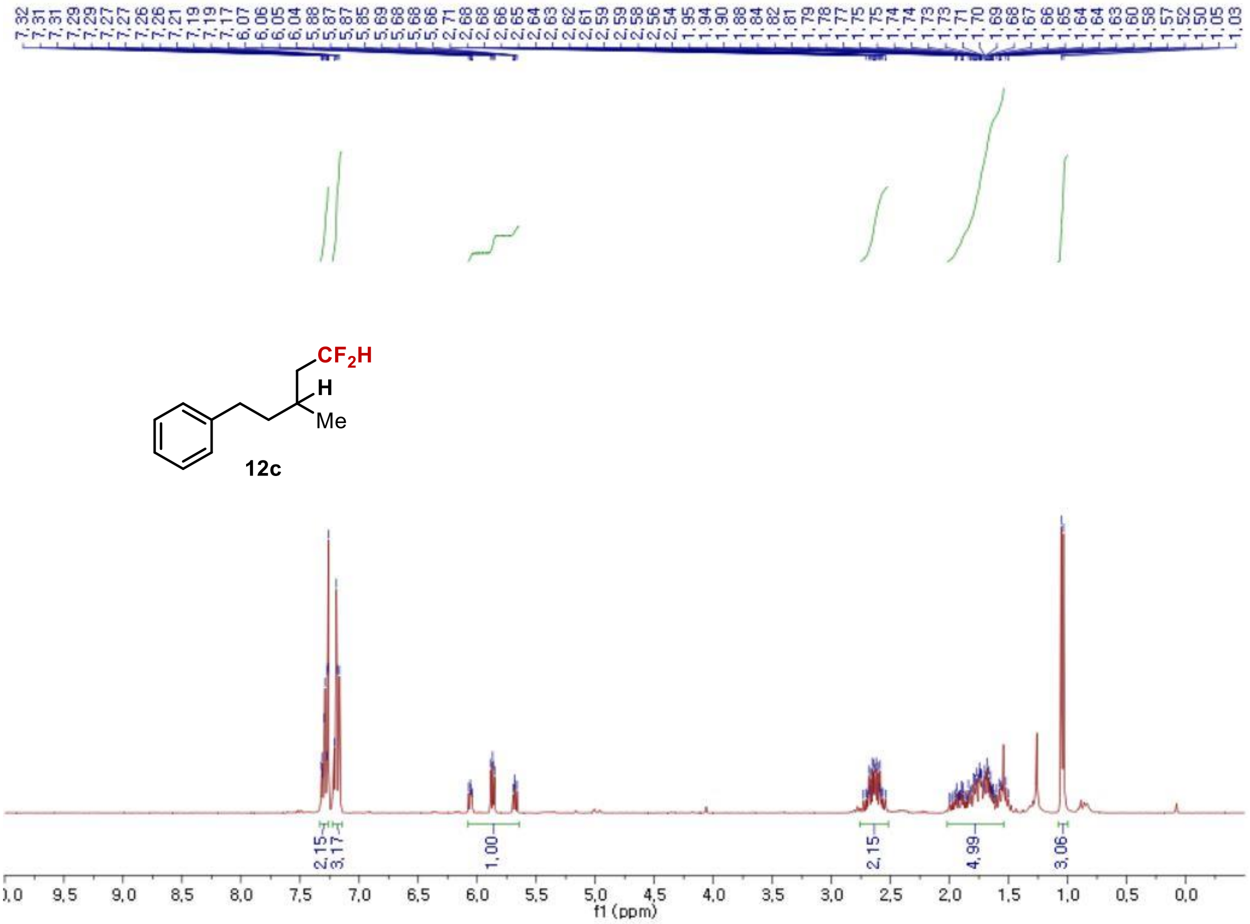

Supplementary Figure 92. ^{1}H NMR Spectrum of (5,5-Difluoro-3-methylpentyl)benzene (**12c**)

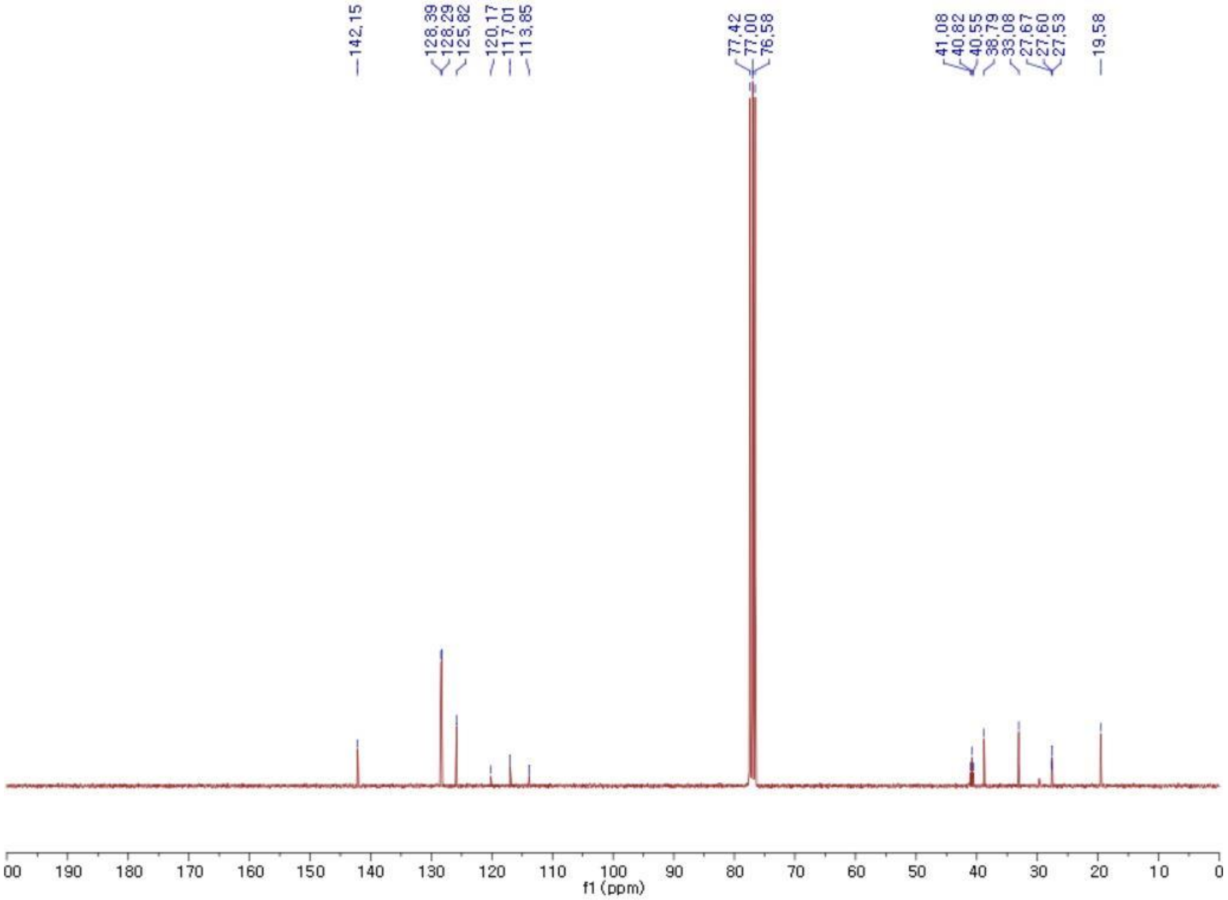

Supplementary Figure 93. ^{13}C NMR Spectrum of (5,5-Difluoro-3-methylpentyl)benzene (**12c**)

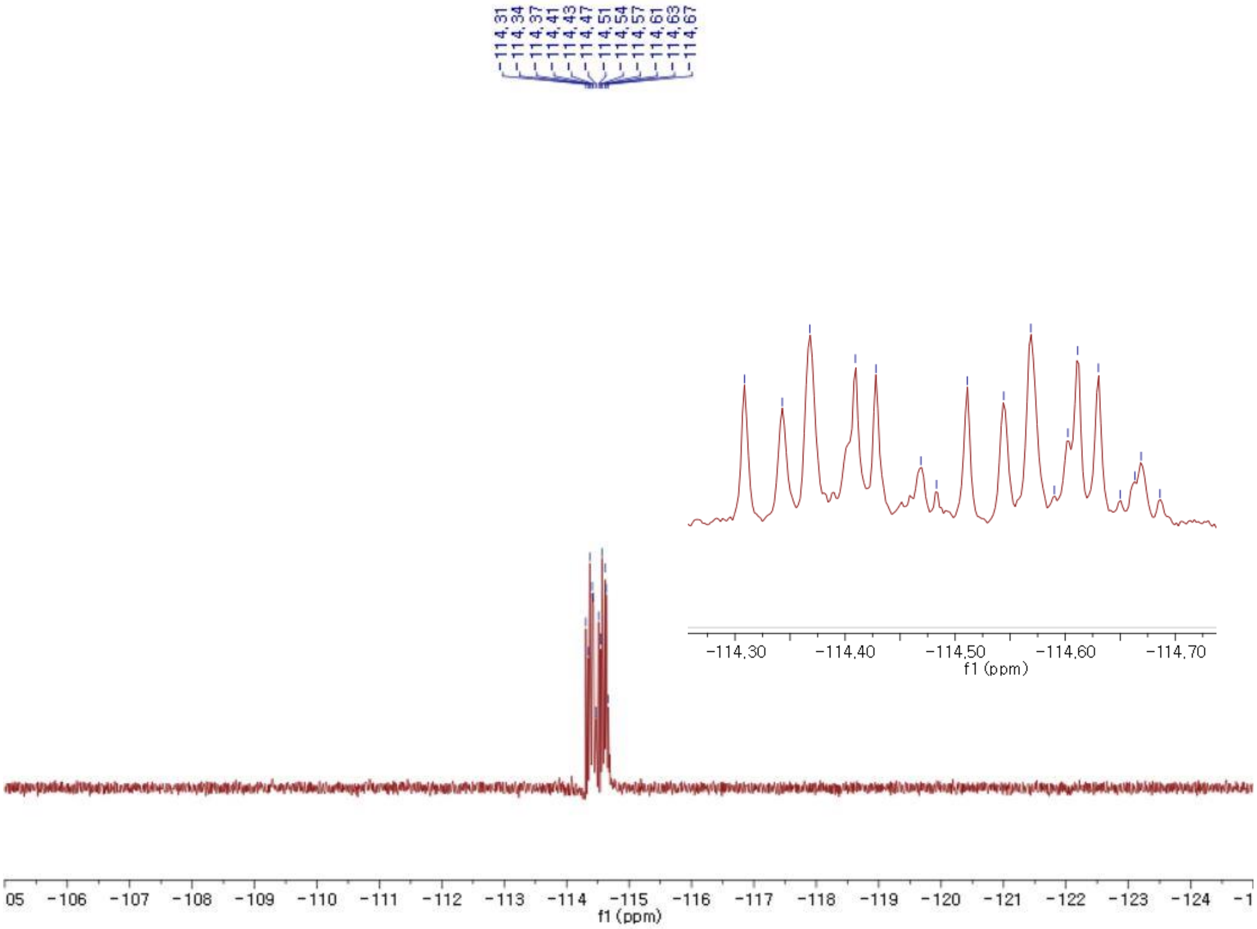

Supplementary Figure 94. ^{19}F NMR Spectrum of (5,5-Difluoro-3-methylpentyl)benzene (**12c**)

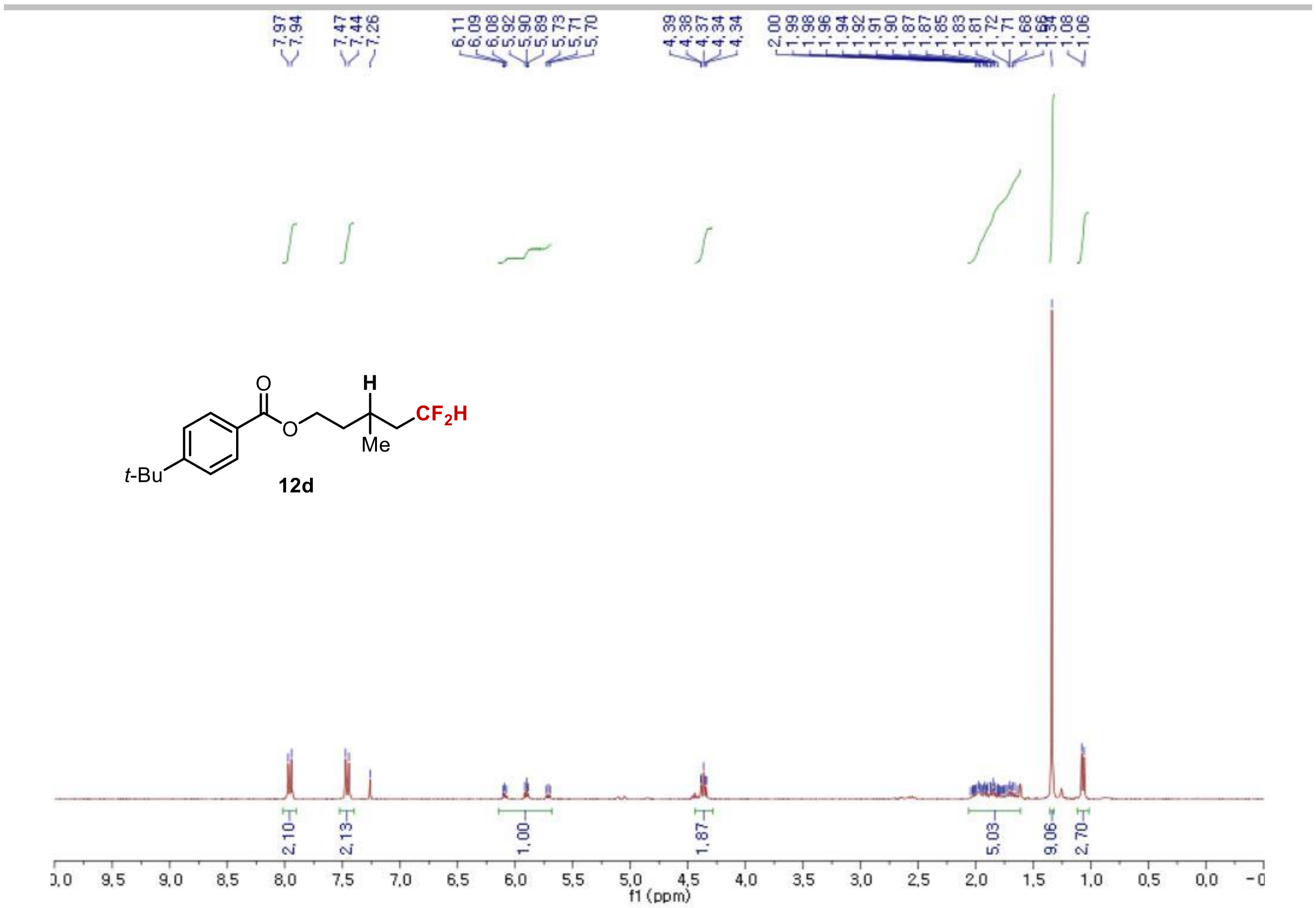

Supplementary Figure 95. ^{1}H NMR Spectrum of 5,5-difluoro-3-methylpentyl 4-(tert-butyl)benzoate (**12d**)

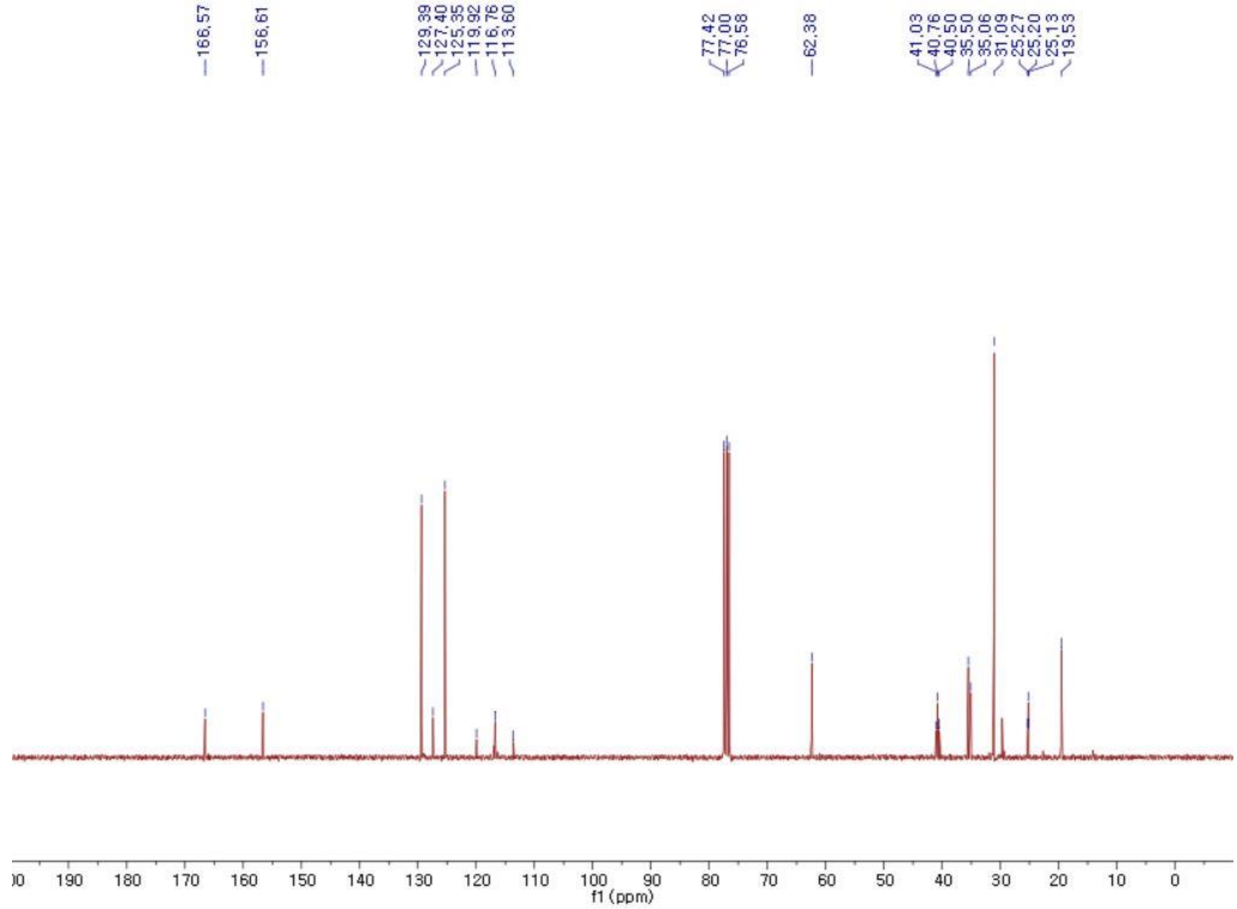

Supplementary Figure 96. ^{13}C NMR Spectrum of 5,5-difluoro-3-methylpentyl 4-(tert-butyl)benzoate (**12d**)

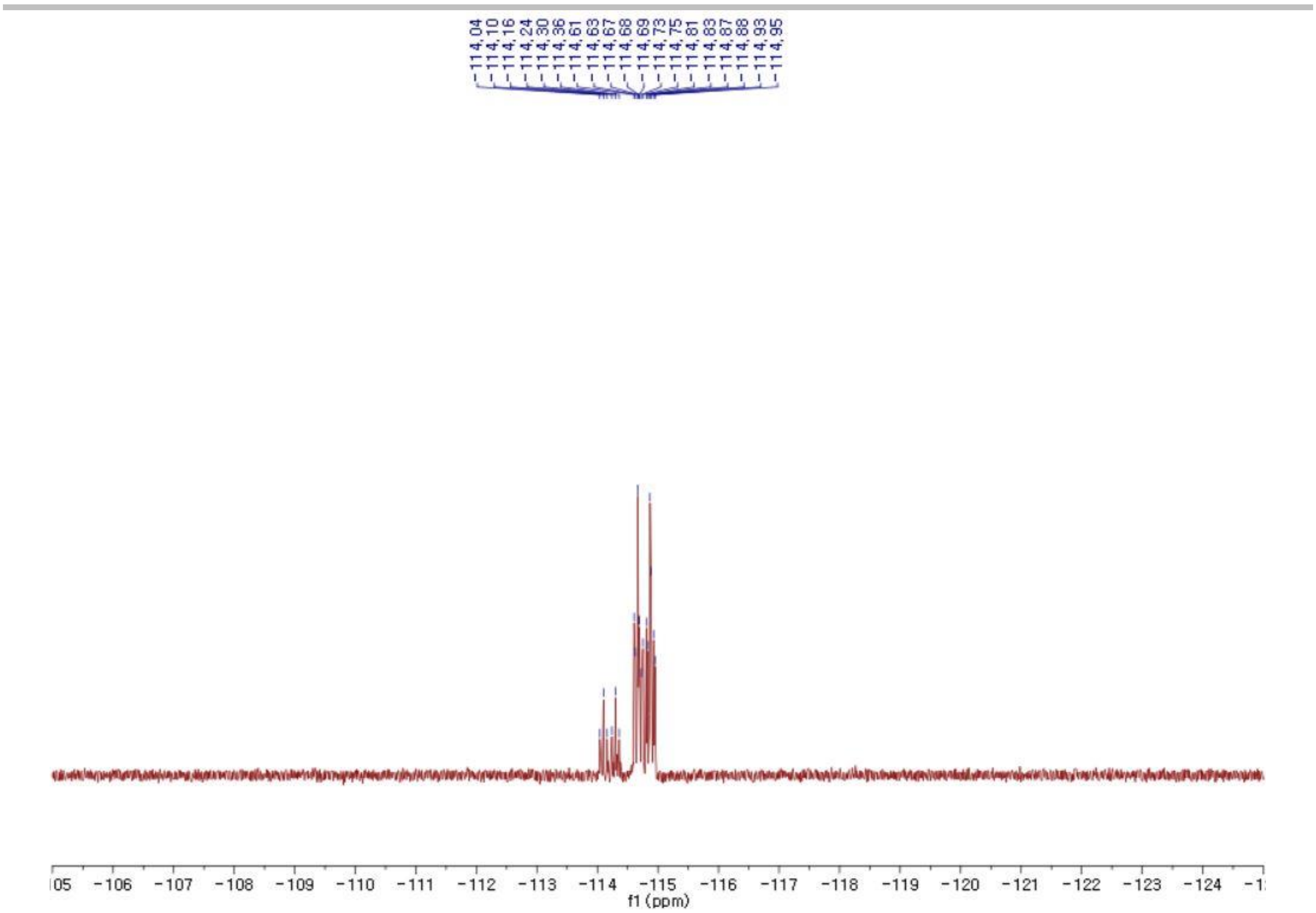

Supplementary Figure 97. ^{19}F NMR Spectrum of 5,5-difluoro-3-methylpentyl 4-(tert-butyl)benzoate (**12d**)

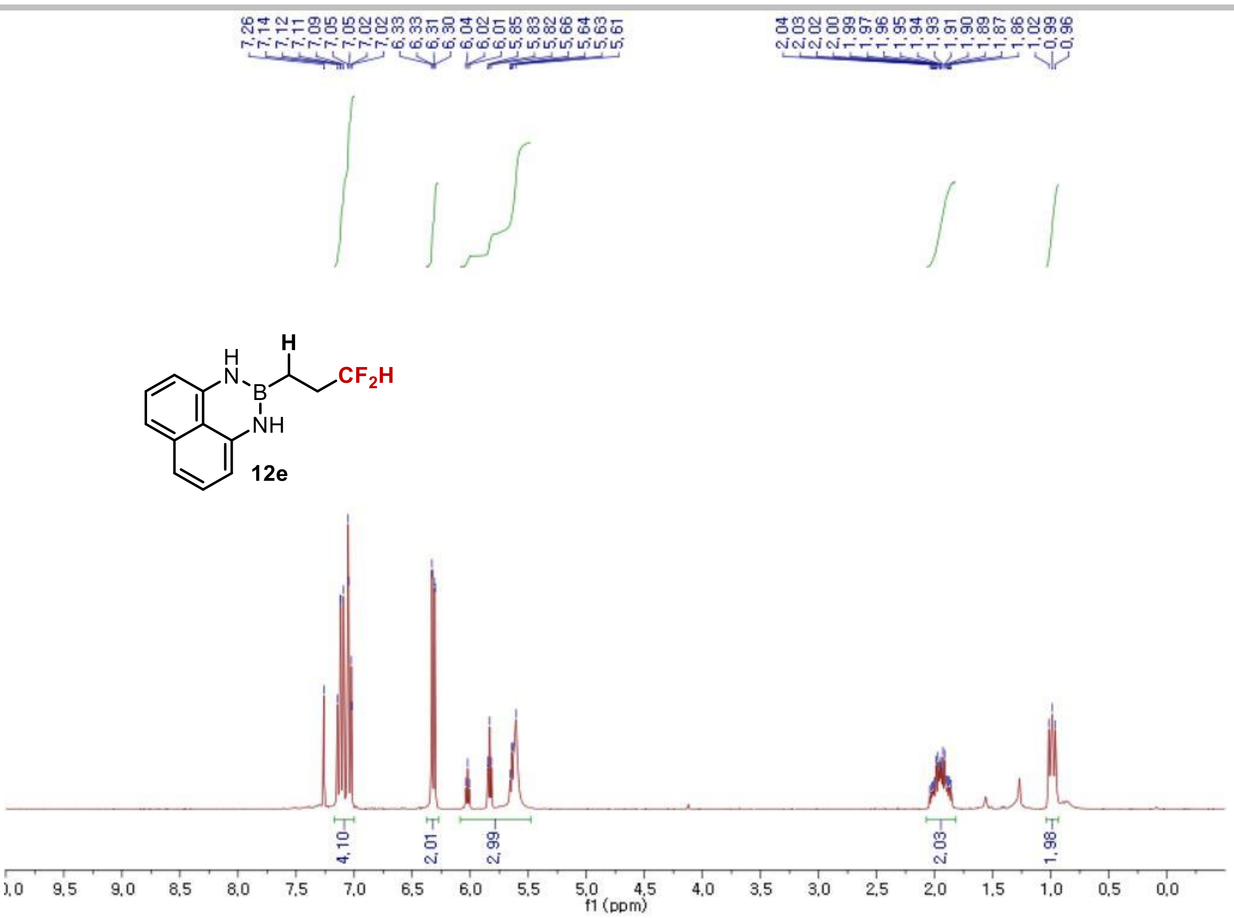

Supplementary Figure 98. ^{1}H NMR Spectrum of 2-(3,3-Difluoropropyl)-2,3-dihydro-1H-naphtho[1,8-de][1,3,2]diazaborinine (**12e**)

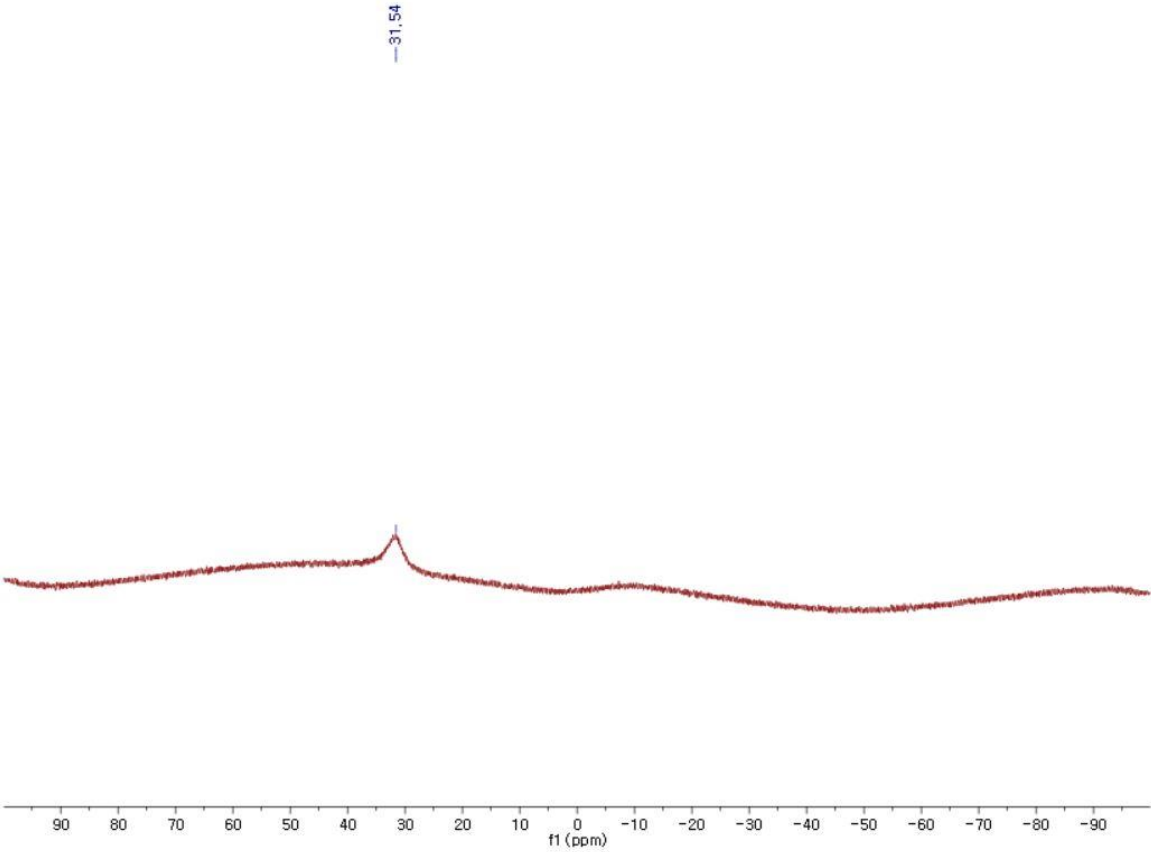

Supplementary Figure 99. ^{11}B NMR Spectrum of 2-(3,3-Difluoropropyl)-2,3-dihydro-1H-naphtho[1,8-de][1,3,2]diazaborinine (**12e**)

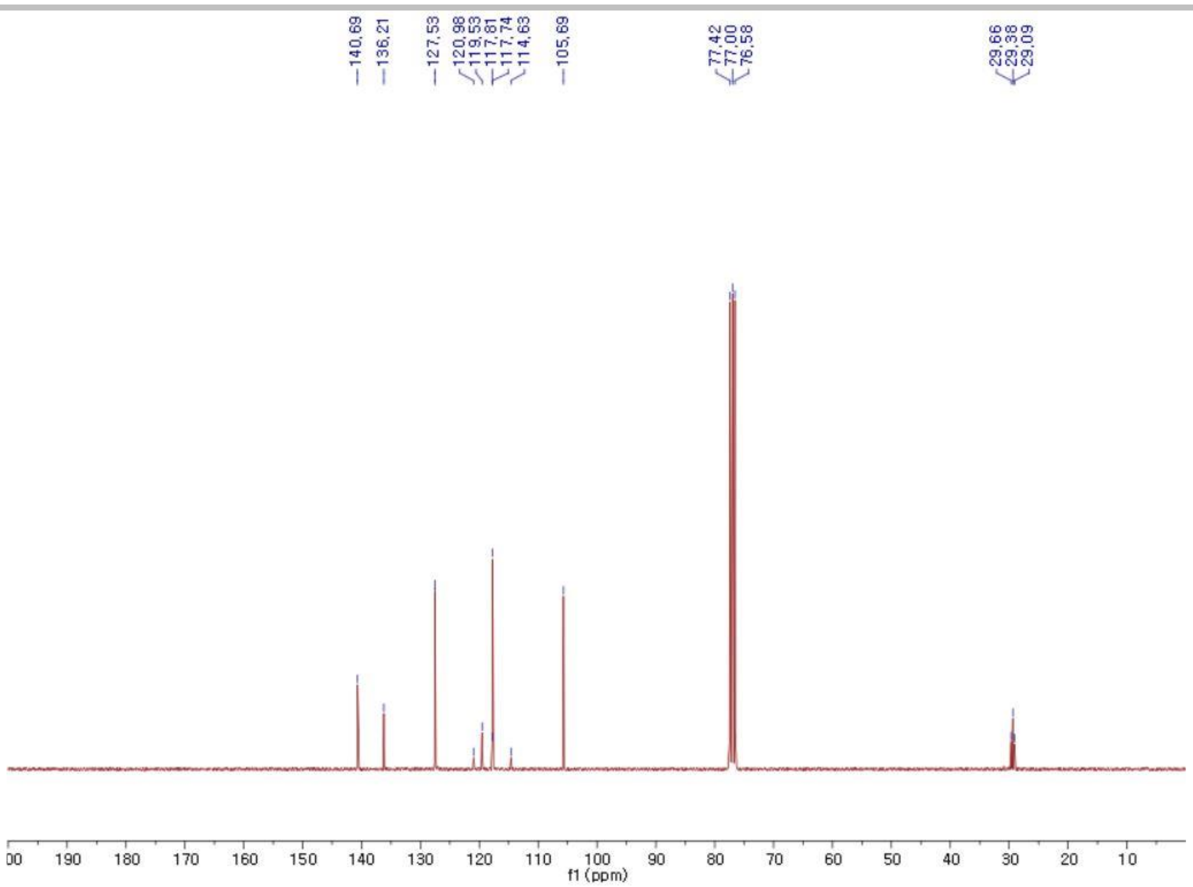

Supplementary Figure 100. ^{13}C NMR Spectrum of 2-(3,3-Difluoropropyl)-2,3-dihydro-1H-naphtho[1,8-de][1,3,2]diazaborinine (**12e**)

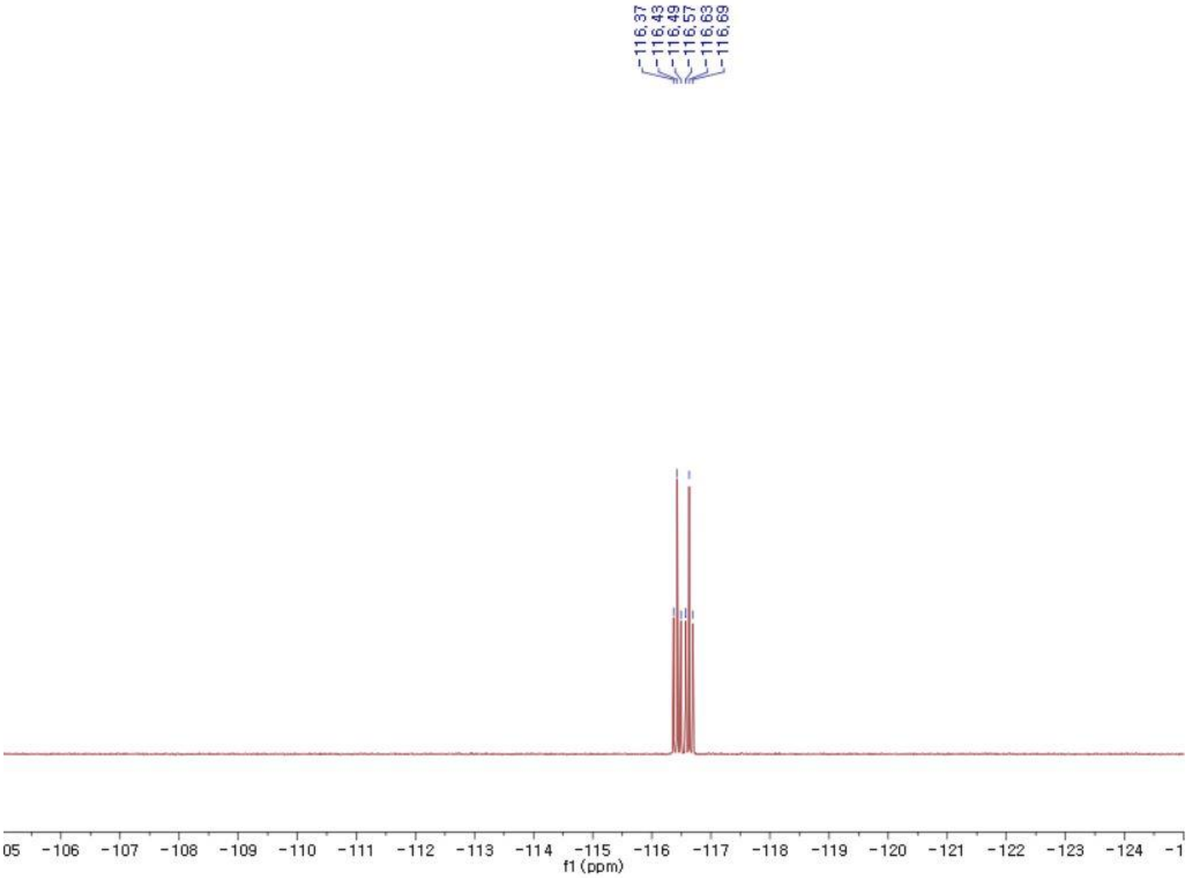

Supplementary Figure 101. ^{19}F NMR Spectrum of 2-(3,3-Difluoropropyl)-2,3-dihydro-1H-naphtho[1,8-de][1,3,2]diazaborinine (**12e**)

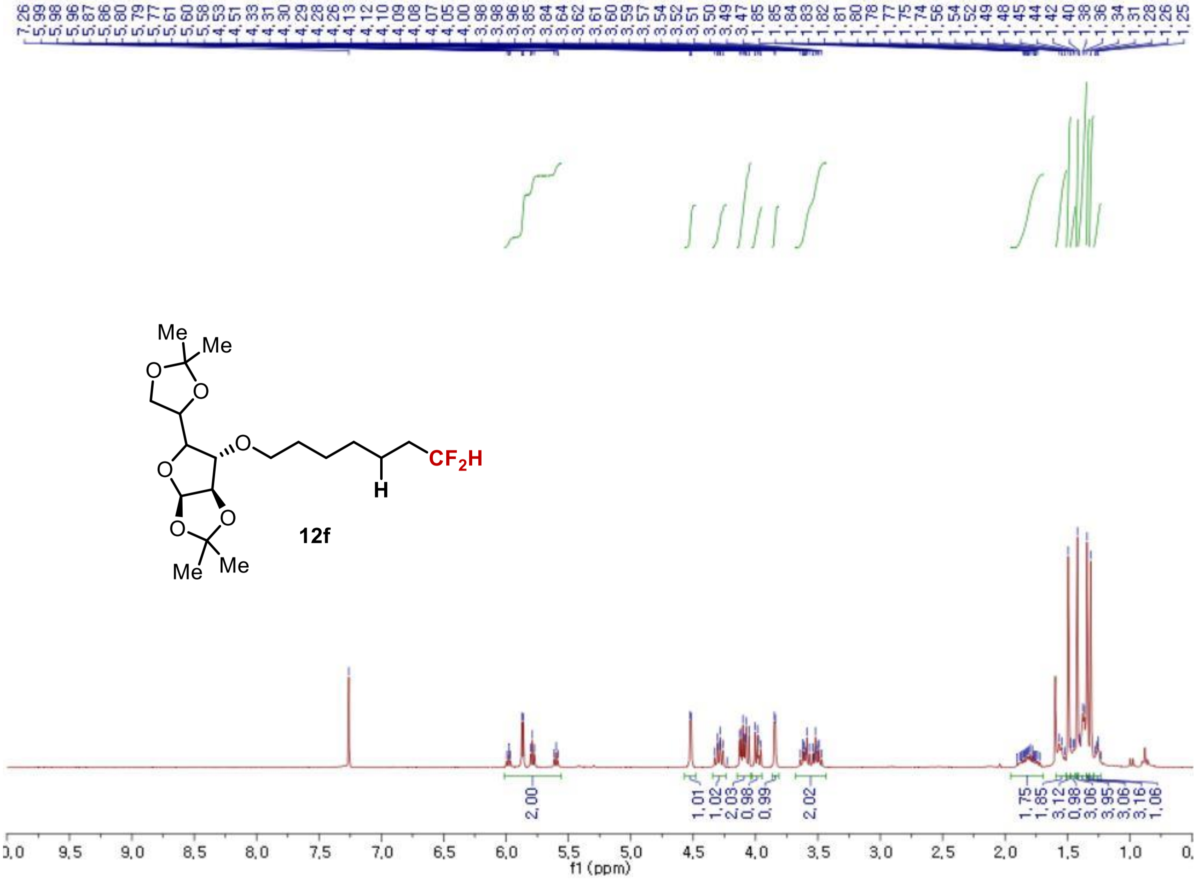

Supplementary Figure 102. ^{1}H NMR Spectrum of (3aR,6S,6aR)-6-((7,7-Difluoroheptyl)oxy)-5-(2,2-dimethyl-1,3-dioxolan-4-yl)-2,2-dimethyltetrahydrofuro[2,3-d][1,3]dioxole (**12f**)

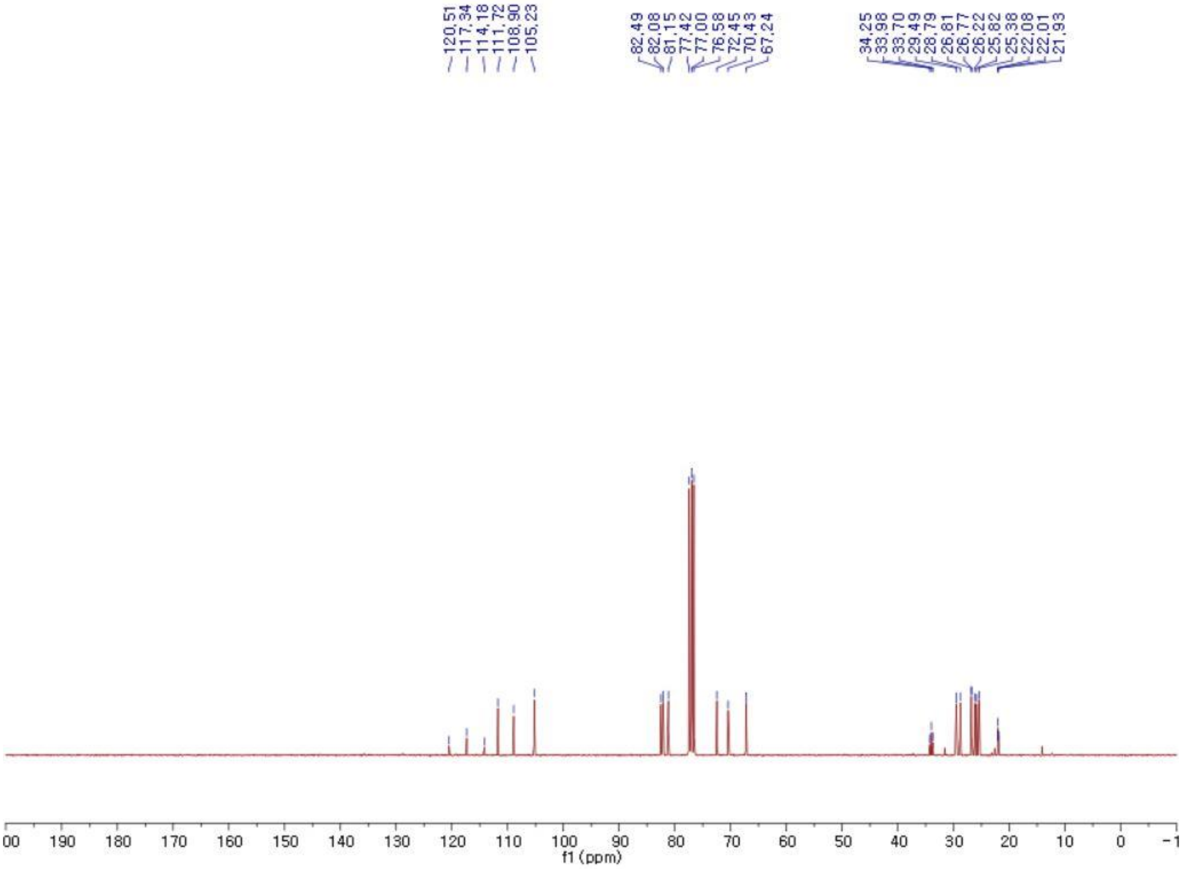

Supplementary Figure 103. ^{13}C NMR Spectrum of (3aR,6S,6aR)-6-((7,7-Difluoroheptyl)oxy)-5-(2,2-dimethyl-1,3-dioxolan-4-yl)-2,2-dimethyltetrahydrofuro[2,3-d][1,3]dioxole (**12f**)

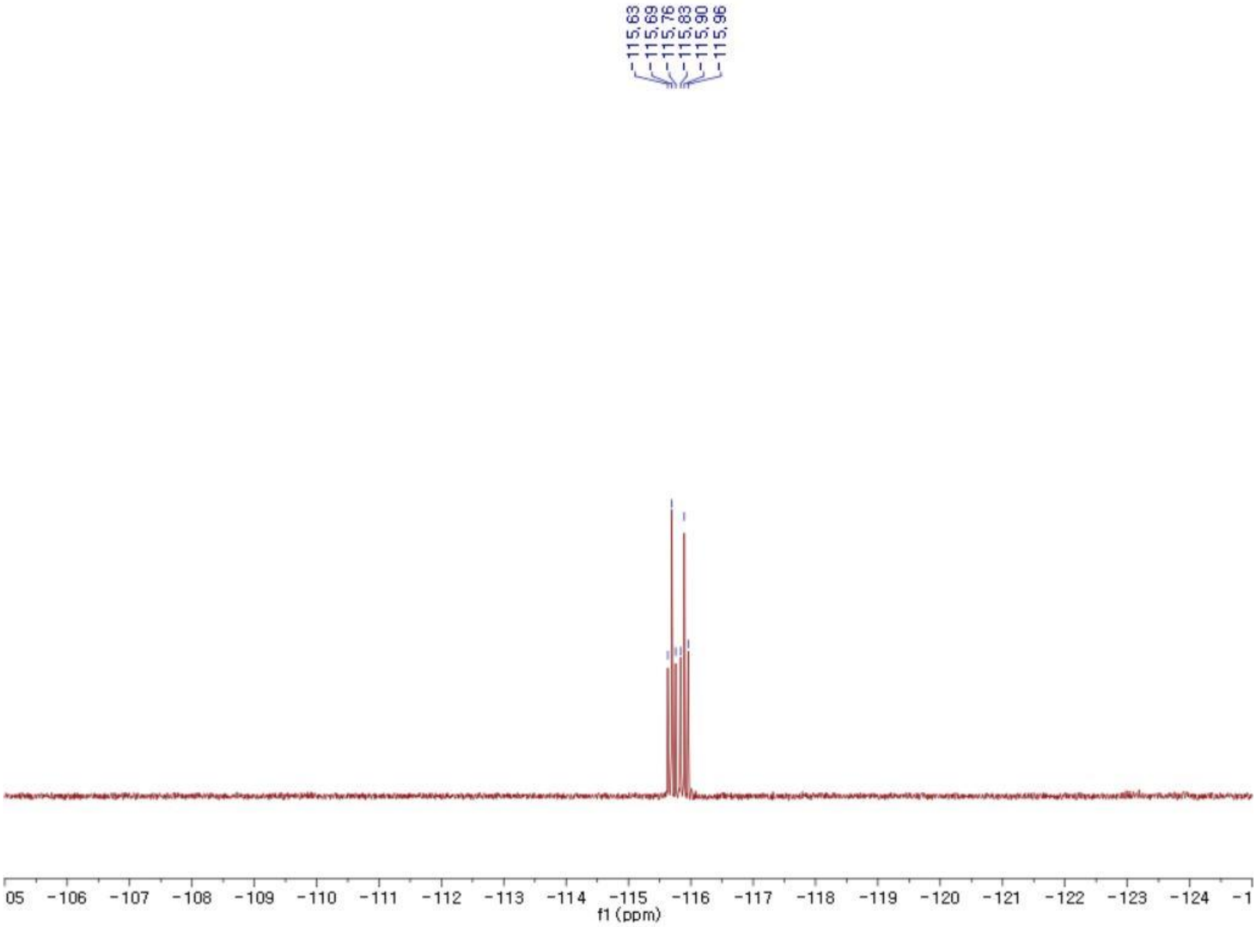

Supplementary Figure 104. ^{19}F NMR Spectrum of (3aR,6S,6aR)-6-((7,7-Difluoroheptyl)oxy)-5-(2,2-dimethyl-1,3-dioxolan-4-yl)-2,2-dimethyltetrahydrofuro[2,3-d][1,3]dioxole (**12f**)

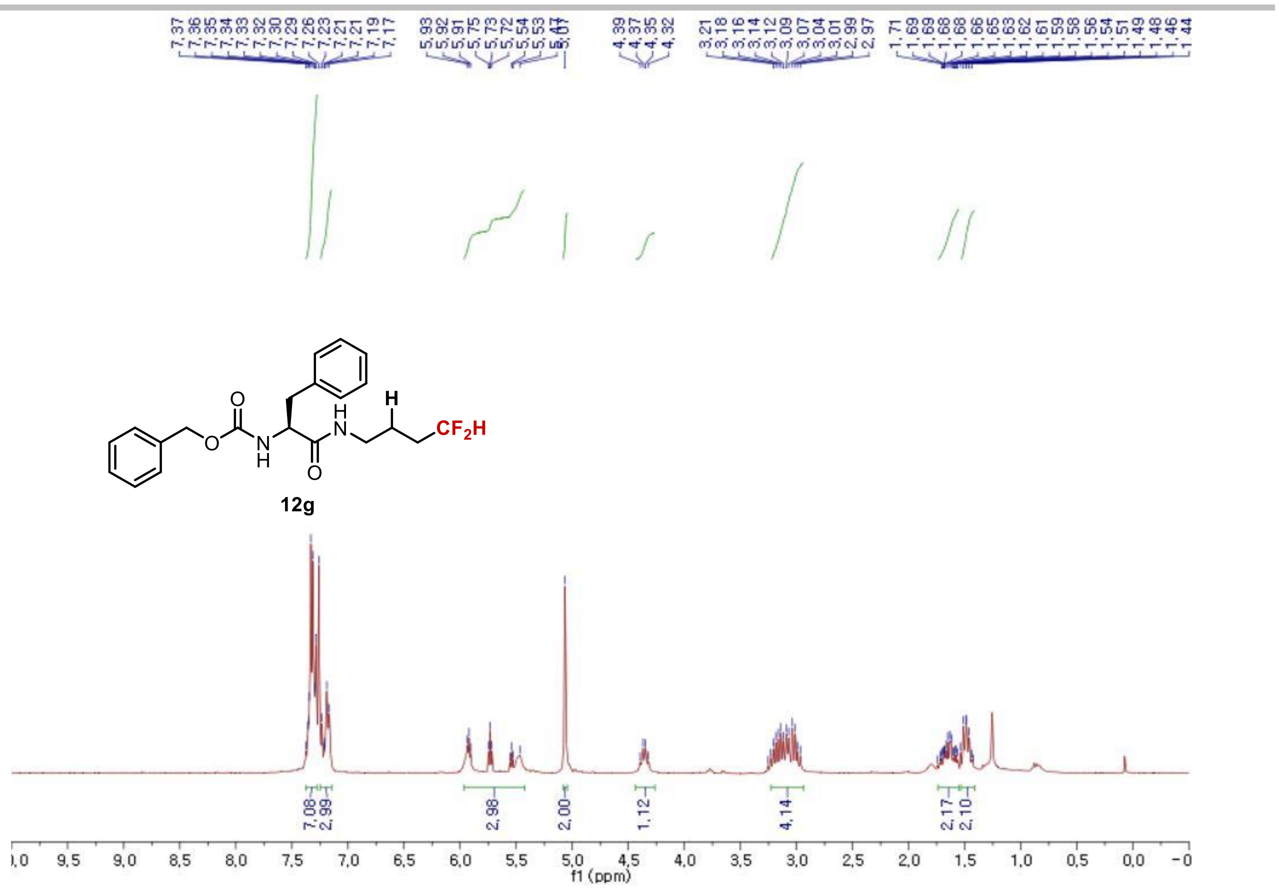

Supplementary Figure 105. ^{1}H NMR Spectrum of Benzyl (S)-(1-((4,4-difluorobutyl)amino)-1-oxo-3-phenylpropan-2-yl)carbamate (**12g**)

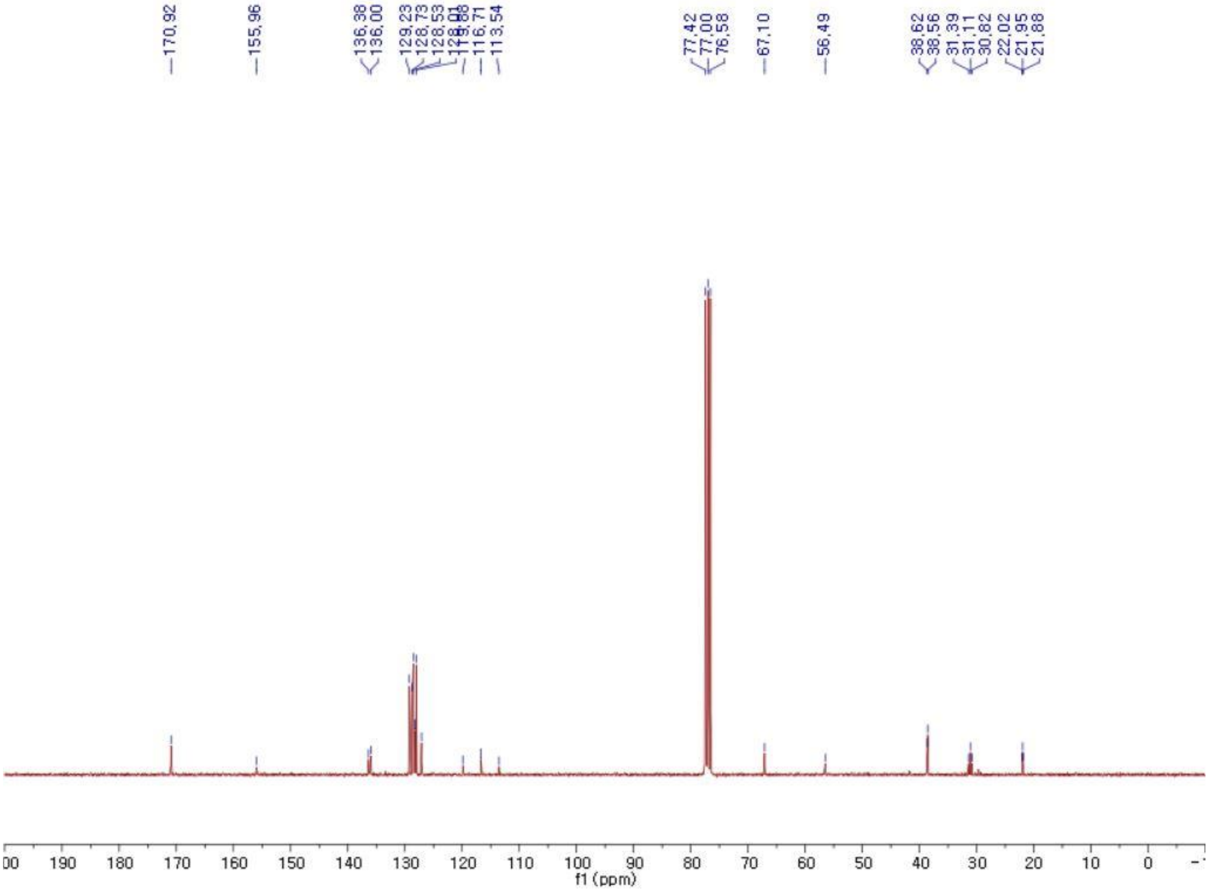

Supplementary Figure 106. ^{13}C NMR Spectrum of Benzyl (S)-(1-((4,4-difluorobutyl)amino)-1-oxo-3-phenylpropan-2-yl)carbamate (**12g**)

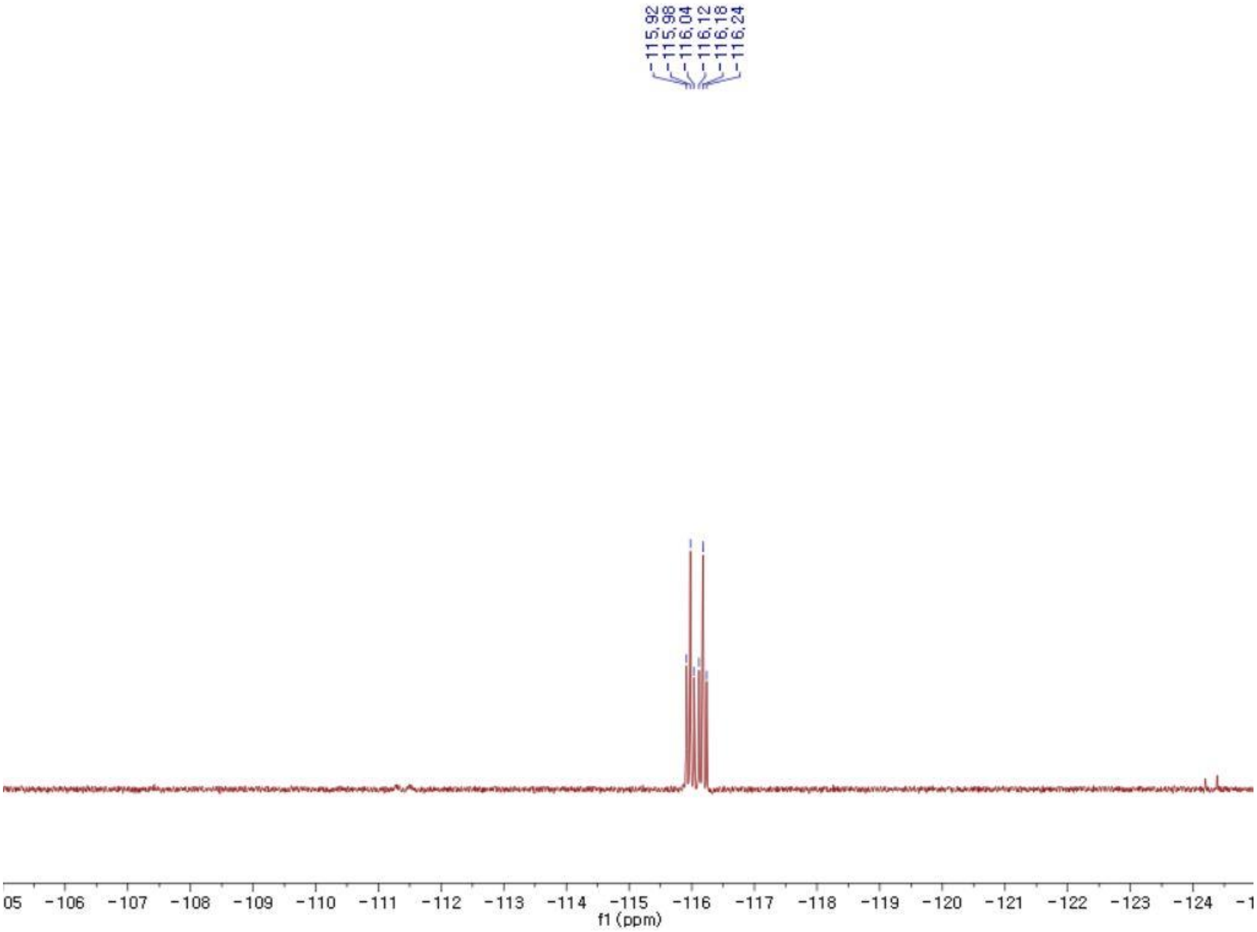

Supplementary Figure 107. ^{19}F NMR Spectrum of Benzyl (S)-(1-((4,4-difluorobutyl)amino)-1-oxo-3-phenylpropan-2-yl)carbamate (**12g**)

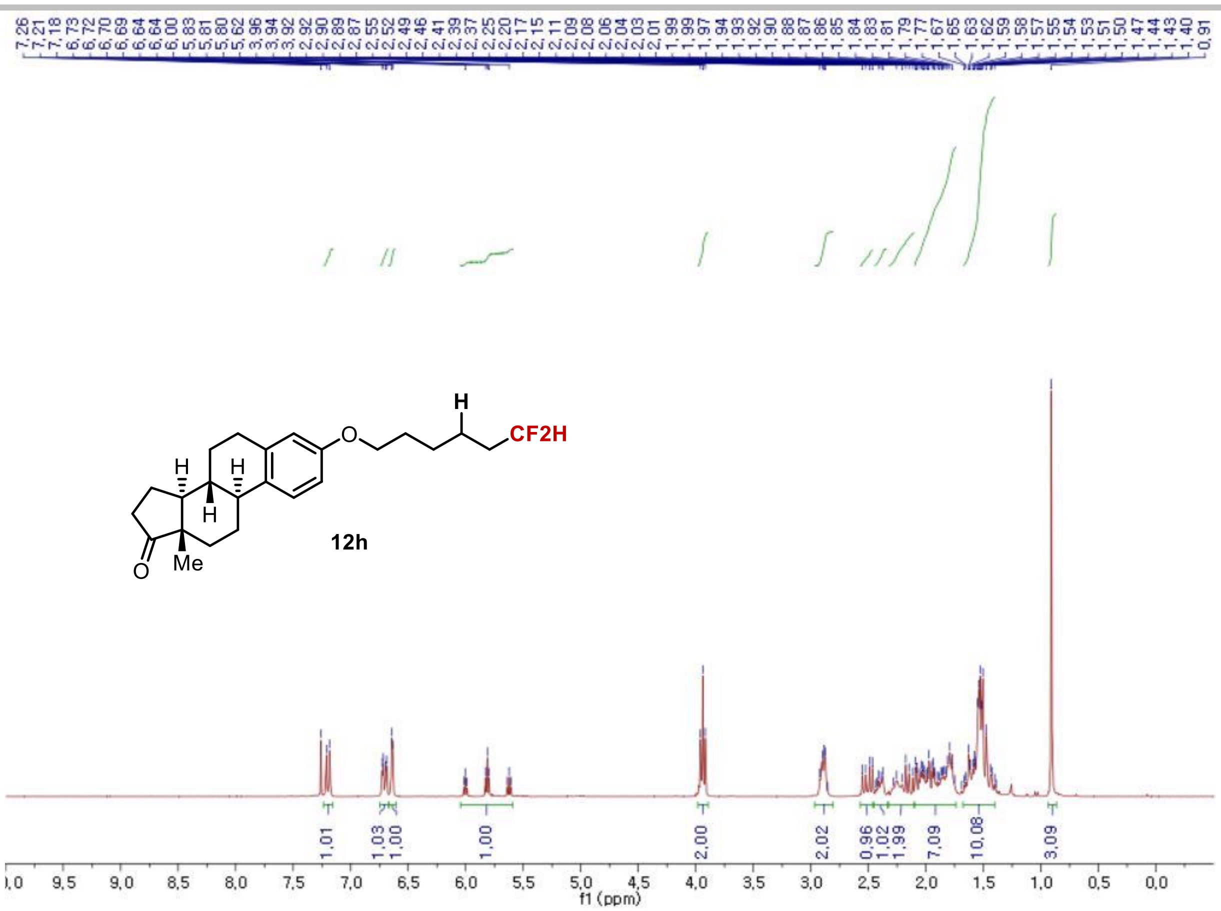

Supplementary Figure 108. ^{1}H NMR Spectrum of (8R,9S,13S,14S)-3-((6,6-difluorohexyl)oxy)-13-methyl-6,7,8,9,11,12,13,14,15,16-decahydro-17H-cyclopenta[a]phenanthren-17-onecarbamate (**12h**)

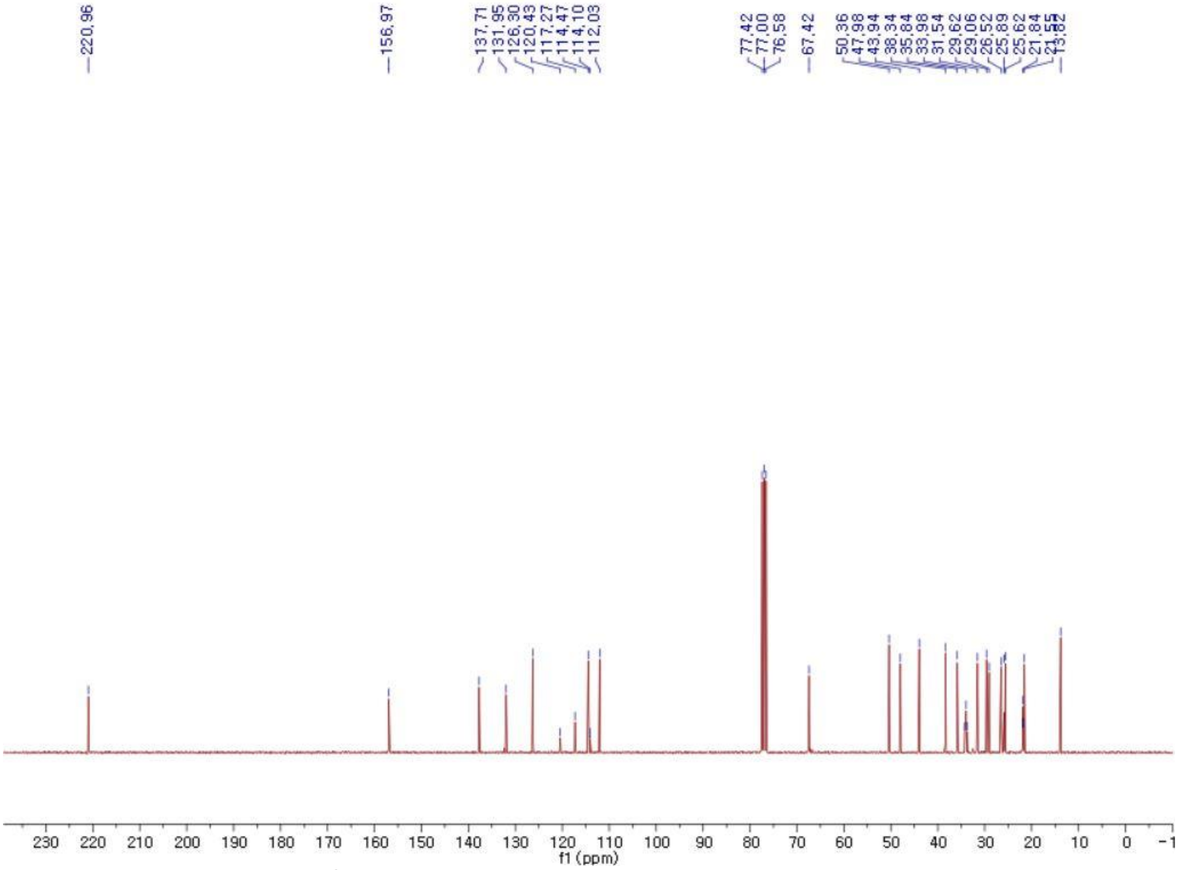

Supplementary Figure 109. ^{13}C NMR Spectrum of (8R,9S,13S,14S)-3-((6,6-difluorohexyl)oxy)-13-methyl-6,7,8,9,11,12,13,14,15,16-decahydro-17H-cyclopenta[a]phenanthren-17-onecarbamate (**12h**)

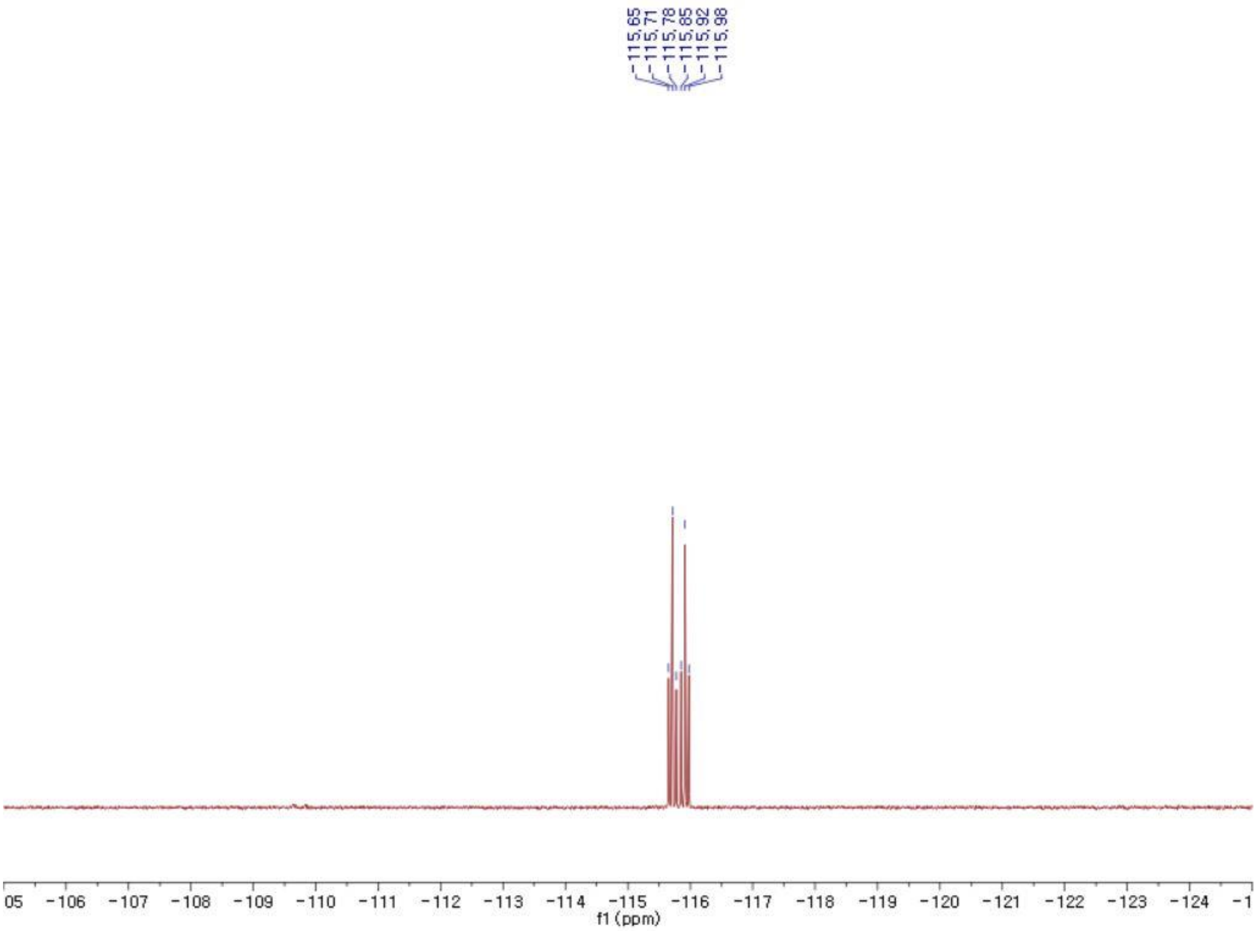

Supplementary Figure 110. ^{19}F NMR Spectrum of (8R,9S,13S,14S)-3-((6,6-difluorohexyl)oxy)-13-methyl-6,7,8,9,11,12,13,14,15,16-decahydro-17H-cyclopenta[a]phenanthren-17-onecarbamate (**12h**)

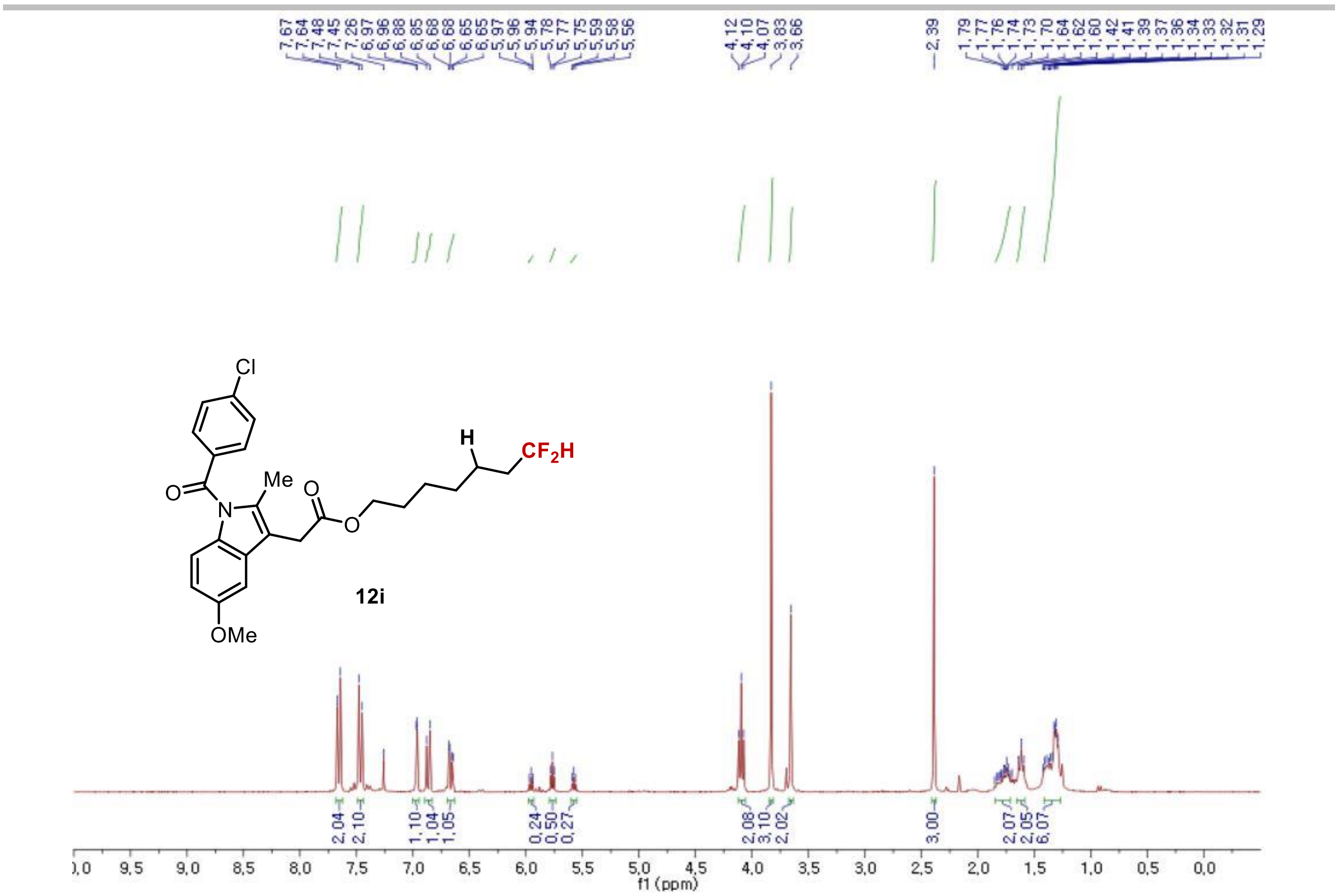

Supplementary Figure 111. ^{1}H NMR Spectrum of 7,7-Difluoroheptyl 2-(1-(4-chlorobenzoyl)-5-methoxy-2-methyl-1H-indol-3-yl)acetate (**12i**)

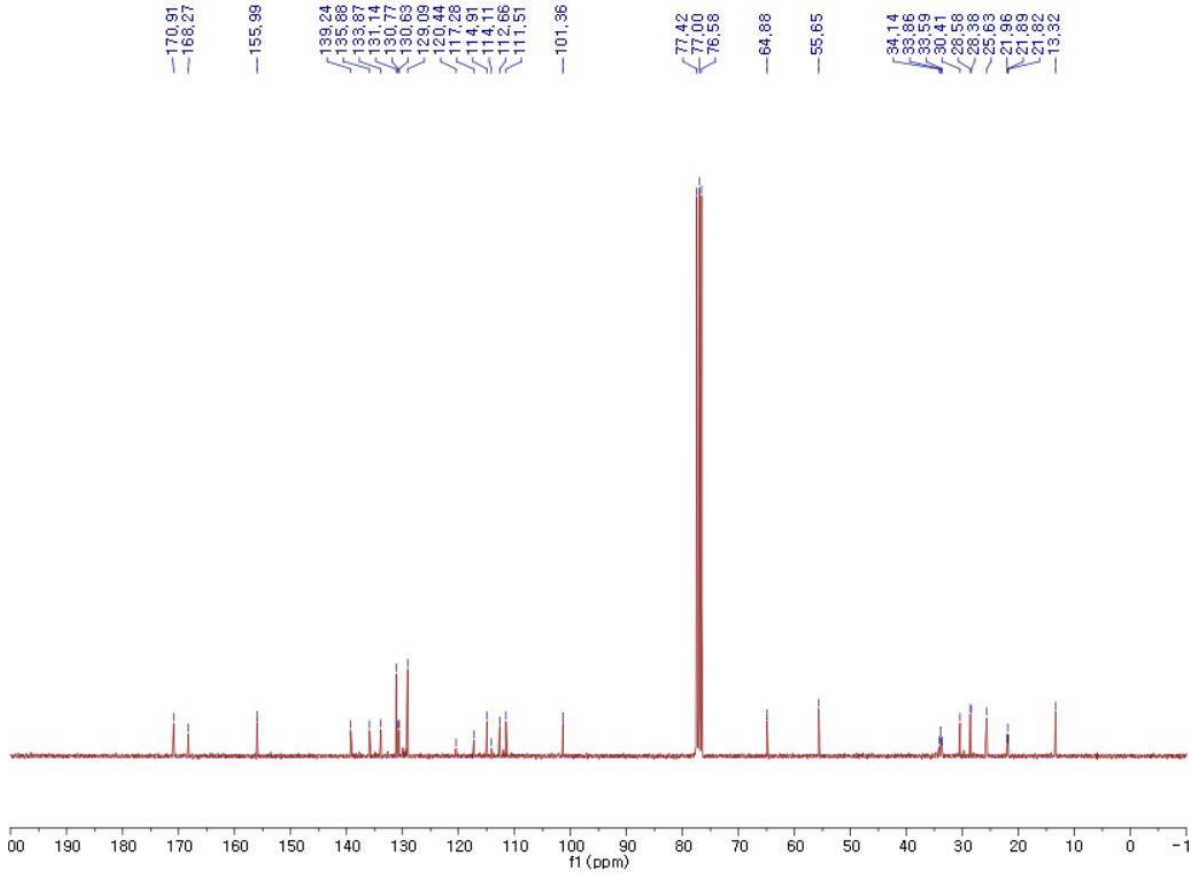

Supplementary Figure 112. ^{13}C NMR Spectrum of 7,7-Difluoroheptyl 2-(1-(4-chlorobenzoyl)-5-methoxy-2-methyl-1H-indol-3-yl)acetate (**12i**)

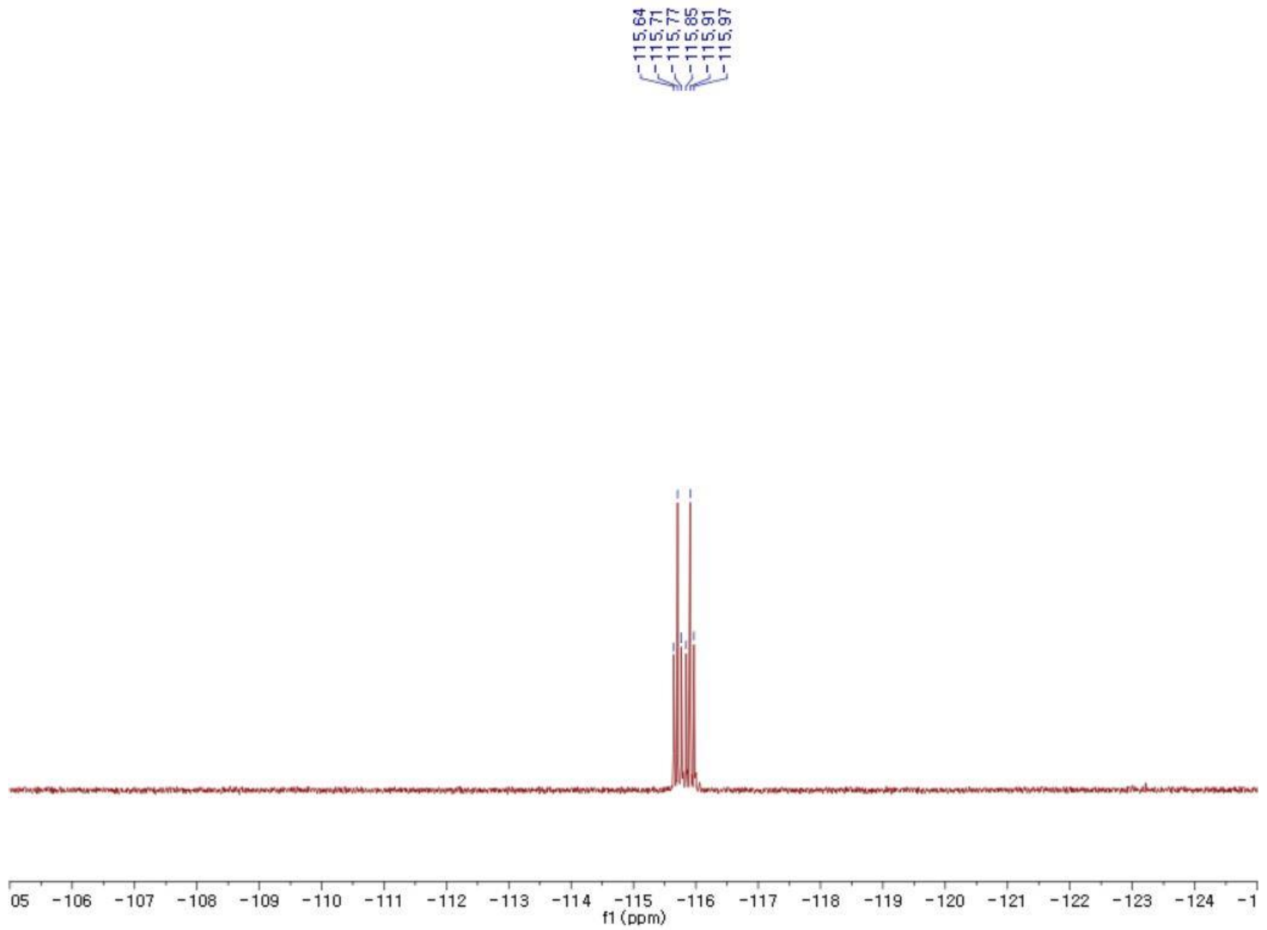

Supplementary Figure 113. ^{19}F NMR Spectrum of 7,7-Difluoroheptyl 2-(1-(4-chlorobenzoyl)-5-methoxy-2-methyl-1H-indol-3-yl)acetate (**12i**)

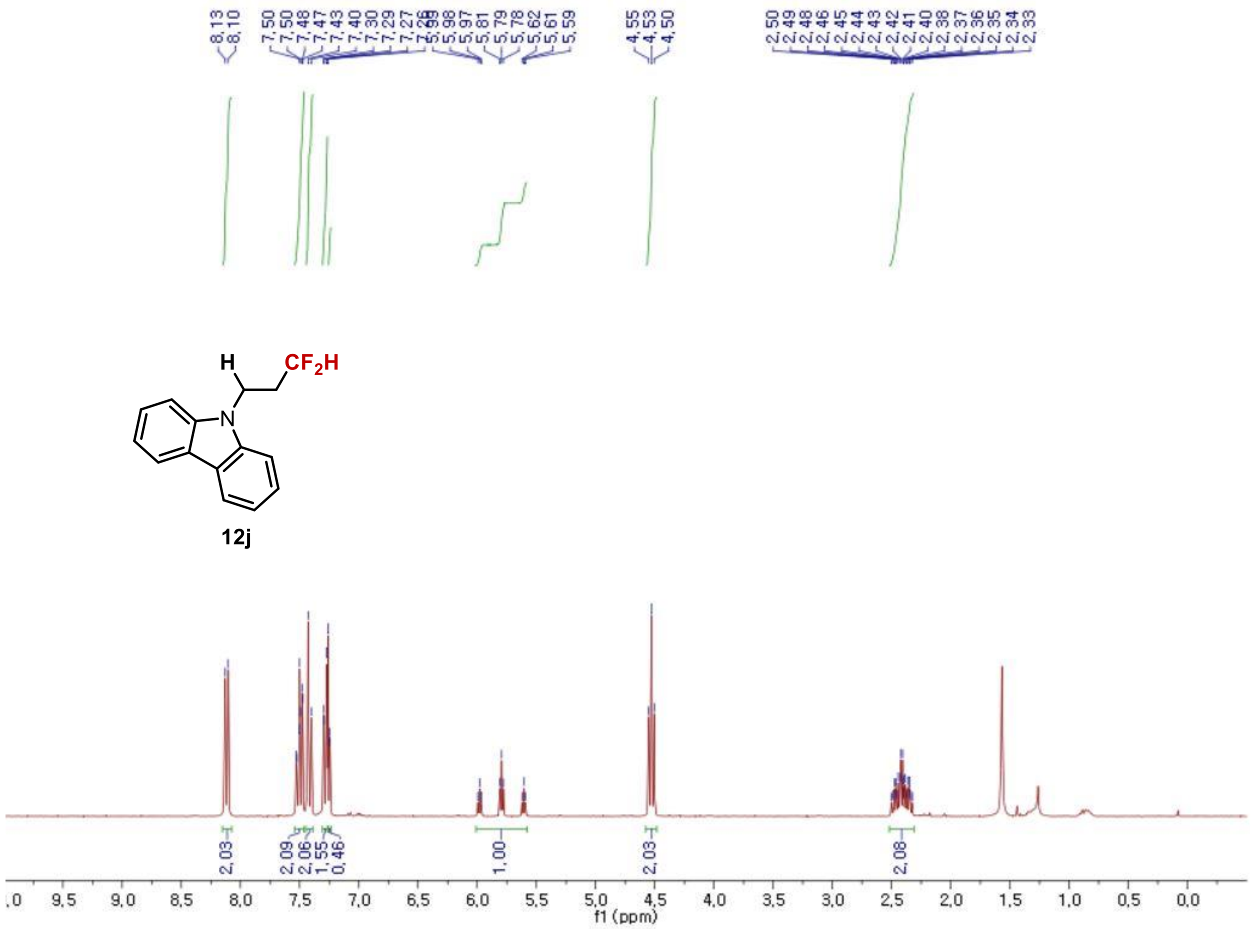

Supplementary Figure 114. ^{1}H NMR Spectrum of 9-(3,3-Difluoropropyl)-9H-carbazole (**12j**)

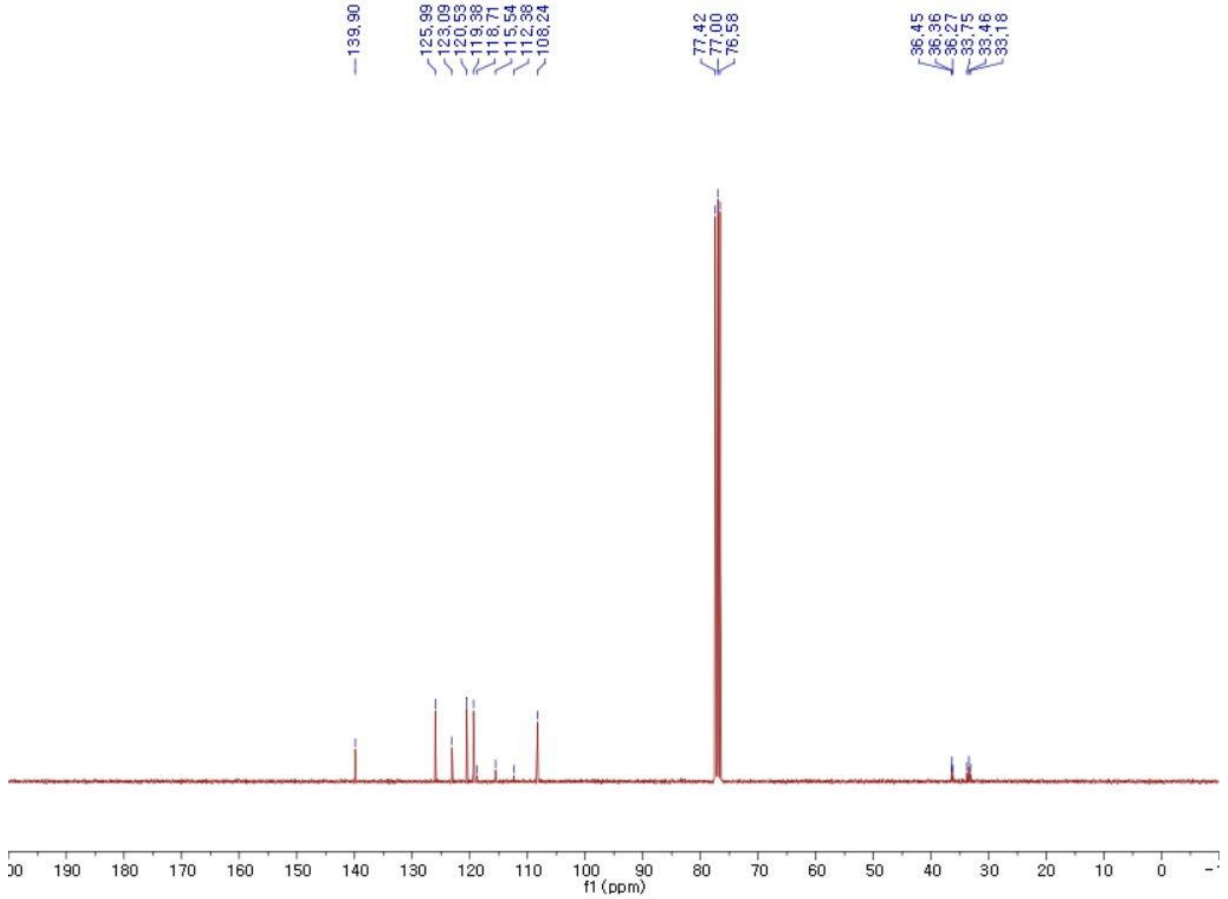

Supplementary Figure 115. ^{13}C NMR Spectrum of 9-(3,3-Difluoropropyl)-9H-carbazole (**12j**)

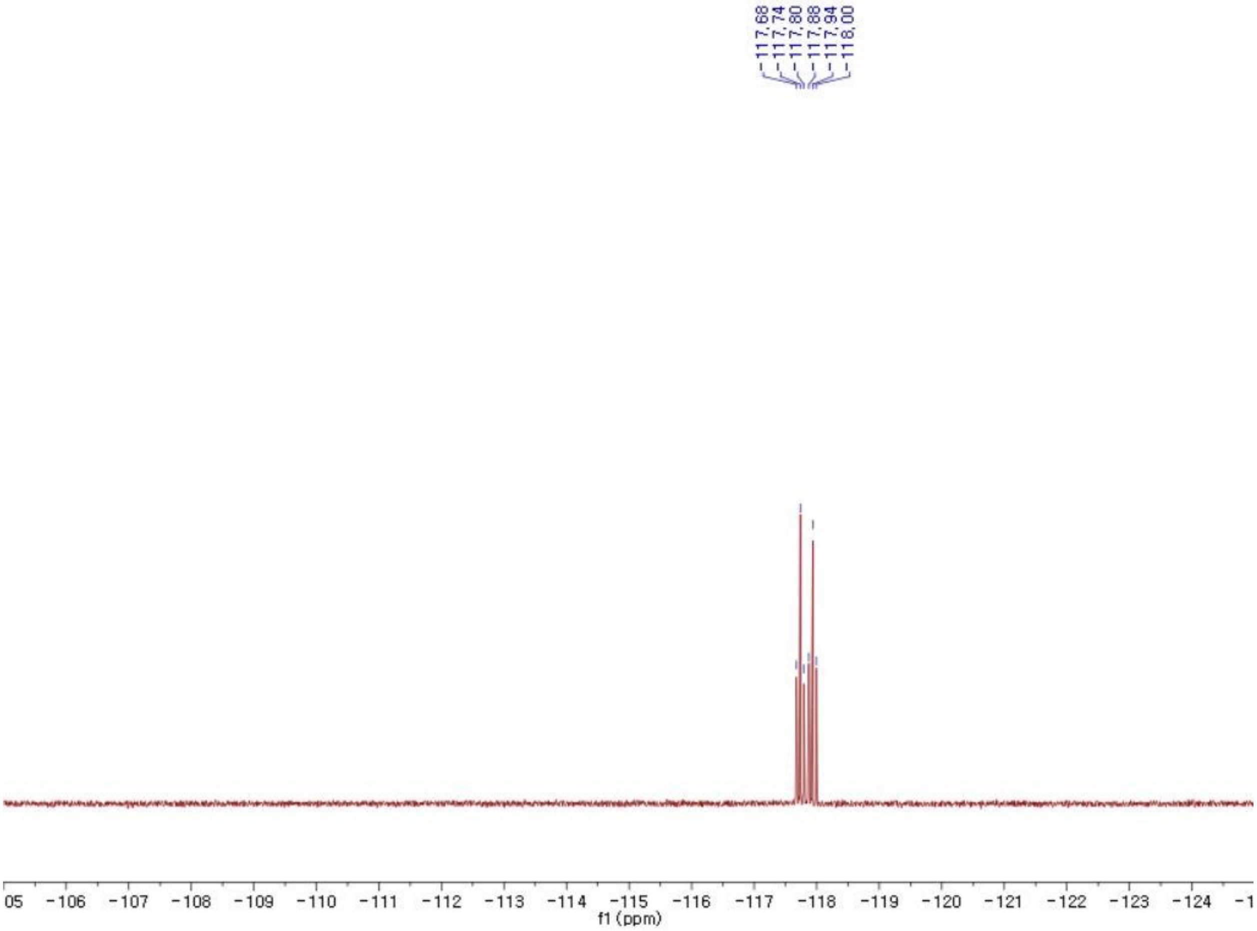

Supplementary Figure 116. ^{19}F NMR Spectrum of 9-(3,3-Difluoropropyl)-9H-carbazole (**12j**)

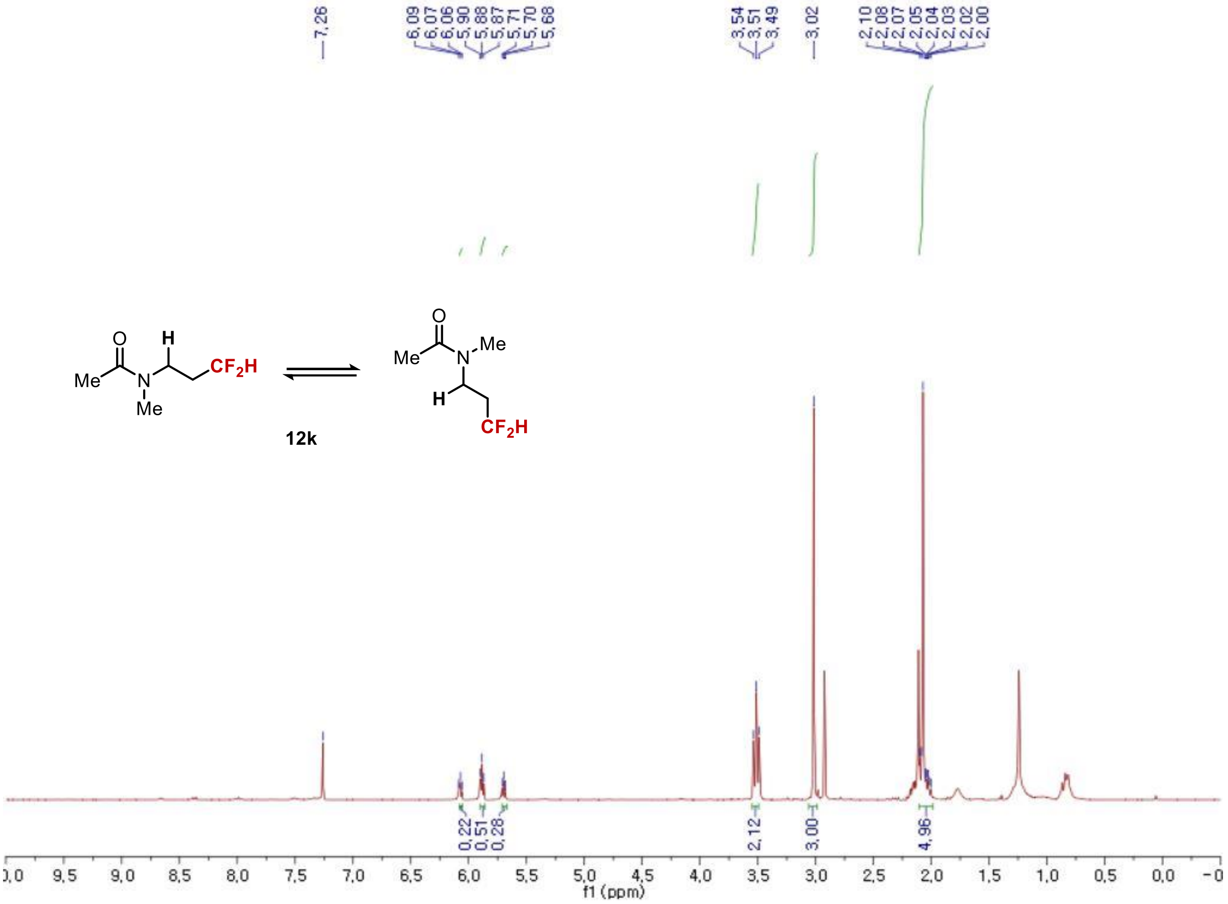

Supplementary Figure 117. ^{1}H NMR Spectrum of N-(3,3-Difluoropropyl)-N-methylacetamide (**12k**)

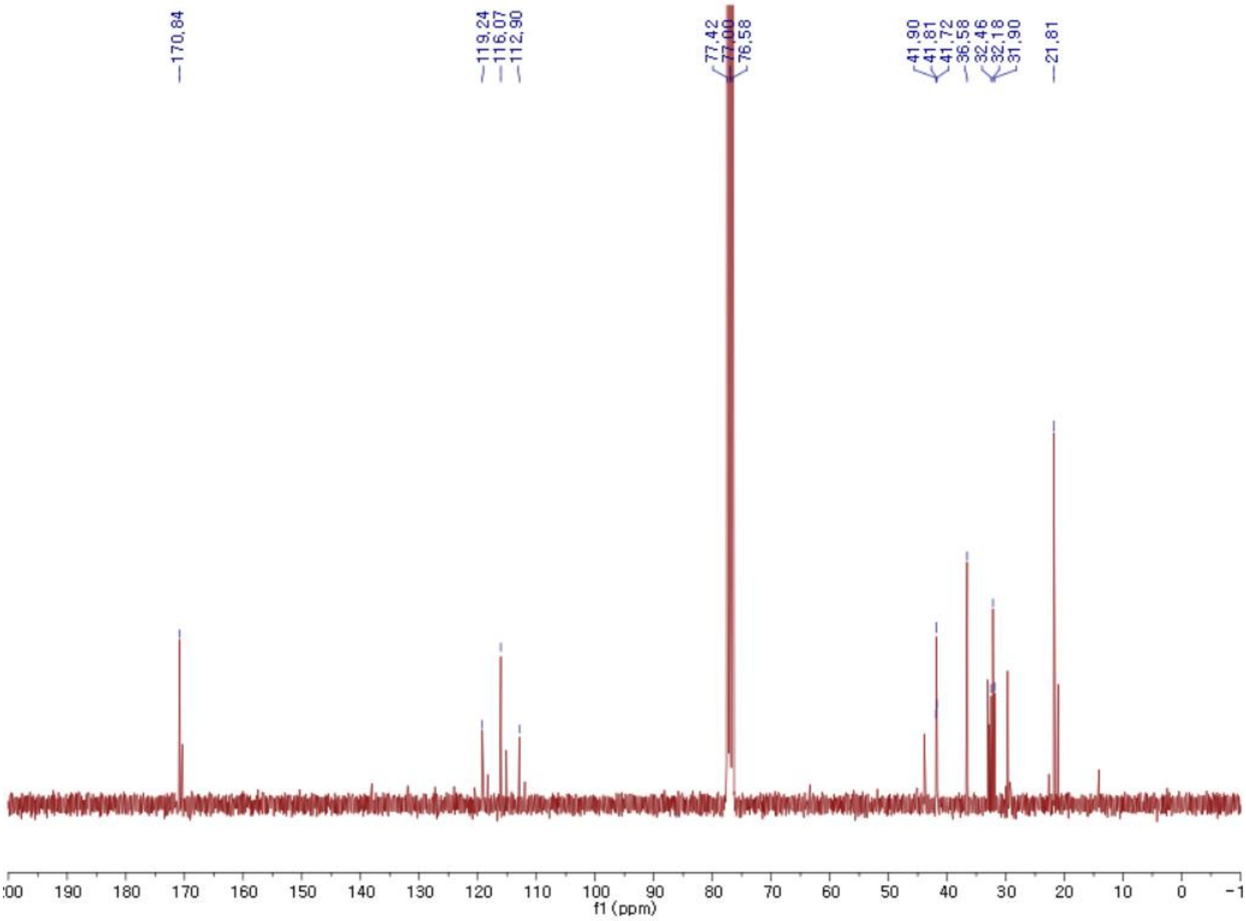

Supplementary Figure 118. ^{13}C NMR Spectrum of N-(3,3-Difluoropropyl)-N-methylacetamide (**12k**)

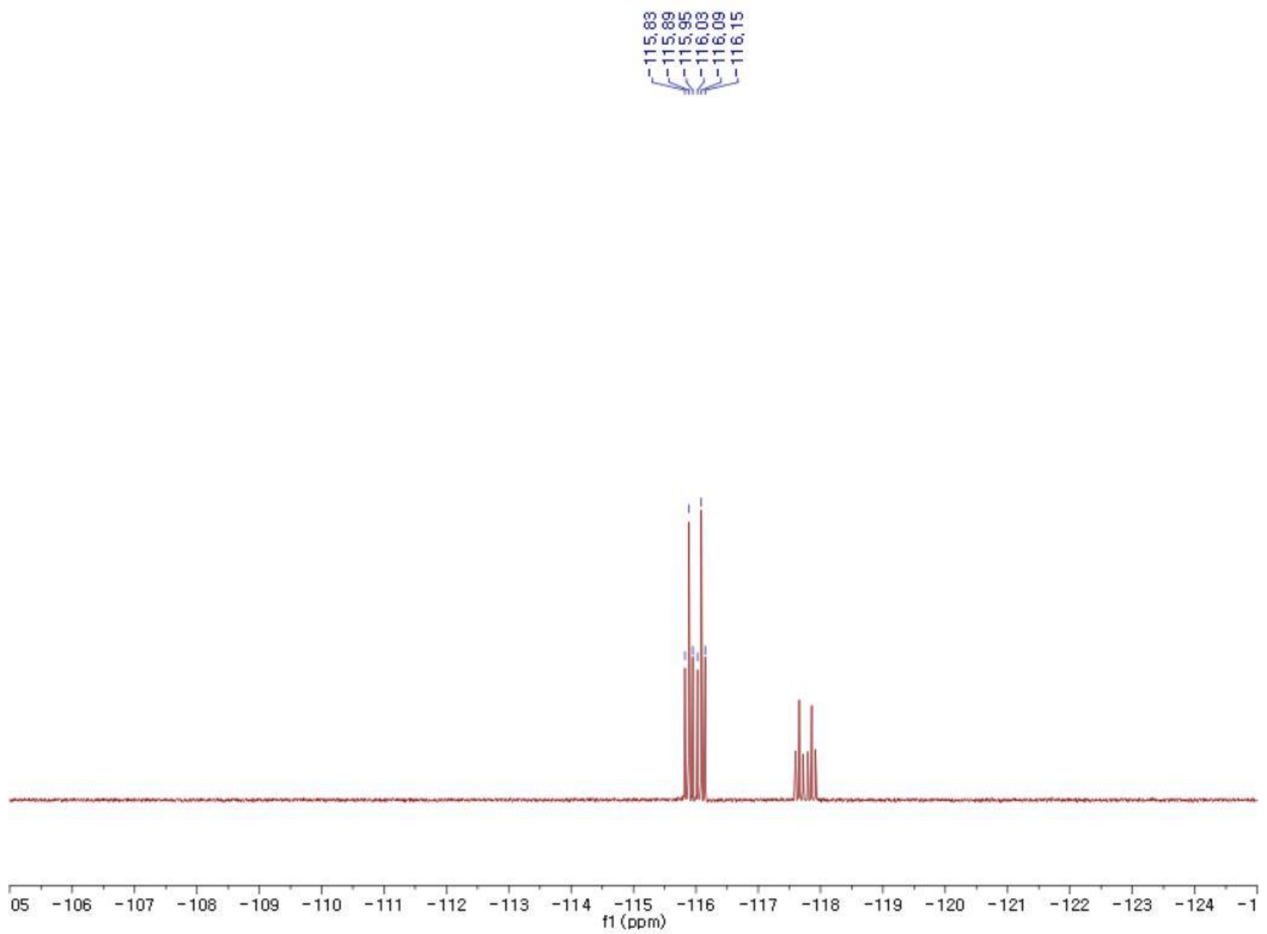

Supplementary Figure 119. ^{19}F NMR Spectrum of N-(3,3-Difluoropropyl)-N-methylacetamide (**12k**)

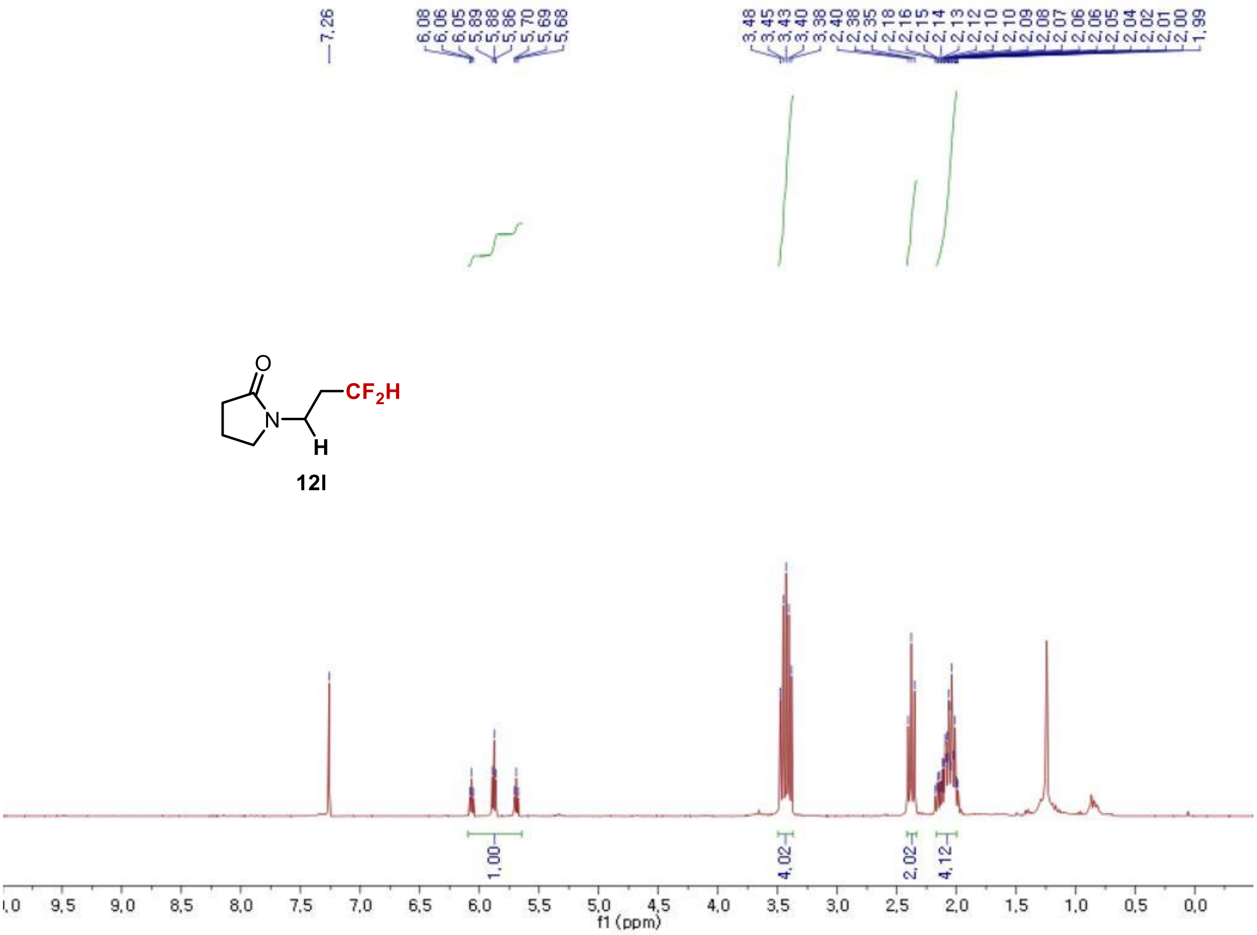

Supplementary Figure 120. ^{1}H NMR Spectrum of 1-(3,3-Difluoropropyl)pyrrolidin-2-one (**12l**)

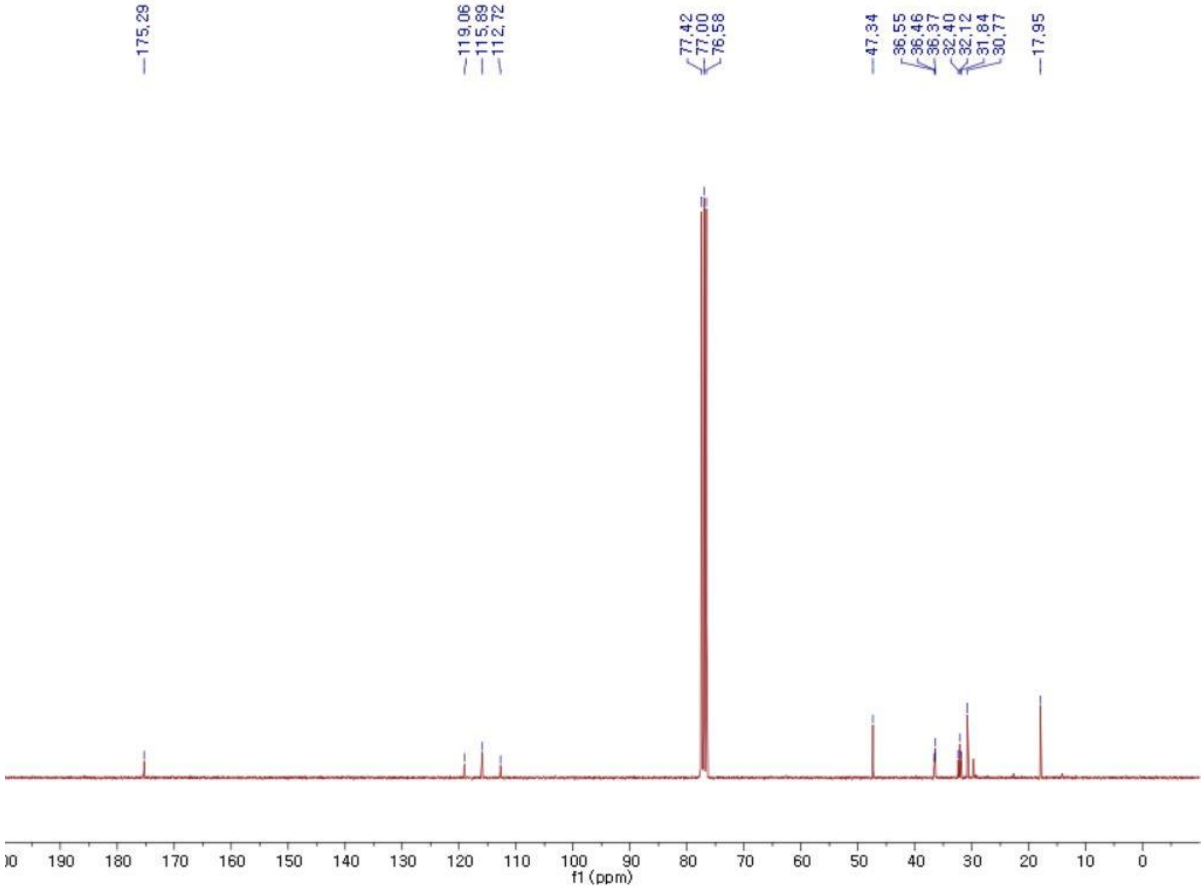

Supplementary Figure 121. ^{13}C NMR Spectrum of 1-(3,3-Difluoropropyl)pyrrolidin-2-one (**12l**)

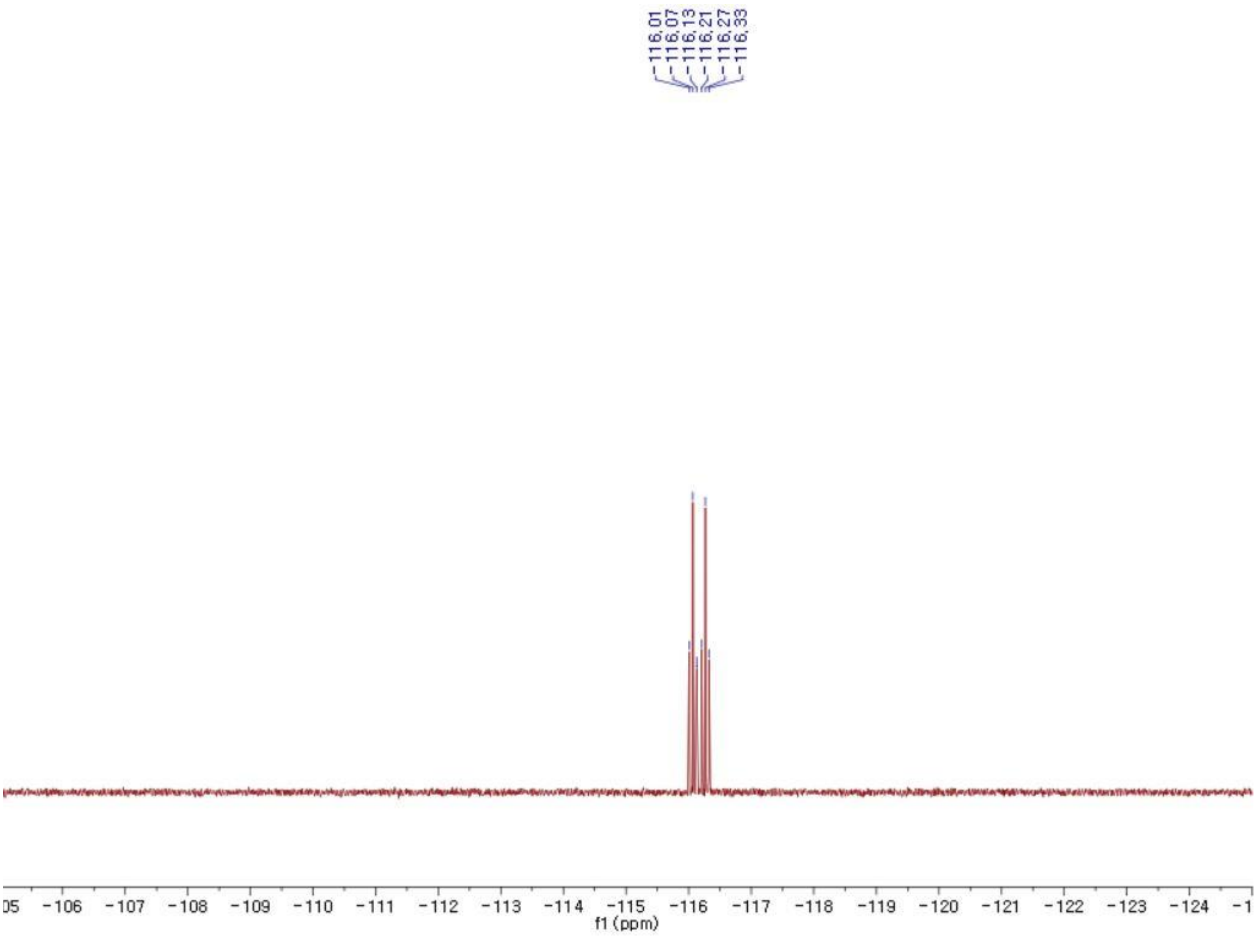

Supplementary Figure 122. ^{19}F NMR Spectrum of 1-(3,3-Difluoropropyl)pyrrolidin-2-one (**12l**)

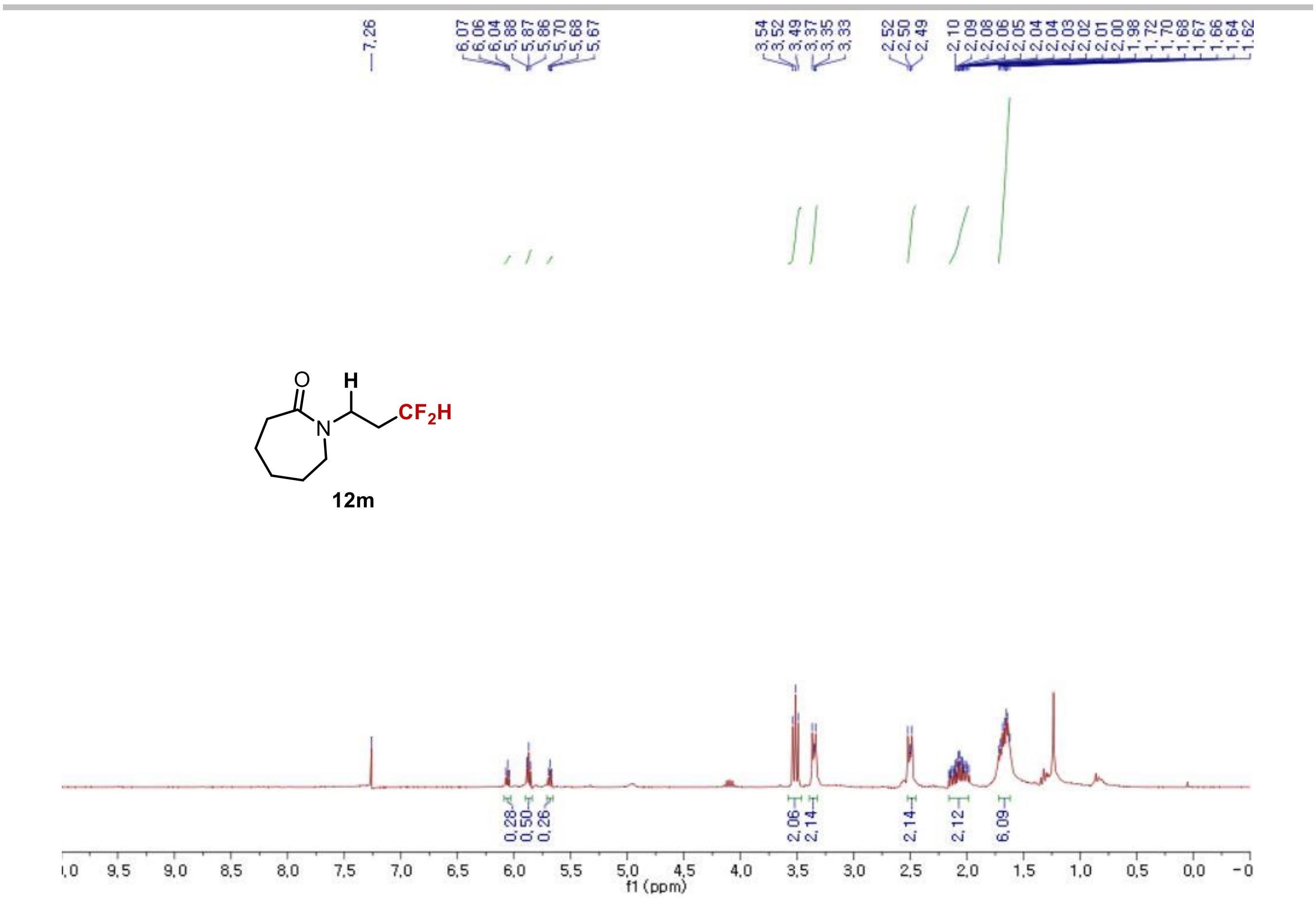

Supplementary Figure 123. ^{1}H NMR Spectrum of 1-(3,3-Difluoropropyl)azepan-2-one (**12m**)

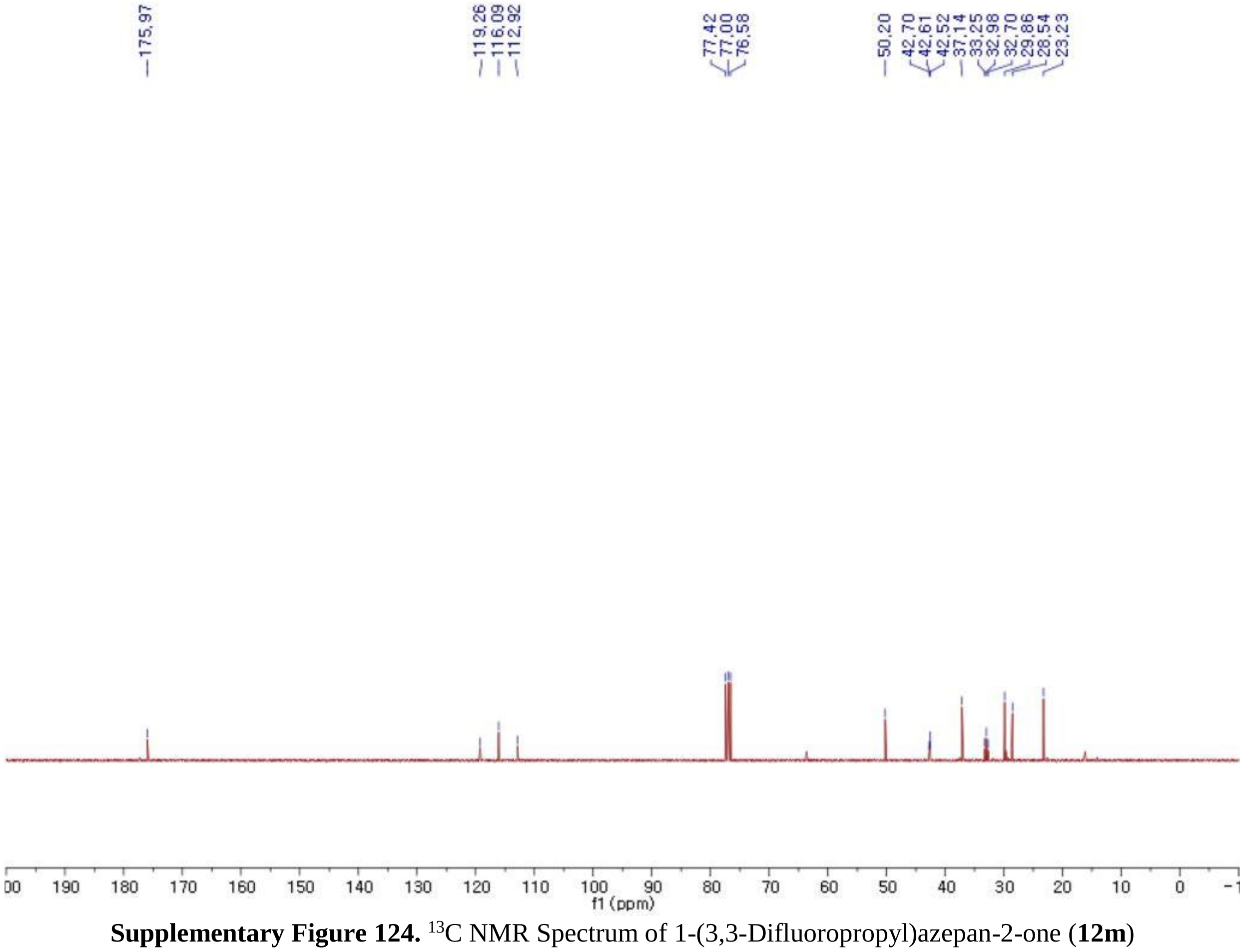

Supplementary Figure 124. ^{13}C NMR Spectrum of 1-(3,3-Difluoropropyl)azepan-2-one (**12m**)

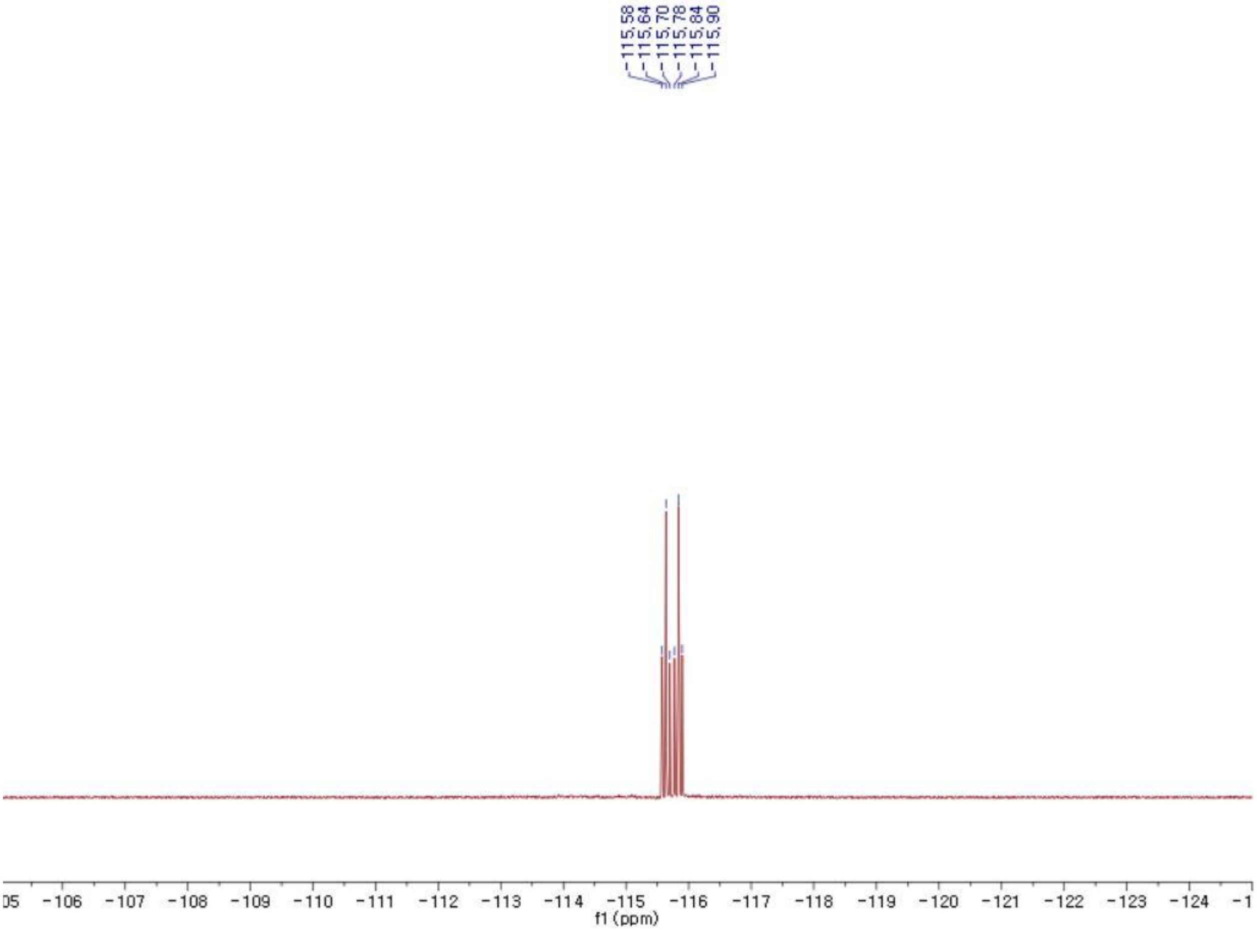

Supplementary Figure 125. ^{19}F NMR Spectrum of 1-(3,3-Difluoropropyl)azepan-2-one (**12m**)

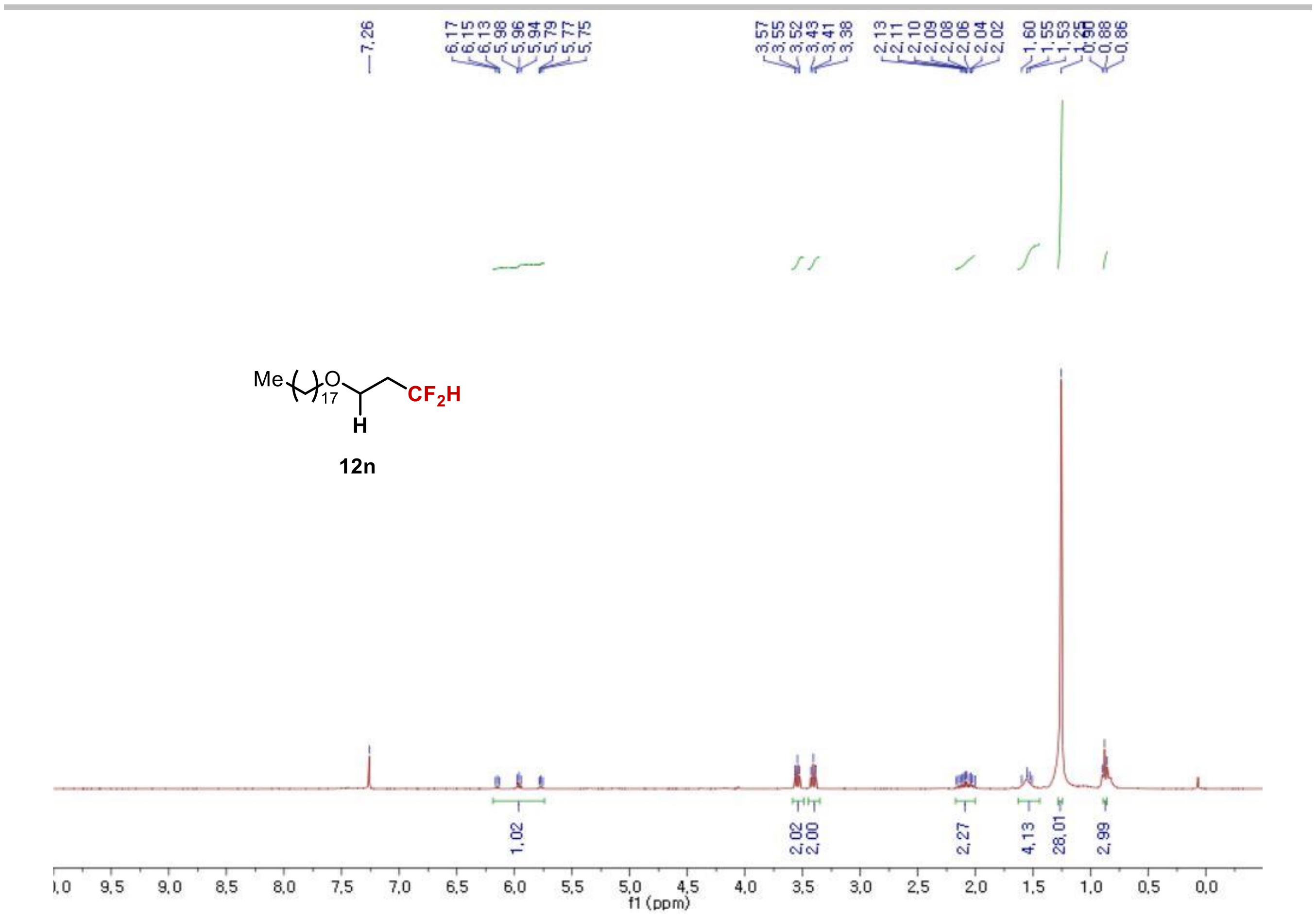

Supplementary Figure 126. ^{1}H NMR Spectrum of 1-(3,3-Difluoropropoxy)octadecane (**12n**)

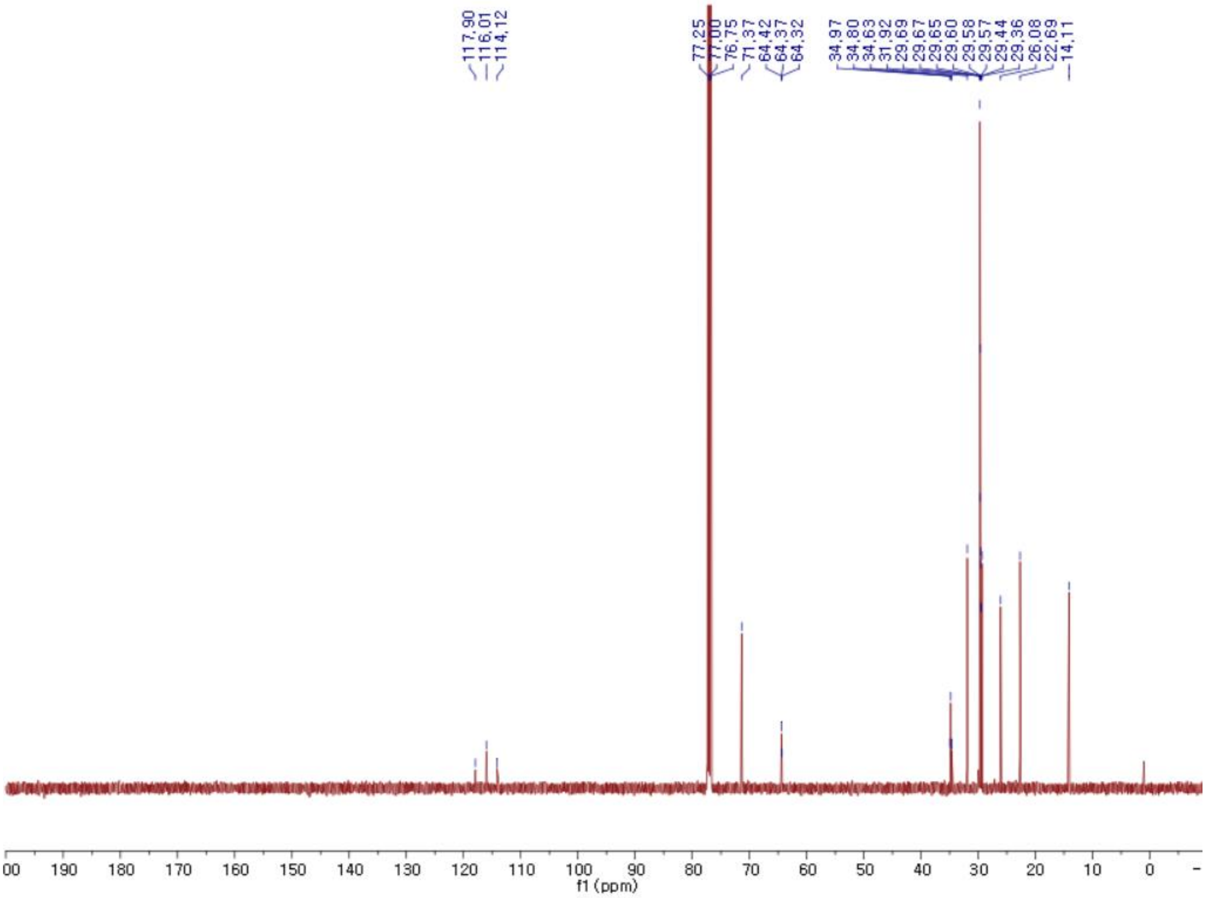

Supplementary Figure 127. ^{13}C NMR Spectrum of 1-(3,3-Difluoropropoxy)octadecane (**12n**)

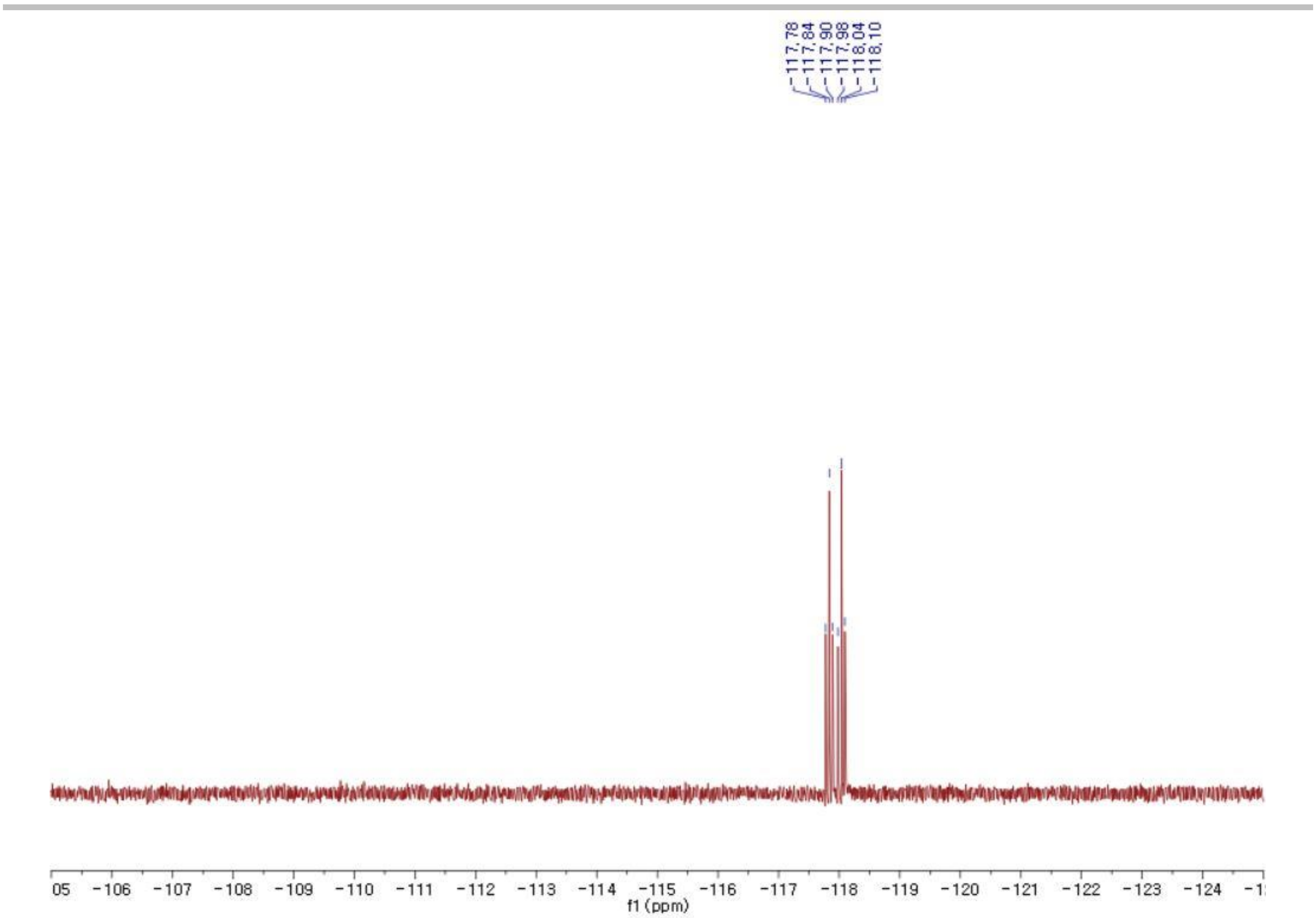

Supplementary Figure 128. ^{19}F NMR Spectrum of 1-(3,3-Difluoropropoxy)octadecane (**12n**)

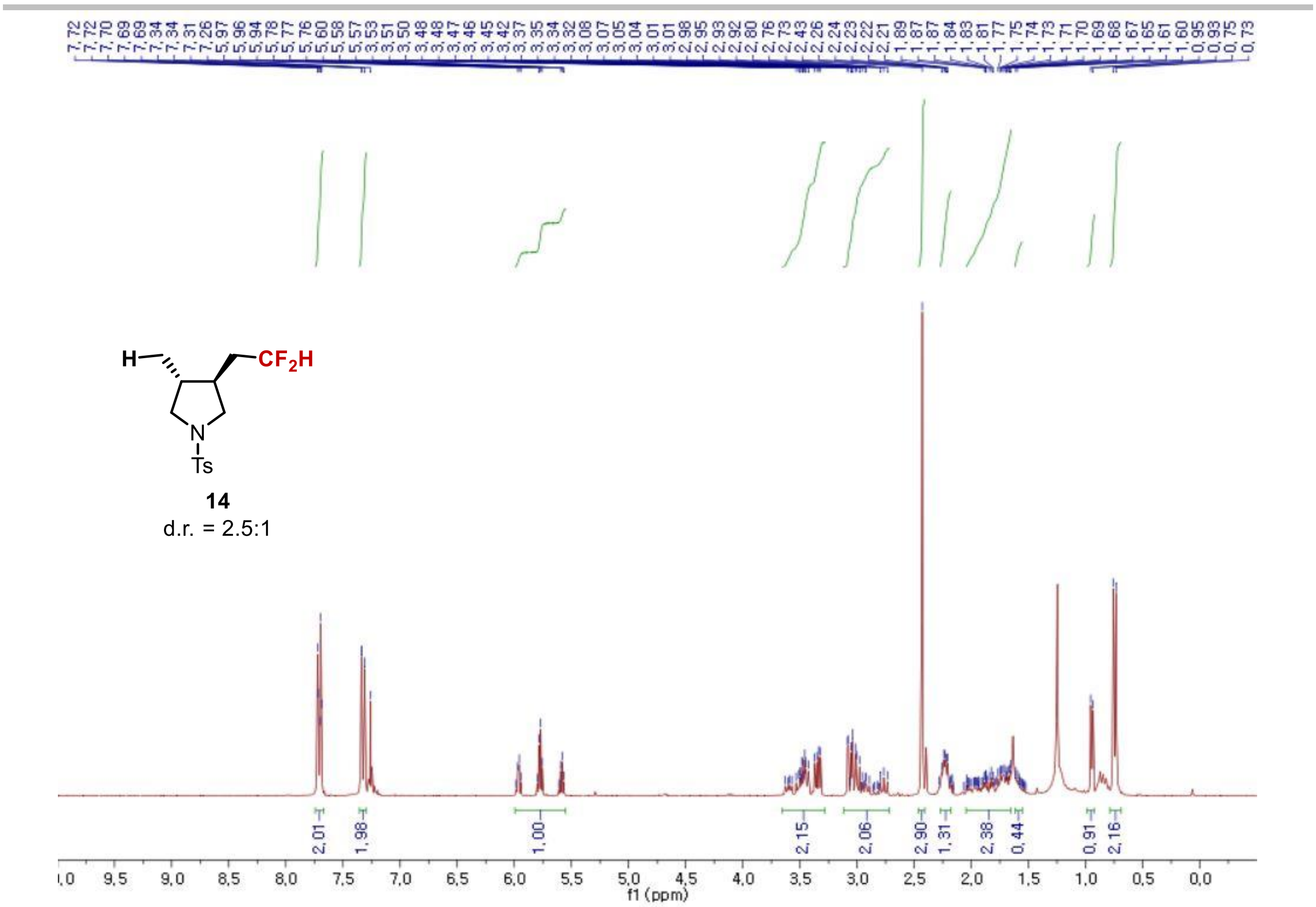

Supplementary Figure 129. ^{1}H NMR Spectrum of (3R,4R)-3-(2,2-Difluoroethyl)-4-methyl-1-tosylpyrrolidine (**14**)

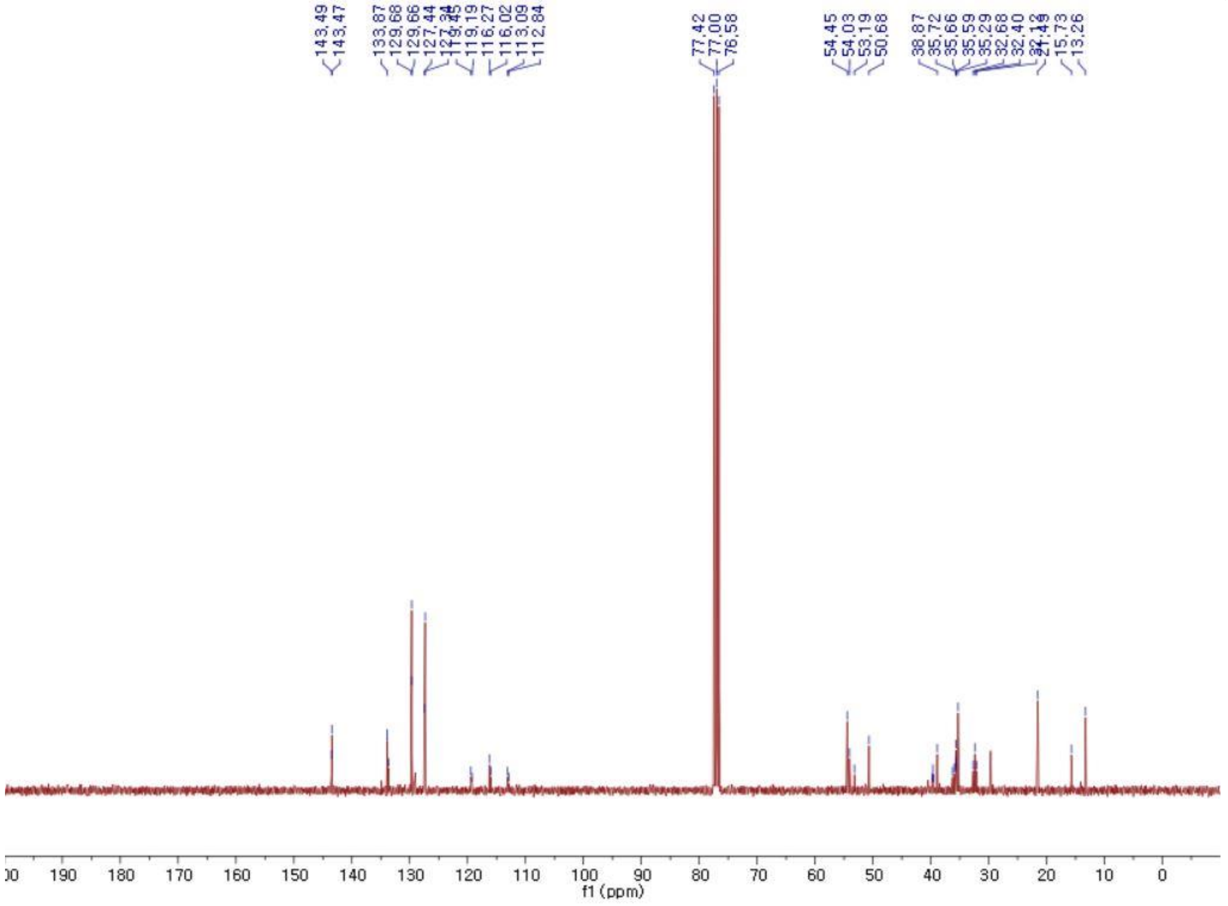

Supplementary Figure 130. ^{13}C NMR Spectrum of (3R,4R)-3-(2,2-Difluoroethyl)-4-methyl-1-tosylpyrrolidine (**14**)

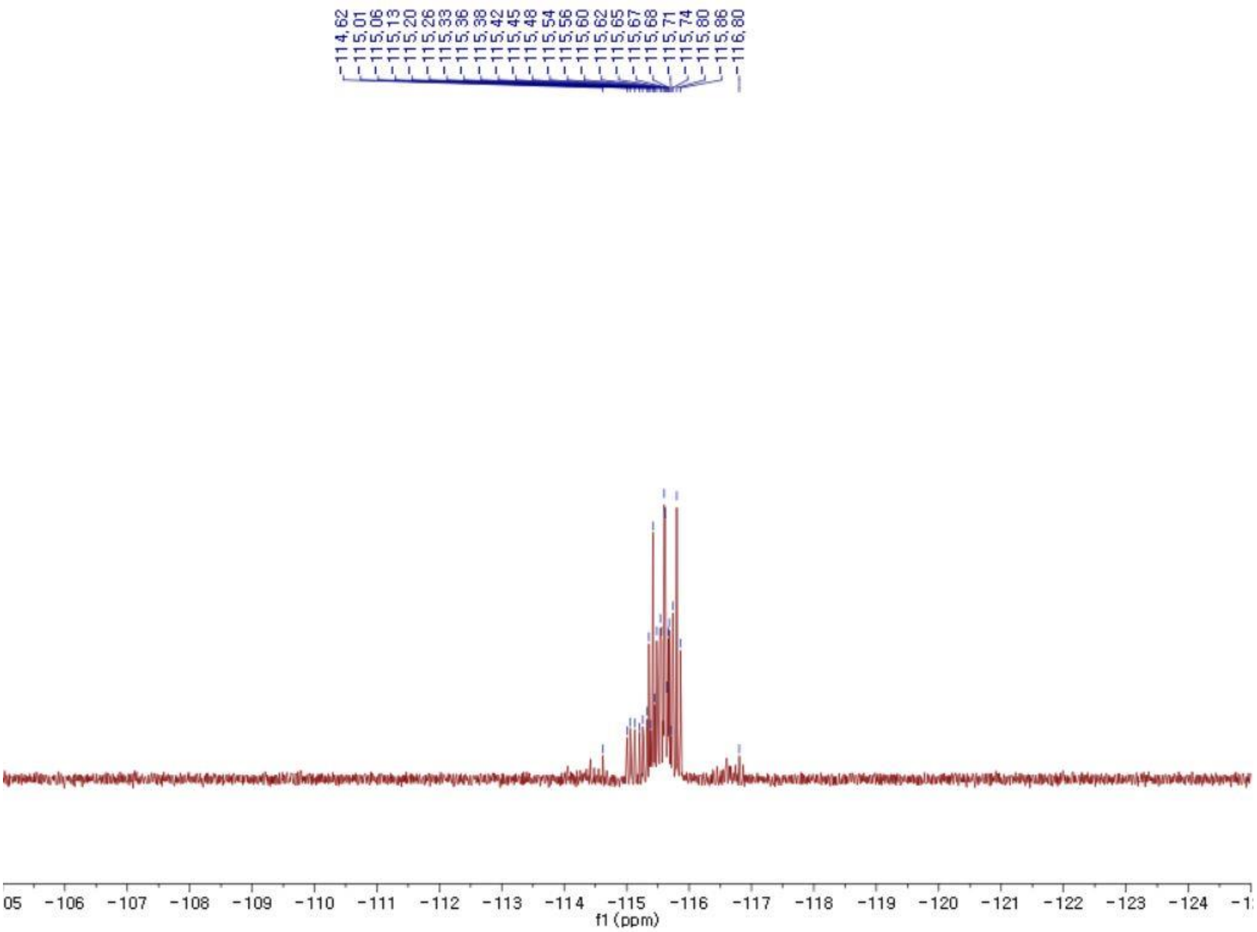

Supplementary Figure 131. ^{19}F NMR Spectrum of (3R,4R)-3-(2,2-Difluoroethyl)-4-methyl-1-tosylpyrrolidine (**14**)

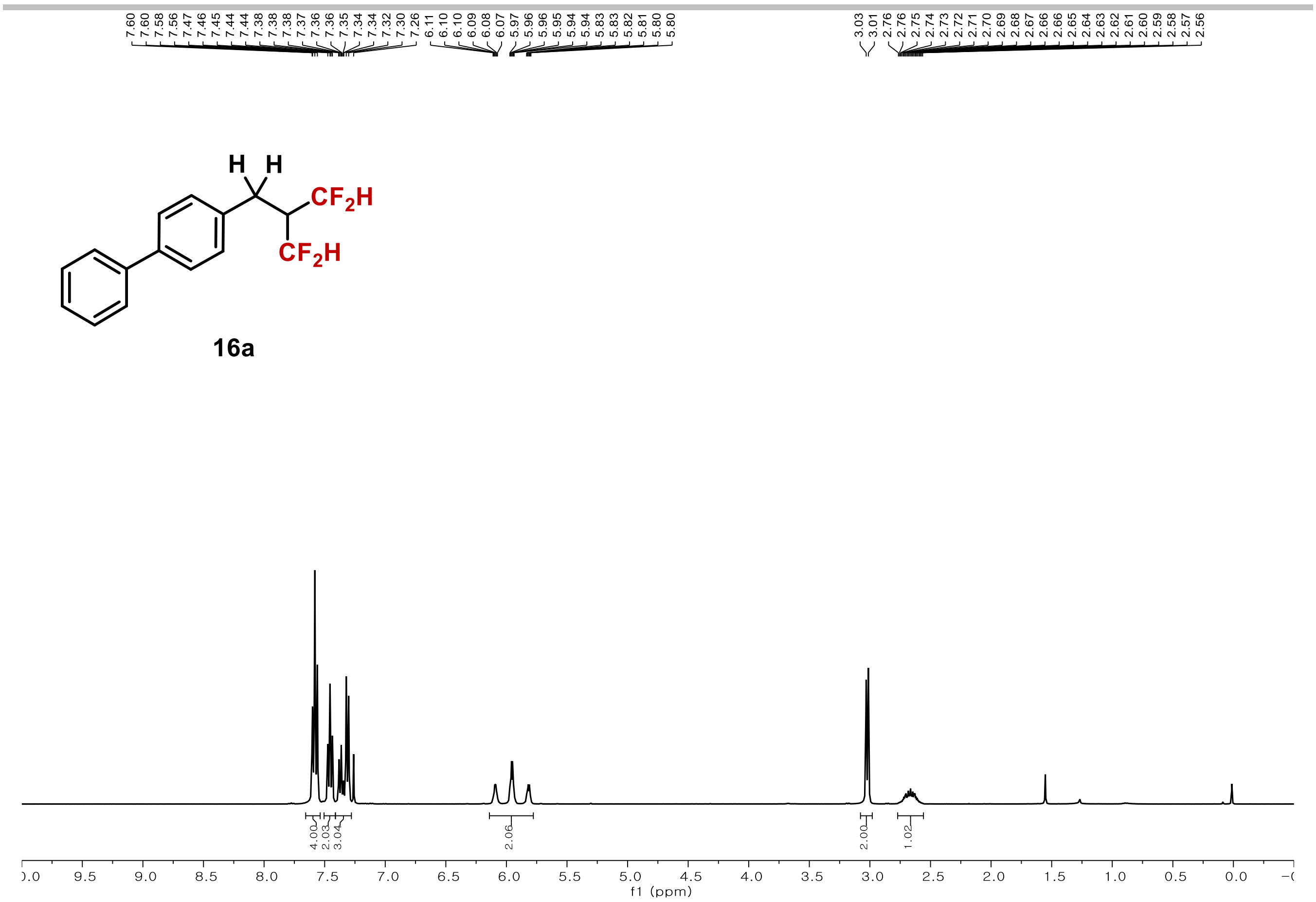

Supplementary Figure 132. ^{1}H NMR Spectrum of 4-(2-(Difluoromethyl)-3,3-difluoropropyl)-1,1'-biphenyl (**16a**)

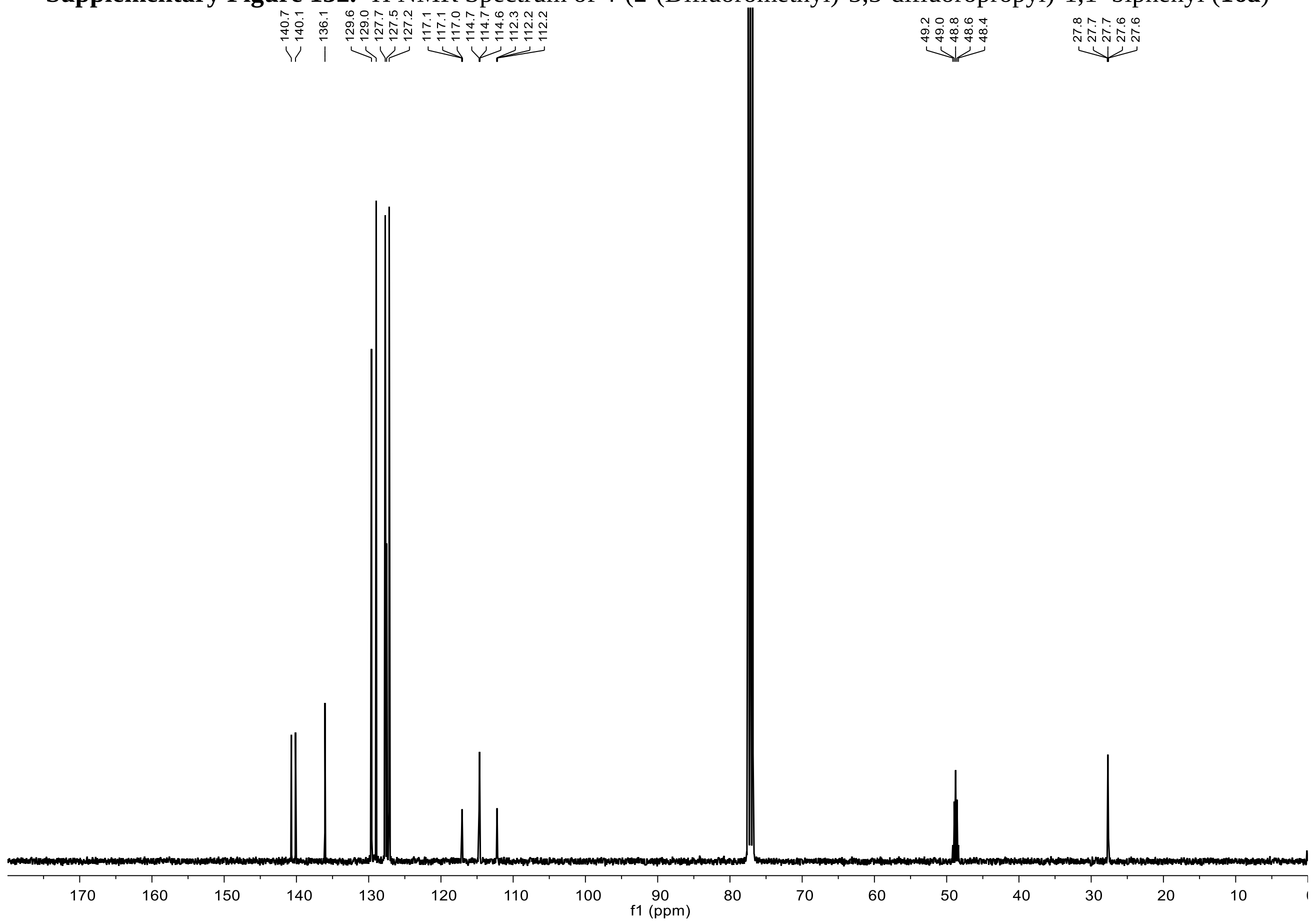

Supplementary Figure 133. ^{13}C NMR Spectrum of 4-(2-(Difluoromethyl)-3,3-difluoropropyl)-1,1'-biphenyl (**16a**)

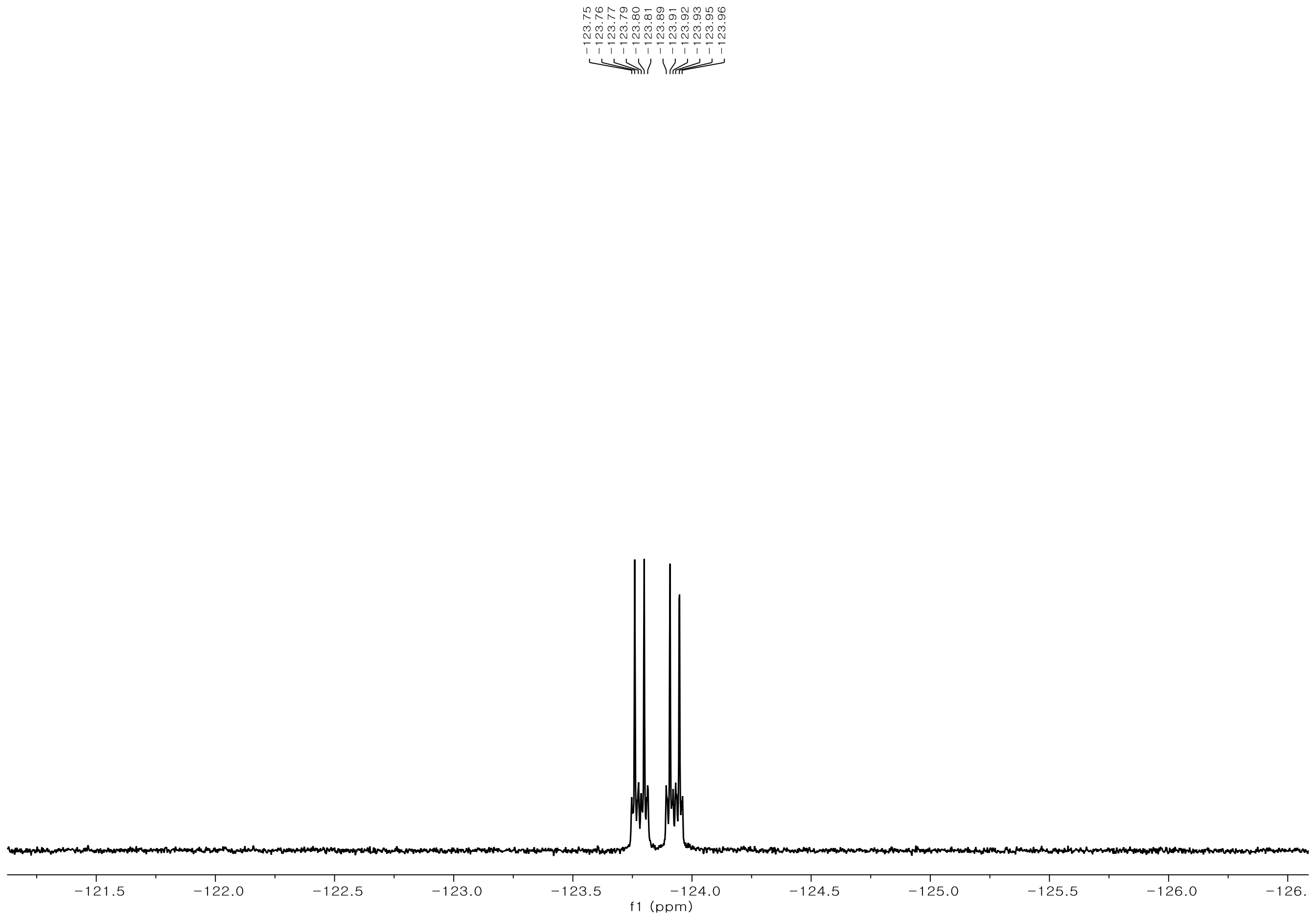

Supplementary Figure 134. ^{19}F NMR Spectrum of 4-(2-(Difluoromethyl)-3,3-difluoropropyl)-1,1'-biphenyl (**16a**)

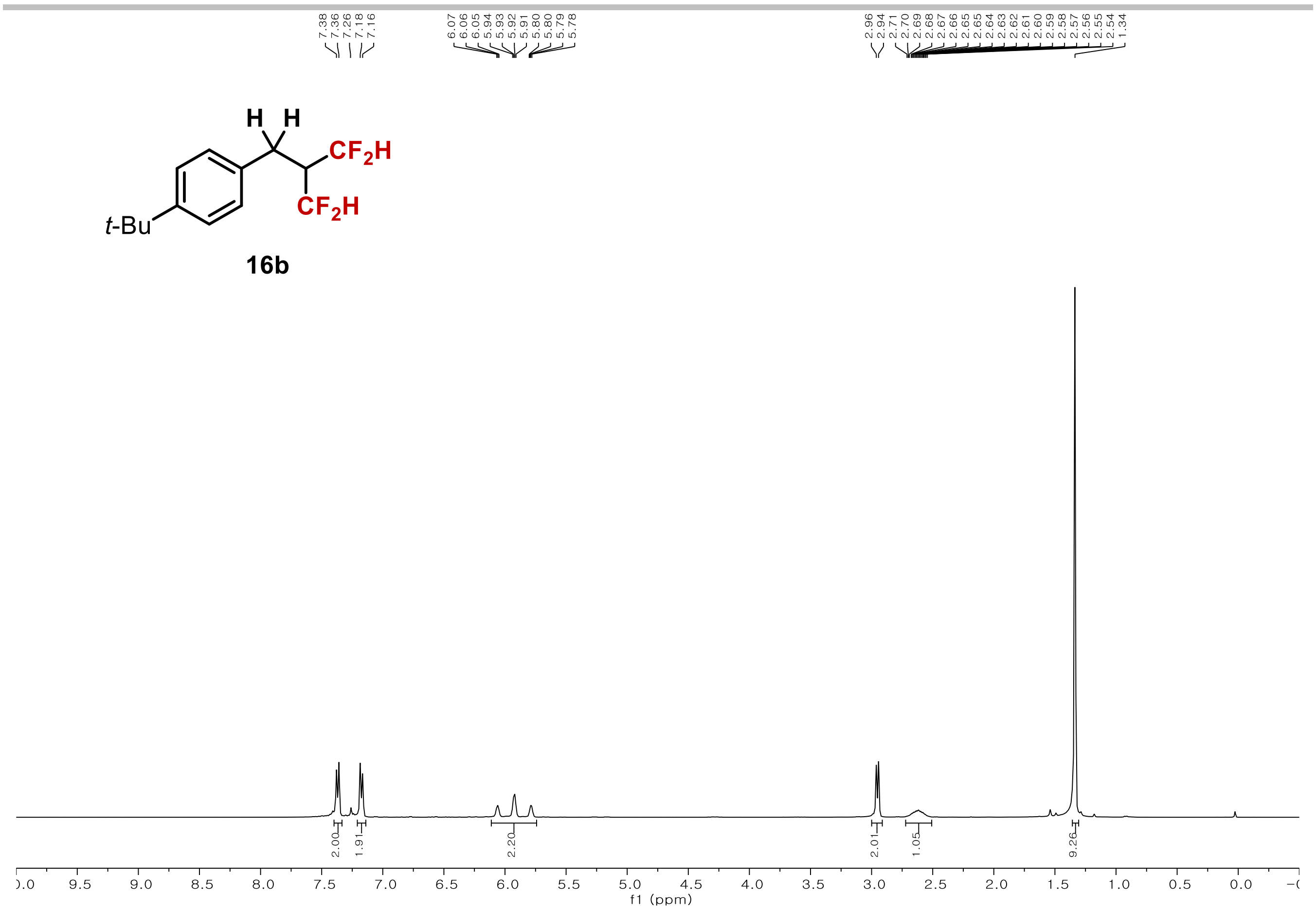

Supplementary Figure 135. ^{1}H NMR Spectrum of 1-(*tert*-Butyl)-4-(2-(difluoromethyl)-3,3-difluoropropyl)benzene (**16b**)

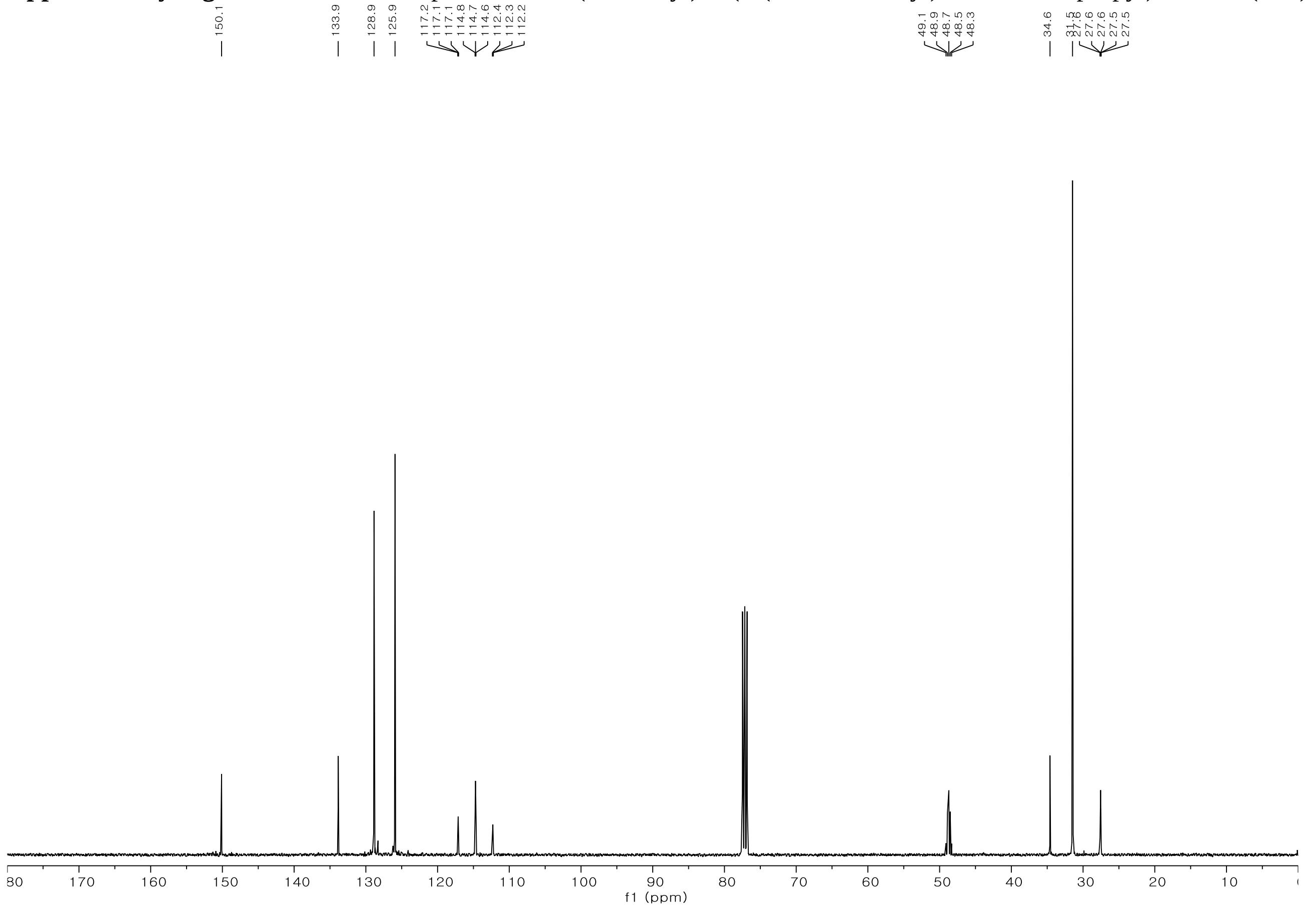

Supplementary Figure 136. ^{13}C NMR Spectrum of 1-(*tert*-Butyl)-4-(2-(difluoromethyl)-3,3-difluoropropyl)benzene (**16b**)

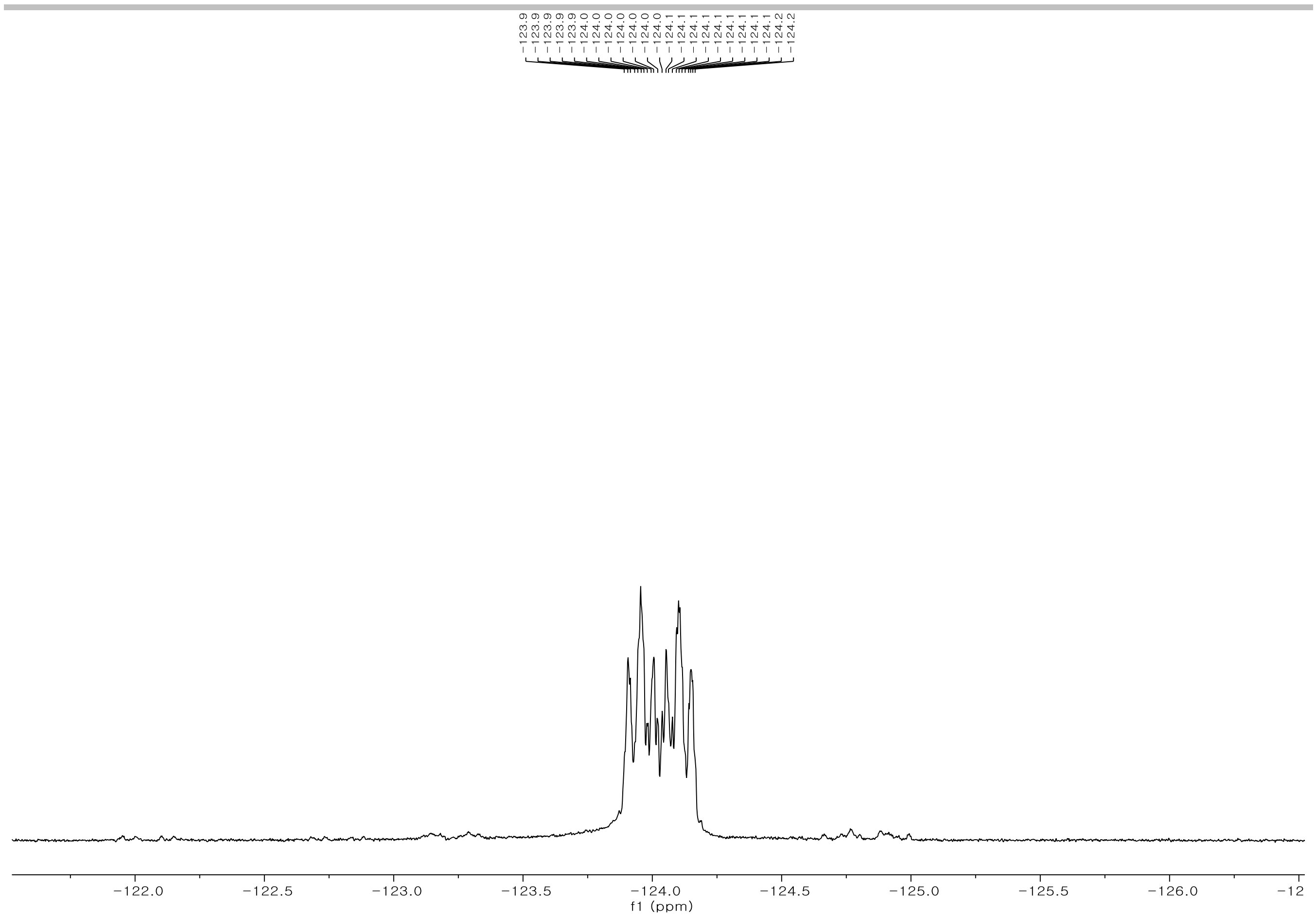

Supplementary Figure 137. ^{19}F NMR Spectrum of 1-(*tert*-Butyl)-4-(2-(difluoromethyl)-3,3-difluoropropyl)benzene (**16b**)

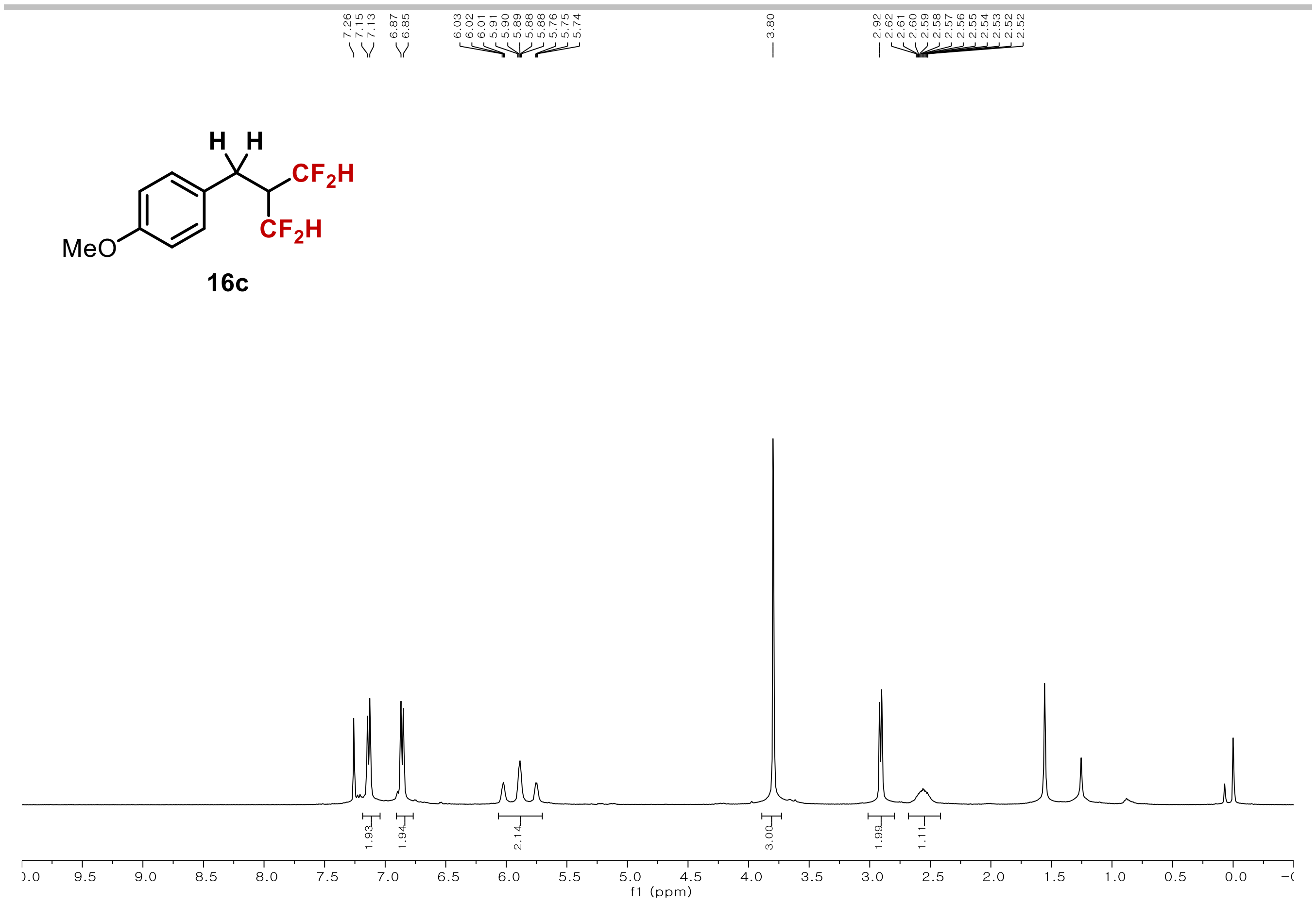

Supplementary Figure 138. ^{1}H NMR Spectrum of 1-(2-(Difluoromethyl)-3,3-difluoropropyl)-4-methoxybenzene (**16c**)

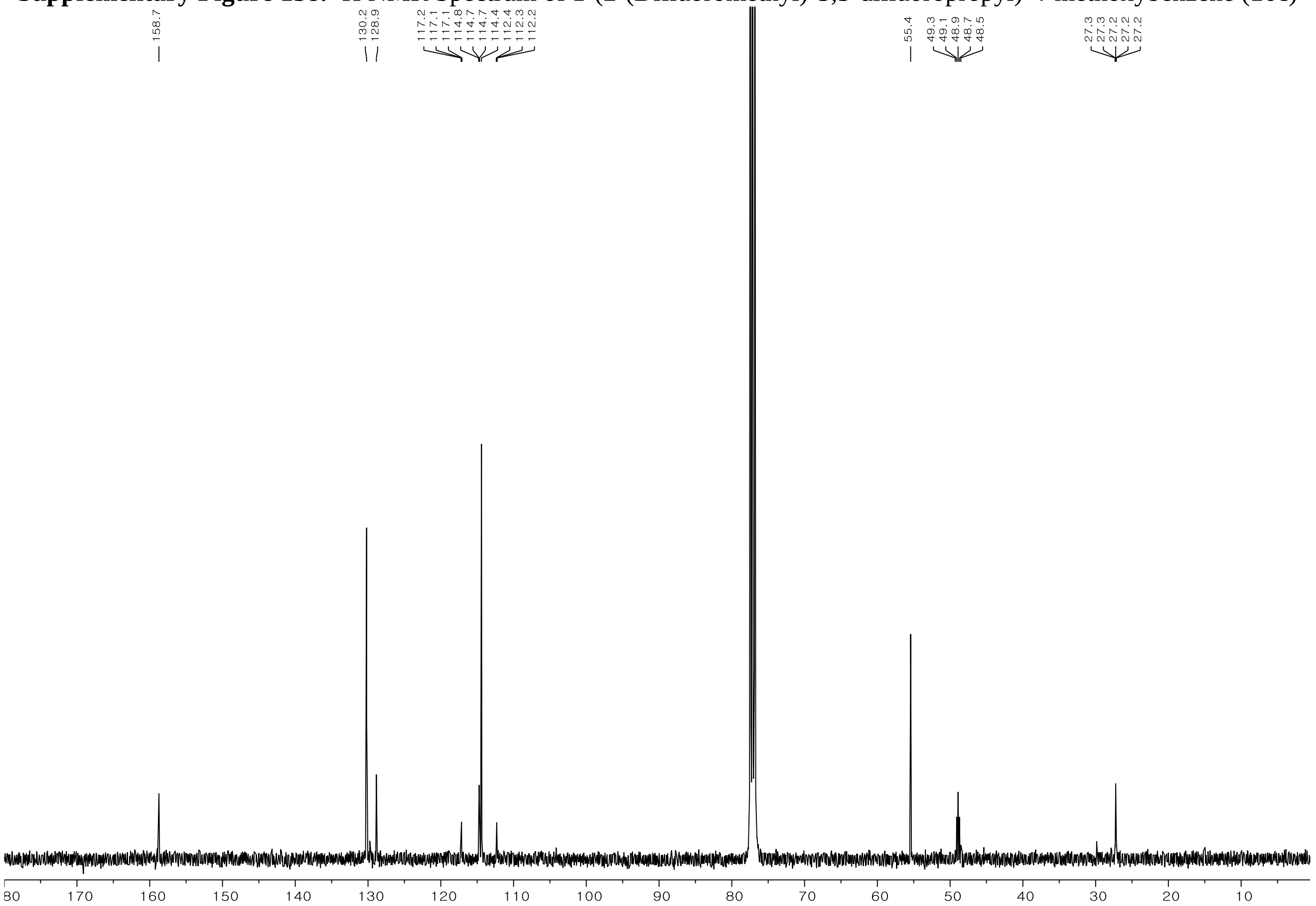

Supplementary Figure 139. ^{13}C NMR Spectrum of 1-(2-(Difluoromethyl)-3,3-difluoropropyl)-4-methoxybenzene (**16c**)

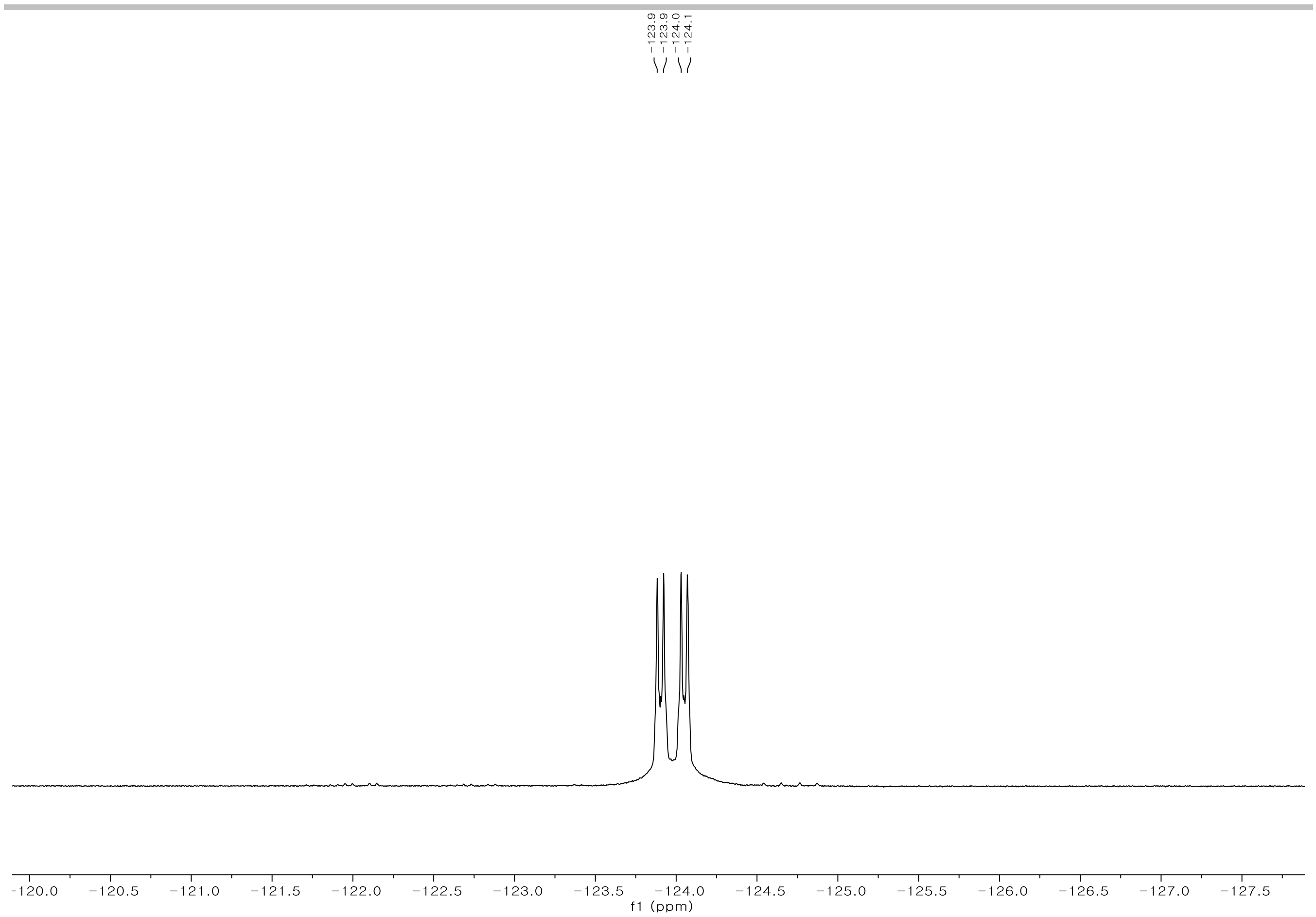

Supplementary Figure 140. ^{19}F NMR Spectrum of 1-(2-(Difluoromethyl)-3,3-difluoropropyl)-4-methoxybenzene (**16c**)

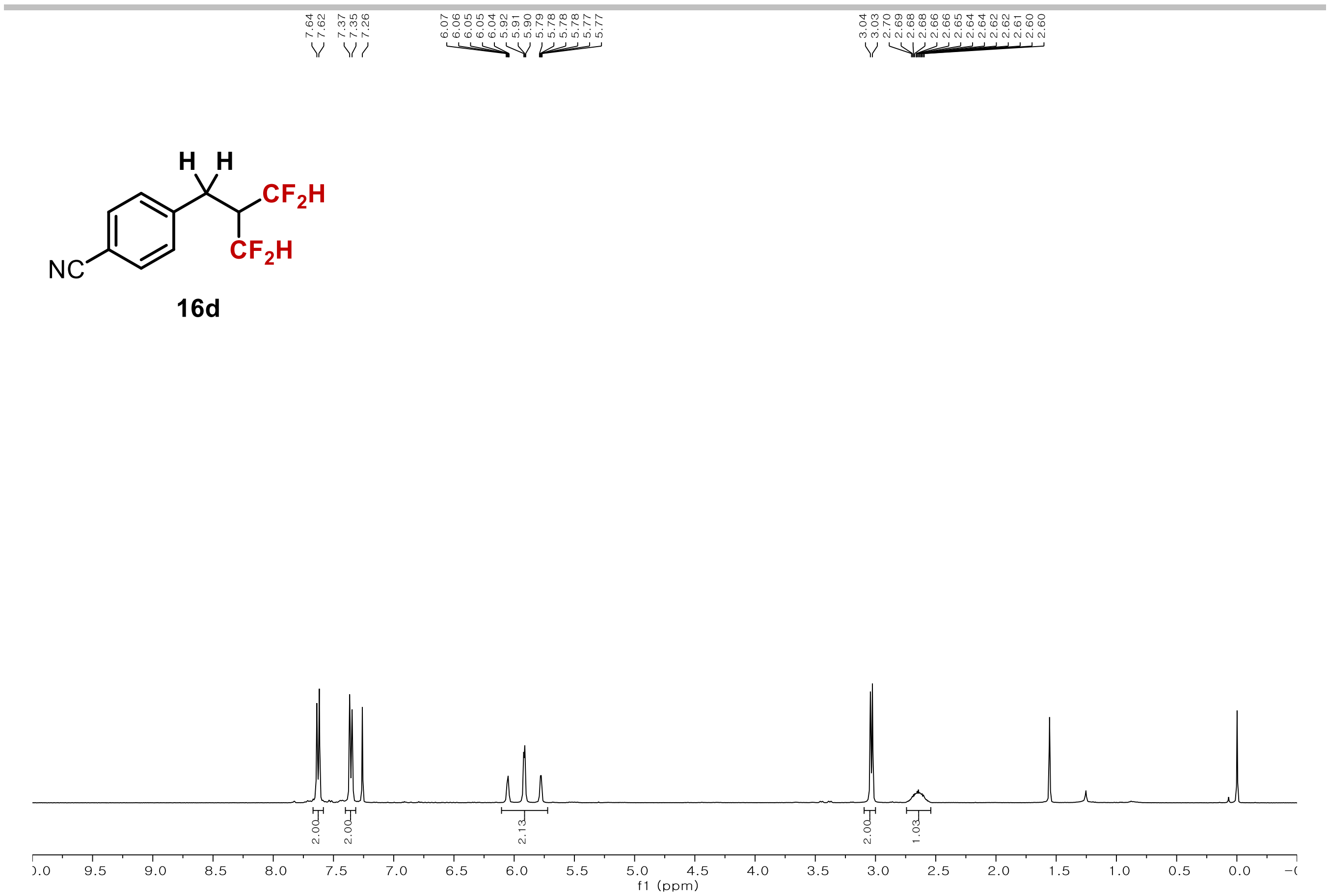

Supplementary Figure 141. ^{1}H NMR Spectrum of 4-(2-(Difluoromethyl)-3,3-difluoropropyl)benzonitrile (**16d**)

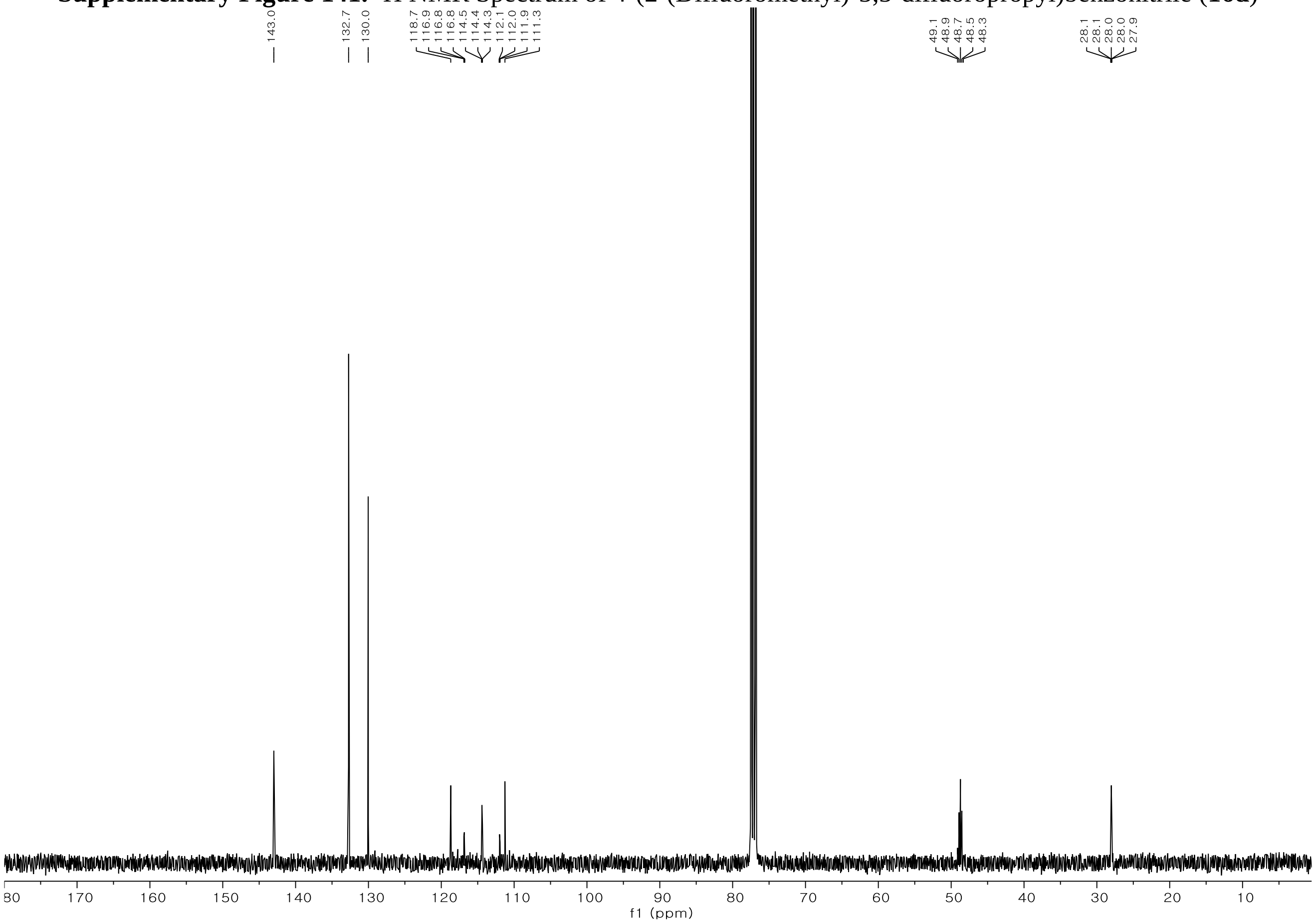

Supplementary Figure 142. ^{13}C NMR Spectrum of 4-(2-(Difluoromethyl)-3,3-difluoropropyl)benzonitrile (**16d**)

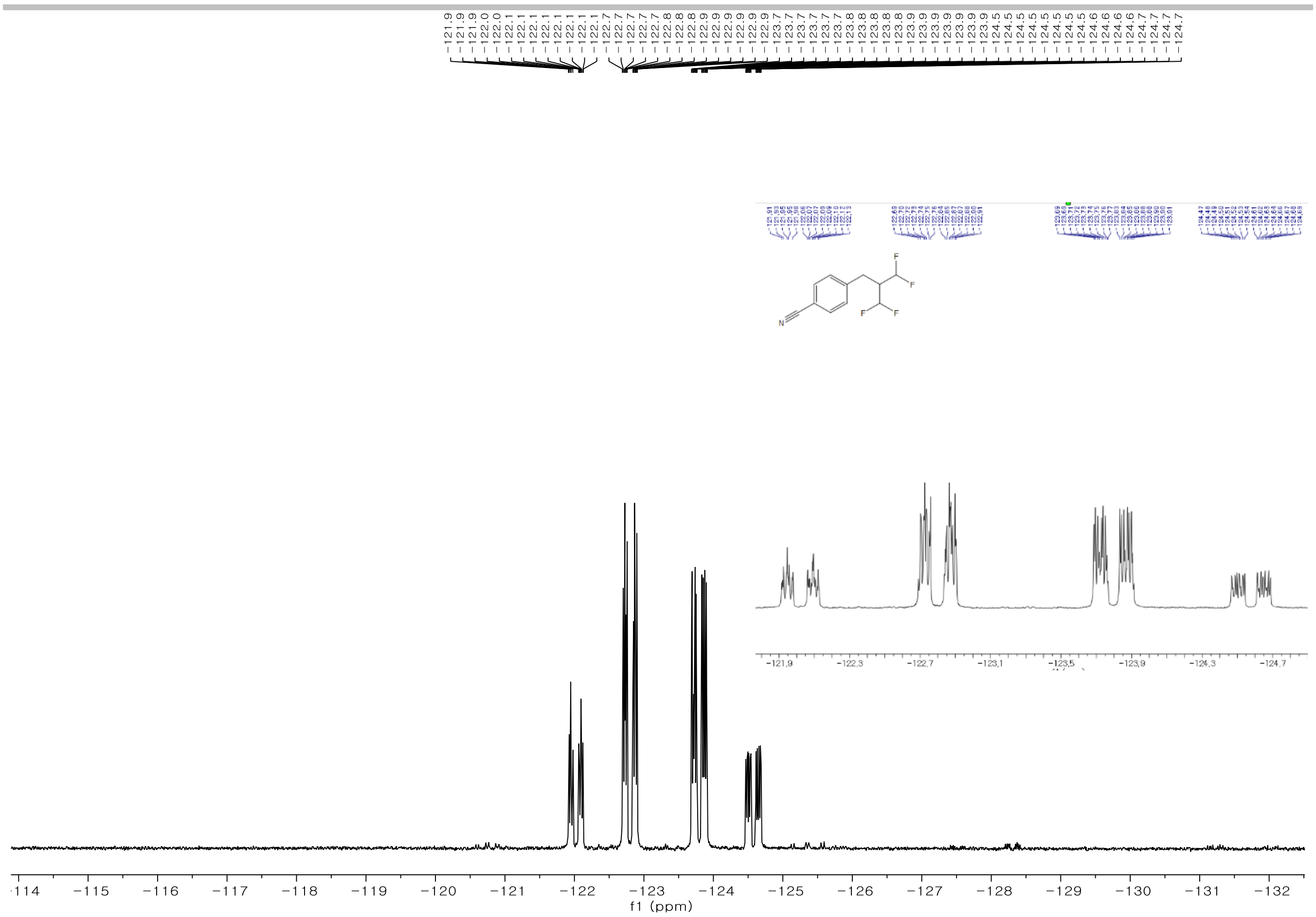

Supplementary Figure 143. ^{19}F NMR Spectrum of 4-(2-(Difluoromethyl)-3,3-difluoropropyl)benzonitrile (**16d**)

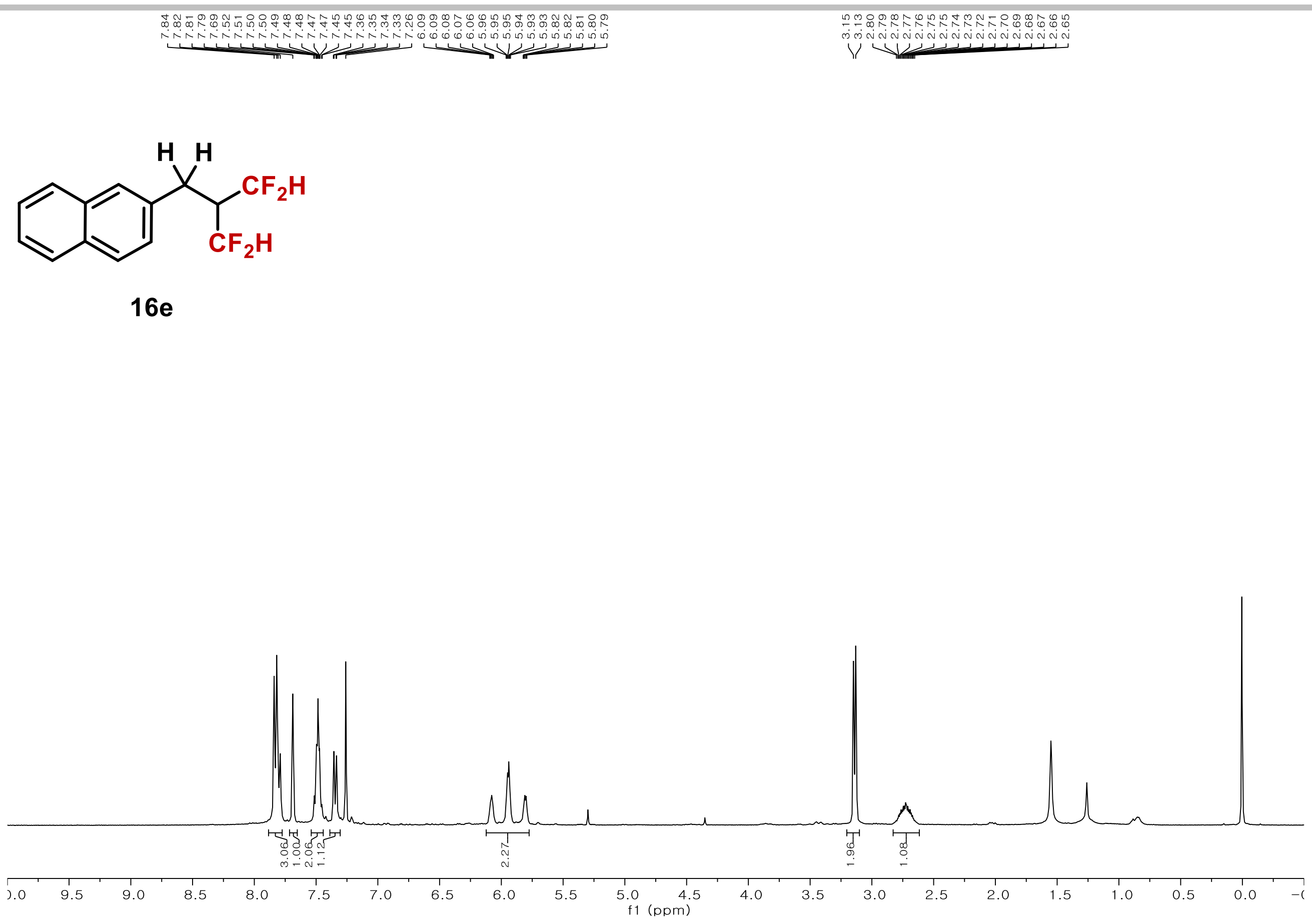

Supplementary Figure 144. ^{1}H NMR Spectrum of 2-(2-(Difluoromethyl)-3,3-difluoropropyl)naphthalene (**16e**)

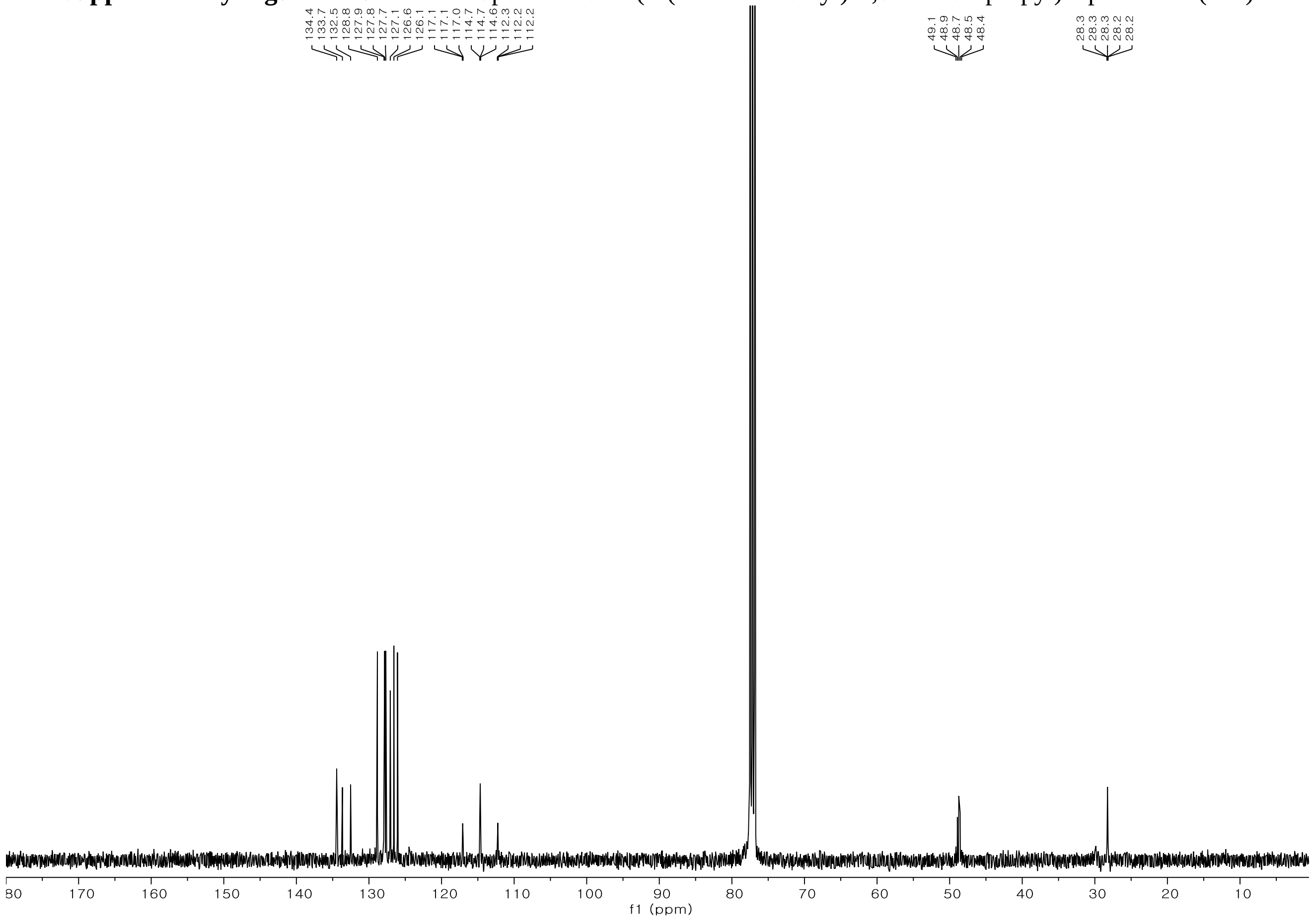

Supplementary Figure 145. ^{13}C NMR Spectrum of 2-(2-(Difluoromethyl)-3,3-difluoropropyl)naphthalene (**16e**)

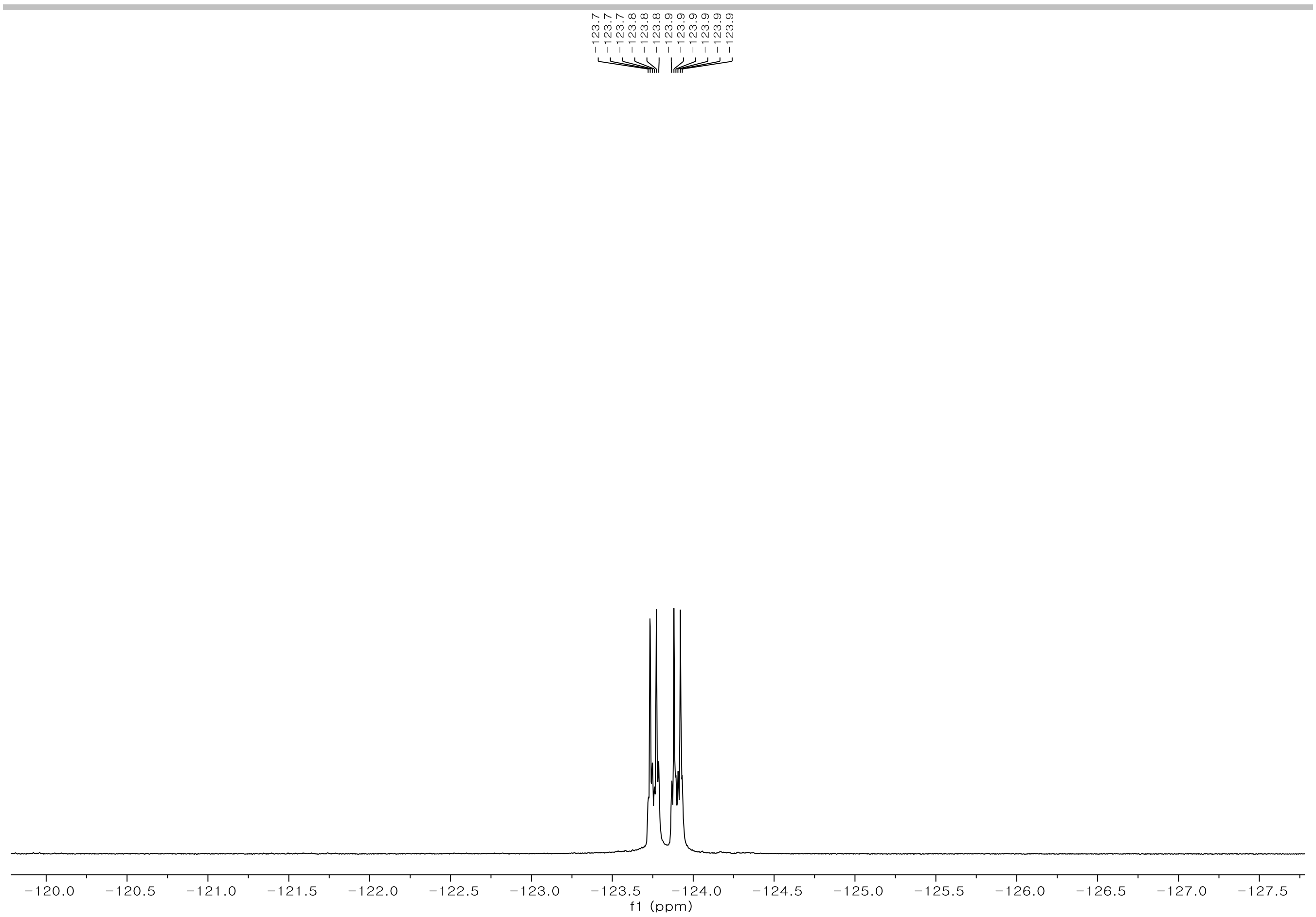

Supplementary Figure 146. ^{19}F NMR Spectrum of 2-(2-(Difluoromethyl)-3,3-difluoropropyl)naphthalene (**16e**)

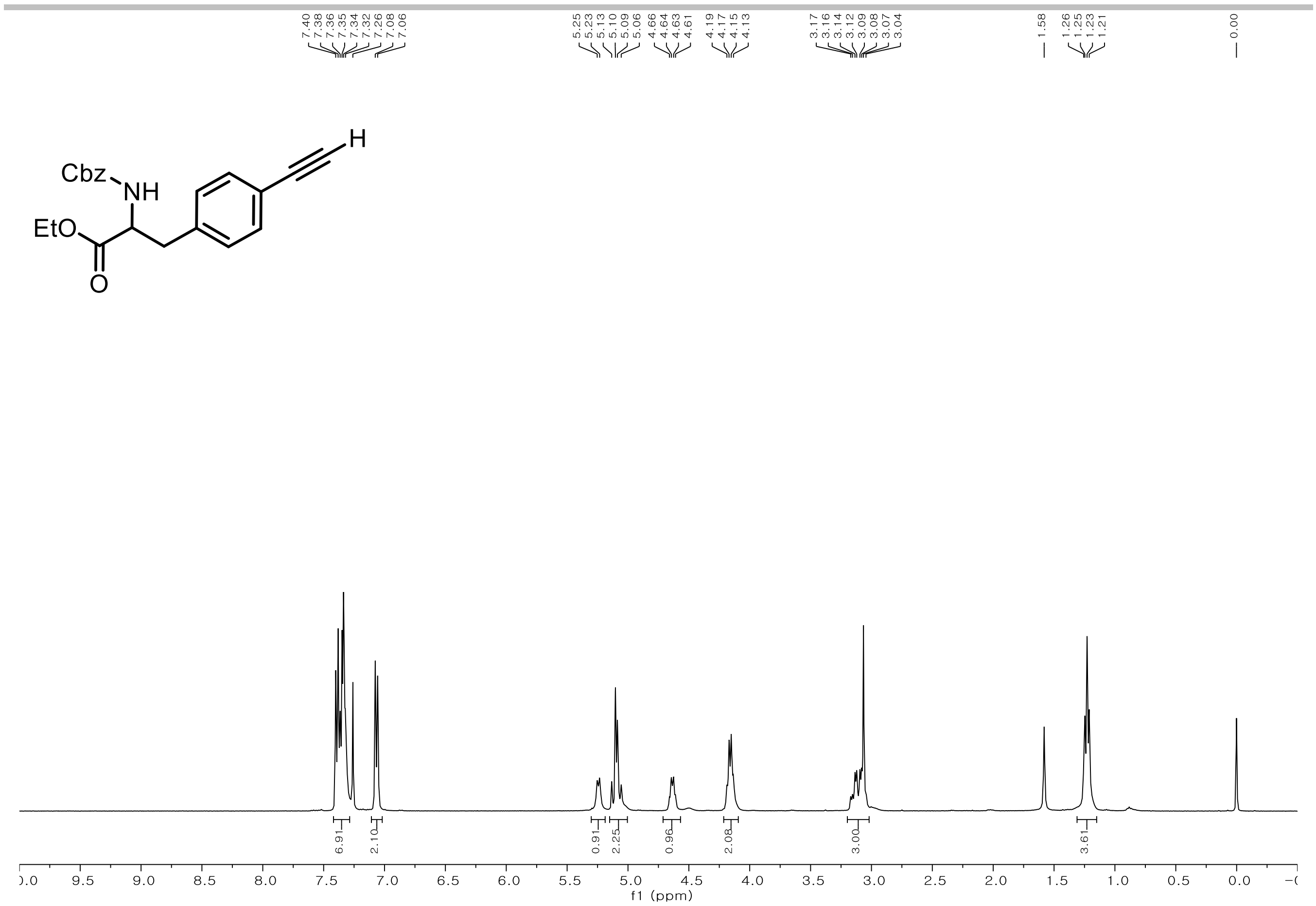

Supplementary Figure 147. ^{1}H NMR Spectrum of Ethyl 2-(((benzyloxy)carbonyl)amino)-3-(4-ethynylphenyl)propanoate

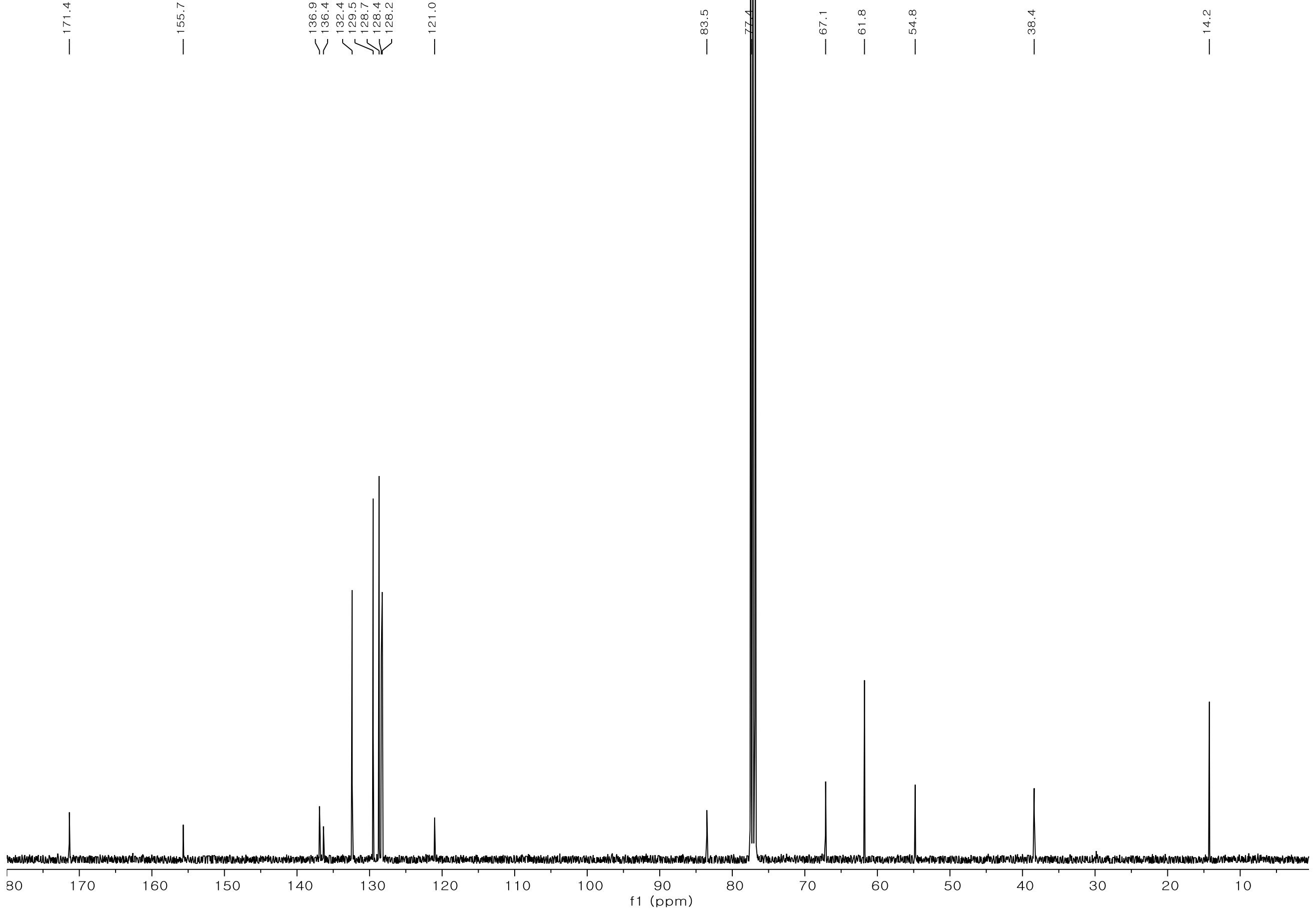

Supplementary Figure 148. ^{13}C NMR Spectrum of Ethyl 2-(((benzyloxy)carbonyl)amino)-3-(4-ethynylphenyl)propanoate

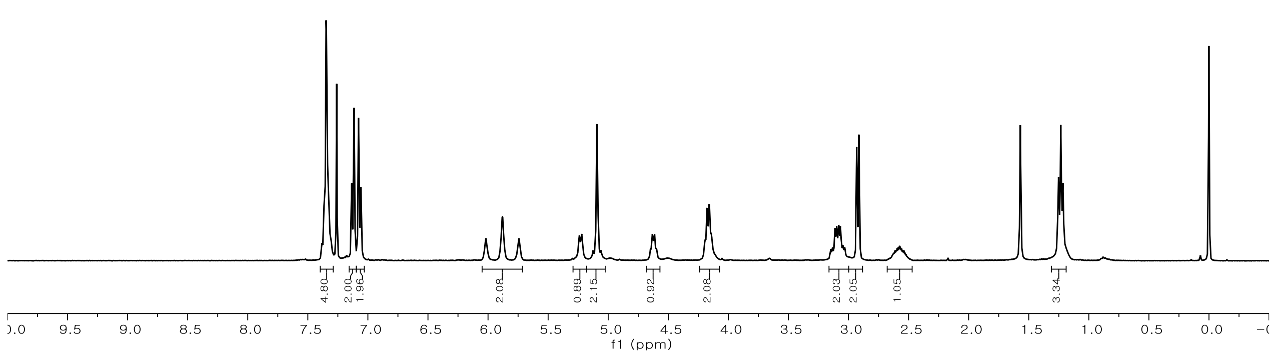

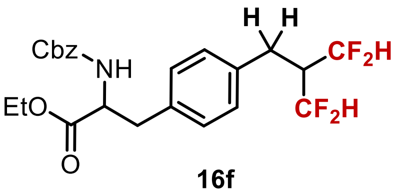

16f

Supplementary Figure 149. ^{1}H NMR Spectrum of Ethyl 2-(((Benzyloxy)carbonyl)amino)-3-(4-(2-(difluoromethyl)-3,3-difluoropropyl)phenyl)propanoate (**16f**)

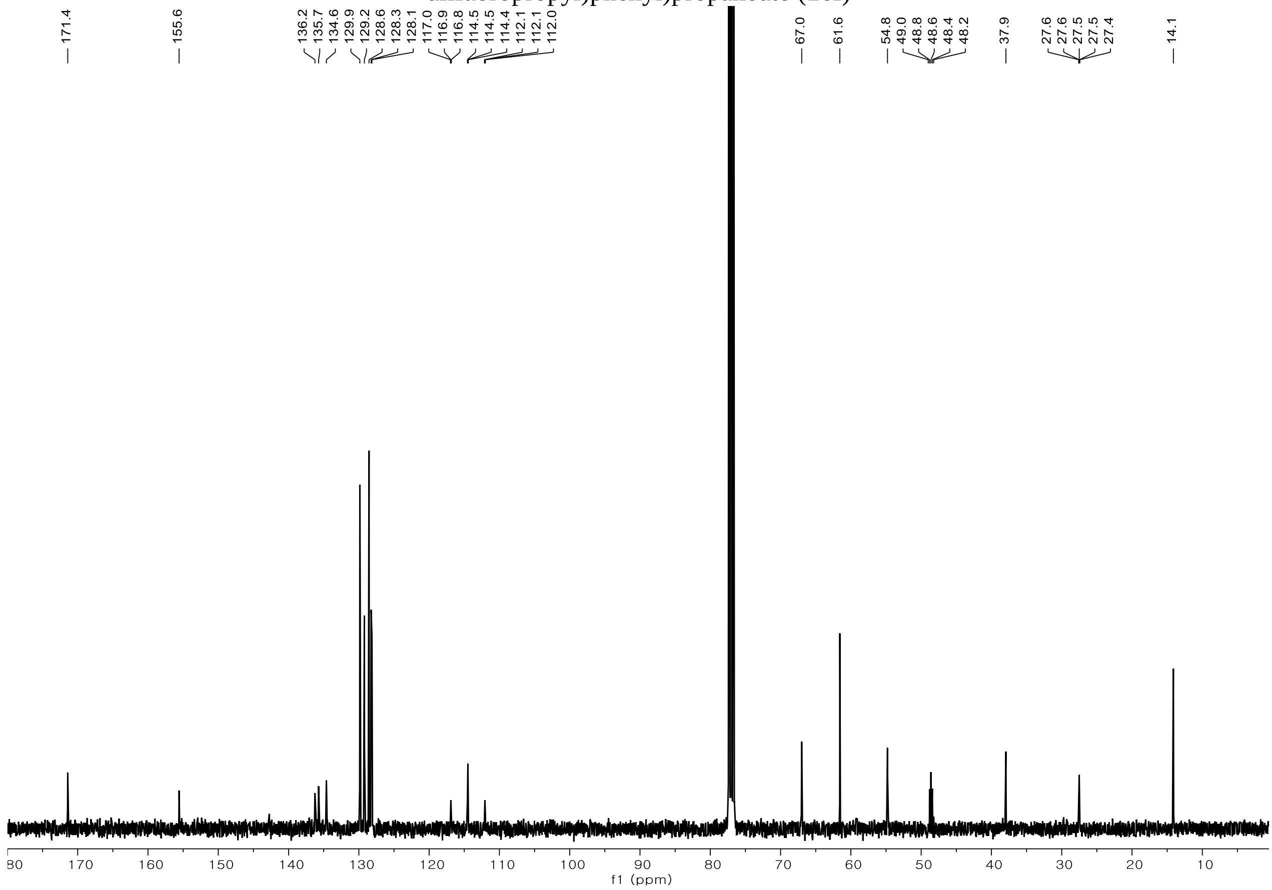

Supplementary Figure 150. ^{13}C NMR Spectrum of Ethyl 2-(((Benzyloxy)carbonyl)amino)-3-(4-(2-(difluoromethyl)-3,3-difluoropropyl)phenyl)propanoate (**16f**)

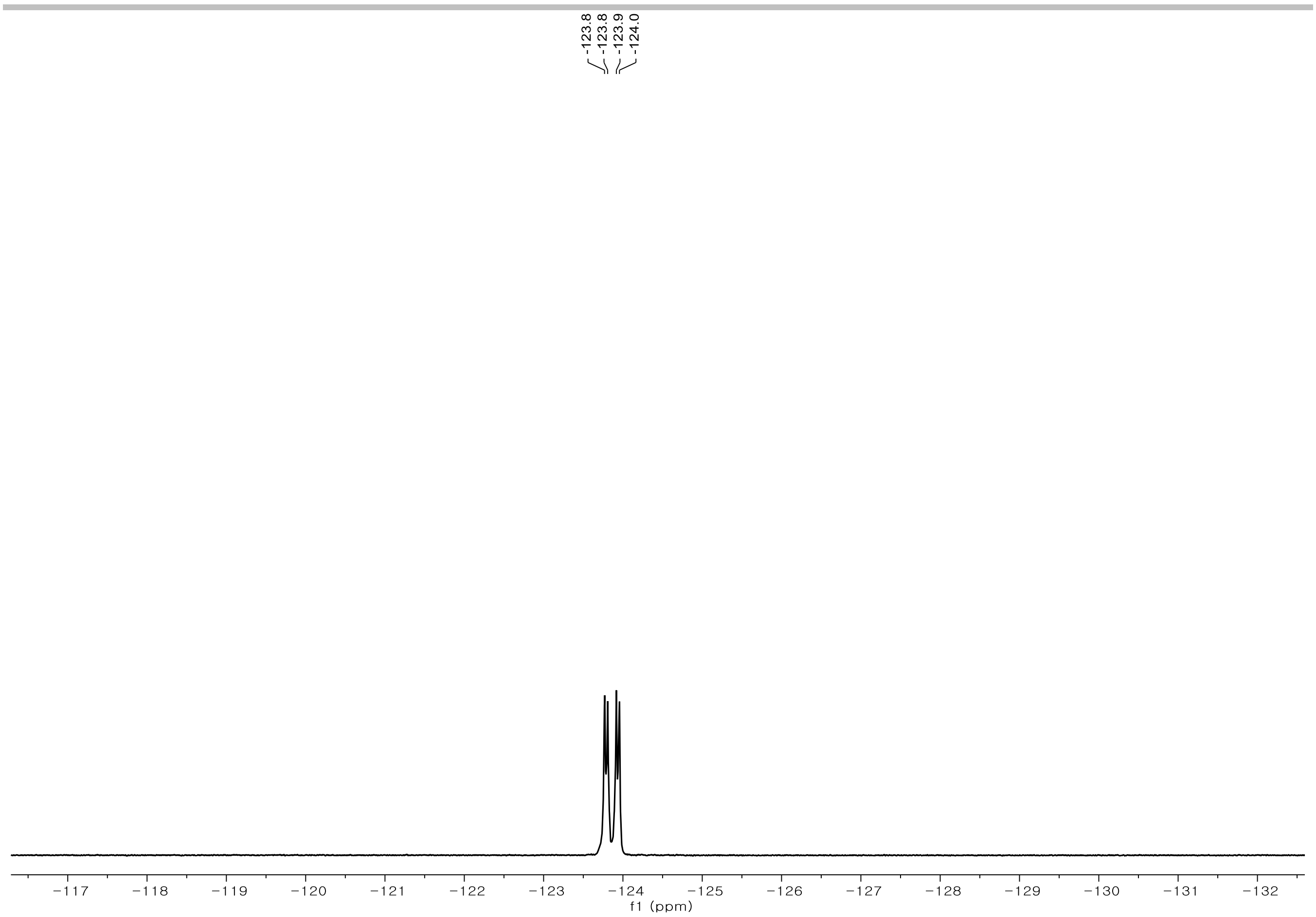

Supplementary Figure 151. ^{19}F NMR Spectrum of Ethyl 2-(((Benzyloxy)carbonyl)amino)-3-(4-(2-(difluoromethyl)-3,3-difluoropropyl)phenyl)propanoate (**16f**)

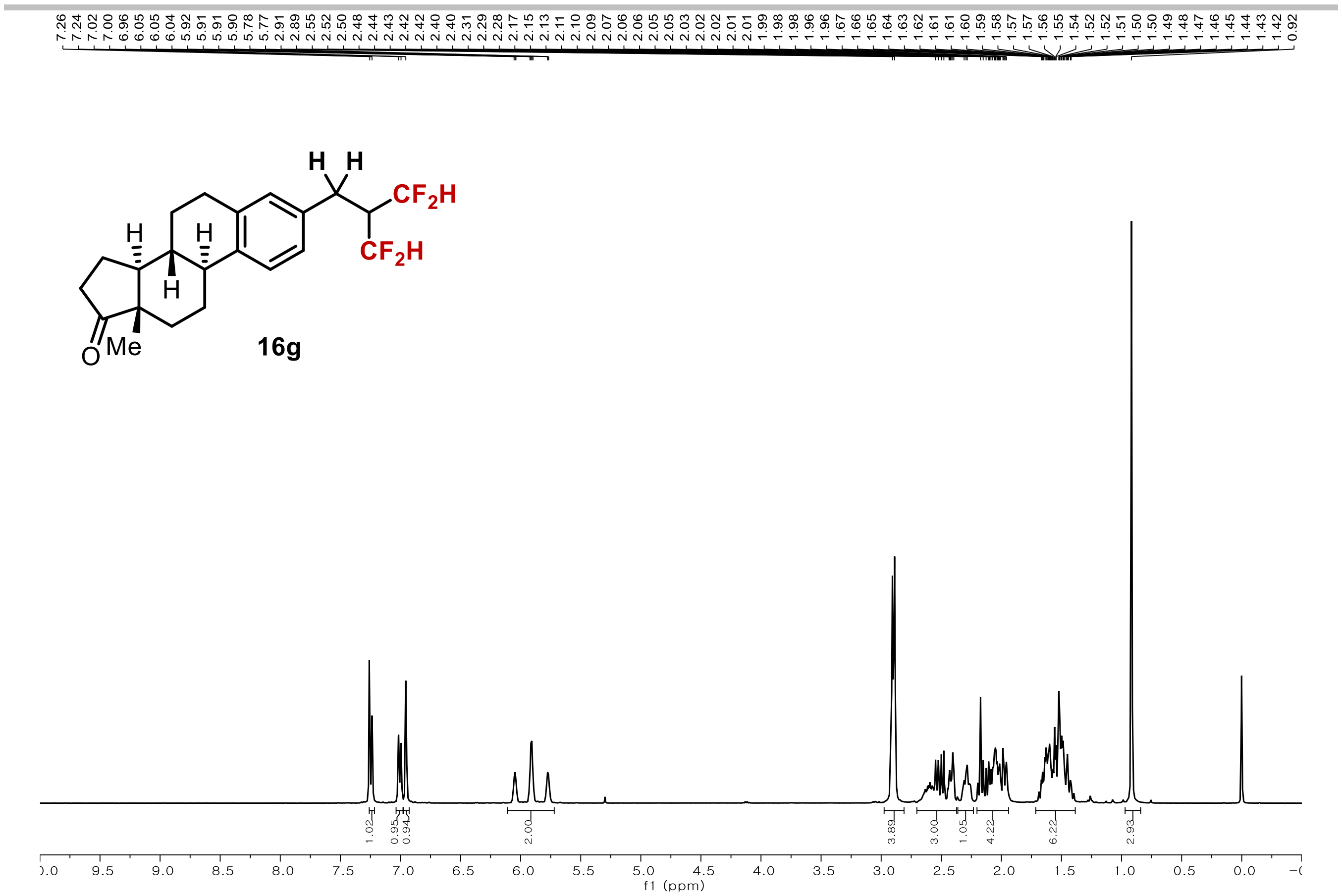

Supplementary Figure 152. ^{1}H NMR Spectrum of (*8R,9S,13S,14S*)-3-(2-(Difluoromethyl)-3,3-difluoropropyl)-13-methyl-6,7,8,9,11,12,13,14,15,16-decahydro-17H-cyclopenta[a]phenanthren-17-one (**16g**)

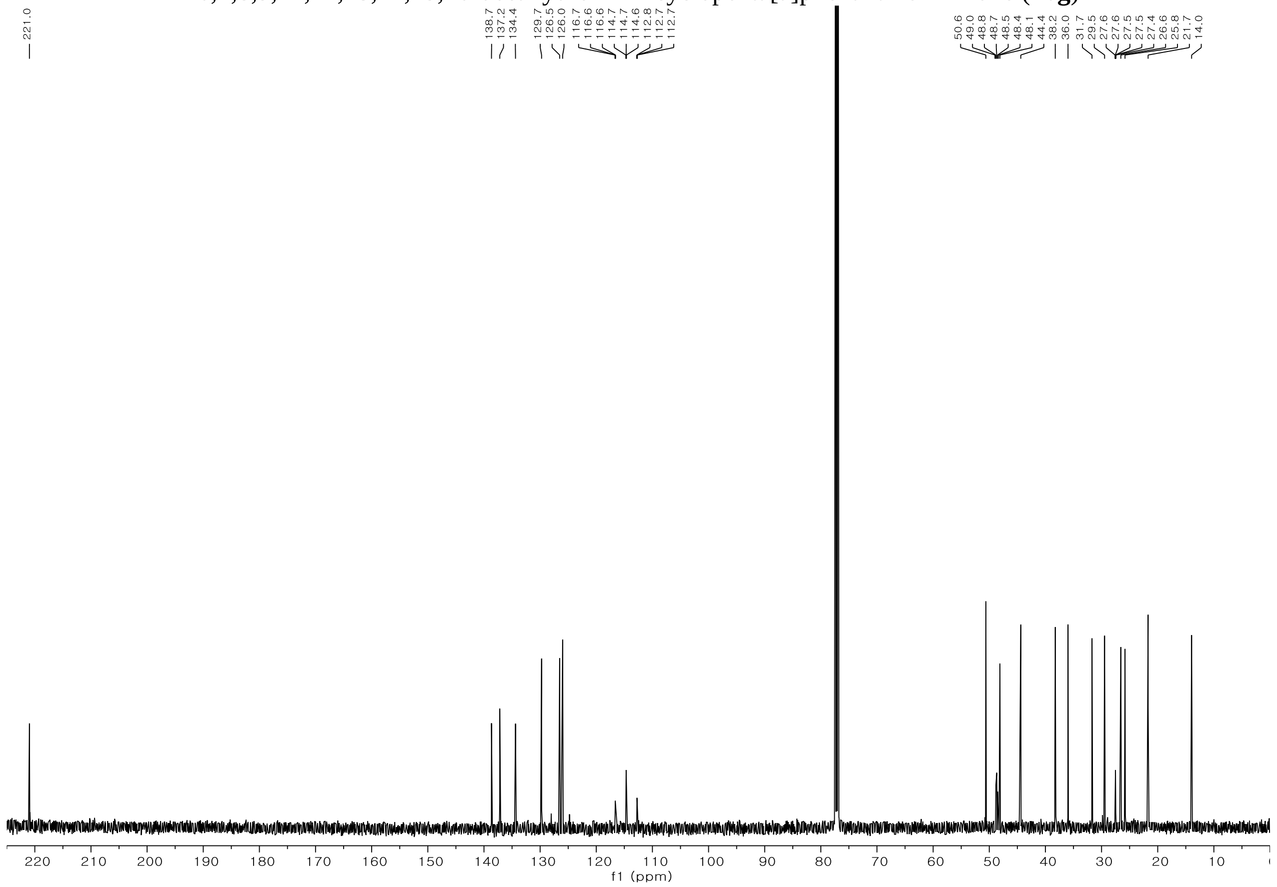

Supplementary Figure 153. ^{13}C NMR Spectrum of (*8R,9S,13S,14S*)-3-(2-(Difluoromethyl)-3,3-difluoropropyl)-13-methyl-6,7,8,9,11,12,13,14,15,16-decahydro-17H-cyclopenta[a]phenanthren-17-one (**16g**)

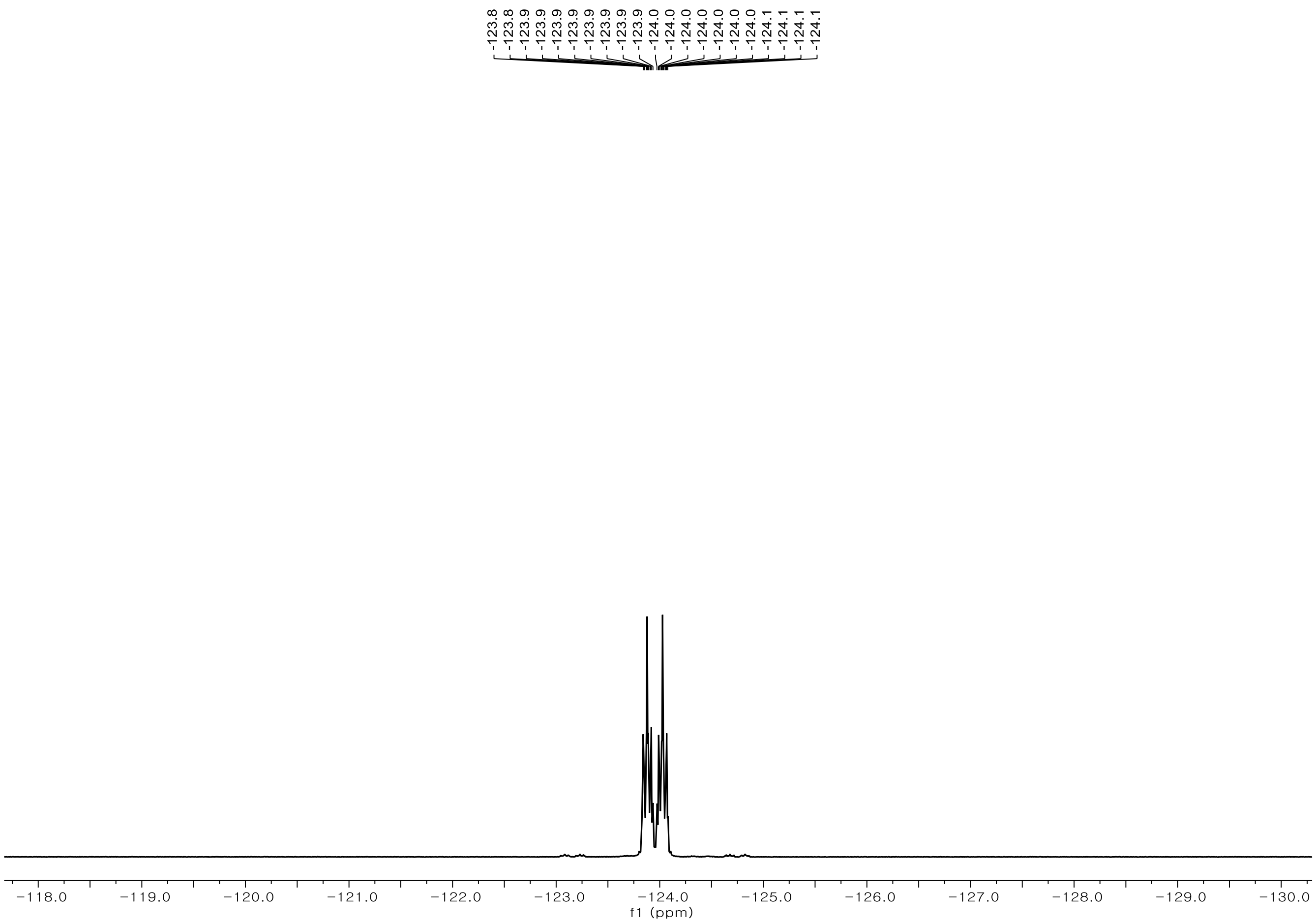

Supplementary Figure 154. ^{19}F NMR Spectrum of (*8R,9S,13S,14S*)-3-(2-(Difluoromethyl)-3,3-difluoropropyl)-13-methyl-6,7,8,9,11,12,13,14,15,16-decahydro-17H-cyclopenta[a]phenanthren-17-one (**16g**)

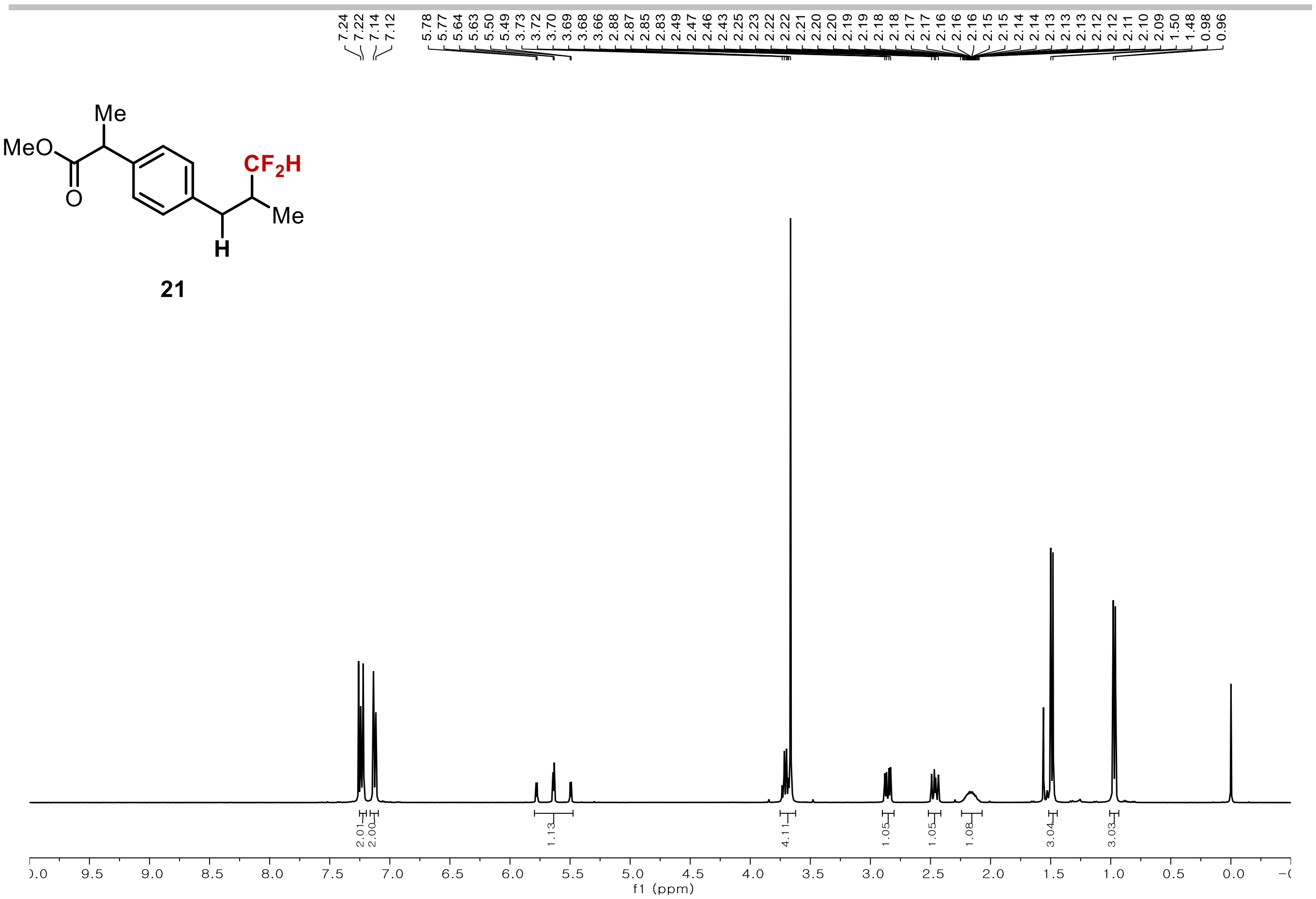

Supplementary Figure 155. ^{1}H NMR Spectrum of Methyl 2-(4-(-3,3-difluoro-2-methylpropyl)phenyl)propanoate (**21**)

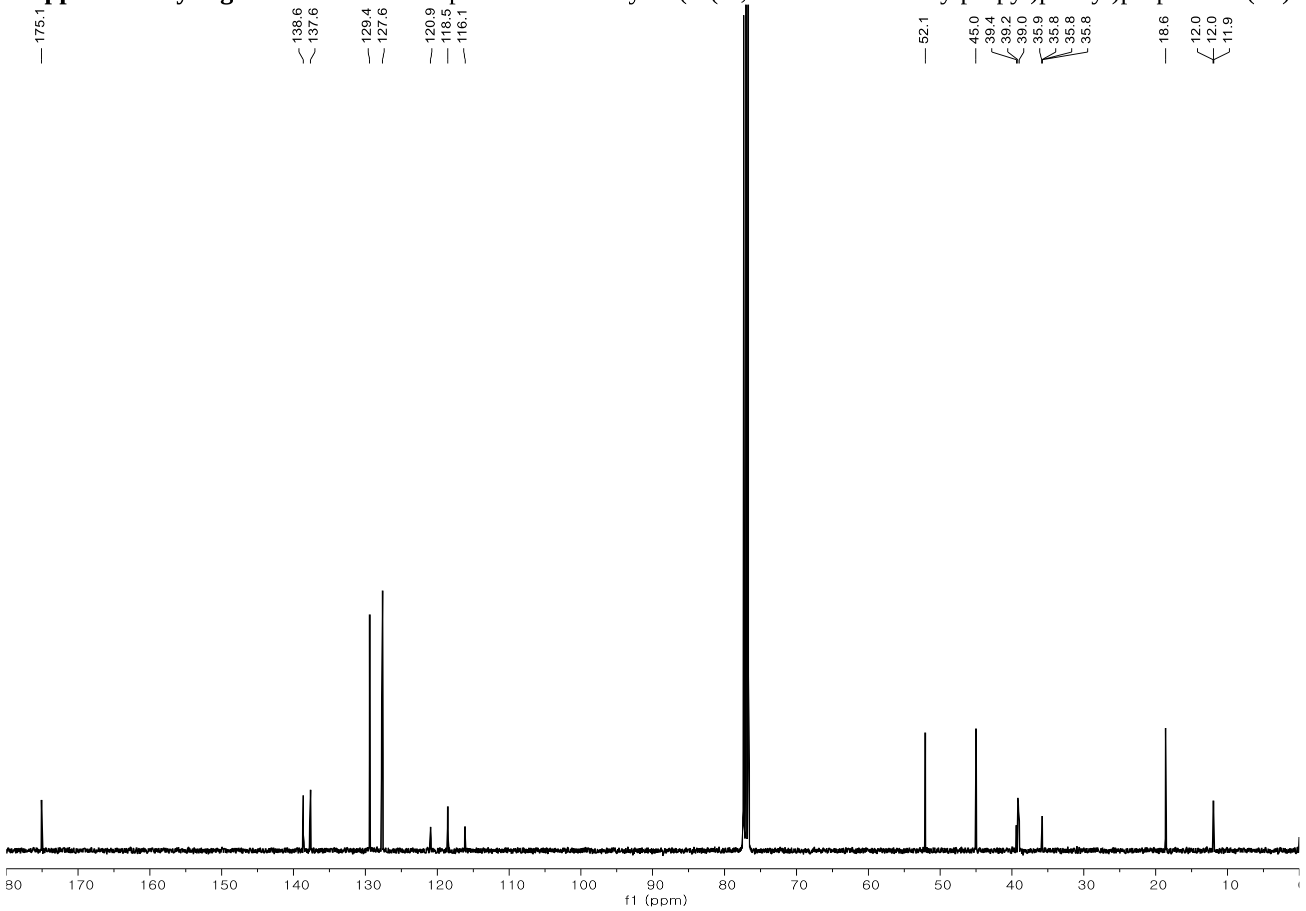

Supplementary Figure 156. ^{13}C NMR Spectrum of Methyl 2-(4-(-3,3-difluoro-2-methylpropyl)phenyl)propanoate (**21**)

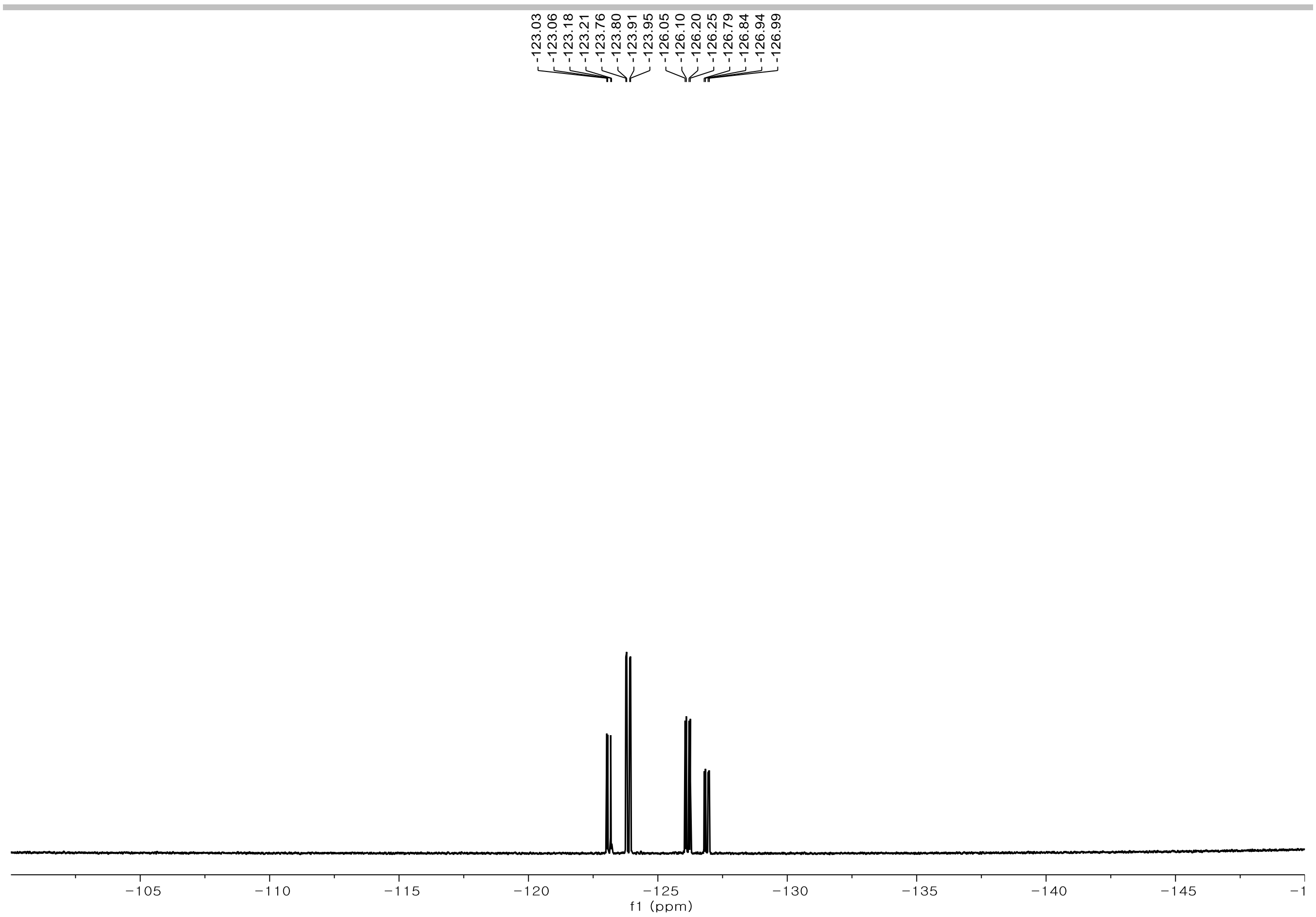

Supplementary Figure 157. ^{19}F NMR Spectrum of Methyl 2-(4-(-3,3-difluoro-2-methylpropyl)phenyl)propanoate (**21**)

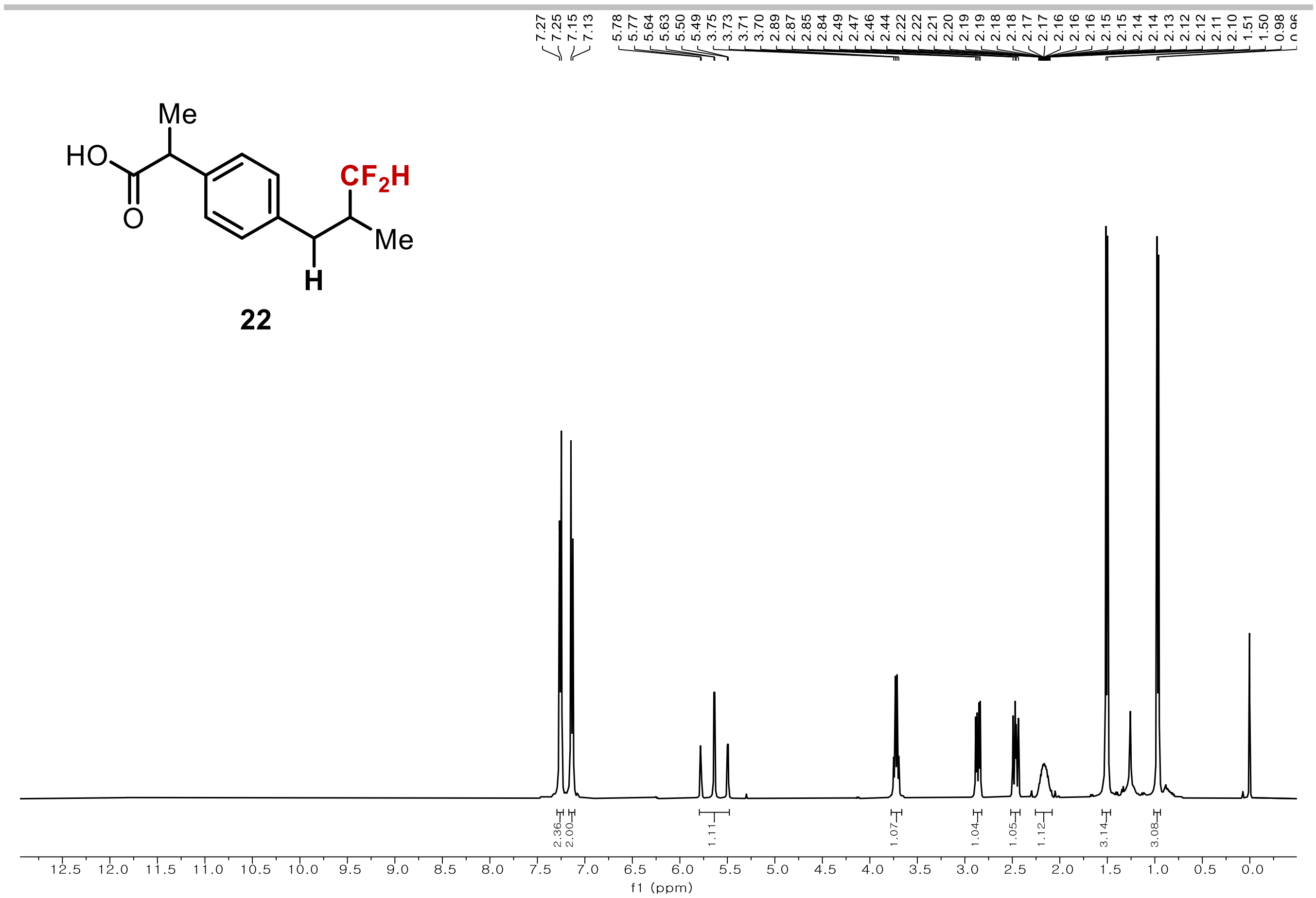

Supplementary Figure 158. ^{1}H NMR Spectrum of 2-(4-(3,3-Difluoro-2-methylpropyl)phenyl)propanoic acid (**22**)

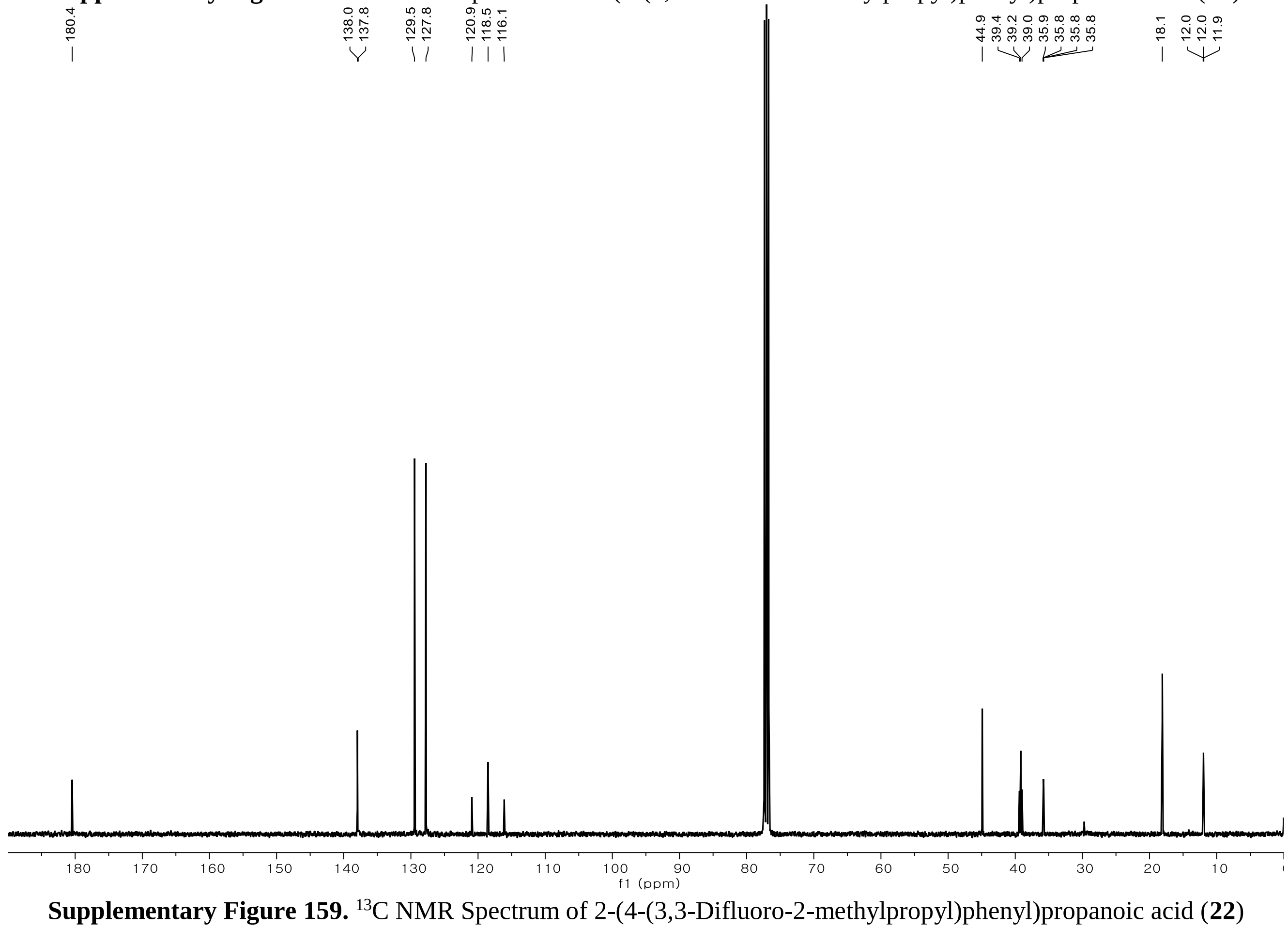

Supplementary Figure 159. ^{13}C NMR Spectrum of 2-(4-(3,3-Difluoro-2-methylpropyl)phenyl)propanoic acid (**22**)

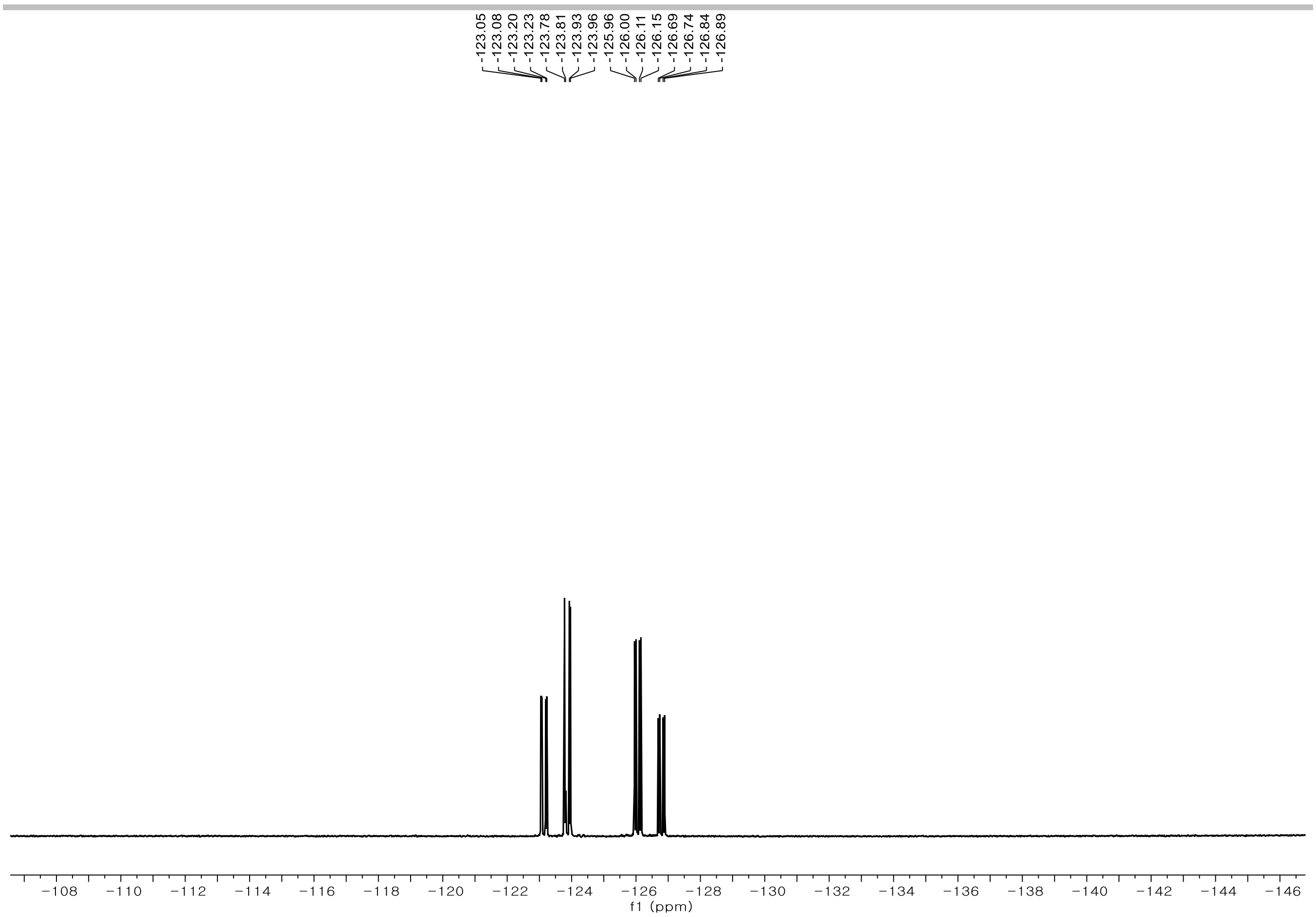

Supplementary Figure 160. ^{19}F NMR Spectrum of 2-(4-(3,3-Difluoro-2-methylpropyl)phenyl)propanoic acid (**22**)

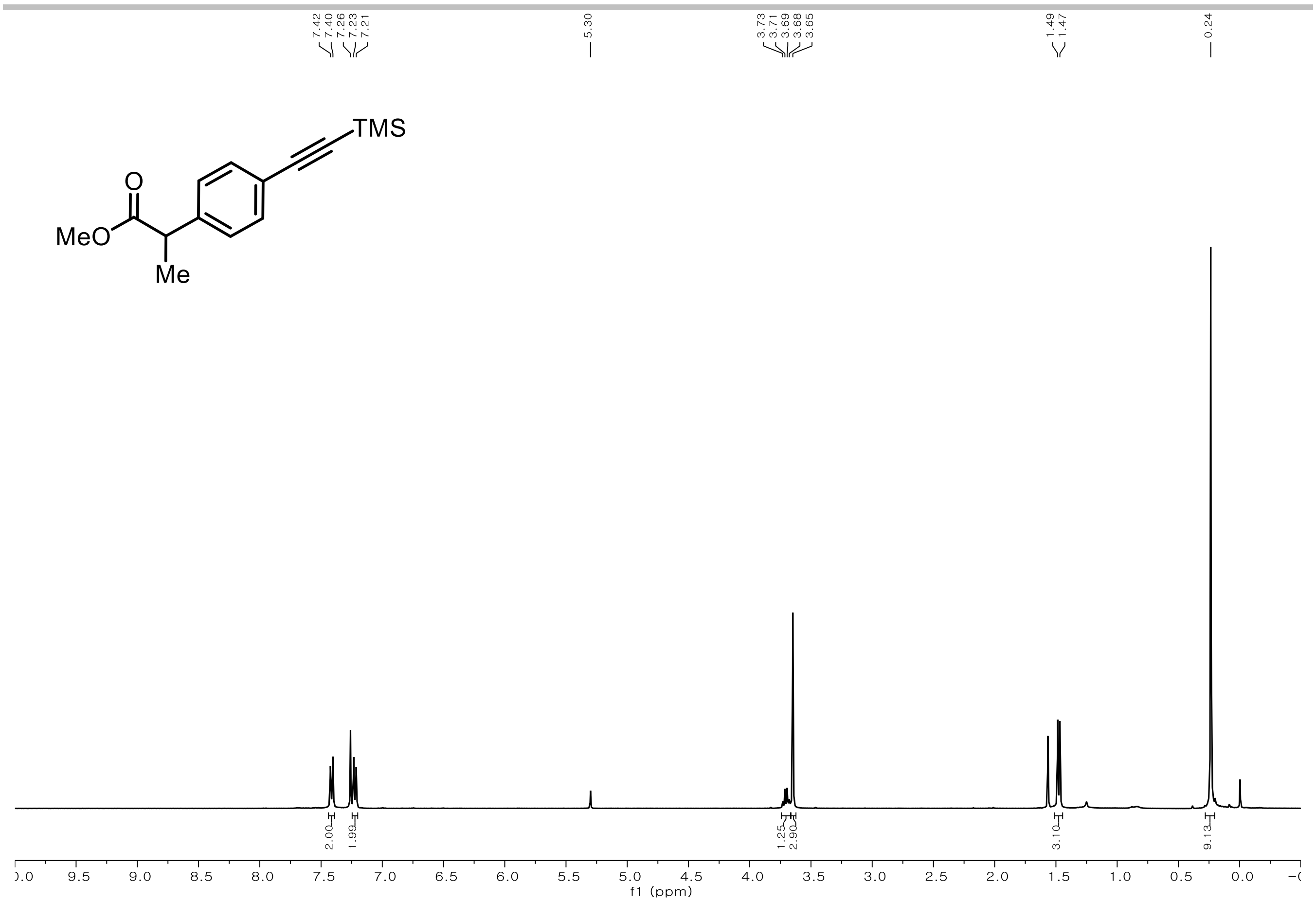

Supplementary Figure 161. ^{1}H NMR Spectrum of Methyl 2-(4-((trimethylsilyl)ethynyl)phenyl)propanoate

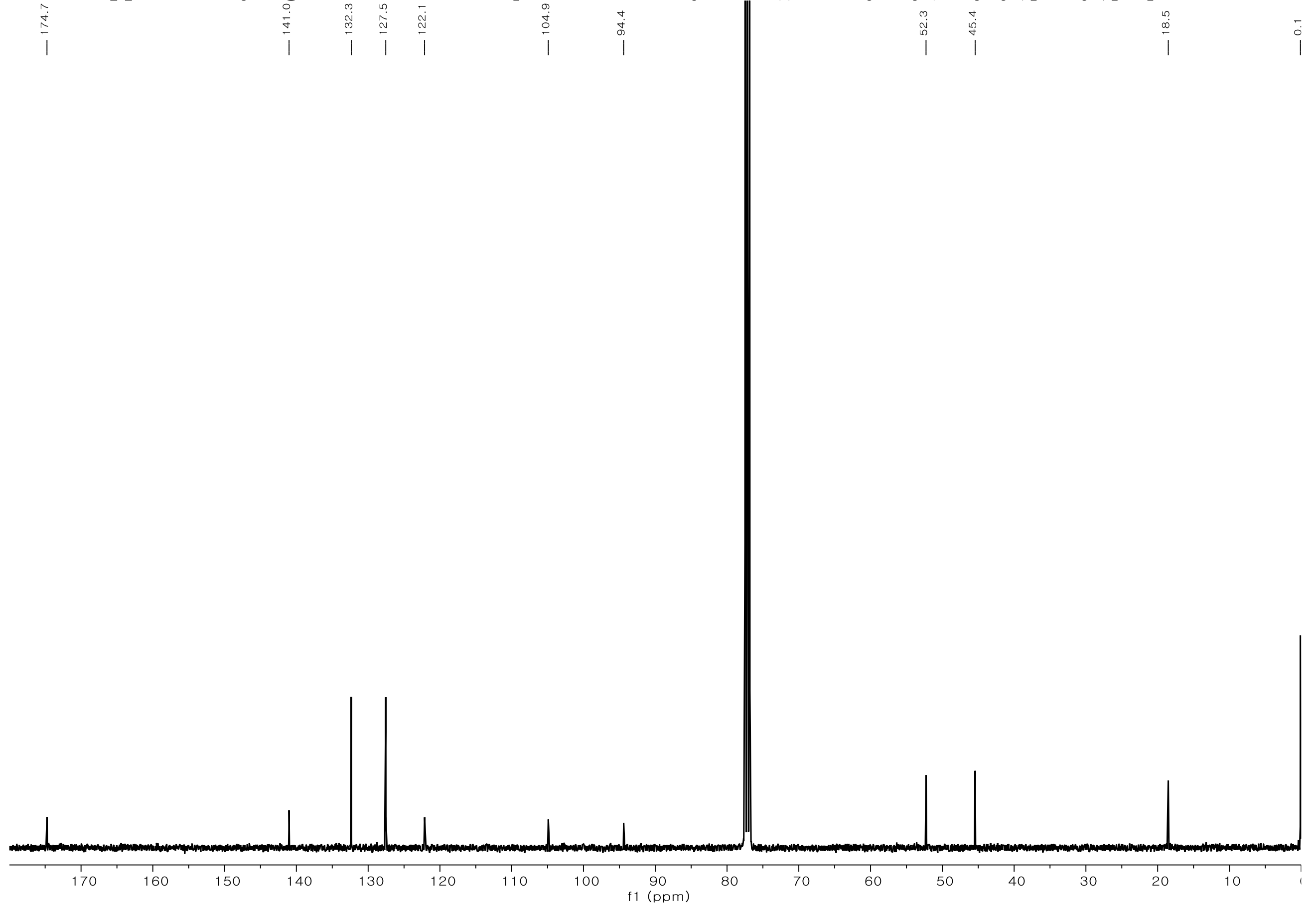

Supplementary Figure 162. ^{13}C NMR Spectrum of Methyl 2-(4-((trimethylsilyl)ethynyl)phenyl)propanoate

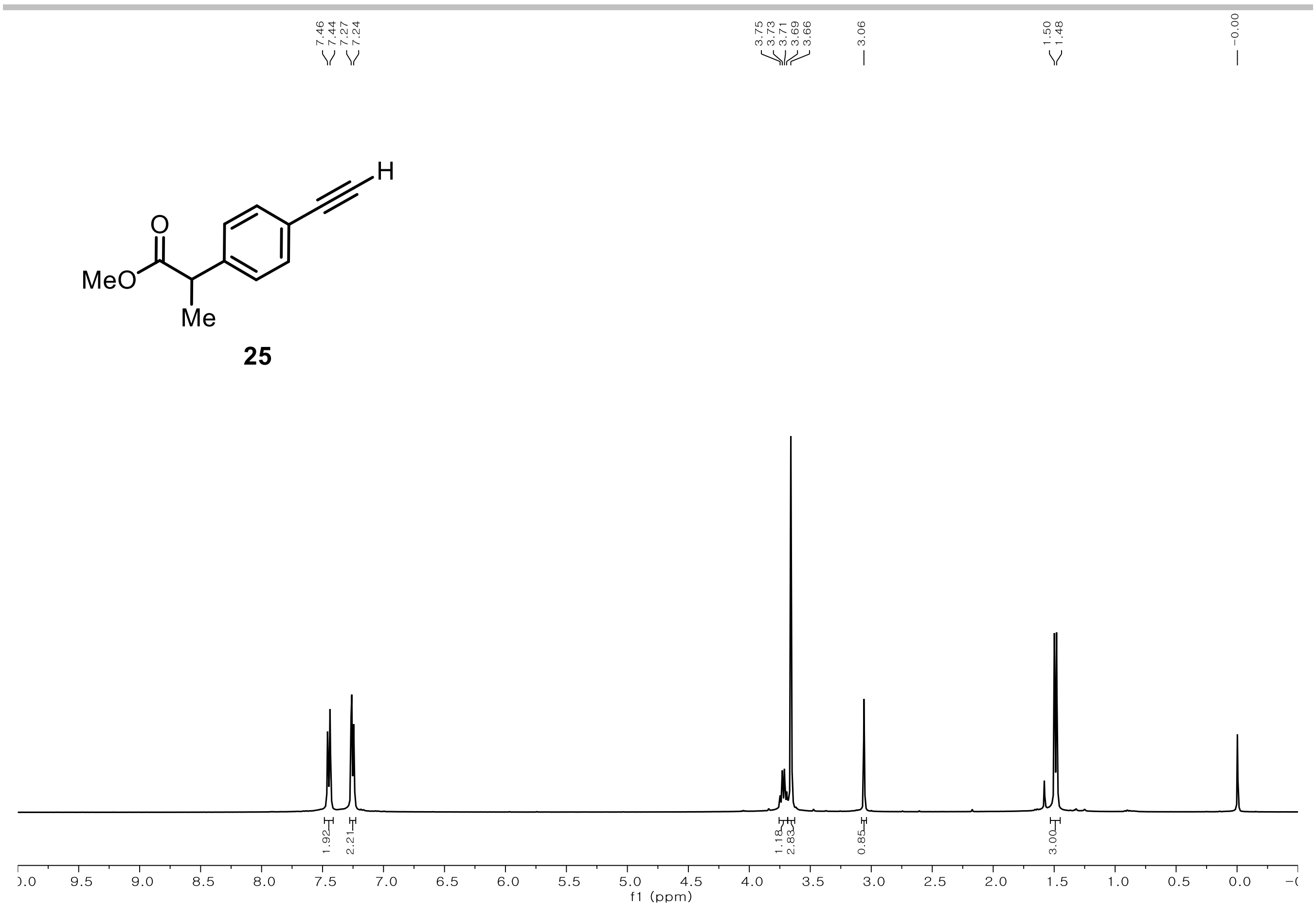

Supplementary Figure 163. ^{1}H NMR Spectrum of Methyl 2-(4-ethynylphenyl)propanoate (**25**)

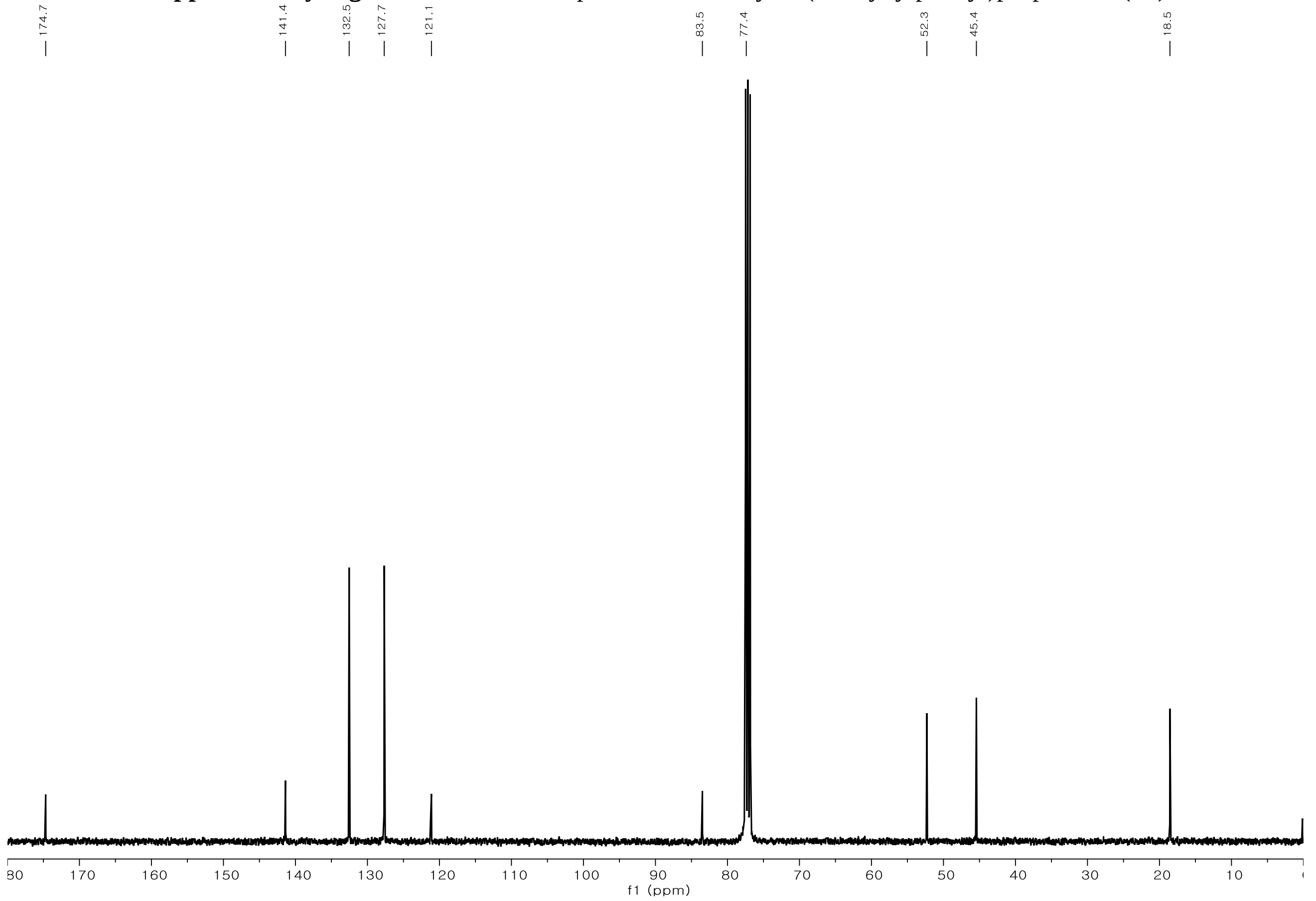

Supplementary Figure 164. ^{13}C NMR Spectrum of Methyl 2-(4-ethynylphenyl)propanoate (**25**)

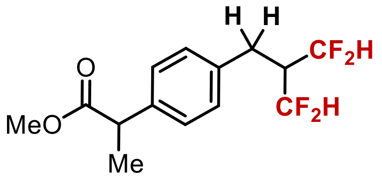

26

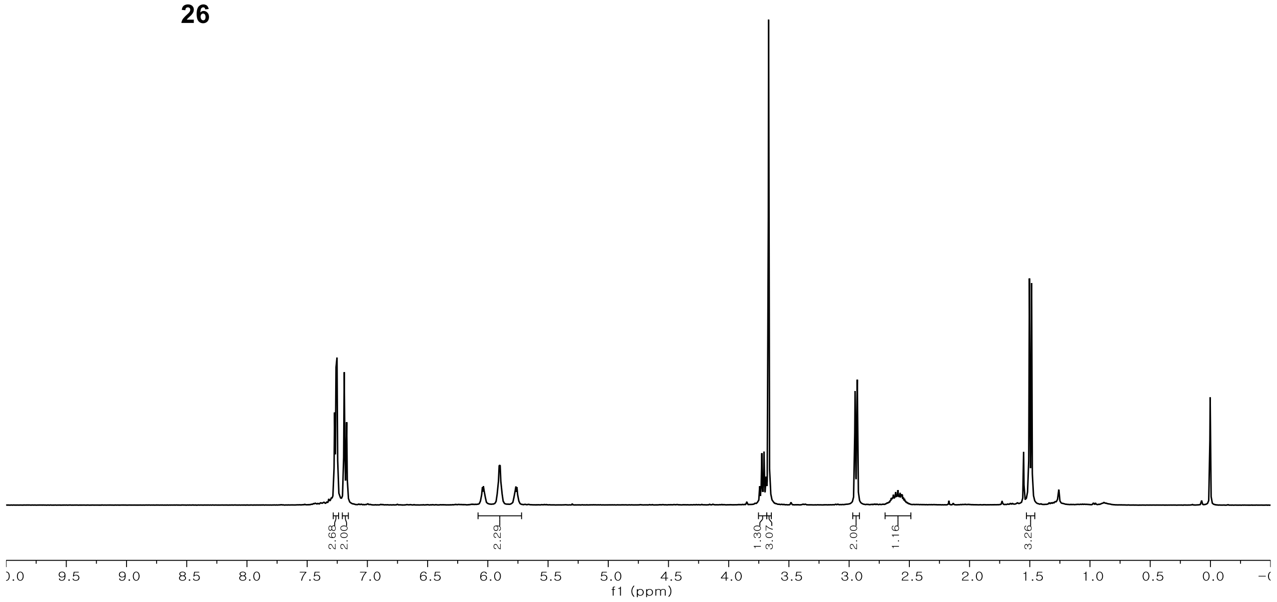

Supplementary Figure 165. ^{1}H NMR Spectrum of Methyl 2-(4-(2-(difluoromethyl)-3,3-difluoropropyl)phenyl)propanoate (**26**)

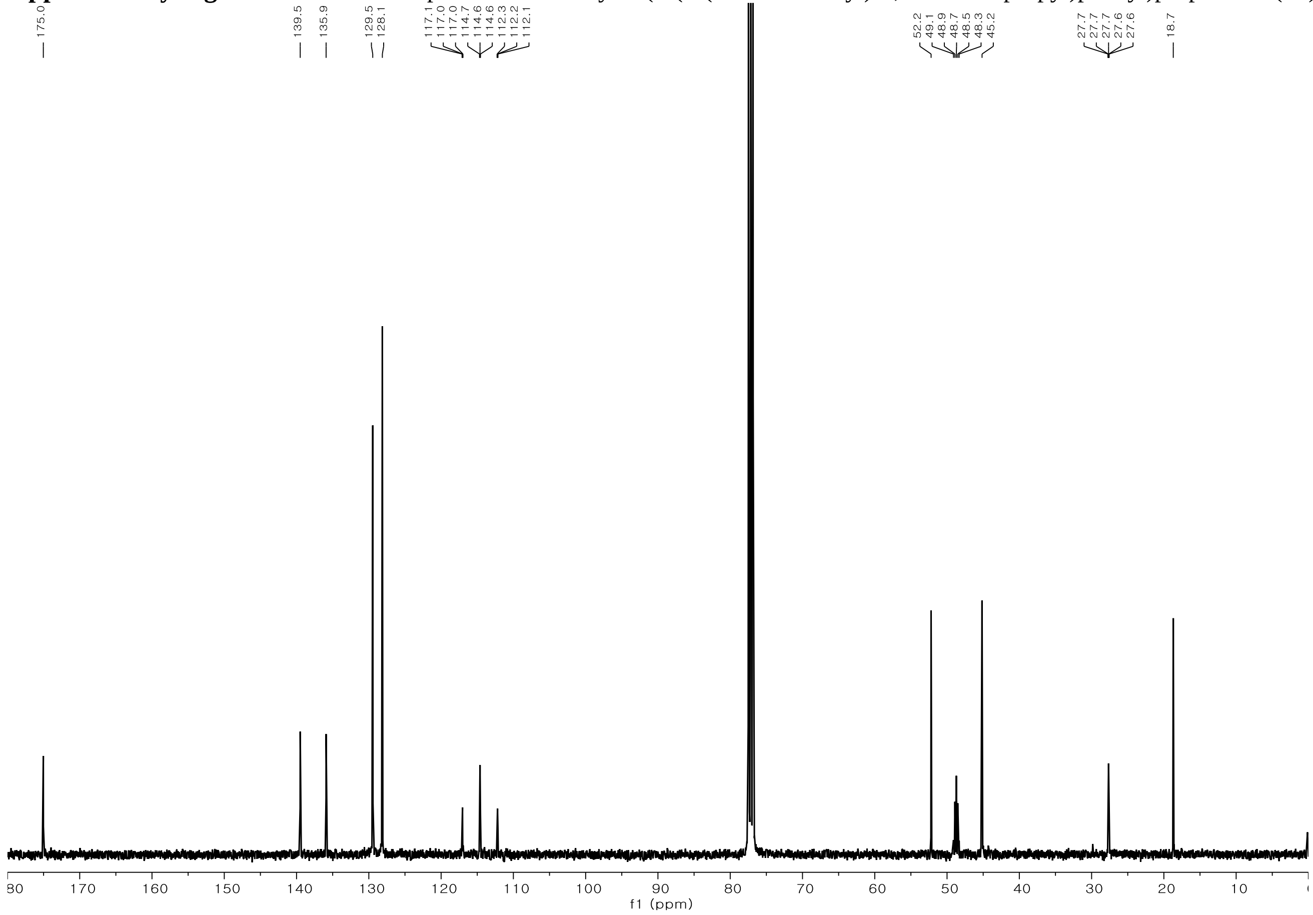

Supplementary Figure 166. ^{13}C NMR Spectrum of Methyl 2-(4-(2-(difluoromethyl)-3,3-difluoropropyl)phenyl)propanoate (**26**)

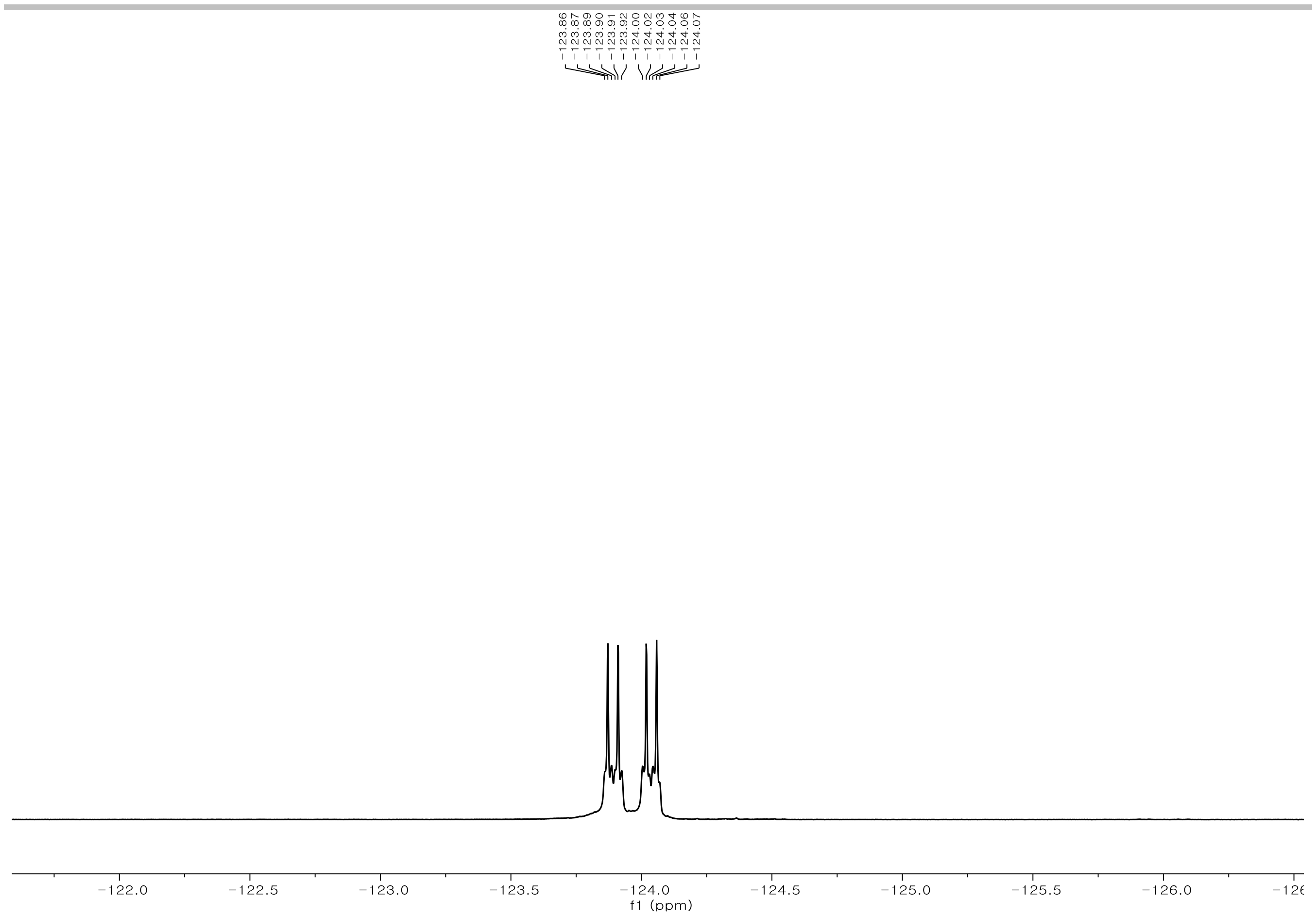

Supplementary Figure 167. ^{19}F NMR Spectrum of Methyl 2-(4-(2-(difluoromethyl)-3,3-difluoropropyl)phenyl)propanoate (**26**)

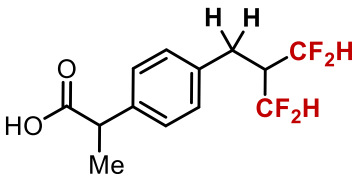

27

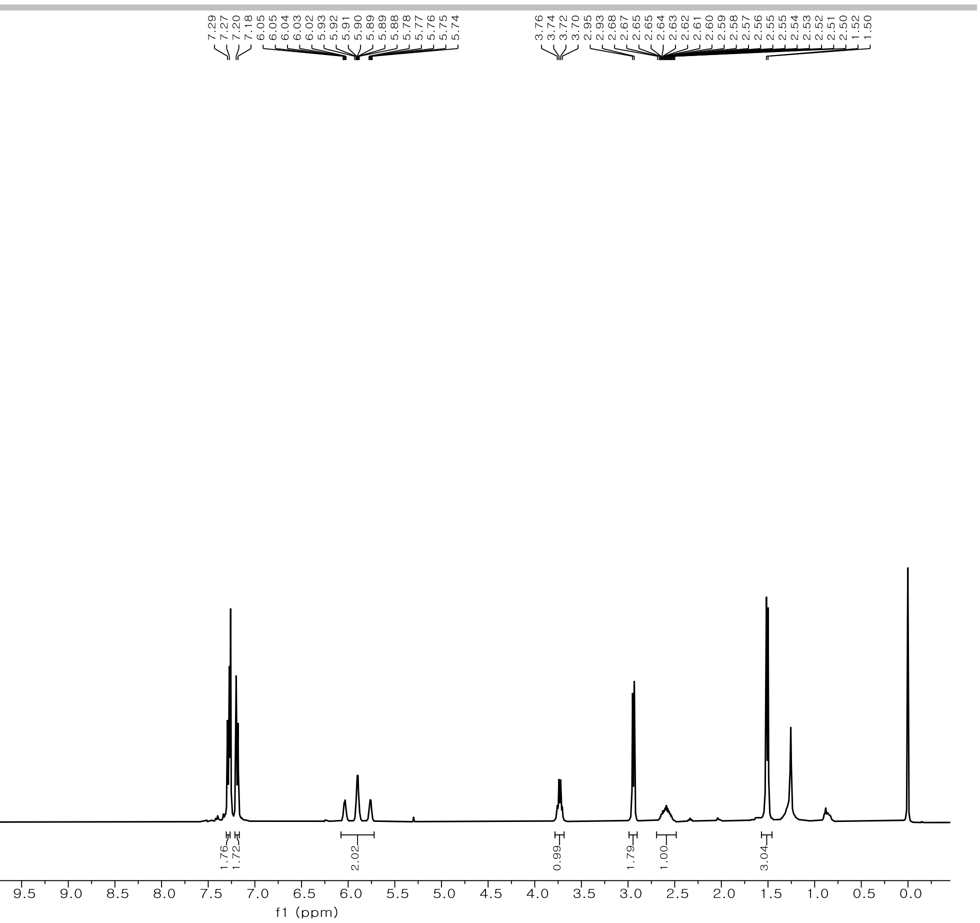

Supplementary Figure 168. ^{1}H NMR Spectrum of 2-(4-(2-(Difluoromethyl)-3,3-difluoropropyl)phenyl) propanoic acid (**27**)

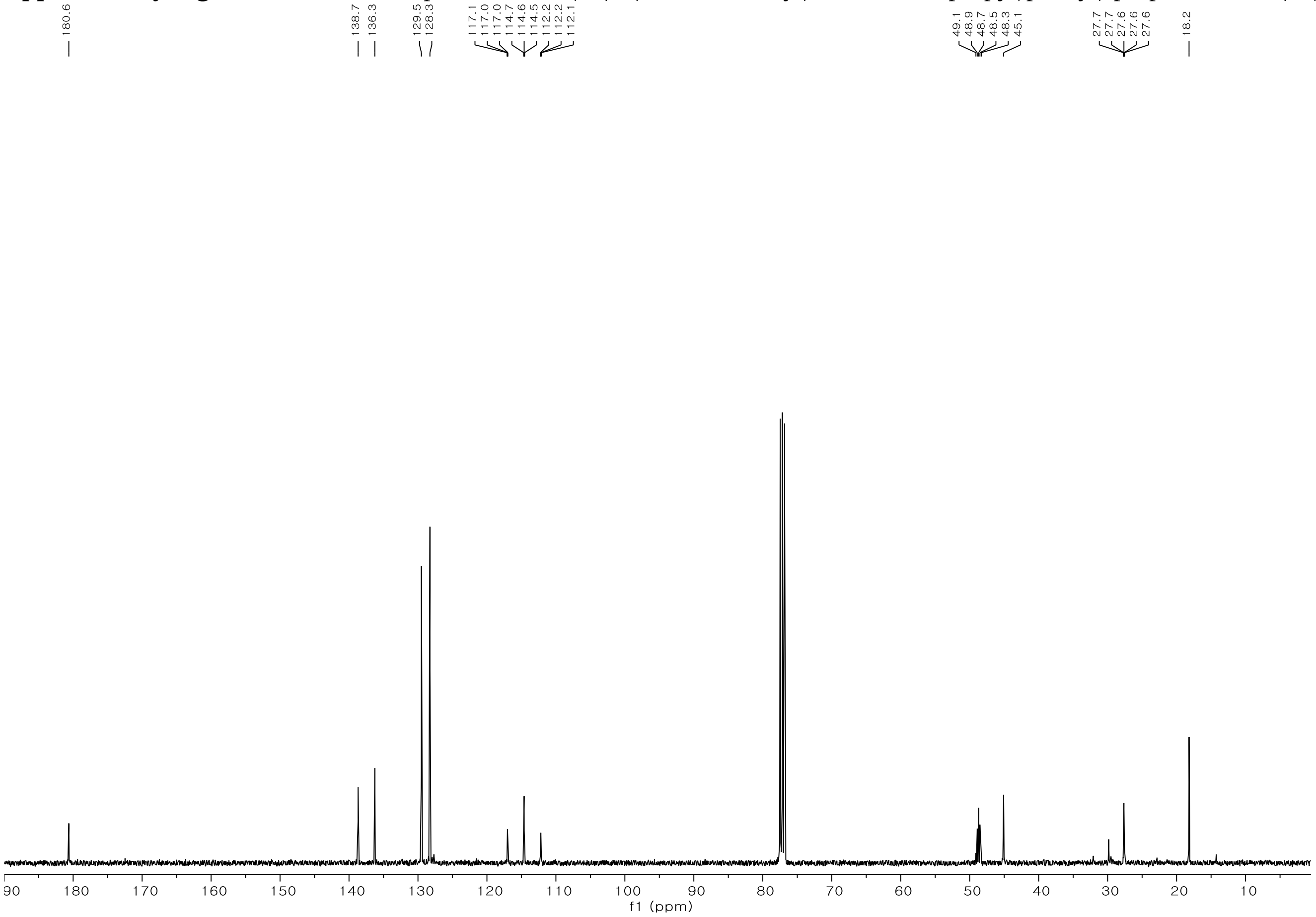

Supplementary Figure 169. ^{13}C NMR Spectrum of 2-(4-(2-(Difluoromethyl)-3,3-difluoropropyl)phenyl) propanoic acid (**27**)

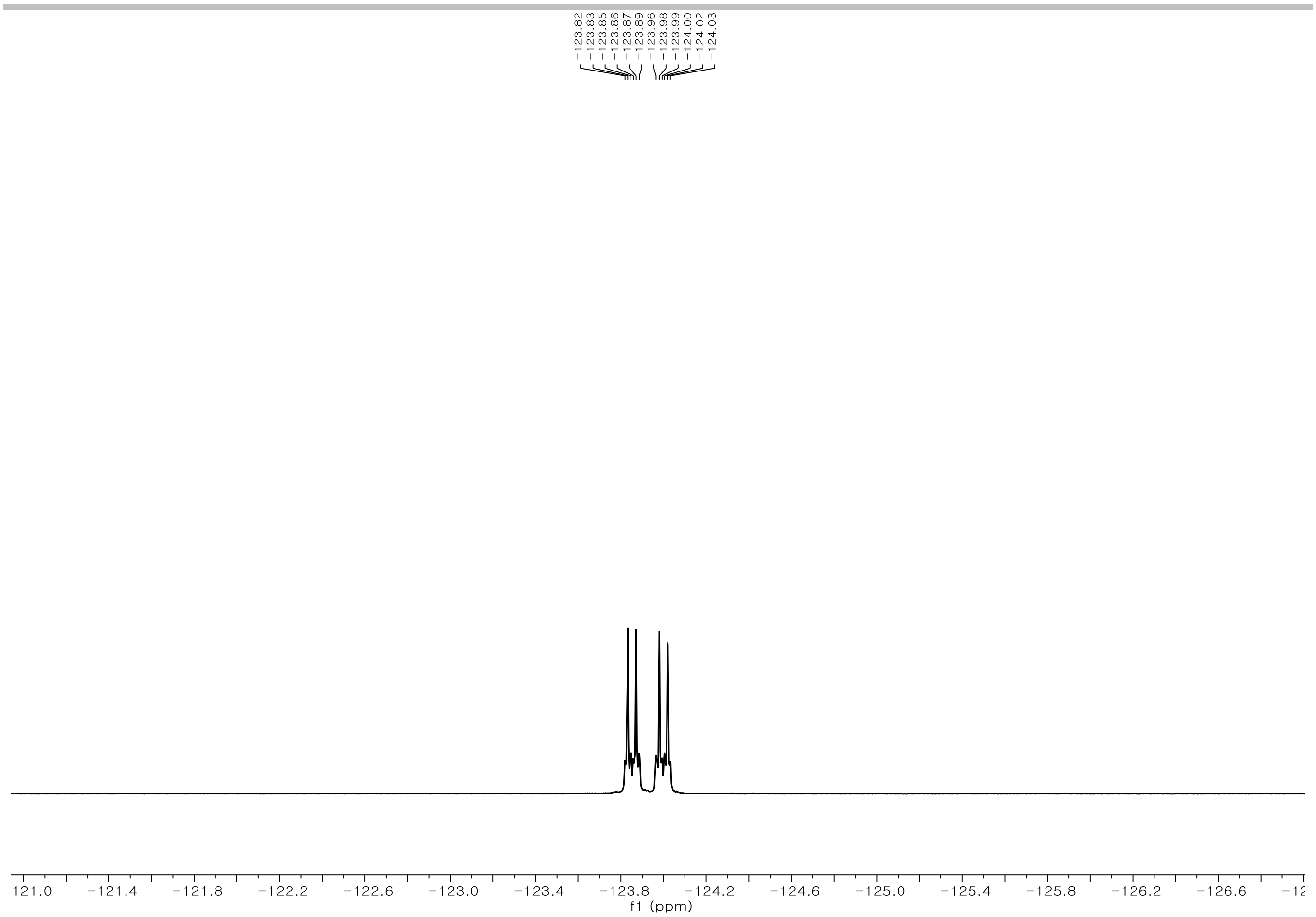

Supplementary Figure 170. ^{19}F NMR Spectrum of 2-(4-(2-(Difluoromethyl)-3,3-difluoropropyl)phenyl) propanoic acid (**27**)

Supplementary References

(1) Zhang, Z. Q., Meng, X. Y., Sheng, J., Lan, Q., Wang, X. S. Enantioselective Copper-Catalyzed 1,5-Cyanotrifluoromethylation of Vinylcyclopropanes. *Org. Lett.* **21**, 8256-8260 (2019).

(2) Heberlig, G. W. *et al.* Chemoenzymatic macrocycle synthesis using resorcylic acid lactone thioesterase domains. *Org. Biomol. Chem.* **16**, 5771-5779 (2018).

(3) Bertrand, X., Paquin, J. F. Direct Hydrofluorination of Methallyl Alkenes Using a Methanesulfonic Acid/Triethylamine Trihydrofluoride Combination. *Org. Lett.* **21**, 9759-9762 (2019).

(4) Liu, Y. *et al.* Zinc-Mediated Intermolecular Reductive Radical Fluoroalkylsulfination of Unsaturated Carbon–Carbon Bonds with Fluoroalkyl Bromides and Sulfur Dioxide. *Chem. Eur. J.* **25**, 1824-1828 (2019).

(5) Dorn, S. K., Tharp, A. E., Brown, M. K. Modular Synthesis of a Versatile Double-Allylation Reagent for Complex Diol Synthesis. *Angew. Chem. Int. Ed.* **60**, 16027-16034 (2021).

(6) Shing, T. K. M., Zhong, Y. L. Syntheses of medium-sized cyclic ethers from carbohydrates via an intramolecular nitrile oxide-alkene cycloaddition strategy. *Synlett* **13**, 1205-1208 (2006).

(7) Zhong, C., Wang, Y., Hung, A. W., Schreiber, S. L., Young, D. W. Diastereoselective control of intramolecular aza-Michael reactions using achiral catalysts. *Org. Lett.* **13**, 5556-5559 (2011).

(8) Li, X., He, S., Song, Q. Diethylzinc-Mediated Radical 1,2-Addition of Alkenes and Alkynes. *Org. Lett.* **23**, 2994-2999 (2021).

(9) Xu, C., Huang, W., Zhang, R., Gao, C., Li, Y., Wang, M. Trifluoromethylations of Alkenes Using PhICF3Cl as Bifunctional Reagent. *J. Org. Chem.* **84**, 14209-14216 (2019).

(10) Schmidt, B., Krehl, S., Jablowski, E. Assisted tandem catalytic RCM-aromatization in the synthesis of pyrroles and furans. *Org. Biomol. Chem.* **10**, 5119-5130 (2012).

(11) M. Allegretti, *et al. J. Med. Chem.* **48**, 4312-4331 (2005).

(12) E. Isabel, K. P. *et al. Bioorg. Med. Chem. Lett.* **20**, 887-892 (2010).

(13) Lin, Q. Y., Xu, X. H., Zhang, K., Qing, F. L. Visible-Light-Induced Hydrodifluoromethylation of Alkenes with a Bromodifluoromethylphosphonium Bromide. *Angew. Chem. Int. Ed.* ***55***, 1479-1483 (2016).
