# Supplementary material for: Radical hydrodifluoromethylation of unsaturated C−C bonds via an electroreductively triggered two-pronged approach
Source: Commun Chem. 2022 Aug 11;5:96. doi: 10.1038/s42004-022-00697-1 (PMC9814520; doi:10.1038/s42004-022-00697-1)
Supplement: Supplementary file 3 — Description of Additional Supplementary Files [file 42004_2022_697_MOESM3_ESM.docx]

Description of Additional Supplementary Files

**File name:** Supplementary Data 1

**Description:** All Figures as Chemdraw Files.
